# Anomalous Dynamics of a Lipid Recognition Protein on a Membrane Surface

**DOI:** 10.1038/srep18245

**Published:** 2015-12-14

**Authors:** Eiji Yamamoto, Antreas C. Kalli, Takuma Akimoto, Kenji Yasuoka, Mark S. P. Sansom

**Affiliations:** 1 Department of Mechanical Engineering, Keio University, Yokohama, Kanagawa, 223-8522, Japan; 2 Department of Biochemistry, University of Oxford, South Parks Road, Oxford, OX1 3QU, United Kingdom

## Abstract

Pleckstrin homology (PH) domains are lipid-binding modules present in peripheral membrane proteins which interact with phosphatidyl-inositol phosphates (PIPs) in cell membranes. We use multiscale molecular dynamics simulations to characterize the localization and anomalous dynamics of the DAPP1 PH domain on the surface of a PIP-containing lipid bilayer. Both translational and rotational diffusion of the PH domain on the lipid membrane surface exhibit transient subdiffusion, with an exponent *α* ≈ 0.5 for times of less than 10 ns. In addition to a PIP_3_ molecule at the canonical binding site of the PH domain, we observe additional PIP molecules in contact with the protein. Fluctuations in the number of PIPs associated with the PH domain exhibit *1/f* noise. We suggest that the anomalous diffusion and long-term correlated interaction of the PH domain with the membrane may contribute to an enhanced probability of encounter with target complexes on cell membrane surfaces.

Association of peripheral proteins with target lipids and/or proteins in cell membranes is essential for many trafficking and signaling events. This association often occurs via lipid binding modules such as pleckstrin homology domains (PH) found on peripheral proteins^1^,^2^. PH domains are a structurally conserved family, containing approximately 100 residues with an antiparallel β-sheet followed by one or two α-helices, but there is a rather low sequence identity between different PH domains. Many PH domains are localized to cell membranes via interactions of a positively charged loop with phosphatidyl-inositol phosphates (PIPs) in the membrane^3^,^4^. PH domains differ in their specificity for PIPs^5^,^6^.

Currently there are more than 150 structures of PH domains deposited in the protein data bank, with about a dozen structures of the PH domain in a complex with an inositol phosphate (InsP), i.e. the headgroup of a PIP lipid molecule. Although there are a wealth of structural, biophysical and functional data, our quantitative understanding of the exact process by which PH domains diffuse within membranes remains incomplete. In particular, despite significant advances from biophysical and computational studies of PH^7^,^8^,^9^ and other membrane-recognizing^10^,^11^ domains, we do not fully understand the dynamic consequences of the interaction of PH domains with PIPs and the likely functional implications of these interactions.

Molecular dynamics (MD) simulations provide a powerful computational tool for investigating membrane protein-lipid interactions. Recent studies have shown that MD simulations can be used to investigate the molecular mechanism of association of peripheral proteins with membranes^10^,^12^,^13^,^14^,^15^. In particular, this *in silico* approach provides insights into the molecular details of the localization, penetration and diffusion of peripheral proteins on a membrane.

The binding mode and orientation of peripheral proteins on a membrane surface have been studied in some detail (e.g.^7^,^9^). In contrast, the diffusive dynamics of peripheral proteins bound to the surface of a membrane is complex and less extensively explored^8^,^11^. Both tightly bound lipids and protein penetration into the bilayer core influence diffusion of peripheral membrane proteins^8^. This suggests that the diffusion of bound peripheral membrane proteins is likely to be determined by their interactions with lipids. Furthermore, a number of studies have indicated anomalous diffusion of lipids within membranes^16^,^17^,^18^,^19^. Furthermore, Weigel *et al.* used single molecule tracking to demonstrate that integral membrane proteins also may exhibit anomalous dynamics within the membrane^20^. Thus we might expect to observe anomalous diffusion of membrane-bound peripheral proteins. To study the diffusion of peripheral proteins we have used “computational” single molecule tracking via molecular dynamics simulations. This allows us to employ a comparable analysis to that employed in experimental single molecule tracking in order to provide novel insights into the diffusion of the intrinsically more complicated example of peripheral membrane proteins involved in recognition of PIP molecules in a cell membrane. Because the temporal and spatial patterns of localization of peripheral proteins on membranes are central to their biochemical activity, the diffusional behavior of bound PH domains may be expected to be linked to their functional roles in the cell. This makes it important to more fully understand the diffusional behavior of peripheral proteins bound to membranes, both from a biophysical and from a functional perspective.

Diffusion is often characterized by the ensemble-averaged mean square displacement (MSD), *i.e.*, (*r*^*2*^(*t*)) = 2*dDt*, where *D* is the diffusion constant and *d* is the dimension. However, for example single-particle tracking experiments have shown subdiffusion to occur in living cells 20,21,22,23,24,


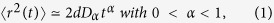


where *α* is the subdiffusive exponent and *D*_*a*_ the generalized diffusion constant. Indeed, “anomalous” dynamic behavior such as subdiffusion is widely observed in biological systems^25^. In the context of peripheral membrane proteins, it is notable that subdiffusive motions are observed in diffusion of lipid molecules^16^,^17^,^18^,^19^ and of water molecules on the surface of a membrane^26^,^27^,^28^,^29^. Interpretations of experimental studies have invoked a number of explanations of anomalous diffusion in complex cell membranes, including molecular crowding and/or the presence of lipid rafts. However, recent theoretical and computational studies have suggested that anomalous diffusion of water and of lipids may arise in relatively simple membranes composed of a single species of lipid^17^,^18^,^19^,^27^. In this study we will focus on a single PH domain (i.e. there is no crowding of membrane proteins) in a relatively simple model membrane which does not include lipid rafts. However, we do incorporate an element of biological complexity by employing a mixed lipid bilayer which contains PIP molecules. We use this system to explore in detail the molecular origins of anomalous diffusion of a PH domain bound to a membrane surface.

In addition to subdiffusion, a more enigmatic aspect of anomalous dynamics is the frequent observation of 1/*f* noise in power spectra *S*(*f*):


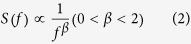


*1/f* noise has been observed in a range of biological processes, including e.g. protein conformational dynamics^30^,^31^, ionic currents through membrane channels^32^, biorecognition^33^, and in the hydration dynamics on lipid membrane surfaces^34^. It has been shown that *1/f* noise underlies the anomalous dynamics of water on a membrane surface. Therefore it is possible that other molecules bound to a membrane surface may exhibit *1/f* noise, and so it is timely to also determine whether comparable anomalous dynamics are observed for proteins bound to a lipid bilayer. It is also possible that *1/f* noise in combination with anomalous diffusion reflect optimization of the process whereby peripheral proteins search for their interaction partners in a membrane, e.g. PIP lipids and/or other proteins^35^.

In this study, we use a serial multiscale simulation approach^36^, combining coarse-grained (CG-MD)^37^ and atomistic molecular dynamics (AT-MD) simulations, to investigate the localization, interactions and dynamics of PH domains on model lipid membrane surfaces. We show that the PH/PIP complex obtained from our simulations is in good agreement with the PH/InsP complex obtained by X-ray crystallography. Extensive CG-MD simulations (totaling 0.25 msec of simulation time) enable us to characterize in detail the dynamics of the PH domains on the membrane. We observe transient subdiffusion in the translational and rotational motions of the PH domain on the membrane surface. Moreover, we show that the interaction between the PH domain and PIP molecules in the membrane exhibits *1/f* noise, and that this is a consequence of a power-law distribution of residence times along with long-term correlation between residence times. We are thus able to propose a simple model whereby *1/f* noise from the power-law distribution of residence times corresponds to an increased residence time of the peripheral protein on the membrane surface, which in turn may facilitate searching for a target protein or complex.

## Results

### Association of PH domains with a PIP-containing bilayer

**Figure 1:**
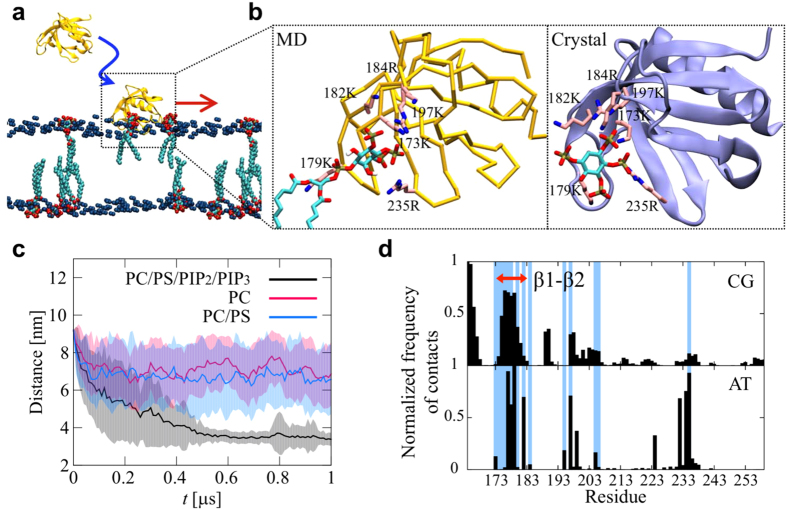
Interaction between the DAPP1 PH domain and PIP molecules. (**A**) Montage formed from snapshots of a simulation to illustrate the binding of the DAPP1 PH domain to PIP_3_-containing lipid bilayer. PIP_3_ molecules are shown in cyan/red and the phosphate atoms of the POPC and POPS lipids are shown as blue spheres. The simulation system and snapshots are shown in Fig. S1 and S2. (**B**) The left inset picture shows a snapshot of the PH/PIP_3_ complex derived (and converted to an atomistic model) from our CG simulations [PH domain (yellow) and PIP_3_ (cyan/red/bronze)]. Right inset is the crystal structure [PDB: 1FAO; PH domain (ice blue) and Ins(1,3,4,5)P_4_ (cyan/red/bronze)]. (**C**) Mean distances (plus/minus standard deviation represented by the transparent colours) between the center of mass of the DAPP1 PH domain and the lipid bilayer for simulations with either a PC/PS/PIP_2_/PIP_3_ (black), PC (red), or a PC/PS (blue) bilayer. The data are averaged over 25 repeat CG simulations. (**D**) Normalized average number of contacts between the protein and PIP_3_ shown for the 25 × 1 μs CG simulations and for the 2 × 1 μs AT simulations. The light blue colours represent the experimental contacts observed in the crystal structure (cutoff distance 0.4 nm). For the normalization, the number of contacts of a residue with a lipid type was divided by the largest number of contacts that the same lipid type made with any residue in the protein. This means that the residue with the most frequent contacts will have the value of 1 and the residue with no contacts with a lipid type will have the value of 0. The position of the β1-β2 loop which binds PIP_3_ is shown by a red arrow. Comparable analysis of PIP_2_ interactions is shown in Fig. S3.

To investigate the molecular mechanism of association of PH domains with PIPs, we have performed CG-MD simulations using the DAPP1 PH domain crystal structure (PDB id 1FAO)^38^ (details of all simulations are given in Table 1). In the initial configuration of the simulations the PH domain was displaced ca. 9 nm away from a preformed lipid bilayer, and 25 repeat simulations were run for 1 μs starting from different initial orientations of the PH relative to the bilayer. During the simulations the PH domain diffuses in the aqueous solution before forming a complex with the lipid bilayer [see Fig. 1A,B]. The progress of this can be measured by the distance between the center of mass (COM) of the DAPP1 PH domain and the lipid membranes [Fig. 1C]. When PIP lipids were present in the bilayer, the PH domain associates with the membrane in all (25 out of 25) of the simulations. In contrast, when the PH domain was simulated with either a zwitterionic (PC) or an anionic but PIP-free (PC/PS 80:20) lipid bilayer, no PH/bilayer complex was formed [Fig. 1C]. This indicates that PIP molecules are needed for the association of the PH domain with the membrane, as is seen to be the case experimentally^39^.

Comparisons across the ensemble of 25 simulations revealed two modes of interaction of the PH domain with the membrane. In one mode (which was the more frequently observed) the PH domain associated with the bilayer via a positively charged loop located between β1 and β2 strands, e.g. K179, in good agreement with the available structural data. The PH/PIP complex corresponding to this major mode of interaction in our simulations is very similar to the crystal structure of DAPP1 PH bound to Ins(1,3,4,5)P4, i.e. the headgroup of PIP_3_ [see Fig. 1B]. In the second mode of interaction (which was observed less frequently) the protein interacted with the membrane via the opposite surface of the PH domain to that seen in the major mode, and PIP lipids did not associate with the binding site seen in the crystal structure. Analysis of the protein/PIP_3_ contacts averaged across the whole ensemble simulations [Fig. 1D and Fig. S3] agrees well with the principal Ins(1,3,4,5)P4 contacts to the PH domain seen in the DAPP1 crystal structure^38^ with the major contacts around residues 173-184. For this analysis we use a cutoff distance of 0.7 nm which approximately corresponds to contact of two CG particles and 0.4 nm for AT particles. Overall these results suggest that our CG-MD simulations yield a PH/PIP complex similar to that observed in the crystal structure of the protein/lipid headgroup.

We performed two extended (2 × 1 *μ*s) AT-MD simulations of the DAPP1 PH/PIP_3_ complex derived from the endpoint of the CG-MD simulations, corresponding to the major mode of interaction (see above) with the PIP_3_ molecule in the binding site also observed in the crystal structure. In one of the extended atomistic simulations, interactions of the PH domain with PIP_3_ were retained over the course of the 1 *μ*s simulation [see Fig. 1D and Fig. S2]. In the other AT-MD simulation we observed dissociation of PIP_3_ from the binding site of the PH domain after ca. 250 ns simulation, but dissociation of the PH domain from the bilayer was not observed within the 1 *μ*s duration of the simulation.

### Translational and rotational diffusion of PH domains on lipid bilayer surface

Having established the mechanism of association of the PH domain with the lipid bilayer, we extended the simulations of the DAPP1 PH domain with PIP-containing bilayers to 10 *μ*s each (overall 0.25 msec; see Table 1). This allowed us to study in detail the diffusion dynamics and motion of the PH domain. Previous experimental studies using e.g. single molecule TIRF microscopy have suggested that peripheral proteins undergo Brownian motion when they are associated with lipids^7^,^8^,^11^.

**Figure 2:**
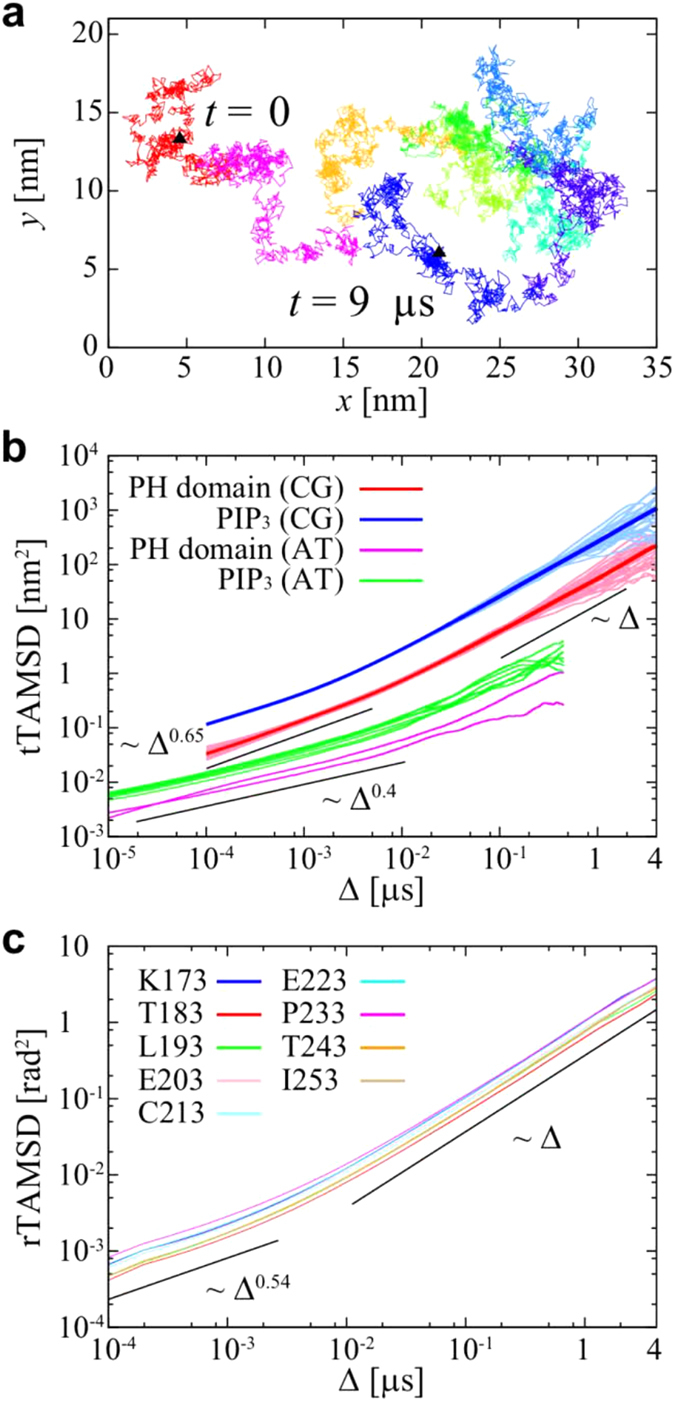
Translational and rotational diffusion of DAPP1 PH domain on the surface of a PC/PS/PIP_2_/PIP_3_ bilayer. (**A**) Lateral trajectory of a protein tracked for 9 μs on the membrane surface. The color of trajectory (from red to blue) represents the timescale, with a change in color every 1 μs. The black triangles indicate *t* = 0 and 9 μs. (**B**) Translational TAMSDs of the DAPP1 PH domain and PIP_3_ molecules; 25 TAMSDs for CG and 8 TAMSDs for PIP_3_ (AT) and 2 TAMSDs for PH domain (AT). The deep colored solid lines of CG show the mean TAMSDs over all 25 CG-MD trajectories. The TAMSDs of the PIP_3_ molecules were calculated for the lipids in the lower bilayer leaflet i.e. the opposite leaflet from that to which the protein is bound. The black solid lines are shown for reference to indicate the subdiffusive exponent (*α* in eq. 1 in the text). The transient subdiffusion of the PH domains is observed in both the AT-MD and CG-MD simulations. (**C**) Rotational mean TAMSDs of all 25 trajectories on the PC/PS/PIP_2_/PIP_3_ membrane surface. The colored lines represent the vectors to different amino acids. The solid lines are shown for reference.

To study the translational diffusion of the DAPP1 PH domain we tracked the protein after it bound to the bilayer. The protein was considered bound to the bilayer when the distance between the COM of the protein and the COM of the bilayer was less than 4 nm. For this calculation we have discarded the first 100 ns after binding to remove the initial non-equilibrium state. Additionally we also subtracted the COM of the associated leaflet from the trajectories of the protein^17^,^19^,^27^,^40^. Figure 2A shows the lateral trajectory of one of the simulations.

We have calculated the translational time-averaged mean square displacements (tTAMSD) defined by 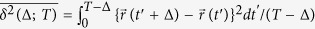, *T* is the measurement time, and 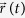 is the position of the tracked particle at time *t*. The tTAMSD of the protein on the lipid membrane surface clearly exhibits transient subdiffusion [see Fig. 2B]. The power-law exponent *α* (see equation 1 above), which was calculated by fitting the ensemble averaged tTAMSD, changes from 0.65 (subdiffusive) to 1.0 (diffusive) around a crossover point at 10 ns. The scaling exponent α as a function of time is shown in Fig. S4. This is similar to the anomalous diffusion observed for lipid molecules which also exhibited a crossover point around 10 ns^19^,^40^. Significantly we see a similar crossover point for the (unbound) PIP_3_ molecules in the bilayer, although their absolute diffusion coefficient is about 5-fold faster. This would be consistent with a model in which the PH domain interacted with 4 or 5 PIP_3_ molecules. We note that Knight & Falke^7^ using tandem constructs of PH domains have shown that the lateral diffusion constants of these proteins are inversely proportional to the number of bound PIP_3_ molecules. Thus we may conclude that on the membrane surface the (bound) lipid motion largely determines the diffusive dynamics of the PH domain (as suggested in^7^). After the crossover time, the diffusion of the PH domain becomes normal Brownian motion. At *t* = 1 μs, the relative standard deviation, 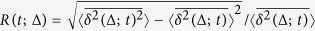, is 0.1, which is smaller than that of a continuous time random walk^25^.

Significantly, similar transient subdiffusion was also observed in our extended AT-MD simulations of the PIP-bound form of the PH domain. Although the diffusive dynamics of the PH domain and of the lipids in the CG-MD simulations is approximately an order of magnitude faster than those of the AT-MD simulations (a well known property of the MARTINI force field as discussed by e.g.^41^,^42^), the fundamental phenomenon of transient subdiffusion is the same. In both the CG and the AT simulations, the crossover point times of the TAMSDs are at the same order of magnitude. This suggests that while the quantitative values are not the same due to the difference in the resolution of the underlying lipid models, the trends in transient subdiffusion are preserved upon coarse-graining. The quantitative discrepancy is likely to be related to the simplifications inherent in the CG representation which may result in a degree of smoothing of the energy landscape for the protein/bilayer interaction. Moreover, we have determined the diffusion coefficient *D* from the mean TAMSD using linear region. The *D* of the DAPP1 PH domain from the CG-MD simulations is about 14 μm^2^/s which agrees well with the diffusion coefficient of the GRP1 PH domain as measured experimentally (*D* = 3 μm^2^/s on a PC/PIP_3_ or *D* = 2 μm^2^/s on a PC/PS/PIP_3_ bilayer^7^) once one takes into account the known CG speed-up (which is in many studies taken implicitly into account as discussed in e.g.^41^).

We have also performed comparable simulations and analysis with the PH domain of protein kinase B (PDB id 1UNQ)^43^, which also is known to bind PIP_3_ molecules. Analysis of these simulations also reveals transient subdiffusion and similar exponent α [see Fig. S5]. This suggests that anomalous diffusion may be a universal property of the diffusion of PH domains (and perhaps other peripheral proteins) on a membrane surface, arising from the properties of the long-term correlated motion of lipid molecules with which they form strong interactions.

In addition to examining translational diffusion, we measured the rotational dynamics of the DAPP1 PH domain on the membrane surface (see SI for details). The rotational TAMSD^27^,^44^ of the PH domain on the PIP-containing lipid membrane surface also exhibits transient subdiffusion [see Fig. 2C]. The power-law exponent for this rotational motion switches from 0.54 to 1.0 around a crossover point at 10 ns. This indicates that rotational and translational motions of the PH domain on the membrane are correlated, reflecting the same underlying dynamic nature of the PIP-mediated PH domain/bilayer interactions. We also examined rotational TAMSDs of PIP molecules [see Fig. S6]. Interestingly, there were no significant differences in rotational motion between PIP molecules bound and or unbound to the DAPP1 PH domain.

### Fluctuations of interaction between PH domain and PIPs

**Figure 3:**
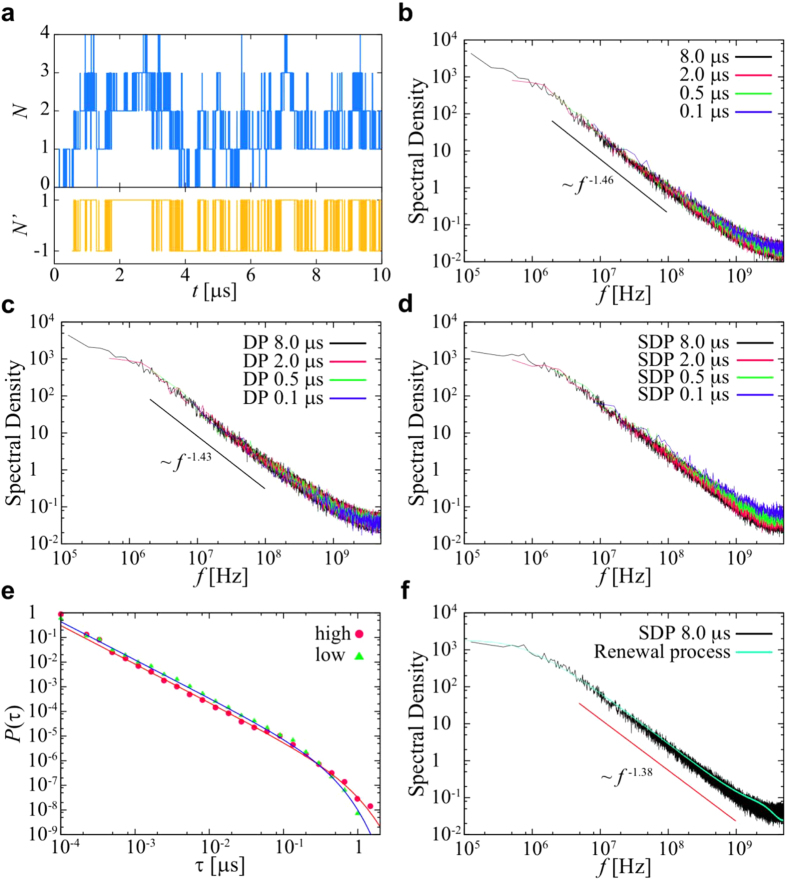
1/f noise of the interaction between PIP_3_ and the DAPP1 PH domain. (**A**) Time series of the number of PIP_3_ molecules around (using a cutoff distance of 0.7 nm) the protein (blue) and of the dichotomous process (DP; yellow). In the dichotomous process we consider the PH domain to be in a “high” (*N’ = *1) state when the number of PIP_3_ molecules around the protein is above the average and “low” (*N’* = −1) state otherwise. (**B**) Ensemble-averaged power spectral density (PSD) of 25 time series of the number of PIP_3_ around the PH domain. The different colored lines represent different measurement times and their power spectra coincide without fitting. The solid lines are shown as reference for higher and lower frequencies. (**C**) Ensemble-averaged PSD of the time series of dichotomous process of the number of PIP_3_ molecules around the protein. (**D**) Ensemble-averaged PSD of the shuffled dichotomous process (SDP) with different measurement times. (**E**) PDFs of the residence times of the “high” and “low” PIP states. The probability density functions (PDFs) of residence times of the protein in the “high” (red circles) and “low” (green triangles) states are fitted by power-law distributions with exponential cutoffs (solid lines): *P*(*τ*)* = Aτ*^*−1−γ*^ exp(*−τ/τ*_*c*_) (high: *γ* = 0.57 and *τ*_*c*_ = 600 ns; low: *γ* = 0.55 and *τ*_*c*_ = 300 ns). (F) Ensemble-averaged PSD of the SDP (black line). Numerical simulation of alternating renewal process; residence times are given by power-law distribution with exponential cutoff, where *γ* and *τ*_*c*_ are the same as observed values (green line).

We have shown above that PIP lipids determine the localization of the PH domains to the bilayer, and that diffusional dynamics are consistent with the PH domain co-diffusing with 3–7 bound PIP molecules. From the simulations we may also directly estimate the number of PIP molecules which associate with the protein during the extended simulations. For this analysis we use a cutoff distance of 0.7nm which approximately corresponds to contact of two CG particles. This yields values of: PIP_2_, 3.6 ± 1.2; PIP_3,_ 1.4 ± 0.9; and PIP_2_ + PIP_3_, 5.0 ± 1.2). The probability of the number of PIPs around the PH domain is shown in Fig. S8. We note that there are 9 PIP_2_ molecules and 4 PIP_3_ molecules in each leaflet of the bilayer in this simulation (corresponding to 5% and 2% of the total lipids respectively, which is close to the physiological composition of a ‘typical’ mammalian plasma membrane). This analysis suggests that the number of PIP_3_ molecules around the protein fluctuates from 0 to 4 over the course of a simulation (out of the 4 PIP_3_ molecules in each bilayer leaflet) [see Fig. 3A]. Figure 3B shows the ensemble-averaged power spectral density (PSD) obtained from averaging 25 power spectra for the number of PIP_3_ molecules associated with the DAPP1 PH domain. For this calculation we have used trajectories only when the distance between the COM of the protein and the bilayer was less than 4 nm (i.e. when the PH was bound to the bilayer) and discarded the first initial non-equilibrium state (100 ns) after binding. Analysis of the PSD reveals *1/f* noise with a power-law exponent *β* = 1.46 ± 0.06 at high frequencies. The standard deviation is calculated from fitted exponents of every 25 PSDs. The PSDs for the numbers of molecules of PIP_2_, PS, or PC around the PH domain are shown in Fig. S9 and S10. These reveal a trend in which the power law exponent decreases from *β* = 1.48 for PIP_2_ to *β* = 1.0 for PC, suggesting that the exponent is smaller for those lipids (PC) which interact less strongly with the PH domain.

We have also calculated the ensemble-averaged PSDs for different measurement times. In this analysis we used trajectories after first discarding the initial 0.1 μs non-equilibrium state after the protein has bound to the membrane surface. For example, in the case of the 2 μs measurement time, we used trajectories from *t′* = 0.1 μs to *t′* = 0.1 + 2 μs after binding to the membrane surface at *t′* = 0 μs. Thus, the number of the ensembles for calculating PSDs is the same. The magnitudes of the PSDs do not depend on the measurement time i.e. suggesting there is no ageing [Fig. 3B]. We have also confirmed that the fluctuation in the number of PIPs, POPC and POPS molecules within a circular area in the opposite leaflet from that to which the protein was bound shows 1/*f* noise at high frequencies [Fig. S11]. However, the range of this 1/*f* noise is much shorter than that for the PH domain. Thus, although the 1/*f* noise is an inherent feature of any lipid bilayer, the interactions between the PH domain and lipid molecules are responsible for the more long-term 1/*f* noise.

To further investigate the *1/f* noise, we have constructed a dichotomous process from the time series of the number of PIP_3_ molecules which are in contact with the protein. In this process we consider two states: a “high” state (*N*′ = 1) in which the number of PIP_3_ molecules contacting the protein is above the average, and a “low” state (*N*′ = −1) when the number of PIP_3_ molecules is below the average. The PSD of the resultant dichotomous processes also exhibits *1/f* noise, with power-law exponent *β* = 1.43 ± 0.06 similar to that of the original time series and again no aging [see Fig. 3C]. The comparison of PSDs of the dichotomous processes using different values of the threshold *N*_*t*_ (i.e. a “high” state (*N’* = 1) if *N*_t_ < *N* and “low” state (*N’* = −1) if *N* ≤ *N*_t_), are shown in Fig. S12. When *N*_t_ is the same as the mean value, the magnitude of PSD had the highest value. If the *N*_t_ is different from the mean value, the residence time of one state becomes larger and that of the other state becomes smaller.

Subsequently, we have calculated the ensemble-averaged PSD for a shuffled dichotomous process, in which the residence times for the “high” and “low” states were randomly shuffled amongst themselves. This shuffling process removes the long-term correlation between residence times. After shuffling, the power-law exponent of PSD at high frequencies is *β* = 1.38 ± 0.06, whilst the PSD converges to a finite value at low frequencies [Fig. 3D]. Moreover, the PSD shows ageing at short measurement times. These phenomena are not observed if there is a long-term correlation in residence times, i.e. in the unshuffled process. This means that one of the origins of the 1/*f* noise is long-term correlation between residence times.

The probability density functions (PDFs) of residence times for “high” and “low” states are well fitted by power-law distributions with exponential cutoffs [see Fig. 3E]. The observed power-law exponent, *γ*, is less than 1, which implies divergence of the mean residence times. Based on these observations above, we performed a numerical simulation in which a time series of “high” and “low” (i.e. (*N*′ = 1 and -1) states was generated with random waiting times drawn from a power-law distribution with an exponential cutoff. In this alternating renewal process, the power-law exponent *β* in the PSD is given by the power-law exponent in the residence time distribution, *i.e.*, *β* = 2 − *γ* as *γ* < 2^45^. The ensemble-averaged PSD of the alternating renewal process using the observed values of *γ* and *τ*_c_ is consistent with the shuffled dichotomous process [Fig. 3F]. The power-law exponent *β* observed here in the PSD is in good agreement with this relationship. This implies that a further origin of the 1/*f* noise is the power-law distribution of residence times. Moreover, ensemble-averaged PSDs of the alternating renewal process at the measurement time below the exponential cutoff in the residence times are not consistent with those above the cutoff, i.e. there is ageing [Fig. S13]. This is in agreement with what we have seen for the shuffled dichotomous process with different measurement time [Fig. 3D]. The PSD does not show non-ergodic behavior (ageing) above the cutoff in the residence time distribution.

Taken together, these results suggest that the *1/f* noise originates from a combination of a power-law residence time and a long-term correlation in the residence times. It has been shown that lipid bilayers exhibit transient subdiffusion originating from fractional Brownian motion (FBM), which indicates viscoelasticity^17^,^18^,^19^. Thus our observation of long-term correlation in the residence times for PH/PIP interactions may reflect underlying long-term correlated motion of lipid molecules. We have repeated the same analysis for PIPs with the protein kinase B PH domain (see above), and confirmed that there is no significant difference in behavior from that seen for DAPP1 [see Fig. S14], suggesting that this behavior may be a shared property of PH domains bound to membranes.

**Figure 4:**
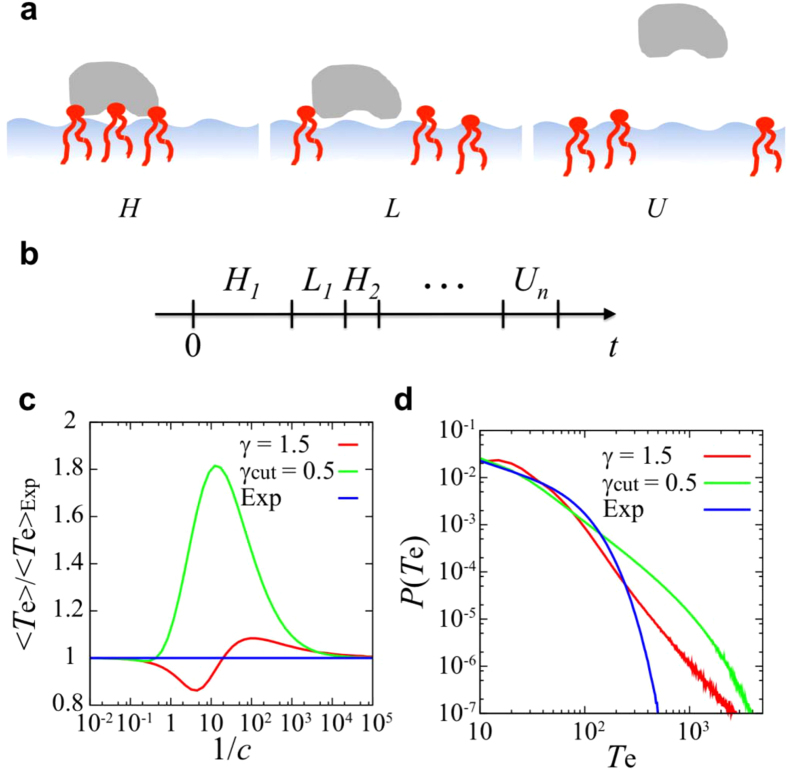
Stochastic modelling of the residence time of a PH domain on a membrane surface. (**A**) Schematic diagram of the molecular interpretation a stochastic model of the observed residence times of the PH domain on a membrane surface in which there are three states, H corresponding to a high number of interactions of the protein with PIP molecules, L corresponding to a low number of interactions of the protein with PIP molecules, and U is the unbound state. (**B**) Illustration of a residence time series for the stochastic models explore by numerical simulations (see Methods for details). In these models we explored three different PDFs: a power-law *P*_*H,L*_(*τ*) ∝ *τ*^*−1−γ*^ with *γ* = 1.5; a power-law with an exponential cutoff *P*_*H,L*_(*τ*) ∝ *τ*^*−1−γ*^exp(*−τ /τ*_*c*_ ) with γ = 0.5 and *τ*_c_ = 1000, and an exponential distribution *P*_*H,L*_(*τ*) ∝ exp(*−τ*), where the means of PDFs are set to be the same. The rate of dissociation of the protein from the bilayer (i.e. from state L to state U) in (**A**) is *c*, so that *1/c* is the mean dissociation time from the weak binding state. (**C**) Mean residence time (*T*_*e*_) on the bilayer normalized relative to that of an exponential distribution, with PDFs modelled as a power-law (red), or a power-law withe an exponential cutoff (green) as a function of the mean dissociation time *1/c.* Note that depending on the mean dissociation time *1/c*, the residence time deviates from that for an exponential distribution. (**D**) PDFs of residence times with *1/c* = 10.

What is the biophysical significance of the *1/f* noise observed in the fluctuation of the number of PIPs associated with a PH domain? In order to answer this, we may equate the observed dichotomous process (above) with a simple underlying model of the interaction of a PH domain on a PIP-containing membrane surface [Fig. 4A,B, details of the model are provided below in the Methods]. In this model we consider a two-state process, consisting of a ‘high PIP’ (i.e. high number of contacting PIP molecules) state and a ‘low PIP’ (i.e. low number of contacting PIP molecules) state of the PH domain. We assume that PH domain can dissociate from the membrane surface only from a ‘low’ state. In these models we explored three different PDFs for the residence time in the membrane bound states: a power-law, a power-law with an exponential cutoff, and a simple exponential distribution. We evaluate the dissociation time *T*_e_ of protein from the membrane. Depending on the mean dissociation time of the protein in the low state from the bilayer 1*/c* (i.e. *c* is the dissociation rate) the dissociation time *T*_e_ deviates from that for an exponential distribution to a greater or less extent [Fig. 4D and S15]. This helps to explain the apparent conflict between experimental studies^7^,^11^ suggesting exponential distributions of protein residence times on membrane surfaces and computational studies^26^,^27^ indicating that residence times of water molecules on the membrane follow a power law distribution. Moreover, this model implies that the power-law distributions of the bound states (as revealed by analysis of the *1/f* noise) provide an increase (relative to an exponential distribution) of the dissociation time of the proteins on the membrane surface [see Fig. 4C].

## Discussion

**Figure 5:**
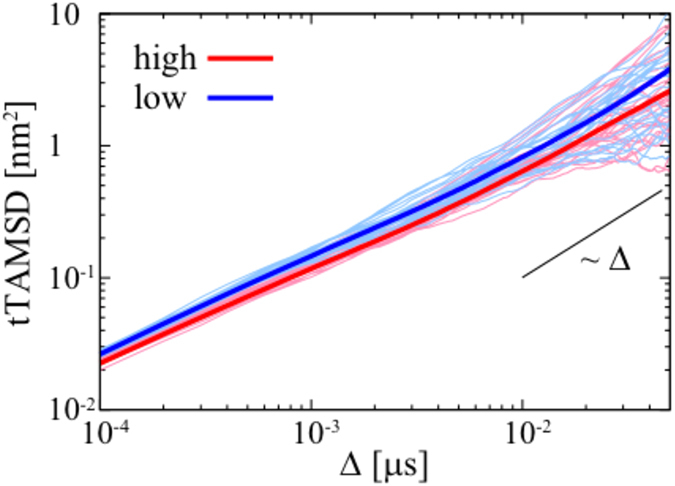
TAMSDs of the PH domain for the two states: “high” (red) and “low” (blue), where we used the trajectories which remain in the same state for more than 0.1 μs. 25 Randomly selected TAMSDs are shown as thin lines, and the deep colored solid lines show the corresponding mean TAMSDs. For the calculation of the mean TAMSDs, ensembles of 153 and 140 are used for ‘high’ and ‘low’ states, respectively. To evaluate the statistical significance, a t-test was performed with a significance level of 5%. The range of the population mean *μ* was [2.32, 2.88] for ‘high’ mean TAMSD and [3.32, 4.31] for ‘low’ mean TAMSD at Δ = 0.05 μs. We also note that the population means of the TAMSDs of each state do not cross.

Overall, we have shown that multiscale MD simulations yield a DAPP1 PH/PIP complex whose structure closely resembles that of the corresponding PH/InsP complex as determined by X-ray crystallography. This demonstrates the predictive capabilities of our approach for identification of the mode of association of a peripheral protein with cell membranes. Most strikingly, our simulations reveal anomalous diffusional dynamics of the PH domain on the surface of a PIP-containing lipid bilayer. Both the translational and the rotational diffusion of a PH domain on the lipid membrane surface exhibit transient subdiffusion. Furthermore, fluctuations of the number of PIPs associated with the PH domain exhibit *1/f* noise. Numerical simulations indicate that this may correspond to an underlying two state model of the PH domain when bound to the membrane surface, the two states differing in the number of PIP molecules associated with the protein. We have also shown that the diffusivity of the PH domain depends on the number of PIPs interacting with the PH domain [see Fig. 5], such that the diffusivity of the PH domain when multiple PIPs are bound is lower than that when a single PIPs is bound (see e.g.^8^). Additionally, the time scale of subdiffusion is stretched upon binding of additional PIPs [see Fig. S7]. Thus the complex interaction between the PH domain and lipids generates a heterogeneous diffusion in time. Such a heterogeneous subdiffusion is also observed for e.g. lipid diffusion in phase-separated lipid membranes^46^ and for tracer subdiffusion in crowded ensembles of sticky obstacles^47^.

Many biological processes on cell membranes require the encounter at the membrane surface of multiple species of proteins and/or lipids. In turn, the diffusion of proteins on the surface and of lipids within the membrane is likely to determine their frequency of interaction. In searching for their target molecules on the membrane, peripheral proteins follow an intermittent search model, with slower diffusion on the membrane and faster diffusion in the aqueous environment. Our results imply that the two-state dynamic interaction of surface bound PH domains processes reflected in anomalous diffusion and that *1/f* noise increases the residence time of a PH domain on the cell membrane surfaces. One might think that subdiffusion would be associated with a slow sampling of possible protein targets by the PH domain. However, using generalized stochastic simulations, it has been shown that the probability of finding a nearby target is actually increased by subdiffusion^35^. Anomalous diffusion will increase the search time and thus the efficiency of the search. Interestingly, Guigas, G. & Weiss^35^ suggest that a value of α = 2/3 provides an optimal target between the probability of finding a target and a long search time. In our simulations we observed a value for α of ~0.65 in the coarse-grained simulations (for the subdiffusive behavior). Thus, the subdiffusion and associated *1/f* noise observed in our simulations of a PH domain on a membrane surface may provide a mechanism whereby encounter with a target protein complex on the membrane is optimized.

Despite a degree of sequence variation in the PH domain family of proteins, PH domains have a conserved positively charged loop between the β1 and β2 strands which binds to PIP molecules in the membrane. We note that there are over 10 structures of PH/PIP complexes in the PDB and in all of them the PIP headgroup is bound close to the β1-β2 loop. This suggests that our conclussions may apply to PH domains in general. A number of other classes of peripheral protein (e.g. C2 domains) also associate with anionic lipids, and so we suggest that our proposed mechanism of optimization of encounter via subdiffusion on the bilayer may be of relevant to membrane-targeting peripheral proteins in general. Testing this experimentally should be achievable via e.g. quantitative single molecule microscopy^48^. It will be important also to test this computational by extending the current analysis to simulations of a wider range of peripheral membrane proteins, and in particular to those domains such as N-BAR which perturb local bilayer properties. Furthermore, it will be of interest to explore the effects of membrane crowding on (sub)diffusion of bound peripheral membrane proteins, as crowding has been shown to influence the diffusive behavior of integral membrane proteins^49^.

## METHODS

### Coarse-grained molecular dynamics simulations (CG-MD)

CG-MD simulations were performed using GROMACS-4.5.5 (see www.gromacs.org)^50^ with the Martini 2.1 force field^42^,^51^. The bilayer used in the simulations consisted of POPC/POPS/PIP_2_/PIP_3_ (259 POPC (73%), 71 POPS (20%), 18 PIP_2_ (5%), and 8 PIP_3_ (2%)), or POPC (356 POPC (100%)), or POPC/POPS (285 POPC (80%) and 71 POPS (20%) lipid molecules, and was modelled as described previously^52^. Note that each leaflet had 4 PIP_3_ molecules and 9 PIP_2_ molecules. Systems were solvated with ca. 14000 CG water molecules and NaCl ions at 150 mM concentration were added to neutralize the system. The system used for the MD simulations is shown in Fig. S1 (in which the system size *L*_*x*_ × *L*_*y*_ × *L*_*z*_ for the CG simulations is 10.9 × 10.9 × 19.4 nm^3^). Flexible loop regions missing from the structures were modeled using MODELLER^53^. All systems were energy minimized for 200 steps, and equilibrated for 1 ns with the protein backbone particles restrained. For each repeat simulation within an ensemble the protein was rotated around the *x*, *y* and *z* axes to form a new initial configuration. For each system an ensemble of 25 simulations of 1 μs each were run, with a time step of 20 fs. For the protein diffusive dynamics, 25 simulations of 10 μs with DAPP1 PH domain (PDB code: 1FAO^38^) and of 5 μs with protein kinase B PH domain (PDB code: 1UNQ^43^) were performed. An elastic network model (ENM) was applied to all backbone particles with a cut-off distance of 0.7 nm to model secondary and tertiary structure^54^. It should be noted that the ENM was *not* applied to the PIP molecules, and also that the PIP-binding loops were flexible in the CG simulations. We checked the overall effect of the ENM by comparing root mean square fluctuations (RMSFs) as a function of residue in the CG-MD and AT-MD simulations, revealing similar RMSF profiles [see Fig. S16]. Lennard-Jones interactions were shifted to zero between 0.9 nm and 1.2 nm and Coulombic interactions between 0 and 1.2 nm, respectively. The pressure of 1 bar and a temperature of 323 K were controlled using the Berendsen’s algorithm^55^ with a coupling time of 1 ps. We note that whilst the CG model may be expected to smooth the energy landscape for protein/lipid dynamics, it has been shown that CG simulations using the MARTINI force field agree reasonably well with experimental measurement of the diffusion of integral membrane proteins^49^.

### Atomistic molecular dynamics simulations (AT-MD)

Conversion of CG to atomistic systems was made using a fragment-based approach^56^. The system used for the AT-MD simulation is shown in Fig. S1 (in which the system size *L*_*x*_ × *L*_*y*_ × *L*_*z*_ is 10.9 × 10.9 × 12.2 nm^3^). We performed 1 μs MD simulation of the system with the DAPP1 PH domain. The GROMOS96 43a1 force field^57^ was used with SPC water molecules using GROMACS-4.5.5 software^50^. The temperature of 323 K was controlled using the velocity rescaling method^58^ with a coupling time of 0.1 ps. The pressure of 1 bar was controlled with anisotropic pressure coupling, where the box length in *x*, *y* was fixed as the same value of CG simulations, using the Parrinello-Rahman barostat^59^ with a coupling time of 1 ps. Bond lengths were constrained to equilibrium lengths using the LINCS method^60^. The time step was set at 2 fs. The particle mesh Ewald method was used, with a specified direct space cutoff distance of 1.0 nm. We also performed two 250 ns MD simulations of DAPP1 PH domain in water (i.e. without any bilayer present. [see Fig. S1].

The DAPP1 PH structure was determined by X-ray diffraction and crystallized under aqueous conditions in a complex with Ins-P4, i.e. the headgroup of PIP_3_. As this is a peripheral membrane protein, the majority of the protein when bound remains exposed to water, and so major structural changes are not anticipated, and indeed have not been seen in extended atomistic simulations of this or other PH domains bound to the surface of a membrane. However, to check this we have compared the root mean square deviations (RMSDs) of the DAPP1 PH domain in AT-MD simulations in both a lipid membrane bound configuration, and in aqueous solution. For the core fold of the protein (i.e. excluding loops) the change in the Cα RMSD from the start to the end of the simulation is ~0.19 nm (averaged over 2 simulations of 1 μs duration each) for the membrane bound form of the PH domain, and ~0.15 nm (averaged over 2 simulations of 0.25 μs duration each) for the PH domain in aqueous solution [see Fig. S16]. We have thereby confirmed that the conformation of the DAPP1 PH domain does not change significantly other than (as anticipated) in the more flexible loops which interact with the membrane. We have also confirmed this by analysis of the root mean square fluctuations (RMSFs) as a function of residue number. For the core residues the Cα RMFS in all four simulations are general between 0.1 and 0.15 nm, indicating that the fold of the protein remains stable both in aqueous solution and when bound to the membrane [see Fig. S16].

### Stochastic model of residence time of a protein on a membrane surface

In this model (see above and Fig. 4a) *H* and *L* are the residence times for ‘high PIP’ and ‘low PIP’ binding states, respectively. Then, the residence time of the PH domain on the membrane surface is given by *T*_e_ = *H*_1_ + *L*_1_ + *H*_2_ +···+ *H*_*n*_ + *U*_*n*_, where *H*_*i*_ and *L*_*i*_ are the *i*-th residence time for ‘high PIP’ and ‘low PIP’ binding states, respectively, and *U*_*n*_ is a dissociation time in the weak binding state. We assume that dissociation occurs at the *n*-th ‘low PIP’ binding state, i.e., *L*_*n*_ > *U*_*n*_ and *L*_*i*_ < *U*_*i*_ for *i* < *n*. In numerical simulations, we assume the PDF of *H*, *P*_*H*_(*τ*), is the same as that of *L*, *P*_*L*_(*τ*). Moreover, the PDF of *U* is given by *P*_*U*_ (*τ*) = *ce*^−*cτ*^, where 1/*c* is the mean dissociation time in the weak binding state.

## Additional Information

**How to cite this article**: Yamamoto, E. *et al.* Anomalous Dynamics of a Lipid Recognition Protein on a Membrane Surface. *Sci. Rep.* **5**, 18245; doi: 10.1038/srep18245 (2015).

## Supplementary Material

Supplementary Information

Supplementary Dataset 1

Supplementary Dataset 2

## Figures and Tables

**Table 1:** Summary of the simulations.

**Protein**	**PDB**	**Membrane**	**Simulation**	**Time**
DAPP1	1FAO^38^	POPC/POPS/PIP_2_/PIP_3_	CG	25 × 10 μs
DAPP1	1FAO	POPC/POPS	CG	25 × 1 μs
DAPP1	1FAO	POPC	CG	25 × 1 μs
DAPP1	1FAO	POPC/POPS/PIP_2_/PIP_3_	AT	2 × 1 μs
DAPP1	1FAO	not present	AT	2 × 0.25 μs
Protein kinase B	1UNQ^43^	POPC/POPS/PIP_2_/PIP_3_	CG	25 × 5 μs

^*^CG = coarse grained; AT = atomistic.
