# Supplementary material for: Anomalous Dynamics of a Lipid Recognition Protein on a Membrane Surface
Source: Sci Rep. 2015 Dec 14;5:18245. doi: 10.1038/srep18245 (PMC4677404; doi:10.1038/srep18245)
Supplement: Supplementary Information [file srep18245-s1.pdf]

Supporting Information for "Anomalous dynamics of a lipid recognition protein on a membrane surface"

Eiji Yamamoto,[1] Antreas C. Kalli,[2] Takuma Akimoto,[1] Kenji Yasuoka,[1] and Mark S. P. Sansom[2]

[1] *Department of Mechanical Engineering, Keio University, Yokohama, Kanagawa 223-8522, Japan*

[2] *Department of Biochemistry, University of Oxford, South Parks Road, Oxford OX1 3QU, United Kingdom*

Rotational TAMSD

To calculate the rotational diffusion, we define a vector $\overrightarrow{\varphi}(t) \equiv \int_{t_0}^{t_0+t} \delta\theta(t')\overrightarrow{p}(t')dt'$, where $\delta\theta(t) \equiv \cos^{-1}(\overrightarrow{\mu}(t) \cdot \overrightarrow{\mu}(t+\delta t))$, direction $\overrightarrow{p}(t) \equiv \overrightarrow{\mu}(t) \times \overrightarrow{\mu}(t+\delta t)$, and $\overrightarrow{\mu}(t)$ is the defined vector at time t [1, 2]. This gives us the trajectory representing the rotational motion. Then, the rotational time-averaged mean square displacements (rTAMSD) is defined by $\overline{\delta_{\varphi}^2(\Delta;T)} = \frac{1}{T-\Delta}\int_0^{T-\Delta} |\vec{\varphi}(t'+\Delta) - \vec{\varphi}(t')|^2 dt'$. We defined vectors $\overrightarrow{\mu}_i$ from the center of mass of the protein to an i th amino acid. We defined 9 different vectors [see Fig. 2C].

---

[1] Mazza, M. G., Giovambattista, N., Starr, F. W. & Stanley, H. E. Relation between rotational and translational dynamic heterogeneities in water. *Phys. Rev. Lett.* **96**, 057803 (2006).

[2] Yamamoto, E., Akimoto, T., Yasui, M. & Yasuoka, K. Origin of subdiffusion of water molecules on cell membrane surfaces. *Sci. Rep.* **4**, 4720 (2014).

[3] Niemann, M., Kantz, H. & Barkai, E. Fluctuations of $1/f$ noise and the low-frequency cutoff paradox. *Phys. Rev. Lett.* **110**, 140603 (2013).

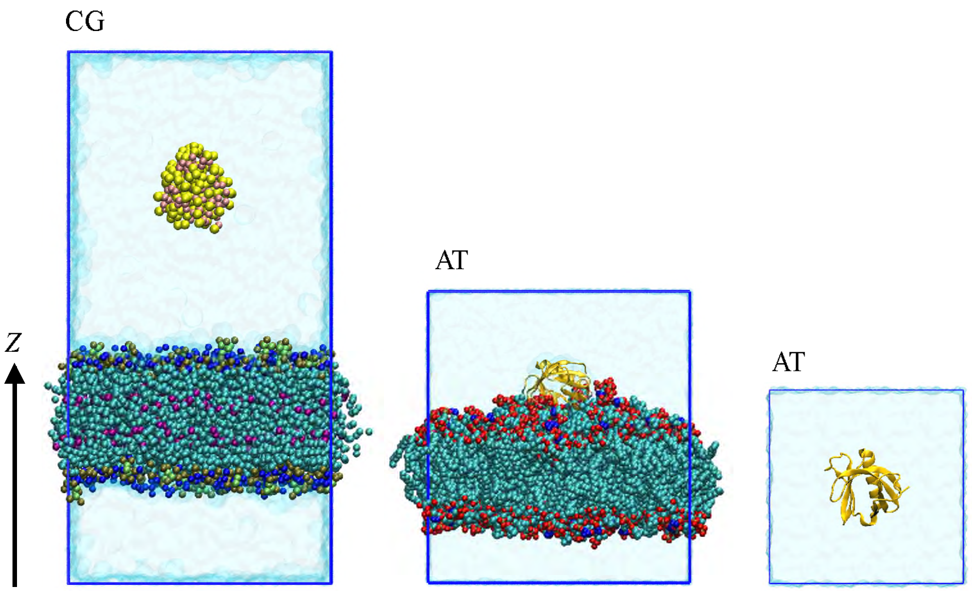

Fig. S 1: The initial system used for MD simulations. The PH domain and lipids are shown in yellow and cyan, respectively. Water molecules correspond to the upper and lower transparent coatings. The blue line of the box defines the unit cell. The system sizes $L_x \times L_y \times L_z$ are CG: 10.9×10.9×19.4 nm^3, AT with lipid membrane: 10.9×10.9×12.2 nm^3, and AT in bulk: 7.0×7.0×7.0 nm^3.

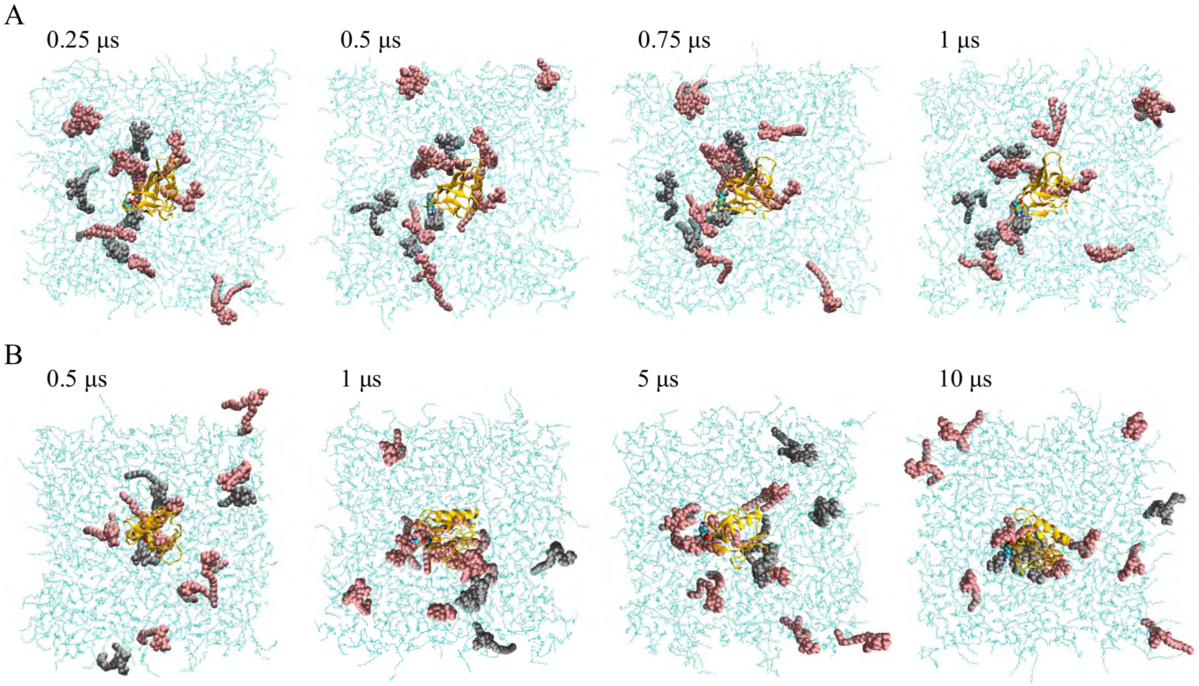

Fig. S 2: Snapshots of the DAPP1 PH domain on the lipid membrane, (A) AT-MD and (B) CG-MD. The leaflet where the PH domain binds is shown. PIP_2 and PIP_3 lipids are shown as pink and grey, respectively. POPC and POPS lipids are colored in cyan. Residue K179 is shown in VDW format (cyan).

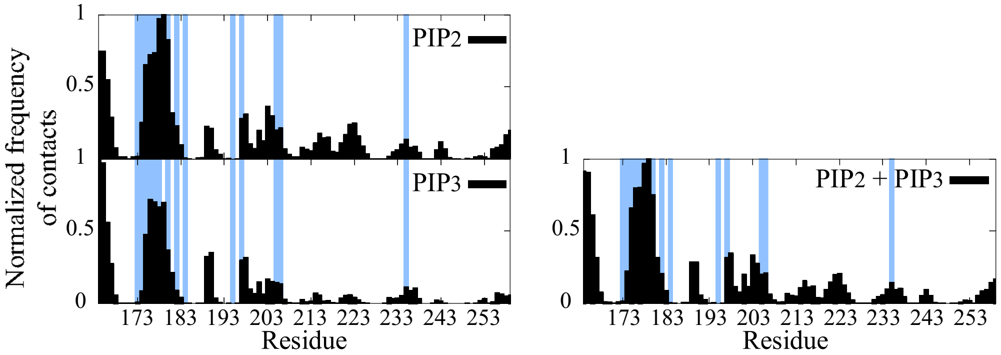

Fig. S 3: Normalized averaged number of contacts between the DAPP1 PH domain and PIP_2 or PIP_3 in the CG-MD simulations on a PC/PS/PIP_2/PIP_3 membrane surface. The light blue colors represent the experimental contacts using the crystal structure (cutoff distance 0.4 nm).

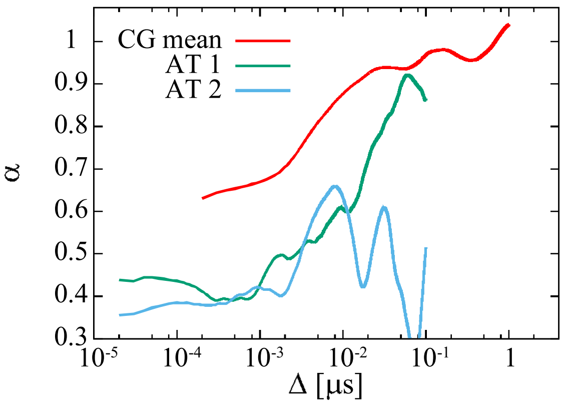

Fig. S 4: Exponent α of the translational TAMSDs versus time. The α was calculated by fitting a straight line in moving window with 0.4 time units in logarithmic scale with a base of 10.

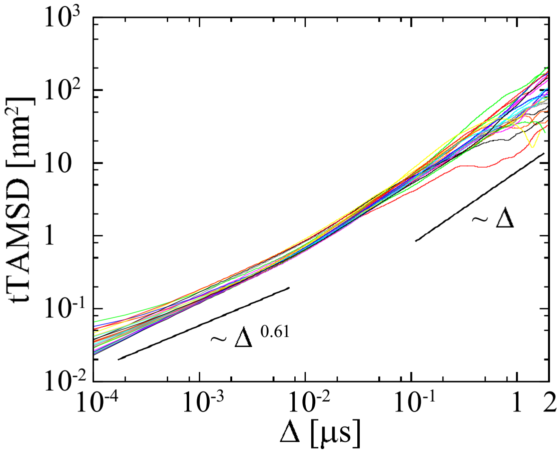

Fig. S 5: Translational TAMSDs of all 25 trajectories of protein kinase B PH domain on the PC/PS/PIP_2/PIP_3 membrane surface. The different colored lines represent the individual 25 trajectories. The black solid lines are shown for reference.

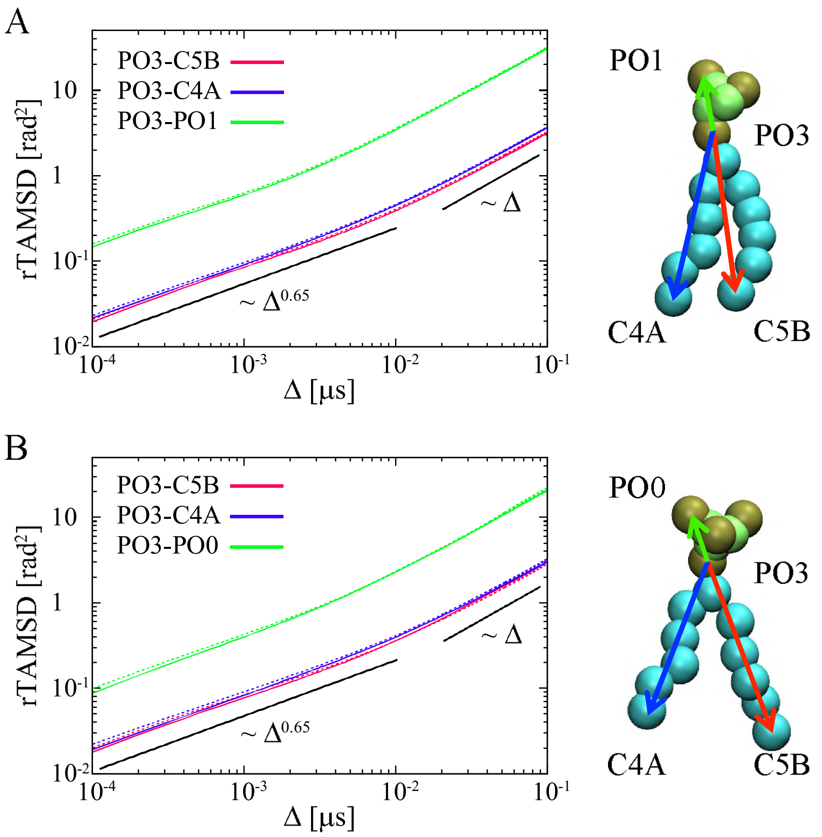

Fig. S 6: Rotational mean TAMSDs of (A) PIP_2 and (B) PIP_3 molecules. The colored lines represent the different vectors. The solid and dashed lines represent two states: bound and unbound states to the DAPP1 PH domain, respectively. The black solid lines are shown for reference. There are no significant differences of rotational motions between bound and unbound states.

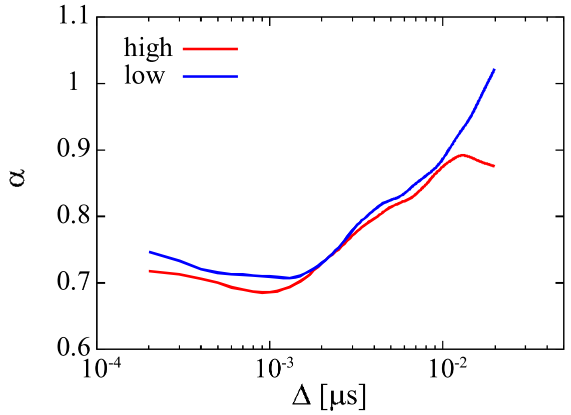

Fig. S 7: Exponent α of translational mean TAMSDs of 'high' and 'low' states plotted in function of the simulation time. The α was calculated by fitting a straight line in moving window with 0.4 time units in logarithmic scale with a base of 10. For the calculation of mean TAMSDs, we used the trajectories which remain in the same state for more than 0.1 μs. This yielded ensembles of 153 and 140 for 'high' and 'low' states, respectively.

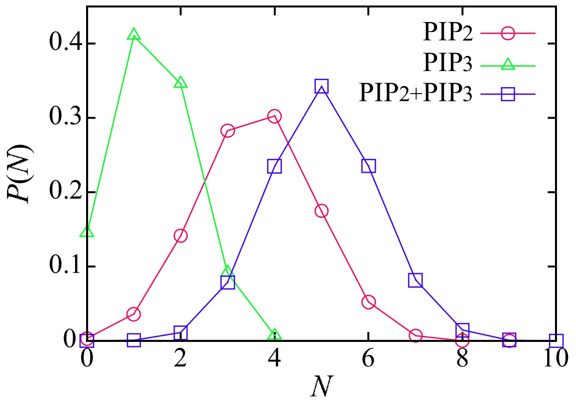

Fig. S 8: Probability of the number of PIP_2, PIP_3, and PIP_2+PIP_3 molecules around the DAPP1 PH domain (using a cutoff distance of 0.7 nm).

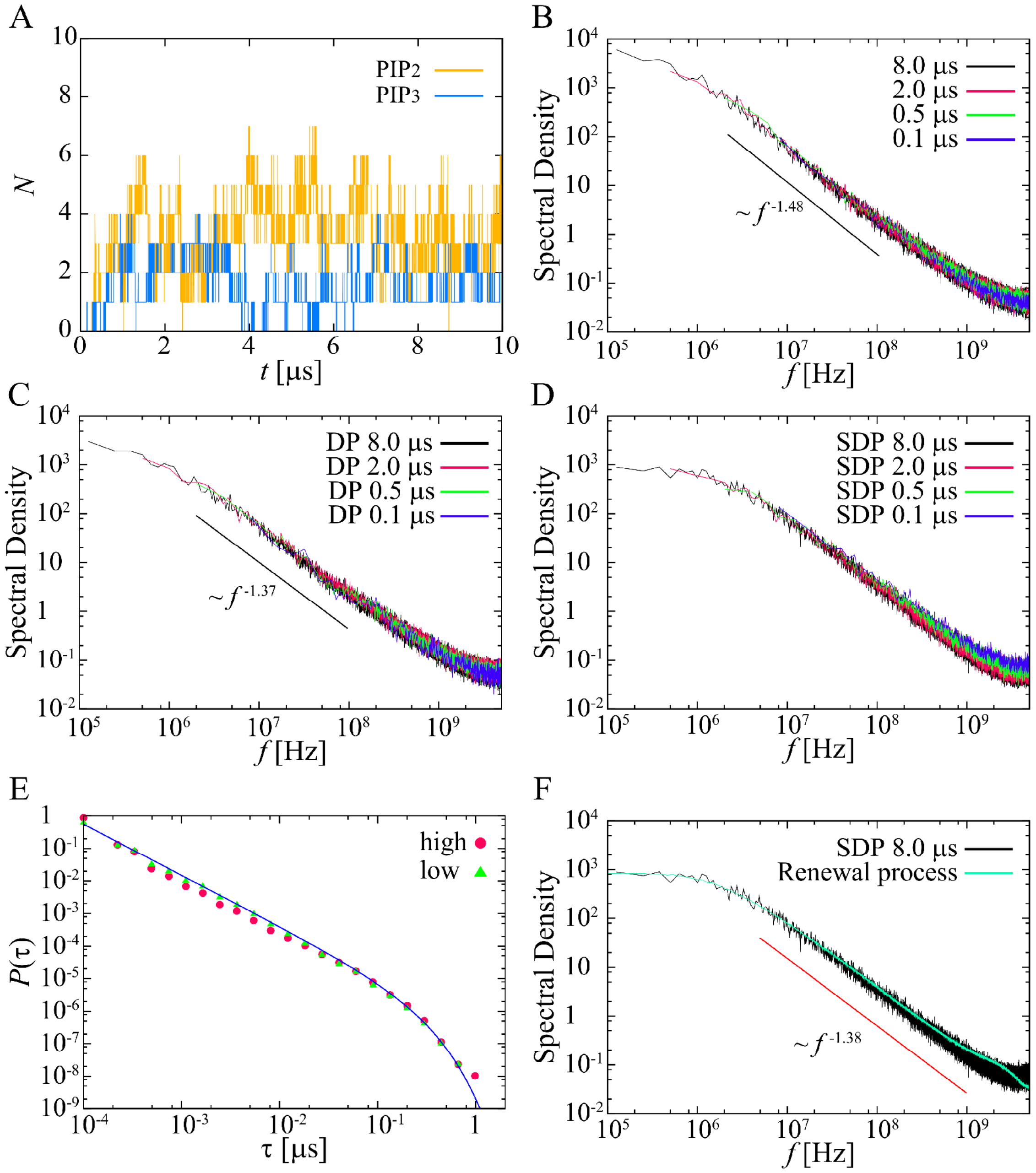

Fig. S 9: $1/f$ noise in the interaction between PIP_2 and the DAPP1 PH domain. (A) Time series of the number of PIP_2 and PIP_3 molecules around the PH domain (using a cutoff distance of 0.7 nm). (B) Ensemble-averaged PSD of 25 time series of number of PIP_2 around the PH domain. Different colored lines distinguish different measurement times. The power spectra coincide without fitting. The power-law exponent is $\beta = 1.48 \pm 0.06$. The solid lines are shown as reference. (C) Ensemble-averaged PSD of the time series of dichotomous process (DP) of the number of PIP_2 around the protein. The power-law exponent is $\beta = 1.37 \pm 0.06$. (D) Ensemble-averaged PSD of shuffled dichotomous processes (SDP) with different measurement times. (E) PDFs of residence times of "high" and "low" states. Solid lines are fitted curves for power-law distributions with exponential cutoffs: $P(\tau) = A\tau^{-1-\gamma}\exp(-\tau/\tau_c)$ ($\gamma = 0.57$, $\tau_c = 200$ ns). (F) Ensemble-averaged PSD of SDP (black line). Numerical simulation of alternating renewal process; residence times are given by power-law distribution with exponential cutoff, where γ and τ_c are the same as observed values (green line). The power-law exponent is $\beta = 1.38 \pm 0.07$.

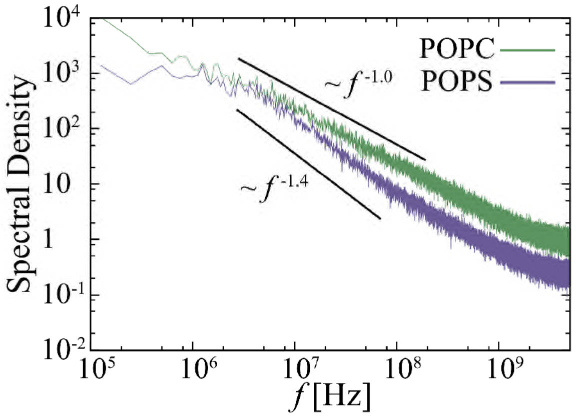

Fig. S 10: Ensemble-averaged PSD of 25 time series of number of POPC and POPS around the DAPP1 PH domain (using a cutoff distance of 0.7 nm). The black solid lines are shown as reference.

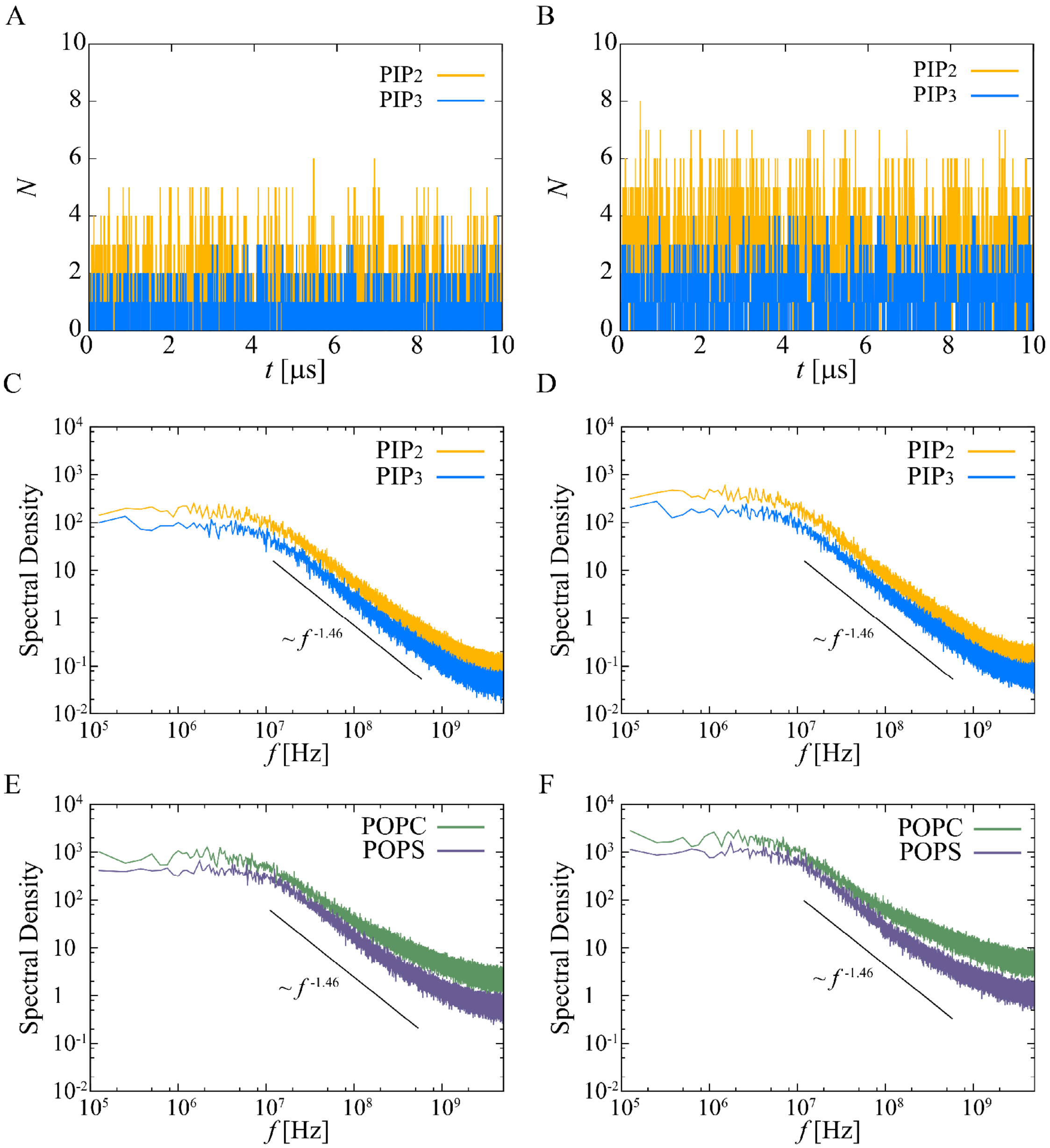

Fig. S 11: Time series of the number of PIP_2 and PIP_3 in a circular area where the radius of the circle is (A) $r = 2.0$ nm and (B) $r = 3.0$ nm. The time series are for PIP molecules in the *opposite* leaflet from that to which the protein was bound. Average number and standard deviation of N are PIP_2: 1.2 ± 1.0 and PIP_3: 0.6 ± 0.7 for $r = 2.0$ nm and PIP_2: 2.5 ± 1.3 and PIP_3: 1.1 ± 0.9 for $r = 3.0$ nm. Ensemble-averaged PSD of 25 time series of number of PIP_2 and PIP_3 in circular areas, (C) $r = 2.0$ nm and (D) $r = 3.0$ nm. Ensemble-averaged PSD of 25 time series of number of PIP_2 and PIP_3 in circular areas, (E) $r = 2.0$ nm and (F) $r = 3.0$ nm. The range of the $1/f$ noise is much shorter than that for PH domain.

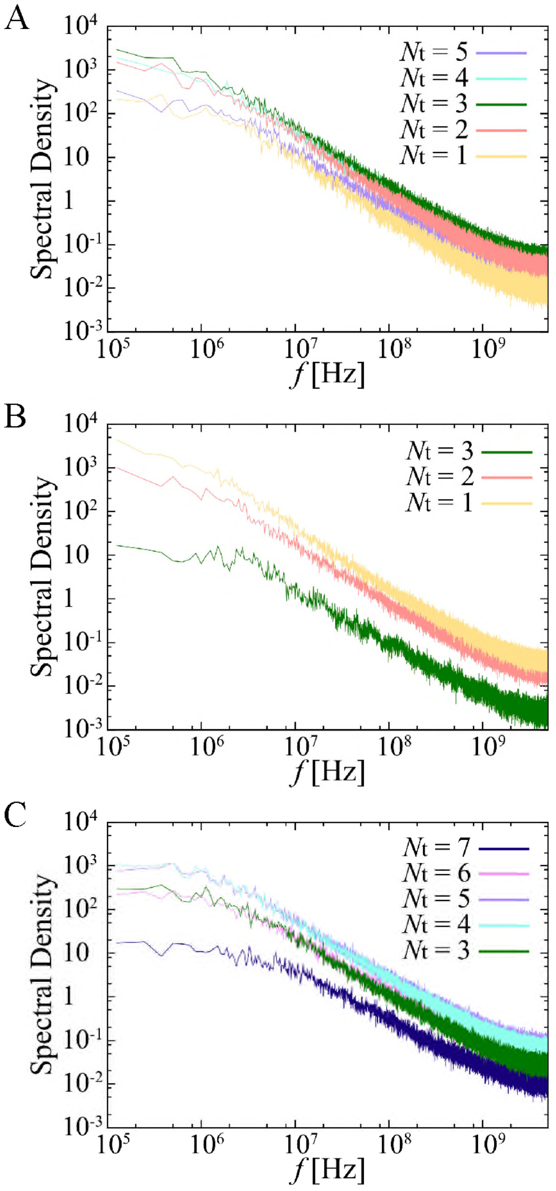

Fig. S 12: Ensemble-averaged PSDs of the time series of dichotomous process (DP) with “high” state ($N' = 1$ if $N_t < N$) and “low” state ($N' = -1$ if $N \leq N_t$) for (A) PIP_2, (B) PIP_3, and (C) PIP_2 + PIP_3. The PSD of PIP_2 with $N_t = 3$ is the same as that using the average number of each trajectory. In the case of PIP_3, $N_t = 1$ is the same as that of the average one. The magnitude of the PSD is given by $\langle S_t(\omega)\rangle \propto N'^2 \frac{\langle n(t)\rangle}{t}$ [3], where $n(t)$ is the number of renewals in $(0, t)$. When the N_t is the same as the mean value, the magnitude of PSD show the highest value because $n(t)$ has the highest value. If the N_t is different from the mean value, the $n(t)$ becomes small, and the residence time of one state becomes large and that of the other state becomes small. Thus, the magnitude of the PSD becomes small. And transition frequency from $1/f$ noise to plateau becomes high because it depend on the smallest cutoff in residence times in the dichotomous process.

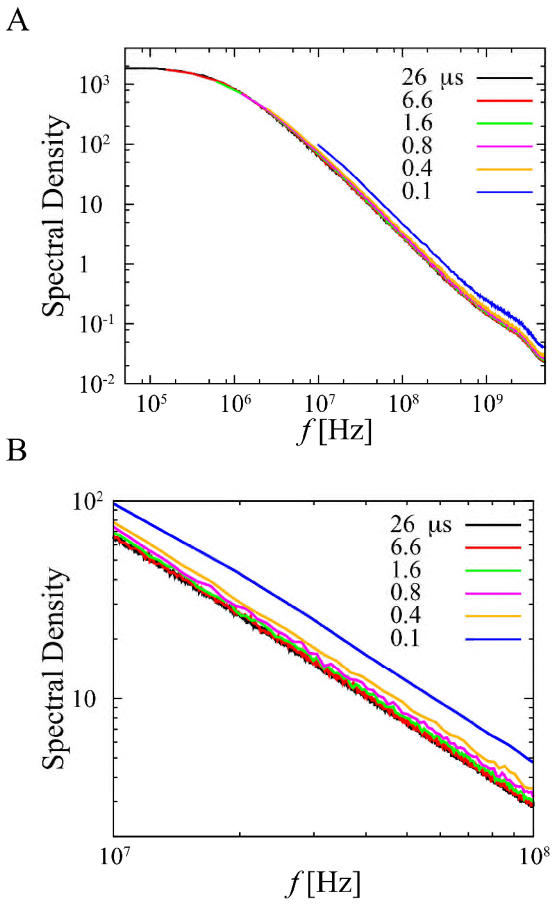

Fig. S 13: (A)(B) Ensemble-averaged PSD of the alternating renewal process. Different colored lines distinguish different measurement times.

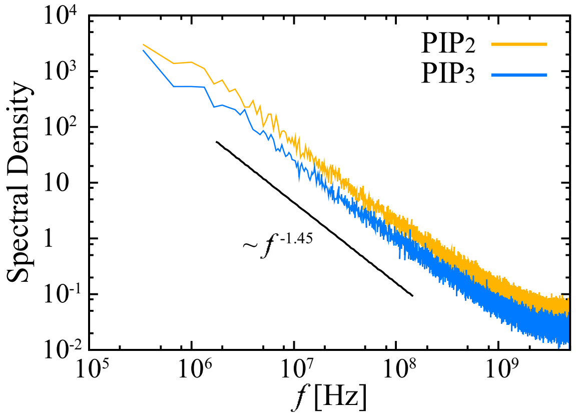

Fig. S 14: Ensemble-averaged PSD of 25 time series of number of PIP_2 and PIP_3 around the protein kinase B PH domain.

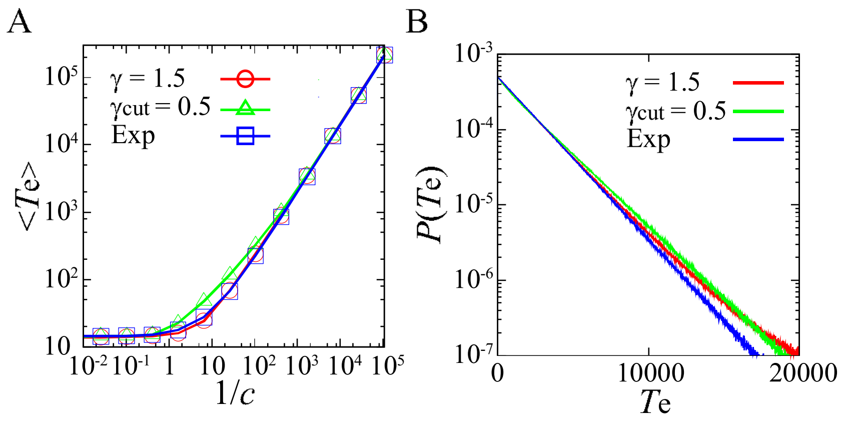

Fig. S 15: Stochastic modeling of the residence time of a PH domain on a membrane surface. (A) Mean residence time $\langle T_e \rangle$ with PDF of power-law (red), power-law withe cutoff (green), and exponential distribution (blue). (B) PDFs of residence times with $1/c = 1000$.

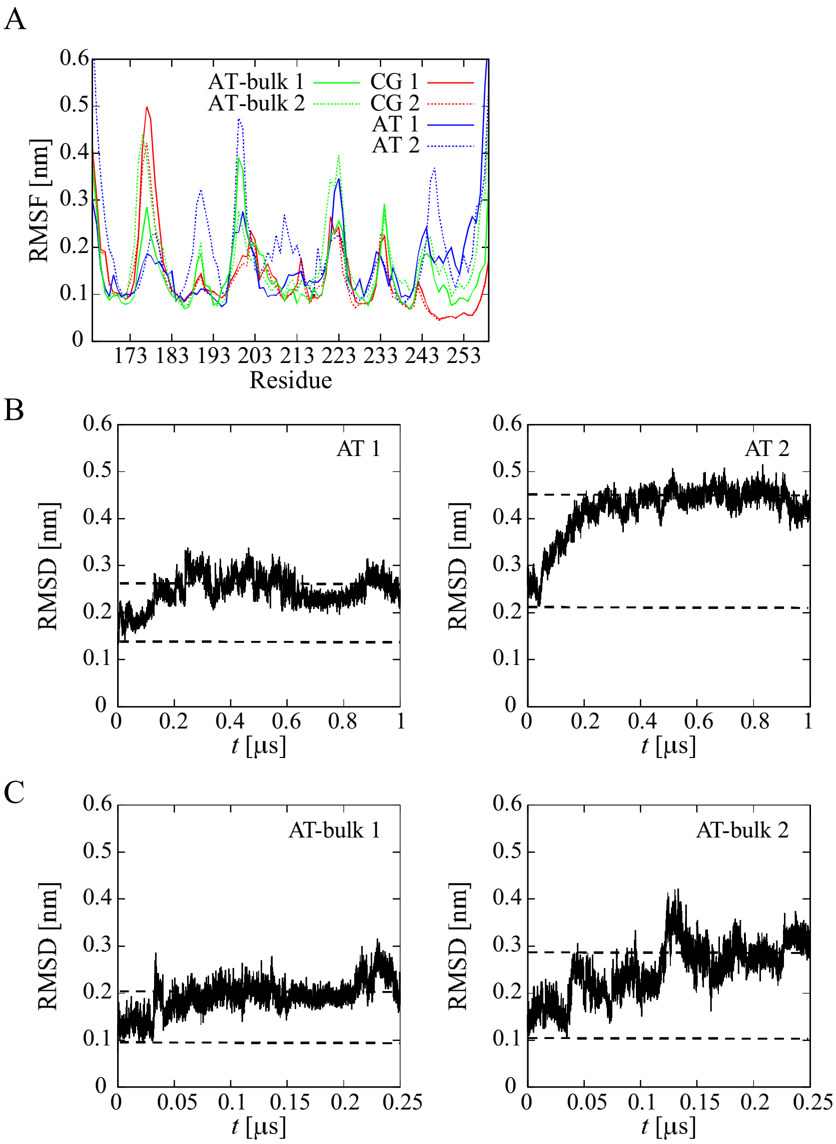

Fig. S 16: (A) Root mean square fluctuation (RMSF) for the C_α atoms of the DAPP1 PH domain. Different line types represent different simulation systems. (B) Root mean square deviation (RMSD) for the C_α atoms of the core residues (i.e. omitting the surface loops) of the DAPP1 PH domain on the membrane surface and (C) in bulk water (i.e. in the absence of a lipid bilayer). The broken horizontal lines indicate the initial RMSD (from the crystal structure) and the average over the latter half of the simulation. The difference between these two values thus provides a measure of the degree of conformational drift of the protein structure over the course of the simulation.
