# Supplementary material for: Anomalous Dynamics of a Lipid Recognition Protein on a Membrane Surface
Source: Sci Rep. 2015 Dec 14;5:18245. doi: 10.1038/srep18245 (PMC4677404; doi:10.1038/srep18245)
Supplement: Supplementary Dataset 1 [file srep18245-s2.doc]

; VARIOUS PREPROCESSING OPTIONS =

title =

cpp = /lib/cpp

include =

define =

; RUN CONTROL PARAMETERS =

integrator = md

; start time and timestep in ps =

tinit = 0.0

dt = 0.02

nsteps = 50000000

; number of steps for center of mass motion removal =

nstcomm = 1

; OUTPUT CONTROL OPTIONS =

; Output frequency for coords (x), velocities (v) and forces (f) =

nstxout = 5000

nstvout = 200000

nstfout = 0

; Output frequency for energies to log file and energy file =

nstlog = 20000

nstenergy = 20000

; Output frequency and precision for xtc file =

nstxtcout = 5000

xtc_precision = 5000

; This selects the subset of atoms for the xtc file. You can =

; select multiple groups. By default all atoms will be written. =

xtc-grps =

; Selection of energy groups =

energygrps = PROTEIN POPC POPS PIP2 PI3 W NA_CL

; NEIGHBORSEARCHING PARAMETERS =

; nblist update frequency =

nstlist = 10

; ns algorithm (simple or grid) =

ns_type = grid

; Periodic boundary conditions: xyz or none =

pbc = xyz

; nblist cut-off =

rlist = 1.3

domain-decomposition = no

; OPTIONS FOR ELECTROSTATICS AND VDW =

; Method for doing electrostatics =

coulombtype = Shift

rcoulomb_switch = 0.0

rcoulomb = 1.2

; Dielectric constant (DC) for cut-off or DC of reaction field =

epsilon_r = 20

; Method for doing Van der Waals =

vdw_type = Shift

; cut-off lengths =

rvdw_switch = 0.9

rvdw = 1.2

; Apply long range dispersion corrections for Energy and Pressure =

DispCorr = No

; Spacing for the PME/PPPM FFT grid =

fourierspacing = 0.12

; FFT grid size, when a value is 0 fourierspacing will be used =

fourier_nx = 10

fourier_ny = 10

fourier_nz = 10

; EWALD/PME/PPPM parameters =

pme_order = 4

ewald_rtol = 1e-05

epsilon_surface = 0

optimize_fft = no

; OPTIONS FOR WEAK COUPLING ALGORITHMS =

; Temperature coupling =

tcoupl = Berendsen

; Groups to couple separately =

tc-grps = PROTEIN POPC POPS PIP2 PI3 W NA_CL

; Time constant (ps) and reference temperature (K) =

tau_t = 1.0 1.0 1.0 1.0 1.0 1.0 1.0

ref_t = 323 323 323 323 323 323 323

; Pressure coupling =

Pcoupl = Berendsen

Pcoupltype = semiisotropic

; Time constant (ps), compressibility (1/bar) and reference P (bar) =

tau_p = 1.0 1.0

compressibility = 5e-6 5e-6

ref_p = 1.0 1.0

; SIMULATED ANNEALING

; Type of annealing for each temperature group (no/single/periodic)

annealing =

; Number of time points to use for specifying annealing in each group

annealing_npoints =

; List of times at the annealing points for each group

annealing_time =

; Temp. at each annealing point, for each group.

annealing_temp =

; GENERATE VELOCITIES FOR STARTUP RUN =

gen_vel = yes

gen_temp = 310

gen_seed = 678407840686218237185773

; OPTIONS FOR BONDS =

constraints = none

; Type of constraint algorithm =

constraint_algorithm = Lincs

; Do not constrain the start configuration =

unconstrained_start = no

; Relative tolerance of shake =

shake_tol = 0.0001

; Highest order in the expansion of the constraint coupling matrix =

lincs_order = 4

; Lincs will write a warning to the stderr if in one step a bond =

; rotates over more degrees than =

lincs_warnangle = 30

; Convert harmonic bonds to morse potentials =

morse = no

; NMR refinement stuff =

; Distance restraints type: No, Simple or Ensemble =

disre = simple

; Force weighting of pairs in one distance restraint: Equal or Conservative =

disre_weighting = Equal

; Use sqrt of the time averaged times the instantaneous violation =

disre_mixed = no

disre_fc = 1000

disre_tau = 50

; Output frequency for pair distances to energy file =

nstdisreout = 100

; Free energy control stuff =

free_energy = no

init_lambda = 0

delta_lambda = 0

sc-alpha = 0

sc-sigma = 0.3

; Non-equilibrium MD stuff =

acc-grps =

accelerate =

freezegrps =

freezedim =

cos-acceleration = 0

energygrp_excl =

; Electric fields =

; Format is number of terms (int) and for all terms an amplitude (real) =

; and a phase angle (real) =

E-x =

E-xt =

E-y =

E-yt =

E-z =

E-zt =

; User defined thingies =

user1-grps =

user2-grps =

userint1 = 0

userint2 = 0

userint3 = 0

userint4 = 0

userreal1 = 0

userreal2 = 0

userreal3 = 0

userreal4 = 0
