# Supplementary material for: Anomalous Dynamics of a Lipid Recognition Protein on a Membrane Surface
Source: Sci Rep. 2015 Dec 14;5:18245. doi: 10.1038/srep18245 (PMC4677404; doi:10.1038/srep18245)
Supplement: Supplementary Dataset 2 [file srep18245-s3.doc]

TITLE Generated by trjconv : BILAYER + PROTEIN t= 0.00000

REMARK THIS IS A SIMULATION BOX

CRYST1 109.387 109.387 194.382 90.00 90.00 90.00 P 1 1

MODEL 1

ATOM 1 BCQd LEU 1 47.890 67.030 104.590 1.00 0.00

ATOM 2 1SCA LEU 1 49.140 65.140 102.160 1.00 0.00

ATOM 3 5BCP GLY 2 48.740 66.210 106.690 1.00 0.00

ATOM 4 5BCP THR 3 52.090 63.490 107.690 1.00 0.00

ATOM 5 1SCP THR 3 53.990 65.110 108.430 1.00 0.00

ATOM 6 BENd LYS 4 51.590 60.480 108.390 1.00 0.00

ATOM 7 3SEC LYS 4 48.730 60.440 110.090 1.00 0.00

ATOM 8 SEQd LYS 4 47.460 61.060 112.630 1.00 0.00

ATOM 9 BENd GLU 5 53.720 58.190 107.870 1.00 0.00

ATOM 10 SEQa GLU 5 55.740 59.290 111.220 1.00 0.00

ATOM 11 BENd GLY 6 54.490 54.740 107.120 1.00 0.00

ATOM 12 BENd TYR 7 54.460 52.310 104.950 1.00 0.00

ATOM 13 4SES TYR 7 56.140 50.290 106.090 1.00 0.00

ATOM 14 4SES TYR 7 58.700 49.770 106.790 1.00 0.00

ATOM 15 1SES TYR 7 56.860 47.790 106.820 1.00 0.00

ATOM 16 BENd LEU 8 53.530 51.250 102.020 1.00 0.00

ATOM 17 1SEA LEU 8 55.170 52.590 100.020 1.00 0.00

ATOM 18 BENd THR 9 54.450 47.540 100.530 1.00 0.00

ATOM 19 1SEP THR 9 53.670 46.550 102.810 1.00 0.00

ATOM 20 BENd LYS 10 54.170 46.910 96.890 1.00 0.00

ATOM 21 3SEC LYS 10 55.570 49.610 95.980 1.00 0.00

ATOM 22 SEQd LYS 10 57.630 50.760 95.640 1.00 0.00

ATOM 23 BENd GLN 11 53.560 45.140 94.470 1.00 0.00

ATOM 24 4SEP GLN 11 50.370 43.640 95.920 1.00 0.00

ATOM 25 5BCP GLY 12 52.940 45.370 91.980 1.00 0.00

ATOM 26 5BSP GLY 13 51.150 47.150 88.460 1.00 0.00

ATOM 27 5BSP LEU 14 52.260 47.820 87.060 1.00 0.00

ATOM 28 1SSA LEU 14 51.260 49.470 84.160 1.00 0.00

ATOM 29 5BSP VAL 15 55.760 45.860 87.720 1.00 0.00

ATOM 30 2SSA VAL 15 56.960 47.440 85.960 1.00 0.00

ATOM 31 5BCP LYS 16 55.870 43.710 90.110 1.00 0.00

ATOM 32 3SCC LYS 16 55.360 40.830 88.580 1.00 0.00

ATOM 33 SCQd LYS 16 55.110 38.790 86.620 1.00 0.00

ATOM 34 5BCP THR 17 57.480 42.950 92.910 1.00 0.00

ATOM 35 1SCP THR 17 58.880 40.790 92.510 1.00 0.00

ATOM 36 BENd TRP 18 57.920 44.670 96.330 1.00 0.00

ATOM 37 4SES TRP 18 56.290 42.100 97.190 1.00 0.00

ATOM 38 1SES TRP 18 53.920 41.010 97.910 1.00 0.00

ATOM 39 4SES TRP 18 55.200 42.700 99.590 1.00 0.00

ATOM 40 4SES TRP 18 52.870 41.570 100.330 1.00 0.00

ATOM 41 BENd LYS 19 59.200 46.720 97.560 1.00 0.00

ATOM 42 3SEC LYS 19 61.890 46.750 99.000 1.00 0.00

ATOM 43 SEQd LYS 19 63.480 48.430 100.070 1.00 0.00

ATOM 44 BENd THR 20 58.290 48.070 101.610 1.00 0.00

ATOM 45 1SEP THR 20 59.580 46.400 103.120 1.00 0.00

ATOM 46 BENd ARG 21 59.440 51.400 102.070 1.00 0.00

ATOM 47 0SEN ARG 21 60.630 52.050 99.090 1.00 0.00

ATOM 48 SEQd ARG 21 62.290 53.100 97.090 1.00 0.00

ATOM 49 BENd TRP 22 58.870 53.980 103.980 1.00 0.00

ATOM 50 4SES TRP 22 59.700 54.230 107.000 1.00 0.00

ATOM 51 1SES TRP 22 59.200 53.560 109.570 1.00 0.00

ATOM 52 4SES TRP 22 58.900 56.120 108.760 1.00 0.00

ATOM 53 4SES TRP 22 58.760 55.510 111.380 1.00 0.00

ATOM 54 BENd PHE 23 57.280 57.150 103.830 1.00 0.00

ATOM 55 4SES PHE 23 54.630 57.080 101.980 1.00 0.00

ATOM 56 4SES PHE 23 52.240 55.970 102.550 1.00 0.00

ATOM 57 4SES PHE 23 52.720 56.940 100.080 1.00 0.00

ATOM 58 BENd THR 24 56.270 60.270 105.110 1.00 0.00

ATOM 59 1SEP THR 24 57.730 61.200 107.050 1.00 0.00

ATOM 60 BENd LEU 25 53.640 62.210 103.600 1.00 0.00

ATOM 61 1SEA LEU 25 50.850 60.410 103.720 1.00 0.00

ATOM 62 BENd HIS 26 53.860 66.010 103.600 1.00 0.00

ATOM 63 4SES HIS 26 56.730 66.860 104.580 1.00 0.00

ATOM 64 1SES HIS 26 57.950 69.150 105.310 1.00 0.00

ATOM 65 1SES HIS 26 58.140 66.970 106.880 1.00 0.00

ATOM 66 BTNd ARG 27 52.290 68.500 101.810 1.00 0.00

ATOM 67 0STN ARG 27 53.820 71.060 101.830 1.00 0.00

ATOM 68 STQd ARG 27 55.990 73.040 102.600 1.00 0.00

ATOM 69 BTNd ASN 28 52.050 67.590 98.430 1.00 0.00

ATOM 70 5STP ASN 28 50.270 69.260 97.400 1.00 0.00

ATOM 71 BENd GLU 29 54.410 65.200 98.440 1.00 0.00

ATOM 72 SEQa GLU 29 57.640 66.280 99.920 1.00 0.00

ATOM 73 BENd LEU 30 55.070 61.860 99.170 1.00 0.00

ATOM 74 1SEA LEU 30 52.340 60.840 97.380 1.00 0.00

ATOM 75 BENd LYS 31 58.180 60.670 99.960 1.00 0.00

ATOM 76 3SEC LYS 31 59.980 61.700 102.090 1.00 0.00

ATOM 77 SEQd LYS 31 61.150 63.680 103.010 1.00 0.00

ATOM 78 BENd TYR 32 59.810 57.390 100.480 1.00 0.00

ATOM 79 4SES TYR 32 57.750 56.480 98.580 1.00 0.00

ATOM 80 4SES TYR 32 55.490 56.710 97.110 1.00 0.00

ATOM 81 1SES TYR 32 57.800 55.980 95.930 1.00 0.00

ATOM 82 BENd PHE 33 63.160 56.300 101.630 1.00 0.00

ATOM 83 4SES PHE 33 63.040 57.460 104.320 1.00 0.00

ATOM 84 4SES PHE 33 61.800 58.370 106.540 1.00 0.00

ATOM 85 4SES PHE 33 63.400 60.010 105.120 1.00 0.00

ATOM 86 5BSP LYS 34 64.890 53.270 101.850 1.00 0.00

ATOM 87 3SSC LYS 34 67.920 54.040 100.980 1.00 0.00

ATOM 88 SSQd LYS 34 69.980 55.980 100.050 1.00 0.00

ATOM 89 5BSP ASP 35 63.530 52.100 104.720 1.00 0.00

ATOM 90 SSQa ASP 35 63.070 49.060 104.560 1.00 0.00

ATOM 91 BTNd GLN 36 64.120 52.740 107.720 1.00 0.00

ATOM 92 4STP GLN 36 62.580 49.780 109.760 1.00 0.00

ATOM 93 BTNd MET 37 65.800 54.960 108.110 1.00 0.00

ATOM 94 5STC MET 37 63.930 56.890 111.050 1.00 0.00

ATOM 95 5BCP SER 38 67.870 55.750 105.500 1.00 0.00

ATOM 96 1SCP SER 38 70.140 55.000 105.090 1.00 0.00

ATOM 97 BSNa PRO 39 68.700 59.480 105.660 1.00 0.00

ATOM 98 2SSA PRO 39 66.710 59.660 108.080 1.00 0.00

ATOM 99 5BSP GLU 40 68.750 60.620 102.320 1.00 0.00

ATOM 100 SSQa GLU 40 72.490 60.150 101.990 1.00 0.00

ATOM 101 BCNa PRO 41 64.910 59.650 100.230 1.00 0.00

ATOM 102 2SCA PRO 41 64.490 62.740 99.360 1.00 0.00

ATOM 103 5BSP ILE 42 64.490 58.190 97.120 1.00 0.00

ATOM 104 1SSA ILE 42 67.030 56.520 96.500 1.00 0.00

ATOM 105 BENd ARG 43 62.030 58.470 95.620 1.00 0.00

ATOM 106 0SEN ARG 43 62.140 56.180 93.380 1.00 0.00

ATOM 107 SEQd ARG 43 64.620 54.180 92.030 1.00 0.00

ATOM 108 BENd ILE 44 59.350 61.120 95.540 1.00 0.00

ATOM 109 1SEA ILE 44 60.500 63.900 96.270 1.00 0.00

ATOM 110 BENd LEU 45 56.250 61.350 94.950 1.00 0.00

ATOM 111 1SEA LEU 45 56.340 60.480 91.810 1.00 0.00

ATOM 112 BENd ASP 46 54.210 64.400 93.740 1.00 0.00

ATOM 113 SEQa ASP 46 56.850 65.940 93.110 1.00 0.00

ATOM 114 BHNd LEU 47 50.850 64.730 94.160 1.00 0.00

ATOM 115 1SHA LEU 47 48.630 64.150 96.330 1.00 0.00

ATOM 116 BHNd THR 48 50.820 66.970 91.860 1.00 0.00

ATOM 117 1SHP THR 48 51.310 69.420 92.590 1.00 0.00

ATOM 118 BHNd GLU 49 51.580 64.850 88.620 1.00 0.00

ATOM 119 SHQa GLU 49 55.370 64.110 88.930 1.00 0.00

ATOM 120 5BCP CYS 50 50.600 61.630 90.270 1.00 0.00

ATOM 121 5SCC CYS 50 51.680 59.890 92.420 1.00 0.00

ATOM 122 5BCP SER 51 47.180 62.220 91.830 1.00 0.00

ATOM 123 1SCP SER 51 45.660 64.090 90.970 1.00 0.00

ATOM 124 0BEN ALA 52 46.850 60.120 93.370 1.00 0.00

ATOM 125 BENd VAL 53 44.840 57.910 94.470 1.00 0.00

ATOM 126 2SEA VAL 53 45.620 57.110 96.870 1.00 0.00

ATOM 127 BENd GLN 54 42.130 55.290 93.750 1.00 0.00

ATOM 128 4SEP GLN 54 40.020 52.840 91.680 1.00 0.00

ATOM 129 BENd PHE 55 41.640 52.840 96.380 1.00 0.00

ATOM 130 4SES PHE 55 38.930 53.130 97.500 1.00 0.00

ATOM 131 4SES PHE 55 37.310 52.270 99.480 1.00 0.00

ATOM 132 4SES PHE 55 37.130 54.810 98.600 1.00 0.00

ATOM 133 BENd ASP 56 41.400 49.370 96.050 1.00 0.00

ATOM 134 SEQa ASP 56 43.030 48.640 93.630 1.00 0.00

ATOM 135 5BCP TYR 57 38.930 47.530 98.080 1.00 0.00

ATOM 136 4SCS TYR 57 41.140 48.230 100.640 1.00 0.00

ATOM 137 4SCS TYR 57 42.390 50.200 102.000 1.00 0.00

ATOM 138 1SCS TYR 57 41.170 48.200 103.340 1.00 0.00

ATOM 139 5BSP SER 58 38.390 44.390 96.620 1.00 0.00

ATOM 140 1SSP SER 58 37.620 42.460 97.910 1.00 0.00

ATOM 141 5BSP GLN 59 42.870 43.090 94.220 1.00 0.00

ATOM 142 4SSP GLN 59 40.940 44.020 91.530 1.00 0.00

ATOM 143 5BSP GLU 60 44.820 41.650 95.090 1.00 0.00

ATOM 144 SSQa GLU 60 41.930 39.210 97.190 1.00 0.00

ATOM 145 5BSP ARG 61 46.620 41.070 98.200 1.00 0.00

ATOM 146 0SSN ARG 61 49.090 39.000 97.940 1.00 0.00

ATOM 147 SSQd ARG 61 50.680 36.340 96.420 1.00 0.00

ATOM 148 5BSP VAL 62 44.320 42.720 101.170 1.00 0.00

ATOM 149 2SSA VAL 62 42.330 43.530 99.610 1.00 0.00

ATOM 150 5BSP ASN 63 45.500 45.810 102.920 1.00 0.00

ATOM 151 5SSP ASN 63 47.440 44.200 104.710 1.00 0.00

ATOM 152 BENd CYS 64 45.500 48.470 99.090 1.00 0.00

ATOM 153 5SEC CYS 64 45.500 46.170 97.060 1.00 0.00

ATOM 154 BENd PHE 65 45.370 51.680 97.610 1.00 0.00

ATOM 155 4SES PHE 65 46.940 53.180 99.540 1.00 0.00

ATOM 156 4SES PHE 65 48.440 55.300 100.280 1.00 0.00

ATOM 157 4SES PHE 65 48.200 53.170 101.930 1.00 0.00

ATOM 158 BENd CYS 66 47.130 52.390 94.520 1.00 0.00

ATOM 159 5SEC CYS 66 45.650 52.250 91.980 1.00 0.00

ATOM 160 BENd LEU 67 48.860 55.590 93.280 1.00 0.00

ATOM 161 1SEA LEU 67 50.600 56.510 95.920 1.00 0.00

ATOM 162 BENd VAL 68 48.440 56.710 90.300 1.00 0.00

ATOM 163 2SEA VAL 68 45.900 56.850 89.560 1.00 0.00

ATOM 164 BENd PHE 69 50.170 58.610 87.960 1.00 0.00

ATOM 165 4SES PHE 69 48.130 60.060 86.380 1.00 0.00

ATOM 166 4SES PHE 69 45.790 61.380 86.670 1.00 0.00

ATOM 167 4SES PHE 69 46.270 59.960 84.430 1.00 0.00

ATOM 168 0BTN PRO 70 53.420 59.430 87.180 1.00 0.00

ATOM 169 2STA PRO 70 52.810 61.800 85.100 1.00 0.00

ATOM 170 BTNd PHE 71 54.420 56.830 85.980 1.00 0.00

ATOM 171 4STS PHE 71 56.090 57.840 83.530 1.00 0.00

ATOM 172 4STS PHE 71 57.130 59.600 81.760 1.00 0.00

ATOM 173 4STS PHE 71 58.080 57.070 81.870 1.00 0.00

ATOM 174 BENd ARG 72 52.640 53.870 86.750 1.00 0.00

ATOM 175 0SEN ARG 72 54.720 51.750 87.290 1.00 0.00

ATOM 176 SEQd ARG 72 58.010 52.170 87.850 1.00 0.00

ATOM 177 BENd THR 73 50.250 52.750 89.030 1.00 0.00

ATOM 178 1SEP THR 73 48.420 51.160 88.100 1.00 0.00

ATOM 179 BENd PHE 74 51.440 51.400 91.980 1.00 0.00

ATOM 180 4SES PHE 74 53.710 53.560 92.500 1.00 0.00

ATOM 181 4SES PHE 74 56.200 54.230 91.710 1.00 0.00

ATOM 182 4SES PHE 74 54.120 55.860 91.150 1.00 0.00

ATOM 183 BENd TYR 75 49.810 49.190 94.130 1.00 0.00

ATOM 184 4SES TYR 75 48.290 47.700 92.230 1.00 0.00

ATOM 185 4SES TYR 75 46.230 46.930 90.660 1.00 0.00

ATOM 186 1SES TYR 75 47.540 45.110 92.170 1.00 0.00

ATOM 187 BENd LEU 76 50.180 49.210 97.750 1.00 0.00

ATOM 188 1SEA LEU 76 51.490 52.200 97.380 1.00 0.00

ATOM 189 BENd CYS 77 49.270 48.080 100.700 1.00 0.00

ATOM 190 5SEC CYS 77 49.520 45.160 100.370 1.00 0.00

ATOM 191 4BCP ALA 78 49.380 48.780 104.400 1.00 0.00

ATOM 192 5BSP LYS 79 50.430 48.420 106.670 1.00 0.00

ATOM 193 3SSC LYS 79 52.400 48.500 109.010 1.00 0.00

ATOM 194 SSQd LYS 79 52.250 48.640 111.400 1.00 0.00

ATOM 195 5BSP THR 80 46.900 47.040 108.490 1.00 0.00

ATOM 196 1SSP THR 80 47.810 44.900 109.660 1.00 0.00

ATOM 197 BHNd GLY 81 44.110 47.290 107.320 1.00 0.00

ATOM 198 BHNd VAL 82 42.930 50.010 108.930 1.00 0.00

ATOM 199 2SHA VAL 82 40.370 50.340 108.350 1.00 0.00

ATOM 200 BHNd GLU 83 45.530 51.690 108.310 1.00 0.00

ATOM 201 SHQa GLU 83 47.140 50.990 111.910 1.00 0.00

ATOM 202 0BHN ALA 84 45.640 51.660 105.530 1.00 0.00

ATOM 203 0BHN ASP 85 42.240 53.760 105.000 1.00 0.00

ATOM 204 SHQa ASP 85 39.570 53.970 104.070 1.00 0.00

ATOM 205 0BHN GLU 86 43.320 56.170 106.490 1.00 0.00

ATOM 206 SHQa GLU 86 41.430 55.350 109.810 1.00 0.00

ATOM 207 0BHN TRP 87 45.790 56.600 104.630 1.00 0.00

ATOM 208 4SHS TRP 87 48.800 56.130 105.240 1.00 0.00

ATOM 209 1SHS TRP 87 49.930 53.790 105.970 1.00 0.00

ATOM 210 4SHS TRP 87 49.430 55.680 107.830 1.00 0.00

ATOM 211 4SHS TRP 87 50.330 53.240 108.580 1.00 0.00

ATOM 212 0BHN ILE 88 44.010 57.040 101.660 1.00 0.00

ATOM 213 1SHA ILE 88 42.140 55.040 100.230 1.00 0.00

ATOM 214 0BHN LYS 89 42.500 59.170 102.860 1.00 0.00

ATOM 215 3SHC LYS 89 39.350 60.380 101.790 1.00 0.00

ATOM 216 SHQd LYS 89 36.890 59.400 101.580 1.00 0.00

ATOM 217 0BHN ILE 90 45.350 61.950 102.960 1.00 0.00

ATOM 218 1SHA ILE 90 46.470 61.480 105.810 1.00 0.00

ATOM 219 BHNa LEU 91 46.160 62.010 99.560 1.00 0.00

ATOM 220 1SHA LEU 91 48.300 59.890 99.660 1.00 0.00

ATOM 221 BHNa ARG 92 43.070 62.570 98.760 1.00 0.00

ATOM 222 0SHN ARG 92 42.990 62.530 95.450 1.00 0.00

ATOM 223 SHQd ARG 92 41.540 64.630 92.890 1.00 0.00

ATOM 224 BHNa TRP 93 42.620 65.330 100.660 1.00 0.00

ATOM 225 4SHS TRP 93 41.580 64.850 103.600 1.00 0.00

ATOM 226 1SHS TRP 93 40.150 65.970 105.590 1.00 0.00

ATOM 227 4SHS TRP 93 42.740 65.340 105.980 1.00 0.00

ATOM 228 4SHS TRP 93 41.340 66.590 107.930 1.00 0.00

ATOM 229 BHNa LYS 94 45.840 67.210 99.160 1.00 0.00

ATOM 230 3SHC LYS 94 45.740 70.220 99.750 1.00 0.00

ATOM 231 SHQd LYS 94 45.540 72.020 97.470 1.00 0.00

ATOM 232 5BCP LEU 95 44.810 67.220 95.920 1.00 0.00

ATOM 233 1SCA LEU 95 46.990 68.900 94.070 1.00 0.00

ATOM 234 BCQa SER 96 40.940 68.060 96.560 1.00 0.00

ATOM 235 1SCP SER 96 40.730 69.970 94.750 1.00 0.00

ATOM 236 NC3 POP 97 60.670 24.700 42.950 1.00 0.00

ATOM 237 PO4 POP 97 60.110 29.290 43.340 1.00 0.00

ATOM 238 GL1 POP 97 59.570 28.330 46.390 1.00 0.00

ATOM 239 GL2 POP 97 60.540 25.220 48.200 1.00 0.00

ATOM 240 C1A POP 97 64.420 25.680 50.520 1.00 0.00

ATOM 241 C2A POP 97 65.300 24.700 54.960 1.00 0.00

ATOM 242 C3A POP 97 64.110 23.750 59.620 1.00 0.00

ATOM 243 C4A POP 97 61.440 25.520 62.870 1.00 0.00

ATOM 244 C1B POP 97 57.210 29.000 49.660 1.00 0.00

ATOM 245 C2B POP 97 56.130 30.860 53.920 1.00 0.00

ATOM 246 D3B POP 97 56.670 30.730 58.260 1.00 0.00

ATOM 247 C4B POP 97 59.030 30.110 61.280 1.00 0.00

ATOM 248 C5B POP 97 63.460 32.220 61.350 1.00 0.00

ATOM 249 NC3 POP 98 47.650 100.160 46.070 1.00 0.00

ATOM 250 PO4 POP 98 50.030 95.340 46.100 1.00 0.00

ATOM 251 GL1 POP 98 48.660 97.200 50.070 1.00 0.00

ATOM 252 GL2 POP 98 46.360 99.790 50.970 1.00 0.00

ATOM 253 C1A POP 98 45.090 101.080 55.420 1.00 0.00

ATOM 254 C2A POP 98 45.800 104.100 59.010 1.00 0.00

ATOM 255 C3A POP 98 46.560 102.670 63.230 1.00 0.00

ATOM 256 C4A POP 98 43.720 100.400 65.250 1.00 0.00

ATOM 257 C1B POP 98 49.180 95.830 53.440 1.00 0.00

ATOM 258 C2B POP 98 48.390 92.010 54.990 1.00 0.00

ATOM 259 D3B POP 98 50.490 88.890 57.760 1.00 0.00

ATOM 260 C4B POP 98 50.190 88.440 61.800 1.00 0.00

ATOM 261 C5B POP 98 47.810 86.250 65.300 1.00 0.00

ATOM 262 NC3 POP 99 13.060 94.430 38.840 1.00 0.00

ATOM 263 PO4 POP 99 11.940 95.900 43.150 1.00 0.00

ATOM 264 GL1 POP 99 9.520 96.650 47.750 1.00 0.00

ATOM 265 GL2 POP 99 9.710 99.870 49.900 1.00 0.00

ATOM 266 C1A POP 99 11.810 97.970 52.570 1.00 0.00

ATOM 267 C2A POP 99 16.050 96.140 53.440 1.00 0.00

ATOM 268 C3A POP 99 19.410 97.890 56.940 1.00 0.00

ATOM 269 C4A POP 99 18.730 98.740 61.280 1.00 0.00

ATOM 270 C1B POP 99 8.450 94.280 51.290 1.00 0.00

ATOM 271 C2B POP 99 8.650 93.960 55.540 1.00 0.00

ATOM 272 D3B POP 99 6.880 94.890 59.070 1.00 0.00

ATOM 273 C4B POP 99 7.010 99.050 61.980 1.00 0.00

ATOM 274 C5B POP 99 10.150 102.870 61.040 1.00 0.00

ATOM 275 NC3 POP 100 96.400 72.660 87.270 1.00 0.00

ATOM 276 PO4 POP 100 92.450 73.650 87.850 1.00 0.00

ATOM 277 GL1 POP 100 92.410 74.090 83.520 1.00 0.00

ATOM 278 GL2 POP 100 95.830 74.510 82.410 1.00 0.00

ATOM 279 C1A POP 100 95.870 78.110 78.970 1.00 0.00

ATOM 280 C2A POP 100 94.280 82.200 78.570 1.00 0.00

ATOM 281 C3A POP 100 94.480 84.520 74.900 1.00 0.00

ATOM 282 C4A POP 100 93.000 85.500 71.100 1.00 0.00

ATOM 283 C1B POP 100 92.510 74.760 78.480 1.00 0.00

ATOM 284 C2B POP 100 91.850 77.080 75.680 1.00 0.00

ATOM 285 D3B POP 100 95.050 78.440 74.130 1.00 0.00

ATOM 286 C4B POP 100 96.980 76.440 70.300 1.00 0.00

ATOM 287 C5B POP 100 100.220 73.330 68.360 1.00 0.00

ATOM 288 NC3 POP 101 5.110 29.020 89.030 1.00 0.00

ATOM 289 PO4 POP 101 3.980 24.180 88.780 1.00 0.00

ATOM 290 GL1 POP 101 3.220 24.560 85.010 1.00 0.00

ATOM 291 GL2 POP 101 2.990 27.670 84.910 1.00 0.00

ATOM 292 C1A POP 101 3.940 30.150 81.350 1.00 0.00

ATOM 293 C2A POP 101 4.060 33.220 78.540 1.00 0.00

ATOM 294 C3A POP 101 1.800 33.600 74.720 1.00 0.00

ATOM 295 C4A POP 101 0.380 31.170 71.850 1.00 0.00

ATOM 296 C1B POP 101 3.640 25.050 79.990 1.00 0.00

ATOM 297 C2B POP 101 6.170 28.450 77.490 1.00 0.00

ATOM 298 D3B POP 101 5.760 30.880 74.740 1.00 0.00

ATOM 299 C4B POP 101 5.910 31.010 70.090 1.00 0.00

ATOM 300 C5B POP 101 5.350 30.160 65.570 1.00 0.00

ATOM 301 NC3 POP 102 9.240 7.580 39.460 1.00 0.00

ATOM 302 PO4 POP 102 6.340 9.730 43.860 1.00 0.00

ATOM 303 GL1 POP 102 3.490 11.240 47.680 1.00 0.00

ATOM 304 GL2 POP 102 4.960 13.630 48.990 1.00 0.00

ATOM 305 C1A POP 102 8.210 12.670 51.630 1.00 0.00

ATOM 306 C2A POP 102 9.760 12.430 56.100 1.00 0.00

ATOM 307 C3A POP 102 12.090 10.890 60.300 1.00 0.00

ATOM 308 C4A POP 102 13.520 11.940 64.340 1.00 0.00

ATOM 309 C1B POP 102 1.340 11.630 51.870 1.00 0.00

ATOM 310 C2B POP 102 2.170 9.400 56.070 1.00 0.00

ATOM 311 D3B POP 102 2.270 6.610 59.630 1.00 0.00

ATOM 312 C4B POP 102 1.550 8.590 62.520 1.00 0.00

ATOM 313 C5B POP 102 1.790 10.550 66.260 1.00 0.00

ATOM 314 NC3 POP 103 38.350 18.210 87.230 1.00 0.00

ATOM 315 PO4 POP 103 41.490 16.250 86.170 1.00 0.00

ATOM 316 GL1 POP 103 39.250 15.230 83.090 1.00 0.00

ATOM 317 GL2 POP 103 36.030 14.570 84.170 1.00 0.00

ATOM 318 C1A POP 103 33.500 13.800 80.450 1.00 0.00

ATOM 319 C2A POP 103 31.640 12.940 76.150 1.00 0.00

ATOM 320 C3A POP 103 29.220 13.380 71.390 1.00 0.00

ATOM 321 C4A POP 103 25.120 14.210 69.880 1.00 0.00

ATOM 322 C1B POP 103 37.160 17.370 79.820 1.00 0.00

ATOM 323 C2B POP 103 35.360 16.420 76.370 1.00 0.00

ATOM 324 D3B POP 103 32.820 17.940 72.860 1.00 0.00

ATOM 325 C4B POP 103 33.170 17.490 68.650 1.00 0.00

ATOM 326 C5B POP 103 32.410 15.420 64.080 1.00 0.00

ATOM 327 NC3 POP 104 114.180 97.170 39.430 1.00 0.00

ATOM 328 PO4 POP 104 111.120 97.840 43.240 1.00 0.00

ATOM 329 GL1 POP 104 111.020 98.350 48.140 1.00 0.00

ATOM 330 GL2 POP 104 108.840 95.410 48.250 1.00 0.00

ATOM 331 C1A POP 104 106.690 95.760 51.860 1.00 0.00

ATOM 332 C2A POP 104 106.780 97.950 55.670 1.00 0.00

ATOM 333 C3A POP 104 107.070 99.330 60.050 1.00 0.00

ATOM 334 C4A POP 104 106.430 98.430 63.270 1.00 0.00

ATOM 335 C1B POP 104 109.480 99.580 51.080 1.00 0.00

ATOM 336 C2B POP 104 106.730 102.750 51.760 1.00 0.00

ATOM 337 D3B POP 104 105.370 106.960 52.320 1.00 0.00

ATOM 338 C4B POP 104 106.340 109.230 56.030 1.00 0.00

ATOM 339 C5B POP 104 110.240 110.860 58.870 1.00 0.00

ATOM 340 NC3 POP 105 13.330 103.120 86.310 1.00 0.00

ATOM 341 PO4 POP 105 17.520 100.590 87.540 1.00 0.00

ATOM 342 GL1 POP 105 18.400 101.180 83.690 1.00 0.00

ATOM 343 GL2 POP 105 15.450 102.010 81.420 1.00 0.00

ATOM 344 C1A POP 105 13.290 98.800 80.820 1.00 0.00

ATOM 345 C2A POP 105 11.200 96.540 77.020 1.00 0.00

ATOM 346 C3A POP 105 11.540 94.640 72.780 1.00 0.00

ATOM 347 C4A POP 105 14.150 96.390 68.770 1.00 0.00

ATOM 348 C1B POP 105 17.910 98.180 79.900 1.00 0.00

ATOM 349 C2B POP 105 16.290 95.700 77.820 1.00 0.00

ATOM 350 D3B POP 105 15.430 92.440 75.090 1.00 0.00

ATOM 351 C4B POP 105 18.220 92.420 72.590 1.00 0.00

ATOM 352 C5B POP 105 20.250 89.300 70.030 1.00 0.00

ATOM 353 NC3 POP 106 62.510 90.680 86.430 1.00 0.00

ATOM 354 PO4 POP 106 58.800 88.770 84.090 1.00 0.00

ATOM 355 GL1 POP 106 62.750 87.610 82.240 1.00 0.00

ATOM 356 GL2 POP 106 64.960 84.630 82.080 1.00 0.00

ATOM 357 C1A POP 106 66.150 87.620 77.870 1.00 0.00

ATOM 358 C2A POP 106 65.060 90.960 75.290 1.00 0.00

ATOM 359 C3A POP 106 66.660 91.760 71.710 1.00 0.00

ATOM 360 C4A POP 106 70.740 93.360 71.010 1.00 0.00

ATOM 361 C1B POP 106 61.220 87.270 78.170 1.00 0.00

ATOM 362 C2B POP 106 61.920 86.770 73.570 1.00 0.00

ATOM 363 D3B POP 106 64.360 88.200 69.540 1.00 0.00

ATOM 364 C4B POP 106 68.800 87.650 69.020 1.00 0.00

ATOM 365 C5B POP 106 68.990 85.370 65.300 1.00 0.00

ATOM 366 NC3 POP 107 95.350 6.370 43.580 1.00 0.00

ATOM 367 PO4 POP 107 97.570 9.050 46.710 1.00 0.00

ATOM 368 GL1 POP 107 95.840 9.610 50.940 1.00 0.00

ATOM 369 GL2 POP 107 97.100 9.480 53.210 1.00 0.00

ATOM 370 C1A POP 107 96.570 8.670 57.250 1.00 0.00

ATOM 371 C2A POP 107 96.030 8.810 60.810 1.00 0.00

ATOM 372 C3A POP 107 97.780 10.470 64.480 1.00 0.00

ATOM 373 C4A POP 107 97.230 12.120 68.820 1.00 0.00

ATOM 374 C1B POP 107 93.190 6.180 53.720 1.00 0.00

ATOM 375 C2B POP 107 93.110 4.800 57.220 1.00 0.00

ATOM 376 D3B POP 107 91.780 5.730 62.120 1.00 0.00

ATOM 377 C4B POP 107 91.260 1.620 64.020 1.00 0.00

ATOM 378 C5B POP 107 93.620 -1.270 66.080 1.00 0.00

ATOM 379 NC3 POP 108 69.020 26.900 85.440 1.00 0.00

ATOM 380 PO4 POP 108 68.360 22.450 85.400 1.00 0.00

ATOM 381 GL1 POP 108 64.990 23.200 82.340 1.00 0.00

ATOM 382 GL2 POP 108 63.580 26.050 83.280 1.00 0.00

ATOM 383 C1A POP 108 63.880 27.690 79.620 1.00 0.00

ATOM 384 C2A POP 108 62.470 28.680 75.720 1.00 0.00

ATOM 385 C3A POP 108 62.850 26.900 71.640 1.00 0.00

ATOM 386 C4A POP 108 61.260 24.070 67.880 1.00 0.00

ATOM 387 C1B POP 108 66.480 21.970 78.790 1.00 0.00

ATOM 388 C2B POP 108 69.700 20.220 75.960 1.00 0.00

ATOM 389 D3B POP 108 70.970 16.190 73.550 1.00 0.00

ATOM 390 C4B POP 108 74.660 16.560 70.790 1.00 0.00

ATOM 391 C5B POP 108 77.780 15.490 74.470 1.00 0.00

ATOM 392 NC3 POP 109 57.550 38.360 44.840 1.00 0.00

ATOM 393 PO4 POP 109 60.480 41.880 44.760 1.00 0.00

ATOM 394 GL1 POP 109 64.250 41.250 46.940 1.00 0.00

ATOM 395 GL2 POP 109 63.310 38.350 47.440 1.00 0.00

ATOM 396 C1A POP 109 63.520 36.390 50.990 1.00 0.00

ATOM 397 C2A POP 109 64.940 35.420 54.350 1.00 0.00

ATOM 398 C3A POP 109 68.020 33.890 57.720 1.00 0.00

ATOM 399 C4A POP 109 69.600 33.320 62.090 1.00 0.00

ATOM 400 C1B POP 109 66.630 40.250 51.000 1.00 0.00

ATOM 401 C2B POP 109 67.810 40.530 54.970 1.00 0.00

ATOM 402 D3B POP 109 66.540 41.440 58.840 1.00 0.00

ATOM 403 C4B POP 109 62.240 41.290 60.780 1.00 0.00

ATOM 404 C5B POP 109 62.050 41.070 65.860 1.00 0.00

ATOM 405 NC3 POP 110 43.650 53.270 87.600 1.00 0.00

ATOM 406 PO4 POP 110 41.450 55.230 87.260 1.00 0.00

ATOM 407 GL1 POP 110 42.910 56.990 83.560 1.00 0.00

ATOM 408 GL2 POP 110 42.060 54.220 81.810 1.00 0.00

ATOM 409 C1A POP 110 38.290 52.720 78.870 1.00 0.00

ATOM 410 C2A POP 110 37.090 54.870 75.380 1.00 0.00

ATOM 411 C3A POP 110 34.640 53.530 71.280 1.00 0.00

ATOM 412 C4A POP 110 33.040 51.770 68.600 1.00 0.00

ATOM 413 C1B POP 110 44.850 57.710 79.740 1.00 0.00

ATOM 414 C2B POP 110 49.190 59.210 76.800 1.00 0.00

ATOM 415 D3B POP 110 53.830 59.130 78.410 1.00 0.00

ATOM 416 C4B POP 110 56.150 55.090 76.540 1.00 0.00

ATOM 417 C5B POP 110 59.690 52.920 74.520 1.00 0.00

ATOM 418 NC3 POP 111 38.120 60.710 93.160 1.00 0.00

ATOM 419 PO4 POP 111 42.380 60.130 90.730 1.00 0.00

ATOM 420 GL1 POP 111 40.860 60.270 87.560 1.00 0.00

ATOM 421 GL2 POP 111 38.110 61.330 87.710 1.00 0.00

ATOM 422 C1A POP 111 34.970 61.860 85.140 1.00 0.00

ATOM 423 C2A POP 111 33.680 63.000 81.570 1.00 0.00

ATOM 424 C3A POP 111 31.130 63.840 78.350 1.00 0.00

ATOM 425 C4A POP 111 29.600 63.910 74.030 1.00 0.00

ATOM 426 C1B POP 111 40.720 62.230 83.120 1.00 0.00

ATOM 427 C2B POP 111 38.560 63.130 80.030 1.00 0.00

ATOM 428 D3B POP 111 35.680 61.250 76.890 1.00 0.00

ATOM 429 C4B POP 111 34.760 62.240 72.910 1.00 0.00

ATOM 430 C5B POP 111 34.150 61.760 68.520 1.00 0.00

ATOM 431 NC3 POP 112 90.880 58.000 45.480 1.00 0.00

ATOM 432 PO4 POP 112 88.700 61.500 47.150 1.00 0.00

ATOM 433 GL1 POP 112 89.230 63.030 50.990 1.00 0.00

ATOM 434 GL2 POP 112 86.250 64.490 52.890 1.00 0.00

ATOM 435 C1A POP 112 84.830 63.520 56.840 1.00 0.00

ATOM 436 C2A POP 112 86.580 63.730 60.520 1.00 0.00

ATOM 437 C3A POP 112 86.260 62.000 64.300 1.00 0.00

ATOM 438 C4A POP 112 85.550 61.850 68.180 1.00 0.00

ATOM 439 C1B POP 112 92.360 63.540 54.110 1.00 0.00

ATOM 440 C2B POP 112 94.670 60.090 56.360 1.00 0.00

ATOM 441 D3B POP 112 95.750 58.140 60.840 1.00 0.00

ATOM 442 C4B POP 112 95.320 59.270 64.150 1.00 0.00

ATOM 443 C5B POP 112 98.110 60.640 67.940 1.00 0.00

ATOM 444 NC3 POP 113 9.990 106.260 89.980 1.00 0.00

ATOM 445 PO4 POP 113 10.550 108.540 86.640 1.00 0.00

ATOM 446 GL1 POP 113 8.480 110.130 81.970 1.00 0.00

ATOM 447 GL2 POP 113 7.210 112.920 81.530 1.00 0.00

ATOM 448 C1A POP 113 9.630 113.060 77.140 1.00 0.00

ATOM 449 C2A POP 113 11.250 111.600 73.040 1.00 0.00

ATOM 450 C3A POP 113 10.880 108.150 69.130 1.00 0.00

ATOM 451 C4A POP 113 13.360 105.950 65.150 1.00 0.00

ATOM 452 C1B POP 113 6.960 109.580 78.080 1.00 0.00

ATOM 453 C2B POP 113 7.270 108.690 74.300 1.00 0.00

ATOM 454 D3B POP 113 7.660 104.490 71.650 1.00 0.00

ATOM 455 C4B POP 113 10.650 102.850 69.970 1.00 0.00

ATOM 456 C5B POP 113 12.720 99.830 71.530 1.00 0.00

ATOM 457 NC3 POP 114 75.180 89.990 95.500 1.00 0.00

ATOM 458 PO4 POP 114 74.660 90.340 91.030 1.00 0.00

ATOM 459 GL1 POP 114 75.560 89.130 85.640 1.00 0.00

ATOM 460 GL2 POP 114 74.580 87.220 84.180 1.00 0.00

ATOM 461 C1A POP 114 74.470 86.580 80.320 1.00 0.00

ATOM 462 C2A POP 114 74.320 85.250 75.790 1.00 0.00

ATOM 463 C3A POP 114 77.910 83.520 72.670 1.00 0.00

ATOM 464 C4A POP 114 79.130 83.660 68.510 1.00 0.00

ATOM 465 C1B POP 114 77.960 91.010 82.540 1.00 0.00

ATOM 466 C2B POP 114 81.410 94.110 82.160 1.00 0.00

ATOM 467 D3B POP 114 85.850 95.390 82.220 1.00 0.00

ATOM 468 C4B POP 114 89.140 95.940 78.980 1.00 0.00

ATOM 469 C5B POP 114 90.330 94.970 75.340 1.00 0.00

ATOM 470 NC3 POP 115 93.360 94.990 41.270 1.00 0.00

ATOM 471 PO4 POP 115 93.760 93.850 45.420 1.00 0.00

ATOM 472 GL1 POP 115 93.880 93.410 49.970 1.00 0.00

ATOM 473 GL2 POP 115 96.780 94.030 51.070 1.00 0.00

ATOM 474 C1A POP 115 97.320 93.660 55.130 1.00 0.00

ATOM 475 C2A POP 115 97.600 95.190 58.190 1.00 0.00

ATOM 476 C3A POP 115 97.840 97.570 60.640 1.00 0.00

ATOM 477 C4A POP 115 100.100 96.950 63.470 1.00 0.00

ATOM 478 C1B POP 115 92.160 92.760 54.040 1.00 0.00

ATOM 479 C2B POP 115 90.070 92.810 58.160 1.00 0.00

ATOM 480 D3B POP 115 90.170 91.280 62.980 1.00 0.00

ATOM 481 C4B POP 115 87.740 91.230 66.710 1.00 0.00

ATOM 482 C5B POP 115 83.800 88.090 67.890 1.00 0.00

ATOM 483 NC3 POP 116 48.830 32.350 87.120 1.00 0.00

ATOM 484 PO4 POP 116 45.440 33.680 84.410 1.00 0.00

ATOM 485 GL1 POP 116 43.990 35.240 81.030 1.00 0.00

ATOM 486 GL2 POP 116 41.260 35.560 81.210 1.00 0.00

ATOM 487 C1A POP 116 39.770 35.300 76.260 1.00 0.00

ATOM 488 C2A POP 116 37.360 35.690 72.520 1.00 0.00

ATOM 489 C3A POP 116 35.850 38.360 68.520 1.00 0.00

ATOM 490 C4A POP 116 35.900 42.490 65.600 1.00 0.00

ATOM 491 C1B POP 116 44.420 36.130 76.030 1.00 0.00

ATOM 492 C2B POP 116 45.760 38.360 72.100 1.00 0.00

ATOM 493 D3B POP 116 48.540 41.020 69.620 1.00 0.00

ATOM 494 C4B POP 116 51.360 42.420 69.580 1.00 0.00

ATOM 495 C5B POP 116 54.600 45.150 71.750 1.00 0.00

ATOM 496 NC3 POP 117 100.540 75.700 49.390 1.00 0.00

ATOM 497 PO4 POP 117 101.040 79.540 50.140 1.00 0.00

ATOM 498 GL1 POP 117 103.870 77.510 52.700 1.00 0.00

ATOM 499 GL2 POP 117 102.580 76.980 55.860 1.00 0.00

ATOM 500 C1A POP 117 102.880 74.940 59.760 1.00 0.00

ATOM 501 C2A POP 117 106.080 72.310 64.030 1.00 0.00

ATOM 502 C3A POP 117 107.190 70.980 68.130 1.00 0.00

ATOM 503 C4A POP 117 110.770 70.870 70.950 1.00 0.00

ATOM 504 C1B POP 117 105.340 72.970 54.370 1.00 0.00

ATOM 505 C2B POP 117 104.350 70.600 58.060 1.00 0.00

ATOM 506 D3B POP 117 100.330 68.040 59.010 1.00 0.00

ATOM 507 C4B POP 117 98.660 67.300 62.790 1.00 0.00

ATOM 508 C5B POP 117 96.250 64.890 65.140 1.00 0.00

ATOM 509 NC3 POP 118 19.690 4.170 86.460 1.00 0.00

ATOM 510 PO4 POP 118 23.510 3.800 82.950 1.00 0.00

ATOM 511 GL1 POP 118 22.300 4.360 78.530 1.00 0.00

ATOM 512 GL2 POP 118 21.490 7.290 77.260 1.00 0.00

ATOM 513 C1A POP 118 19.520 6.840 74.120 1.00 0.00

ATOM 514 C2A POP 118 17.030 9.430 71.760 1.00 0.00

ATOM 515 C3A POP 118 16.840 11.420 68.270 1.00 0.00

ATOM 516 C4A POP 118 17.700 14.680 64.230 1.00 0.00

ATOM 517 C1B POP 118 19.980 2.160 76.010 1.00 0.00

ATOM 518 C2B POP 118 16.270 -0.910 73.750 1.00 0.00

ATOM 519 D3B POP 118 14.800 -4.990 71.920 1.00 0.00

ATOM 520 C4B POP 118 16.350 -8.420 68.270 1.00 0.00

ATOM 521 C5B POP 118 17.520 -11.020 65.640 1.00 0.00

ATOM 522 NC3 POP 119 75.530 71.050 44.280 1.00 0.00

ATOM 523 PO4 POP 119 72.360 68.840 46.690 1.00 0.00

ATOM 524 GL1 POP 119 69.620 69.880 50.060 1.00 0.00

ATOM 525 GL2 POP 119 69.500 72.460 51.320 1.00 0.00

ATOM 526 C1A POP 119 66.800 73.590 54.070 1.00 0.00

ATOM 527 C2A POP 119 66.250 76.170 58.310 1.00 0.00

ATOM 528 C3A POP 119 66.530 77.480 62.470 1.00 0.00

ATOM 529 C4A POP 119 68.020 75.820 65.730 1.00 0.00

ATOM 530 C1B POP 119 66.670 67.670 53.510 1.00 0.00

ATOM 531 C2B POP 119 66.930 66.130 57.340 1.00 0.00

ATOM 532 D3B POP 119 66.770 62.220 58.880 1.00 0.00

ATOM 533 C4B POP 119 62.590 60.640 60.610 1.00 0.00

ATOM 534 C5B POP 119 60.070 58.170 58.650 1.00 0.00

ATOM 535 NC3 POP 120 96.110 62.450 39.830 1.00 0.00

ATOM 536 PO4 POP 120 95.080 59.430 44.100 1.00 0.00

ATOM 537 GL1 POP 120 93.830 58.960 49.070 1.00 0.00

ATOM 538 GL2 POP 120 91.380 58.140 51.170 1.00 0.00

ATOM 539 C1A POP 120 92.340 54.060 54.480 1.00 0.00

ATOM 540 C2A POP 120 95.330 53.590 57.800 1.00 0.00

ATOM 541 C3A POP 120 97.230 53.930 62.750 1.00 0.00

ATOM 542 C4A POP 120 101.000 52.810 65.050 1.00 0.00

ATOM 543 C1B POP 120 95.830 56.910 52.570 1.00 0.00

ATOM 544 C2B POP 120 98.690 57.360 55.120 1.00 0.00

ATOM 545 D3B POP 120 99.680 61.520 56.470 1.00 0.00

ATOM 546 C4B POP 120 101.350 61.530 59.800 1.00 0.00

ATOM 547 C5B POP 120 100.310 63.060 64.120 1.00 0.00

ATOM 548 NC3 POP 121 94.320 89.550 47.440 1.00 0.00

ATOM 549 PO4 POP 121 97.340 85.990 47.780 1.00 0.00

ATOM 550 GL1 POP 121 98.200 88.730 50.670 1.00 0.00

ATOM 551 GL2 POP 121 95.040 88.890 52.720 1.00 0.00

ATOM 552 C1A POP 121 93.650 87.450 56.700 1.00 0.00

ATOM 553 C2A POP 121 94.310 89.440 61.350 1.00 0.00

ATOM 554 C3A POP 121 95.630 92.800 63.560 1.00 0.00

ATOM 555 C4A POP 121 93.170 94.700 66.610 1.00 0.00

ATOM 556 C1B POP 121 100.450 90.210 54.560 1.00 0.00

ATOM 557 C2B POP 121 98.870 89.790 59.210 1.00 0.00

ATOM 558 D3B POP 121 100.370 92.140 62.210 1.00 0.00

ATOM 559 C4B POP 121 104.020 90.590 64.700 1.00 0.00

ATOM 560 C5B POP 121 107.550 89.860 66.910 1.00 0.00

ATOM 561 NC3 POP 122 70.750 89.250 87.690 1.00 0.00

ATOM 562 PO4 POP 122 72.860 93.280 86.390 1.00 0.00

ATOM 563 GL1 POP 122 72.560 93.970 82.260 1.00 0.00

ATOM 564 GL2 POP 122 74.530 97.100 81.340 1.00 0.00

ATOM 565 C1A POP 122 78.150 96.280 79.060 1.00 0.00

ATOM 566 C2A POP 122 78.680 97.190 75.410 1.00 0.00

ATOM 567 C3A POP 122 81.580 94.560 72.060 1.00 0.00

ATOM 568 C4A POP 122 83.320 94.020 67.690 1.00 0.00

ATOM 569 C1B POP 122 74.970 91.400 78.810 1.00 0.00

ATOM 570 C2B POP 122 75.750 92.610 74.670 1.00 0.00

ATOM 571 D3B POP 122 76.620 94.010 70.660 1.00 0.00

ATOM 572 C4B POP 122 76.060 98.900 70.320 1.00 0.00

ATOM 573 C5B POP 122 76.950 101.930 73.090 1.00 0.00

ATOM 574 NC3 POP 123 29.970 87.300 89.890 1.00 0.00

ATOM 575 PO4 POP 123 33.970 87.130 88.320 1.00 0.00

ATOM 576 GL1 POP 123 32.280 89.470 85.000 1.00 0.00

ATOM 577 GL2 POP 123 28.160 89.680 84.040 1.00 0.00

ATOM 578 C1A POP 123 30.350 88.030 80.960 1.00 0.00

ATOM 579 C2A POP 123 34.800 87.250 80.570 1.00 0.00

ATOM 580 C3A POP 123 39.500 86.870 80.450 1.00 0.00

ATOM 581 C4A POP 123 43.360 89.630 81.640 1.00 0.00

ATOM 582 C1B POP 123 33.840 92.390 81.730 1.00 0.00

ATOM 583 C2B POP 123 33.970 96.230 79.300 1.00 0.00

ATOM 584 D3B POP 123 32.140 96.510 75.220 1.00 0.00

ATOM 585 C4B POP 123 28.100 94.080 74.150 1.00 0.00

ATOM 586 C5B POP 123 25.590 92.120 73.920 1.00 0.00

ATOM 587 NC3 POP 124 90.300 35.760 87.360 1.00 0.00

ATOM 588 PO4 POP 124 88.550 31.500 87.690 1.00 0.00

ATOM 589 GL1 POP 124 89.000 30.510 83.490 1.00 0.00

ATOM 590 GL2 POP 124 88.810 28.870 82.240 1.00 0.00

ATOM 591 C1A POP 124 85.730 27.530 80.190 1.00 0.00

ATOM 592 C2A POP 124 82.060 28.030 79.860 1.00 0.00

ATOM 593 C3A POP 124 78.190 29.820 76.240 1.00 0.00

ATOM 594 C4A POP 124 74.010 28.190 74.030 1.00 0.00

ATOM 595 C1B POP 124 92.010 32.690 80.720 1.00 0.00

ATOM 596 C2B POP 124 91.050 35.720 76.860 1.00 0.00

ATOM 597 D3B POP 124 88.800 36.920 73.250 1.00 0.00

ATOM 598 C4B POP 124 91.490 36.070 70.440 1.00 0.00

ATOM 599 C5B POP 124 94.690 36.210 67.720 1.00 0.00

ATOM 600 NC3 POP 125 103.410 93.860 40.670 1.00 0.00

ATOM 601 PO4 POP 125 102.410 94.810 44.880 1.00 0.00

ATOM 602 GL1 POP 125 101.680 97.000 48.180 1.00 0.00

ATOM 603 GL2 POP 125 100.880 99.530 47.030 1.00 0.00

ATOM 604 C1A POP 125 102.930 99.630 52.020 1.00 0.00

ATOM 605 C2A POP 125 104.510 102.830 55.890 1.00 0.00

ATOM 606 C3A POP 125 106.620 105.600 59.090 1.00 0.00

ATOM 607 C4A POP 125 110.930 106.630 60.900 1.00 0.00

ATOM 608 C1B POP 125 101.380 94.970 52.140 1.00 0.00

ATOM 609 C2B POP 125 102.410 94.380 56.270 1.00 0.00

ATOM 610 D3B POP 125 105.160 94.610 59.530 1.00 0.00

ATOM 611 C4B POP 125 107.710 93.130 62.210 1.00 0.00

ATOM 612 C5B POP 125 111.650 91.400 60.920 1.00 0.00

ATOM 613 NC3 POP 126 21.300 15.350 84.130 1.00 0.00

ATOM 614 PO4 POP 126 17.900 11.810 84.530 1.00 0.00

ATOM 615 GL1 POP 126 16.950 10.200 80.060 1.00 0.00

ATOM 616 GL2 POP 126 16.710 6.930 78.730 1.00 0.00

ATOM 617 C1A POP 126 14.870 2.780 76.560 1.00 0.00

ATOM 618 C2A POP 126 12.080 -0.630 77.540 1.00 0.00

ATOM 619 C3A POP 126 10.680 -4.910 75.590 1.00 0.00

ATOM 620 C4A POP 126 8.880 -8.540 77.390 1.00 0.00

ATOM 621 C1B POP 126 18.610 11.000 76.100 1.00 0.00

ATOM 622 C2B POP 126 21.060 12.040 72.160 1.00 0.00

ATOM 623 D3B POP 126 21.670 11.070 67.200 1.00 0.00

ATOM 624 C4B POP 126 23.440 9.860 62.060 1.00 0.00

ATOM 625 C5B POP 126 26.220 10.530 57.890 1.00 0.00

ATOM 626 NC3 POP 127 65.670 44.500 91.120 1.00 0.00

ATOM 627 PO4 POP 127 67.700 45.210 87.760 1.00 0.00

ATOM 628 GL1 POP 127 65.240 47.120 85.240 1.00 0.00

ATOM 629 GL2 POP 127 62.220 46.540 84.890 1.00 0.00

ATOM 630 C1A POP 127 59.280 47.050 80.900 1.00 0.00

ATOM 631 C2A POP 127 59.870 48.370 76.230 1.00 0.00

ATOM 632 C3A POP 127 63.150 48.060 73.280 1.00 0.00

ATOM 633 C4A POP 127 67.020 49.490 71.710 1.00 0.00

ATOM 634 C1B POP 127 63.860 50.420 82.220 1.00 0.00

ATOM 635 C2B POP 127 60.890 52.450 80.740 1.00 0.00

ATOM 636 D3B POP 127 56.240 51.120 80.490 1.00 0.00

ATOM 637 C4B POP 127 52.740 54.060 81.040 1.00 0.00

ATOM 638 C5B POP 127 50.720 57.590 82.650 1.00 0.00

ATOM 639 NC3 POP 128 86.110 56.910 94.900 1.00 0.00

ATOM 640 PO4 POP 128 89.730 54.410 93.290 1.00 0.00

ATOM 641 GL1 POP 128 88.350 55.990 89.240 1.00 0.00

ATOM 642 GL2 POP 128 90.220 58.810 87.880 1.00 0.00

ATOM 643 C1A POP 128 87.420 58.520 84.050 1.00 0.00

ATOM 644 C2A POP 128 86.420 55.980 80.500 1.00 0.00

ATOM 645 C3A POP 128 83.790 55.530 77.010 1.00 0.00

ATOM 646 C4A POP 128 85.310 51.450 75.520 1.00 0.00

ATOM 647 C1B POP 128 90.620 54.550 85.730 1.00 0.00

ATOM 648 C2B POP 128 90.440 53.610 81.640 1.00 0.00

ATOM 649 D3B POP 128 89.810 53.210 77.450 1.00 0.00

ATOM 650 C4B POP 128 88.240 56.550 74.660 1.00 0.00

ATOM 651 C5B POP 128 85.470 56.500 70.710 1.00 0.00

ATOM 652 NC3 POP 129 28.730 26.320 86.190 1.00 0.00

ATOM 653 PO4 POP 129 28.450 23.350 83.910 1.00 0.00

ATOM 654 GL1 POP 129 30.120 23.170 79.450 1.00 0.00

ATOM 655 GL2 POP 129 32.880 26.140 77.270 1.00 0.00

ATOM 656 C1A POP 129 31.390 28.010 73.440 1.00 0.00

ATOM 657 C2A POP 129 32.440 24.970 69.690 1.00 0.00

ATOM 658 C3A POP 129 33.020 26.220 65.710 1.00 0.00

ATOM 659 C4A POP 129 34.600 24.790 62.560 1.00 0.00

ATOM 660 C1B POP 129 27.680 26.110 76.660 1.00 0.00

ATOM 661 C2B POP 129 26.350 26.390 73.180 1.00 0.00

ATOM 662 D3B POP 129 25.790 25.060 69.000 1.00 0.00

ATOM 663 C4B POP 129 22.830 26.080 66.080 1.00 0.00

ATOM 664 C5B POP 129 22.780 26.300 61.100 1.00 0.00

ATOM 665 NC3 POP 130 74.130 62.890 84.990 1.00 0.00

ATOM 666 PO4 POP 130 77.170 64.360 83.730 1.00 0.00

ATOM 667 GL1 POP 130 75.620 65.040 80.150 1.00 0.00

ATOM 668 GL2 POP 130 73.630 62.620 80.460 1.00 0.00

ATOM 669 C1A POP 130 73.940 61.510 75.990 1.00 0.00

ATOM 670 C2A POP 130 72.470 60.610 71.490 1.00 0.00

ATOM 671 C3A POP 130 67.350 57.220 69.220 1.00 0.00

ATOM 672 C4A POP 130 66.270 53.510 66.710 1.00 0.00

ATOM 673 C1B POP 130 73.500 66.780 76.800 1.00 0.00

ATOM 674 C2B POP 130 71.710 65.650 72.430 1.00 0.00

ATOM 675 D3B POP 130 70.680 64.770 68.150 1.00 0.00

ATOM 676 C4B POP 130 66.980 66.830 66.050 1.00 0.00

ATOM 677 C5B POP 130 68.680 67.770 61.540 1.00 0.00

ATOM 678 NC3 POP 131 95.440 106.670 88.450 1.00 0.00

ATOM 679 PO4 POP 131 92.360 108.490 89.000 1.00 0.00

ATOM 680 GL1 POP 131 92.300 108.530 84.650 1.00 0.00

ATOM 681 GL2 POP 131 94.870 108.600 82.740 1.00 0.00

ATOM 682 C1A POP 131 96.550 110.970 79.070 1.00 0.00

ATOM 683 C2A POP 131 98.120 110.630 75.180 1.00 0.00

ATOM 684 C3A POP 131 99.320 110.900 71.590 1.00 0.00

ATOM 685 C4A POP 131 98.480 114.470 70.210 1.00 0.00

ATOM 686 C1B POP 131 90.200 109.690 81.420 1.00 0.00

ATOM 687 C2B POP 131 90.900 109.540 77.290 1.00 0.00

ATOM 688 D3B POP 131 91.930 108.460 72.700 1.00 0.00

ATOM 689 C4B POP 131 96.060 107.260 70.870 1.00 0.00

ATOM 690 C5B POP 131 98.730 105.370 68.460 1.00 0.00

ATOM 691 NC3 POP 132 41.650 91.040 88.720 1.00 0.00

ATOM 692 PO4 POP 132 45.060 93.060 88.020 1.00 0.00

ATOM 693 GL1 POP 132 44.830 94.880 84.380 1.00 0.00

ATOM 694 GL2 POP 132 45.850 98.980 83.770 1.00 0.00

ATOM 695 C1A POP 132 48.290 97.200 80.360 1.00 0.00

ATOM 696 C2A POP 132 51.700 98.550 75.870 1.00 0.00

ATOM 697 C3A POP 132 53.930 96.720 74.230 1.00 0.00

ATOM 698 C4A POP 132 56.390 94.740 71.350 1.00 0.00

ATOM 699 C1B POP 132 46.890 92.430 79.990 1.00 0.00

ATOM 700 C2B POP 132 50.300 88.310 78.430 1.00 0.00

ATOM 701 D3B POP 132 52.850 85.610 75.640 1.00 0.00

ATOM 702 C4B POP 132 56.750 85.220 76.860 1.00 0.00

ATOM 703 C5B POP 132 59.020 81.690 76.760 1.00 0.00

ATOM 704 NC3 POP 133 78.220 100.200 91.370 1.00 0.00

ATOM 705 PO4 POP 133 74.800 101.050 87.990 1.00 0.00

ATOM 706 GL1 POP 133 76.780 101.240 83.810 1.00 0.00

ATOM 707 GL2 POP 133 74.470 102.070 82.640 1.00 0.00

ATOM 708 C1A POP 133 71.110 100.400 79.340 1.00 0.00

ATOM 709 C2A POP 133 67.960 98.780 75.890 1.00 0.00

ATOM 710 C3A POP 133 66.110 96.660 73.430 1.00 0.00

ATOM 711 C4A POP 133 66.410 97.720 69.240 1.00 0.00

ATOM 712 C1B POP 133 80.430 99.260 81.920 1.00 0.00

ATOM 713 C2B POP 133 84.540 100.490 80.930 1.00 0.00

ATOM 714 D3B POP 133 88.710 100.340 82.330 1.00 0.00

ATOM 715 C4B POP 133 91.800 104.370 81.490 1.00 0.00

ATOM 716 C5B POP 133 94.720 106.280 77.810 1.00 0.00

ATOM 717 NC3 POP 134 91.030 71.960 48.440 1.00 0.00

ATOM 718 PO4 POP 134 90.770 75.870 51.100 1.00 0.00

ATOM 719 GL1 POP 134 92.440 73.070 54.170 1.00 0.00

ATOM 720 GL2 POP 134 95.400 73.430 53.870 1.00 0.00

ATOM 721 C1A POP 134 96.660 74.710 56.950 1.00 0.00

ATOM 722 C2A POP 134 98.570 73.020 60.140 1.00 0.00

ATOM 723 C3A POP 134 101.780 71.730 62.910 1.00 0.00

ATOM 724 C4A POP 134 104.050 67.550 62.760 1.00 0.00

ATOM 725 C1B POP 134 93.340 70.660 57.890 1.00 0.00

ATOM 726 C2B POP 134 94.710 71.040 62.570 1.00 0.00

ATOM 727 D3B POP 134 93.740 69.980 65.810 1.00 0.00

ATOM 728 C4B POP 134 91.140 65.680 66.690 1.00 0.00

ATOM 729 C5B POP 134 88.670 65.200 69.900 1.00 0.00

ATOM 730 NC3 POP 135 91.430 49.850 40.590 1.00 0.00

ATOM 731 PO4 POP 135 91.090 49.150 46.050 1.00 0.00

ATOM 732 GL1 POP 135 90.840 49.750 50.510 1.00 0.00

ATOM 733 GL2 POP 135 92.290 46.510 51.220 1.00 0.00

ATOM 734 C1A POP 135 94.450 43.470 54.010 1.00 0.00

ATOM 735 C2A POP 135 95.490 39.330 54.660 1.00 0.00

ATOM 736 C3A POP 135 94.430 34.870 53.050 1.00 0.00

ATOM 737 C4A POP 135 91.830 30.980 52.670 1.00 0.00

ATOM 738 C1B POP 135 90.110 50.040 55.460 1.00 0.00

ATOM 739 C2B POP 135 90.520 50.760 58.730 1.00 0.00

ATOM 740 D3B POP 135 93.390 50.760 62.560 1.00 0.00

ATOM 741 C4B POP 135 91.600 50.590 67.100 1.00 0.00

ATOM 742 C5B POP 135 91.800 47.690 69.830 1.00 0.00

ATOM 743 NC3 POP 136 24.010 62.550 93.640 1.00 0.00

ATOM 744 PO4 POP 136 20.910 60.830 93.070 1.00 0.00

ATOM 745 GL1 POP 136 20.240 61.480 88.900 1.00 0.00

ATOM 746 GL2 POP 136 20.650 58.470 87.640 1.00 0.00

ATOM 747 C1A POP 136 20.930 54.540 85.200 1.00 0.00

ATOM 748 C2A POP 136 21.070 49.780 83.370 1.00 0.00

ATOM 749 C3A POP 136 22.560 49.550 78.990 1.00 0.00

ATOM 750 C4A POP 136 23.470 47.850 74.850 1.00 0.00

ATOM 751 C1B POP 136 19.860 62.060 84.050 1.00 0.00

ATOM 752 C2B POP 136 21.080 58.800 81.510 1.00 0.00

ATOM 753 D3B POP 136 20.370 56.900 78.030 1.00 0.00

ATOM 754 C4B POP 136 20.440 58.530 74.000 1.00 0.00

ATOM 755 C5B POP 136 22.770 60.370 70.970 1.00 0.00

ATOM 756 NC3 POP 137 6.720 30.150 41.610 1.00 0.00

ATOM 757 PO4 POP 137 7.200 32.620 45.030 1.00 0.00

ATOM 758 GL1 POP 137 7.020 30.240 48.590 1.00 0.00

ATOM 759 GL2 POP 137 9.050 32.920 50.800 1.00 0.00

ATOM 760 C1A POP 137 7.810 30.650 54.510 1.00 0.00

ATOM 761 C2A POP 137 5.940 30.600 58.580 1.00 0.00

ATOM 762 C3A POP 137 3.090 28.410 60.760 1.00 0.00

ATOM 763 C4A POP 137 3.610 25.740 64.940 1.00 0.00

ATOM 764 C1B POP 137 5.610 27.770 50.990 1.00 0.00

ATOM 765 C2B POP 137 5.740 23.940 51.980 1.00 0.00

ATOM 766 D3B POP 137 7.630 21.720 56.100 1.00 0.00

ATOM 767 C4B POP 137 5.420 17.460 56.450 1.00 0.00

ATOM 768 C5B POP 137 5.050 13.340 55.270 1.00 0.00

ATOM 769 NC3 POP 138 79.230 19.040 41.770 1.00 0.00

ATOM 770 PO4 POP 138 78.600 18.400 45.910 1.00 0.00

ATOM 771 GL1 POP 138 80.070 19.040 49.390 1.00 0.00

ATOM 772 GL2 POP 138 79.370 20.470 52.110 1.00 0.00

ATOM 773 C1A POP 138 81.220 20.160 57.110 1.00 0.00

ATOM 774 C2A POP 138 82.730 23.190 59.250 1.00 0.00

ATOM 775 C3A POP 138 81.550 25.900 62.140 1.00 0.00

ATOM 776 C4A POP 138 82.310 28.440 66.840 1.00 0.00

ATOM 777 C1B POP 138 83.650 18.680 52.620 1.00 0.00

ATOM 778 C2B POP 138 86.380 20.610 55.710 1.00 0.00

ATOM 779 D3B POP 138 87.730 18.760 59.410 1.00 0.00

ATOM 780 C4B POP 138 86.930 20.910 63.080 1.00 0.00

ATOM 781 C5B POP 138 85.750 19.320 66.660 1.00 0.00

ATOM 782 NC3 POP 139 51.960 69.240 46.170 1.00 0.00

ATOM 783 PO4 POP 139 55.600 72.640 45.240 1.00 0.00

ATOM 784 GL1 POP 139 56.980 70.940 48.970 1.00 0.00

ATOM 785 GL2 POP 139 59.430 72.540 48.780 1.00 0.00

ATOM 786 C1A POP 139 62.070 72.970 52.140 1.00 0.00

ATOM 787 C2A POP 139 62.370 72.440 56.310 1.00 0.00

ATOM 788 C3A POP 139 64.930 71.640 59.540 1.00 0.00

ATOM 789 C4A POP 139 64.590 71.890 64.940 1.00 0.00

ATOM 790 C1B POP 139 58.270 70.480 53.490 1.00 0.00

ATOM 791 C2B POP 139 61.010 67.720 56.500 1.00 0.00

ATOM 792 D3B POP 139 63.520 67.220 60.460 1.00 0.00

ATOM 793 C4B POP 139 61.690 68.690 63.270 1.00 0.00

ATOM 794 C5B POP 139 58.470 69.940 66.370 1.00 0.00

ATOM 795 NC3 POP 140 75.220 6.430 42.680 1.00 0.00

ATOM 796 PO4 POP 140 72.140 10.740 43.310 1.00 0.00

ATOM 797 GL1 POP 140 73.000 8.870 46.780 1.00 0.00

ATOM 798 GL2 POP 140 71.550 11.180 47.870 1.00 0.00

ATOM 799 C1A POP 140 73.530 11.810 52.760 1.00 0.00

ATOM 800 C2A POP 140 75.210 12.410 57.460 1.00 0.00

ATOM 801 C3A POP 140 76.830 11.000 62.120 1.00 0.00

ATOM 802 C4A POP 140 78.000 8.470 65.890 1.00 0.00

ATOM 803 C1B POP 140 73.540 6.910 51.200 1.00 0.00

ATOM 804 C2B POP 140 75.560 7.260 54.240 1.00 0.00

ATOM 805 D3B POP 140 74.210 7.960 58.410 1.00 0.00

ATOM 806 C4B POP 140 75.230 5.820 62.430 1.00 0.00

ATOM 807 C5B POP 140 78.820 3.510 62.020 1.00 0.00

ATOM 808 NC3 POP 141 87.380 25.030 44.950 1.00 0.00

ATOM 809 PO4 POP 141 83.590 27.060 46.930 1.00 0.00

ATOM 810 GL1 POP 141 84.890 24.260 49.190 1.00 0.00

ATOM 811 GL2 POP 141 86.620 21.940 48.990 1.00 0.00

ATOM 812 C1A POP 141 90.160 20.310 51.790 1.00 0.00

ATOM 813 C2A POP 141 92.270 17.200 53.560 1.00 0.00

ATOM 814 C3A POP 141 92.320 17.060 57.640 1.00 0.00

ATOM 815 C4A POP 141 93.100 14.070 60.310 1.00 0.00

ATOM 816 C1B POP 141 85.320 25.660 54.100 1.00 0.00

ATOM 817 C2B POP 141 87.930 24.230 58.330 1.00 0.00

ATOM 818 D3B POP 141 86.100 26.320 61.730 1.00 0.00

ATOM 819 C4B POP 141 89.440 28.970 61.670 1.00 0.00

ATOM 820 C5B POP 141 93.170 30.410 61.130 1.00 0.00

ATOM 821 NC3 POP 142 95.960 43.310 90.830 1.00 0.00

ATOM 822 PO4 POP 142 93.530 45.840 89.590 1.00 0.00

ATOM 823 GL1 POP 142 94.890 48.790 86.650 1.00 0.00

ATOM 824 GL2 POP 142 95.190 52.300 86.200 1.00 0.00

ATOM 825 C1A POP 142 98.920 54.620 82.520 1.00 0.00

ATOM 826 C2A POP 142 99.360 57.220 79.720 1.00 0.00

ATOM 827 C3A POP 142 96.010 59.480 79.330 1.00 0.00

ATOM 828 C4A POP 142 95.140 63.190 77.140 1.00 0.00

ATOM 829 C1B POP 142 93.490 50.120 82.200 1.00 0.00

ATOM 830 C2B POP 142 93.990 49.090 78.600 1.00 0.00

ATOM 831 D3B POP 142 90.620 48.500 74.830 1.00 0.00

ATOM 832 C4B POP 142 94.020 51.220 72.770 1.00 0.00

ATOM 833 C5B POP 142 97.860 51.910 69.290 1.00 0.00

ATOM 834 NC3 POP 143 18.690 5.910 40.500 1.00 0.00

ATOM 835 PO4 POP 143 22.490 2.510 41.620 1.00 0.00

ATOM 836 GL1 POP 143 21.670 3.640 47.550 1.00 0.00

ATOM 837 GL2 POP 143 23.350 1.160 49.360 1.00 0.00

ATOM 838 C1A POP 143 24.790 1.200 53.740 1.00 0.00

ATOM 839 C2A POP 143 25.100 1.090 57.580 1.00 0.00

ATOM 840 C3A POP 143 27.050 -0.730 60.760 1.00 0.00

ATOM 841 C4A POP 143 29.360 -4.050 60.100 1.00 0.00

ATOM 842 C1B POP 143 21.680 6.600 50.110 1.00 0.00

ATOM 843 C2B POP 143 21.550 11.240 49.330 1.00 0.00

ATOM 844 D3B POP 143 21.420 15.940 50.400 1.00 0.00

ATOM 845 C4B POP 143 20.340 17.530 55.130 1.00 0.00

ATOM 846 C5B POP 143 20.350 15.380 59.030 1.00 0.00

ATOM 847 NC3 POP 144 46.020 2.340 42.060 1.00 0.00

ATOM 848 PO4 POP 144 47.830 4.720 44.910 1.00 0.00

ATOM 849 GL1 POP 144 46.600 3.940 50.110 1.00 0.00

ATOM 850 GL2 POP 144 48.780 1.150 51.830 1.00 0.00

ATOM 851 C1A POP 144 47.140 -1.060 54.780 1.00 0.00

ATOM 852 C2A POP 144 47.650 -0.450 59.230 1.00 0.00

ATOM 853 C3A POP 144 49.230 3.310 61.790 1.00 0.00

ATOM 854 C4A POP 144 53.840 3.830 63.610 1.00 0.00

ATOM 855 C1B POP 144 44.760 2.970 53.990 1.00 0.00

ATOM 856 C2B POP 144 44.940 4.380 58.410 1.00 0.00

ATOM 857 D3B POP 144 44.350 2.150 62.510 1.00 0.00

ATOM 858 C4B POP 144 43.190 -1.240 61.650 1.00 0.00

ATOM 859 C5B POP 144 42.610 -4.020 64.120 1.00 0.00

ATOM 860 NC3 POP 145 48.320 49.180 41.790 1.00 0.00

ATOM 861 PO4 POP 145 50.140 51.160 45.050 1.00 0.00

ATOM 862 GL1 POP 145 49.440 50.370 49.770 1.00 0.00

ATOM 863 GL2 POP 145 46.210 50.440 51.200 1.00 0.00

ATOM 864 C1A POP 145 45.340 48.820 53.350 1.00 0.00

ATOM 865 C2A POP 145 45.200 45.550 54.750 1.00 0.00

ATOM 866 C3A POP 145 46.710 45.100 59.590 1.00 0.00

ATOM 867 C4A POP 145 48.880 48.690 62.490 1.00 0.00

ATOM 868 C1B POP 145 49.770 53.200 53.780 1.00 0.00

ATOM 869 C2B POP 145 49.940 55.640 57.750 1.00 0.00

ATOM 870 D3B POP 145 48.540 58.540 61.330 1.00 0.00

ATOM 871 C4B POP 145 49.390 56.730 65.340 1.00 0.00

ATOM 872 C5B POP 145 50.760 54.910 69.310 1.00 0.00

ATOM 873 NC3 POP 146 107.660 33.710 46.950 1.00 0.00

ATOM 874 PO4 POP 146 111.300 31.730 45.320 1.00 0.00

ATOM 875 GL1 POP 146 110.010 29.440 49.550 1.00 0.00

ATOM 876 GL2 POP 146 110.170 26.210 49.390 1.00 0.00

ATOM 877 C1A POP 146 110.930 25.150 53.870 1.00 0.00

ATOM 878 C2A POP 146 114.480 25.770 56.500 1.00 0.00

ATOM 879 C3A POP 146 114.660 23.680 60.410 1.00 0.00

ATOM 880 C4A POP 146 116.730 19.720 60.340 1.00 0.00

ATOM 881 C1B POP 146 106.620 28.600 52.190 1.00 0.00

ATOM 882 C2B POP 146 102.700 27.330 54.120 1.00 0.00

ATOM 883 D3B POP 146 99.890 24.200 53.750 1.00 0.00

ATOM 884 C4B POP 146 97.630 24.710 57.710 1.00 0.00

ATOM 885 C5B POP 146 98.630 28.490 60.460 1.00 0.00

ATOM 886 NC3 POP 147 64.710 30.710 39.990 1.00 0.00

ATOM 887 PO4 POP 147 65.340 34.470 40.610 1.00 0.00

ATOM 888 GL1 POP 147 64.940 32.970 44.340 1.00 0.00

ATOM 889 GL2 POP 147 62.400 33.420 45.830 1.00 0.00

ATOM 890 C1A POP 147 62.420 30.260 49.740 1.00 0.00

ATOM 891 C2A POP 147 65.300 30.300 53.010 1.00 0.00

ATOM 892 C3A POP 147 68.110 29.120 56.580 1.00 0.00

ATOM 893 C4A POP 147 69.820 24.950 58.320 1.00 0.00

ATOM 894 C1B POP 147 66.850 33.450 48.150 1.00 0.00

ATOM 895 C2B POP 147 70.020 31.680 51.220 1.00 0.00

ATOM 896 D3B POP 147 72.920 32.840 54.840 1.00 0.00

ATOM 897 C4B POP 147 72.950 30.790 58.880 1.00 0.00

ATOM 898 C5B POP 147 74.130 30.750 64.200 1.00 0.00

ATOM 899 NC3 POP 148 2.700 106.670 42.740 1.00 0.00

ATOM 900 PO4 POP 148 1.390 110.670 42.600 1.00 0.00

ATOM 901 GL1 POP 148 1.660 109.800 46.780 1.00 0.00

ATOM 902 GL2 POP 148 5.060 108.870 49.080 1.00 0.00

ATOM 903 C1A POP 148 5.690 107.930 53.870 1.00 0.00

ATOM 904 C2A POP 148 6.200 106.740 57.930 1.00 0.00

ATOM 905 C3A POP 148 5.570 103.880 61.560 1.00 0.00

ATOM 906 C4A POP 148 7.480 102.750 66.050 1.00 0.00

ATOM 907 C1B POP 148 0.230 109.890 51.590 1.00 0.00

ATOM 908 C2B POP 148 0.680 106.440 54.670 1.00 0.00

ATOM 909 D3B POP 148 1.760 101.870 55.470 1.00 0.00

ATOM 910 C4B POP 148 4.480 100.900 58.180 1.00 0.00

ATOM 911 C5B POP 148 8.110 99.380 57.050 1.00 0.00

ATOM 912 NC3 POP 149 54.460 19.860 45.390 1.00 0.00

ATOM 913 PO4 POP 149 58.240 20.540 46.160 1.00 0.00

ATOM 914 GL1 POP 149 57.130 21.450 50.660 1.00 0.00

ATOM 915 GL2 POP 149 55.520 18.920 54.020 1.00 0.00

ATOM 916 C1A POP 149 52.720 20.940 56.260 1.00 0.00

ATOM 917 C2A POP 149 52.250 24.240 57.820 1.00 0.00

ATOM 918 C3A POP 149 55.610 26.090 55.910 1.00 0.00

ATOM 919 C4A POP 149 60.280 27.780 53.430 1.00 0.00

ATOM 920 C1B POP 149 59.130 22.510 53.640 1.00 0.00

ATOM 921 C2B POP 149 59.290 25.060 58.430 1.00 0.00

ATOM 922 D3B POP 149 56.130 25.910 61.880 1.00 0.00

ATOM 923 C4B POP 149 54.270 27.770 65.180 1.00 0.00

ATOM 924 C5B POP 149 52.100 27.220 68.330 1.00 0.00

ATOM 925 NC3 POP 150 35.620 92.260 43.210 1.00 0.00

ATOM 926 PO4 POP 150 31.510 93.390 45.060 1.00 0.00

ATOM 927 GL1 POP 150 29.040 92.880 48.300 1.00 0.00

ATOM 928 GL2 POP 150 27.520 95.900 48.960 1.00 0.00

ATOM 929 C1A POP 150 24.550 95.000 52.310 1.00 0.00

ATOM 930 C2A POP 150 24.370 96.920 55.530 1.00 0.00

ATOM 931 C3A POP 150 23.850 99.100 60.190 1.00 0.00

ATOM 932 C4A POP 150 23.040 98.760 64.360 1.00 0.00

ATOM 933 C1B POP 150 29.110 92.410 52.890 1.00 0.00

ATOM 934 C2B POP 150 29.120 94.150 57.530 1.00 0.00

ATOM 935 D3B POP 150 27.320 95.320 60.650 1.00 0.00

ATOM 936 C4B POP 150 26.760 95.870 65.200 1.00 0.00

ATOM 937 C5B POP 150 25.790 95.950 69.880 1.00 0.00

ATOM 938 NC3 POP 151 84.760 41.430 44.480 1.00 0.00

ATOM 939 PO4 POP 151 87.500 39.900 46.890 1.00 0.00

ATOM 940 GL1 POP 151 87.460 37.180 49.540 1.00 0.00

ATOM 941 GL2 POP 151 90.420 38.140 50.680 1.00 0.00

ATOM 942 C1A POP 151 90.380 40.230 55.300 1.00 0.00

ATOM 943 C2A POP 151 88.600 39.180 59.150 1.00 0.00

ATOM 944 C3A POP 151 88.910 40.570 63.190 1.00 0.00

ATOM 945 C4A POP 151 85.400 42.910 64.130 1.00 0.00

ATOM 946 C1B POP 151 86.990 36.360 53.940 1.00 0.00

ATOM 947 C2B POP 151 84.490 35.020 56.500 1.00 0.00

ATOM 948 D3B POP 151 80.900 36.100 58.490 1.00 0.00

ATOM 949 C4B POP 151 79.500 36.390 62.880 1.00 0.00

ATOM 950 C5B POP 151 81.990 39.300 66.110 1.00 0.00

ATOM 951 NC3 POP 152 30.910 58.450 94.070 1.00 0.00

ATOM 952 PO4 POP 152 28.930 62.000 91.600 1.00 0.00

ATOM 953 GL1 POP 152 29.210 62.670 86.670 1.00 0.00

ATOM 954 GL2 POP 152 30.640 59.830 84.440 1.00 0.00

ATOM 955 C1A POP 152 28.320 59.830 80.850 1.00 0.00

ATOM 956 C2A POP 152 25.110 58.300 77.570 1.00 0.00

ATOM 957 C3A POP 152 27.170 58.510 73.370 1.00 0.00

ATOM 958 C4A POP 152 27.560 61.270 69.830 1.00 0.00

ATOM 959 C1B POP 152 28.870 64.310 83.160 1.00 0.00

ATOM 960 C2B POP 152 28.450 67.230 80.090 1.00 0.00

ATOM 961 D3B POP 152 30.680 68.780 77.000 1.00 0.00

ATOM 962 C4B POP 152 32.630 68.600 72.070 1.00 0.00

ATOM 963 C5B POP 152 35.650 66.800 69.010 1.00 0.00

ATOM 964 NC3 POP 153 100.660 25.600 86.270 1.00 0.00

ATOM 965 PO4 POP 153 96.260 25.470 85.590 1.00 0.00

ATOM 966 GL1 POP 153 93.880 27.870 82.330 1.00 0.00

ATOM 967 GL2 POP 153 95.960 27.160 79.940 1.00 0.00

ATOM 968 C1A POP 153 98.600 29.270 77.350 1.00 0.00

ATOM 969 C2A POP 153 101.870 28.570 74.570 1.00 0.00

ATOM 970 C3A POP 153 105.480 32.200 74.980 1.00 0.00

ATOM 971 C4A POP 153 107.150 35.660 73.220 1.00 0.00

ATOM 972 C1B POP 153 91.210 27.830 78.270 1.00 0.00

ATOM 973 C2B POP 153 92.430 29.570 74.300 1.00 0.00

ATOM 974 D3B POP 153 96.080 27.590 72.810 1.00 0.00

ATOM 975 C4B POP 153 98.500 30.340 69.760 1.00 0.00

ATOM 976 C5B POP 153 102.660 32.470 69.530 1.00 0.00

ATOM 977 NC3 POP 154 18.890 89.270 88.980 1.00 0.00

ATOM 978 PO4 POP 154 16.440 92.490 87.710 1.00 0.00

ATOM 979 GL1 POP 154 17.310 90.920 83.600 1.00 0.00

ATOM 980 GL2 POP 154 16.240 87.910 84.440 1.00 0.00

ATOM 981 C1A POP 154 16.740 84.180 83.770 1.00 0.00

ATOM 982 C2A POP 154 15.490 80.670 82.320 1.00 0.00

ATOM 983 C3A POP 154 11.240 77.740 80.000 1.00 0.00

ATOM 984 C4A POP 154 7.750 75.910 81.560 1.00 0.00

ATOM 985 C1B POP 154 18.210 91.770 79.520 1.00 0.00

ATOM 986 C2B POP 154 21.150 92.930 76.160 1.00 0.00

ATOM 987 D3B POP 154 21.420 96.220 73.820 1.00 0.00

ATOM 988 C4B POP 154 19.560 96.090 69.260 1.00 0.00

ATOM 989 C5B POP 154 21.410 94.560 66.250 1.00 0.00

ATOM 990 NC3 POP 155 51.550 61.280 41.860 1.00 0.00

ATOM 991 PO4 POP 155 52.080 63.650 45.830 1.00 0.00

ATOM 992 GL1 POP 155 51.790 65.500 50.200 1.00 0.00

ATOM 993 GL2 POP 155 53.780 67.450 51.140 1.00 0.00

ATOM 994 C1A POP 155 53.330 71.900 51.810 1.00 0.00

ATOM 995 C2A POP 155 49.190 73.670 53.440 1.00 0.00

ATOM 996 C3A POP 155 44.960 74.850 53.090 1.00 0.00

ATOM 997 C4A POP 155 42.860 79.260 53.740 1.00 0.00

ATOM 998 C1B POP 155 47.980 69.230 51.340 1.00 0.00

ATOM 999 C2B POP 155 46.270 69.990 55.590 1.00 0.00

ATOM 1000 D3B POP 155 46.840 68.350 59.120 1.00 0.00

ATOM 1001 C4B POP 155 44.640 66.650 62.590 1.00 0.00

ATOM 1002 C5B POP 155 43.850 61.860 63.650 1.00 0.00

ATOM 1003 NC3 POP 156 84.610 97.480 41.070 1.00 0.00

ATOM 1004 PO4 POP 156 85.380 95.030 45.620 1.00 0.00

ATOM 1005 GL1 POP 156 83.050 95.760 49.060 1.00 0.00

ATOM 1006 GL2 POP 156 80.450 95.440 48.110 1.00 0.00

ATOM 1007 C1A POP 156 79.230 98.900 50.610 1.00 0.00

ATOM 1008 C2A POP 156 79.420 104.530 51.480 1.00 0.00

ATOM 1009 C3A POP 156 80.440 108.000 52.550 1.00 0.00

ATOM 1010 C4A POP 156 82.760 110.930 53.730 1.00 0.00

ATOM 1011 C1B POP 156 82.250 97.140 54.480 1.00 0.00

ATOM 1012 C2B POP 156 82.320 97.890 58.720 1.00 0.00

ATOM 1013 D3B POP 156 83.320 98.680 63.550 1.00 0.00

ATOM 1014 C4B POP 156 81.180 102.250 65.820 1.00 0.00

ATOM 1015 C5B POP 156 78.470 105.450 66.000 1.00 0.00

ATOM 1016 NC3 POP 157 8.130 78.150 43.200 1.00 0.00

ATOM 1017 PO4 POP 157 10.950 79.940 44.820 1.00 0.00

ATOM 1018 GL1 POP 157 10.180 80.210 48.750 1.00 0.00

ATOM 1019 GL2 POP 157 13.150 82.330 50.830 1.00 0.00

ATOM 1020 C1A POP 157 15.330 83.560 55.330 1.00 0.00

ATOM 1021 C2A POP 157 15.690 82.970 60.160 1.00 0.00

ATOM 1022 C3A POP 157 15.050 85.910 62.960 1.00 0.00

ATOM 1023 C4A POP 157 12.720 90.140 62.370 1.00 0.00

ATOM 1024 C1B POP 157 6.550 78.860 52.510 1.00 0.00

ATOM 1025 C2B POP 157 6.420 79.690 57.020 1.00 0.00

ATOM 1026 D3B POP 157 6.630 81.230 61.790 1.00 0.00

ATOM 1027 C4B POP 157 4.100 79.770 64.650 1.00 0.00

ATOM 1028 C5B POP 157 -0.800 76.650 65.620 1.00 0.00

ATOM 1029 NC3 POP 158 74.220 72.770 84.950 1.00 0.00

ATOM 1030 PO4 POP 158 69.940 70.760 84.110 1.00 0.00

ATOM 1031 GL1 POP 158 72.190 71.490 80.450 1.00 0.00

ATOM 1032 GL2 POP 158 75.840 72.480 80.470 1.00 0.00

ATOM 1033 C1A POP 158 77.540 69.590 77.690 1.00 0.00

ATOM 1034 C2A POP 158 76.970 68.120 73.570 1.00 0.00

ATOM 1035 C3A POP 158 76.800 63.370 71.860 1.00 0.00

ATOM 1036 C4A POP 158 77.430 60.230 68.360 1.00 0.00

ATOM 1037 C1B POP 158 71.090 74.080 77.400 1.00 0.00

ATOM 1038 C2B POP 158 68.240 74.050 73.940 1.00 0.00

ATOM 1039 D3B POP 158 65.740 73.720 70.380 1.00 0.00

ATOM 1040 C4B POP 158 63.540 76.030 68.360 1.00 0.00

ATOM 1041 C5B POP 158 60.080 79.160 68.730 1.00 0.00

ATOM 1042 NC3 POP 159 87.730 95.790 91.860 1.00 0.00

ATOM 1043 PO4 POP 159 87.190 91.860 90.300 1.00 0.00

ATOM 1044 GL1 POP 159 86.920 90.140 86.610 1.00 0.00

ATOM 1045 GL2 POP 159 90.000 89.230 86.380 1.00 0.00

ATOM 1046 C1A POP 159 90.240 91.900 82.600 1.00 0.00

ATOM 1047 C2A POP 159 91.490 91.030 78.460 1.00 0.00

ATOM 1048 C3A POP 159 92.130 90.210 74.880 1.00 0.00

ATOM 1049 C4A POP 159 92.950 93.360 71.260 1.00 0.00

ATOM 1050 C1B POP 159 84.810 90.380 82.750 1.00 0.00

ATOM 1051 C2B POP 159 86.560 90.650 78.980 1.00 0.00

ATOM 1052 D3B POP 159 85.750 91.710 74.440 1.00 0.00

ATOM 1053 C4B POP 159 81.820 89.610 72.920 1.00 0.00

ATOM 1054 C5B POP 159 78.810 87.920 75.300 1.00 0.00

ATOM 1055 NC3 POP 160 71.690 98.350 42.990 1.00 0.00

ATOM 1056 PO4 POP 160 71.470 94.800 45.270 1.00 0.00

ATOM 1057 GL1 POP 160 72.190 96.160 49.290 1.00 0.00

ATOM 1058 GL2 POP 160 75.250 95.110 49.620 1.00 0.00

ATOM 1059 C1A POP 160 76.420 95.840 53.430 1.00 0.00

ATOM 1060 C2A POP 160 78.320 99.870 55.440 1.00 0.00

ATOM 1061 C3A POP 160 79.260 103.990 56.990 1.00 0.00

ATOM 1062 C4A POP 160 79.340 109.330 57.330 1.00 0.00

ATOM 1063 C1B POP 160 71.400 96.020 53.330 1.00 0.00

ATOM 1064 C2B POP 160 73.470 98.220 56.510 1.00 0.00

ATOM 1065 D3B POP 160 73.840 96.920 61.130 1.00 0.00

ATOM 1066 C4B POP 160 70.870 94.040 61.870 1.00 0.00

ATOM 1067 C5B POP 160 68.740 93.040 65.740 1.00 0.00

ATOM 1068 NC3 POP 161 11.630 100.460 44.940 1.00 0.00

ATOM 1069 PO4 POP 161 9.980 104.170 45.090 1.00 0.00

ATOM 1070 GL1 POP 161 12.750 107.190 47.890 1.00 0.00

ATOM 1071 GL2 POP 161 11.980 111.540 47.250 1.00 0.00

ATOM 1072 C1A POP 161 8.770 111.680 50.050 1.00 0.00

ATOM 1073 C2A POP 161 8.510 112.350 53.830 1.00 0.00

ATOM 1074 C3A POP 161 6.150 111.480 57.280 1.00 0.00

ATOM 1075 C4A POP 161 5.940 112.470 61.180 1.00 0.00

ATOM 1076 C1B POP 161 11.730 108.160 52.180 1.00 0.00

ATOM 1077 C2B POP 161 11.020 109.320 56.800 1.00 0.00

ATOM 1078 D3B POP 161 10.580 107.790 60.910 1.00 0.00

ATOM 1079 C4B POP 161 9.920 109.910 64.930 1.00 0.00

ATOM 1080 C5B POP 161 11.050 113.680 68.060 1.00 0.00

ATOM 1081 NC3 POP 162 59.990 26.280 88.160 1.00 0.00

ATOM 1082 PO4 POP 162 61.570 29.780 87.230 1.00 0.00

ATOM 1083 GL1 POP 162 59.880 29.950 83.880 1.00 0.00

ATOM 1084 GL2 POP 162 61.090 32.900 82.700 1.00 0.00

ATOM 1085 C1A POP 162 59.190 35.730 79.780 1.00 0.00

ATOM 1086 C2A POP 162 56.880 38.070 77.070 1.00 0.00

ATOM 1087 C3A POP 162 56.000 40.130 72.540 1.00 0.00

ATOM 1088 C4A POP 162 58.290 41.530 69.560 1.00 0.00

ATOM 1089 C1B POP 162 59.500 30.750 79.180 1.00 0.00

ATOM 1090 C2B POP 162 58.960 33.950 75.670 1.00 0.00

ATOM 1091 D3B POP 162 57.780 35.530 72.220 1.00 0.00

ATOM 1092 C4B POP 162 59.330 35.290 67.910 1.00 0.00

ATOM 1093 C5B POP 162 62.510 35.400 65.520 1.00 0.00

ATOM 1094 NC3 POP 163 85.370 65.000 90.330 1.00 0.00

ATOM 1095 PO4 POP 163 88.700 63.870 90.690 1.00 0.00

ATOM 1096 GL1 POP 163 88.750 63.310 85.740 1.00 0.00

ATOM 1097 GL2 POP 163 91.780 60.740 83.650 1.00 0.00

ATOM 1098 C1A POP 163 91.390 58.340 80.160 1.00 0.00

ATOM 1099 C2A POP 163 92.830 57.960 76.350 1.00 0.00

ATOM 1100 C3A POP 163 92.340 56.220 71.320 1.00 0.00

ATOM 1101 C4A POP 163 94.450 55.070 66.890 1.00 0.00

ATOM 1102 C1B POP 163 88.010 63.420 81.280 1.00 0.00

ATOM 1103 C2B POP 163 89.080 62.450 77.210 1.00 0.00

ATOM 1104 D3B POP 163 90.500 65.840 74.700 1.00 0.00

ATOM 1105 C4B POP 163 93.760 64.090 71.380 1.00 0.00

ATOM 1106 C5B POP 163 95.930 60.580 72.700 1.00 0.00

ATOM 1107 NC3 POP 164 41.390 81.330 47.950 1.00 0.00

ATOM 1108 PO4 POP 164 44.280 79.100 47.270 1.00 0.00

ATOM 1109 GL1 POP 164 46.860 79.820 51.390 1.00 0.00

ATOM 1110 GL2 POP 164 45.630 83.130 51.870 1.00 0.00

ATOM 1111 C1A POP 164 45.790 81.710 56.760 1.00 0.00

ATOM 1112 C2A POP 164 45.860 76.480 57.380 1.00 0.00

ATOM 1113 C3A POP 164 48.940 72.870 58.950 1.00 0.00

ATOM 1114 C4A POP 164 48.680 70.260 63.110 1.00 0.00

ATOM 1115 C1B POP 164 50.570 81.800 51.970 1.00 0.00

ATOM 1116 C2B POP 164 50.170 85.050 54.360 1.00 0.00

ATOM 1117 D3B POP 164 50.490 83.520 58.310 1.00 0.00

ATOM 1118 C4B POP 164 51.660 83.560 62.660 1.00 0.00

ATOM 1119 C5B POP 164 53.490 84.440 66.200 1.00 0.00

ATOM 1120 NC3 POP 165 68.680 17.110 84.010 1.00 0.00

ATOM 1121 PO4 POP 165 64.630 17.680 84.010 1.00 0.00

ATOM 1122 GL1 POP 165 62.590 19.230 80.170 1.00 0.00

ATOM 1123 GL2 POP 165 60.840 21.260 78.970 1.00 0.00

ATOM 1124 C1A POP 165 63.710 23.470 75.290 1.00 0.00

ATOM 1125 C2A POP 165 67.300 22.240 72.120 1.00 0.00

ATOM 1126 C3A POP 165 70.740 20.380 69.250 1.00 0.00

ATOM 1127 C4A POP 165 75.260 21.160 66.270 1.00 0.00

ATOM 1128 C1B POP 165 65.640 16.980 76.170 1.00 0.00

ATOM 1129 C2B POP 165 66.620 17.560 71.550 1.00 0.00

ATOM 1130 D3B POP 165 67.990 16.570 67.910 1.00 0.00

ATOM 1131 C4B POP 165 65.980 11.870 68.000 1.00 0.00

ATOM 1132 C5B POP 165 65.770 7.440 67.030 1.00 0.00

ATOM 1133 NC3 POP 166 86.920 32.200 43.500 1.00 0.00

ATOM 1134 PO4 POP 166 85.870 32.850 47.640 1.00 0.00

ATOM 1135 GL1 POP 166 85.040 31.100 51.430 1.00 0.00

ATOM 1136 GL2 POP 166 81.760 31.440 51.370 1.00 0.00

ATOM 1137 C1A POP 166 80.580 35.100 53.420 1.00 0.00

ATOM 1138 C2A POP 166 82.800 39.280 53.950 1.00 0.00

ATOM 1139 C3A POP 166 85.450 40.620 55.820 1.00 0.00

ATOM 1140 C4A POP 166 85.910 43.440 59.090 1.00 0.00

ATOM 1141 C1B POP 166 87.140 31.080 55.610 1.00 0.00

ATOM 1142 C2B POP 166 89.860 33.700 56.820 1.00 0.00

ATOM 1143 D3B POP 166 92.850 36.120 58.530 1.00 0.00

ATOM 1144 C4B POP 166 92.060 35.220 62.580 1.00 0.00

ATOM 1145 C5B POP 166 90.340 33.570 66.510 1.00 0.00

ATOM 1146 NC3 POP 167 65.760 6.500 82.140 1.00 0.00

ATOM 1147 PO4 POP 167 69.770 7.410 81.450 1.00 0.00

ATOM 1148 GL1 POP 167 72.010 8.350 79.720 1.00 0.00

ATOM 1149 GL2 POP 167 72.250 11.380 77.300 1.00 0.00

ATOM 1150 C1A POP 167 75.370 11.250 73.510 1.00 0.00

ATOM 1151 C2A POP 167 77.850 12.690 69.400 1.00 0.00

ATOM 1152 C3A POP 167 79.220 17.420 68.570 1.00 0.00

ATOM 1153 C4A POP 167 81.020 21.040 65.620 1.00 0.00

ATOM 1154 C1B POP 167 74.370 7.160 75.910 1.00 0.00

ATOM 1155 C2B POP 167 78.010 6.810 74.350 1.00 0.00

ATOM 1156 D3B POP 167 80.160 8.570 71.180 1.00 0.00

ATOM 1157 C4B POP 167 84.040 9.490 68.950 1.00 0.00

ATOM 1158 C5B POP 167 87.950 7.590 70.000 1.00 0.00

ATOM 1159 NC3 POP 168 72.000 67.350 92.230 1.00 0.00

ATOM 1160 PO4 POP 168 71.310 66.550 87.600 1.00 0.00

ATOM 1161 GL1 POP 168 69.480 65.660 83.120 1.00 0.00

ATOM 1162 GL2 POP 168 66.080 64.190 82.650 1.00 0.00

ATOM 1163 C1A POP 168 68.990 60.820 80.840 1.00 0.00

ATOM 1164 C2A POP 168 70.500 57.990 77.110 1.00 0.00

ATOM 1165 C3A POP 168 70.670 56.250 73.030 1.00 0.00

ATOM 1166 C4A POP 168 70.540 53.010 70.680 1.00 0.00

ATOM 1167 C1B POP 168 69.000 65.560 78.940 1.00 0.00

ATOM 1168 C2B POP 168 69.040 62.770 75.590 1.00 0.00

ATOM 1169 D3B POP 168 67.530 61.530 72.820 1.00 0.00

ATOM 1170 C4B POP 168 65.570 62.390 68.060 1.00 0.00

ATOM 1171 C5B POP 168 65.720 62.540 63.570 1.00 0.00

ATOM 1172 NC3 POP 169 24.000 21.460 45.550 1.00 0.00

ATOM 1173 PO4 POP 169 20.140 19.230 46.710 1.00 0.00

ATOM 1174 GL1 POP 169 18.350 20.310 50.200 1.00 0.00

ATOM 1175 GL2 POP 169 20.690 20.900 51.820 1.00 0.00

ATOM 1176 C1A POP 169 18.840 21.930 56.510 1.00 0.00

ATOM 1177 C2A POP 169 17.690 19.870 59.420 1.00 0.00

ATOM 1178 C3A POP 169 16.590 19.310 63.690 1.00 0.00

ATOM 1179 C4A POP 169 12.630 19.650 67.320 1.00 0.00

ATOM 1180 C1B POP 169 15.000 21.060 52.570 1.00 0.00

ATOM 1181 C2B POP 169 12.700 21.790 56.160 1.00 0.00

ATOM 1182 D3B POP 169 11.960 20.400 59.890 1.00 0.00

ATOM 1183 C4B POP 169 13.030 23.480 62.290 1.00 0.00

ATOM 1184 C5B POP 169 11.510 25.530 65.430 1.00 0.00

ATOM 1185 NC3 POP 170 79.410 78.830 86.990 1.00 0.00

ATOM 1186 PO4 POP 170 75.130 78.610 85.930 1.00 0.00

ATOM 1187 GL1 POP 170 75.690 77.120 81.750 1.00 0.00

ATOM 1188 GL2 POP 170 72.320 76.240 81.600 1.00 0.00

ATOM 1189 C1A POP 170 72.140 79.450 79.120 1.00 0.00

ATOM 1190 C2A POP 170 72.680 80.080 75.550 1.00 0.00

ATOM 1191 C3A POP 170 71.610 77.350 72.210 1.00 0.00

ATOM 1192 C4A POP 170 71.420 74.910 68.830 1.00 0.00

ATOM 1193 C1B POP 170 76.610 77.130 77.770 1.00 0.00

ATOM 1194 C2B POP 170 77.690 78.670 73.720 1.00 0.00

ATOM 1195 D3B POP 170 77.130 75.950 69.440 1.00 0.00

ATOM 1196 C4B POP 170 79.110 71.540 69.260 1.00 0.00

ATOM 1197 C5B POP 170 80.670 67.560 70.170 1.00 0.00

ATOM 1198 NC3 POP 171 87.920 14.630 89.120 1.00 0.00

ATOM 1199 PO4 POP 171 89.670 16.630 86.770 1.00 0.00

ATOM 1200 GL1 POP 171 88.790 18.650 82.550 1.00 0.00

ATOM 1201 GL2 POP 171 85.060 17.730 82.400 1.00 0.00

ATOM 1202 C1A POP 171 85.680 19.260 78.620 1.00 0.00

ATOM 1203 C2A POP 171 87.030 19.590 75.300 1.00 0.00

ATOM 1204 C3A POP 171 87.180 21.740 71.460 1.00 0.00

ATOM 1205 C4A POP 171 89.830 21.250 67.920 1.00 0.00

ATOM 1206 C1B POP 171 91.280 19.320 77.960 1.00 0.00

ATOM 1207 C2B POP 171 91.590 18.690 73.350 1.00 0.00

ATOM 1208 D3B POP 171 88.920 17.060 70.380 1.00 0.00

ATOM 1209 C4B POP 171 83.970 18.030 70.920 1.00 0.00

ATOM 1210 C5B POP 171 82.730 22.880 69.780 1.00 0.00

ATOM 1211 NC3 POP 172 66.670 68.090 86.540 1.00 0.00

ATOM 1212 PO4 POP 172 63.970 65.020 86.750 1.00 0.00

ATOM 1213 GL1 POP 172 61.540 67.440 83.320 1.00 0.00

ATOM 1214 GL2 POP 172 65.270 69.280 82.080 1.00 0.00

ATOM 1215 C1A POP 172 66.270 70.430 77.800 1.00 0.00

ATOM 1216 C2A POP 172 69.140 68.640 75.070 1.00 0.00

ATOM 1217 C3A POP 172 72.460 70.640 73.020 1.00 0.00

ATOM 1218 C4A POP 172 76.050 73.600 73.930 1.00 0.00

ATOM 1219 C1B POP 172 62.110 65.880 79.070 1.00 0.00

ATOM 1220 C2B POP 172 64.940 66.420 75.290 1.00 0.00

ATOM 1221 D3B POP 172 65.660 66.670 72.050 1.00 0.00

ATOM 1222 C4B POP 172 68.470 69.710 69.680 1.00 0.00

ATOM 1223 C5B POP 172 69.370 70.960 65.610 1.00 0.00

ATOM 1224 NC3 POP 173 104.360 108.530 87.370 1.00 0.00

ATOM 1225 PO4 POP 173 105.130 103.590 86.370 1.00 0.00

ATOM 1226 GL1 POP 173 107.400 103.910 81.930 1.00 0.00

ATOM 1227 GL2 POP 173 107.720 101.120 80.790 1.00 0.00

ATOM 1228 C1A POP 173 107.690 97.770 77.040 1.00 0.00

ATOM 1229 C2A POP 173 106.380 96.780 72.840 1.00 0.00

ATOM 1230 C3A POP 173 106.370 92.580 71.220 1.00 0.00

ATOM 1231 C4A POP 173 108.230 88.960 72.550 1.00 0.00

ATOM 1232 C1B POP 173 109.370 105.290 77.910 1.00 0.00

ATOM 1233 C2B POP 173 108.790 102.290 74.660 1.00 0.00

ATOM 1234 D3B POP 173 106.080 101.810 70.590 1.00 0.00

ATOM 1235 C4B POP 173 108.160 98.870 68.480 1.00 0.00

ATOM 1236 C5B POP 173 108.550 94.900 67.520 1.00 0.00

ATOM 1237 NC3 POP 174 52.770 23.970 38.570 1.00 0.00

ATOM 1238 PO4 POP 174 51.590 22.940 42.100 1.00 0.00

ATOM 1239 GL1 POP 174 50.810 22.580 46.510 1.00 0.00

ATOM 1240 GL2 POP 174 47.870 24.230 48.400 1.00 0.00

ATOM 1241 C1A POP 174 47.100 25.130 52.200 1.00 0.00

ATOM 1242 C2A POP 174 47.100 26.030 57.280 1.00 0.00

ATOM 1243 C3A POP 174 45.540 28.110 59.950 1.00 0.00

ATOM 1244 C4A POP 174 45.930 31.320 62.320 1.00 0.00

ATOM 1245 C1B POP 174 51.620 20.730 50.970 1.00 0.00

ATOM 1246 C2B POP 174 47.760 19.870 55.070 1.00 0.00

ATOM 1247 D3B POP 174 44.220 19.240 57.600 1.00 0.00

ATOM 1248 C4B POP 174 39.370 20.900 56.440 1.00 0.00

ATOM 1249 C5B POP 174 35.400 22.980 57.500 1.00 0.00

ATOM 1250 NC3 POP 175 14.320 80.100 90.130 1.00 0.00

ATOM 1251 PO4 POP 175 11.780 82.570 89.510 1.00 0.00

ATOM 1252 GL1 POP 175 11.640 84.500 85.300 1.00 0.00

ATOM 1253 GL2 POP 175 9.450 86.570 84.330 1.00 0.00

ATOM 1254 C1A POP 175 10.500 89.460 80.590 1.00 0.00

ATOM 1255 C2A POP 175 11.210 91.070 77.190 1.00 0.00

ATOM 1256 C3A POP 175 12.520 88.550 73.160 1.00 0.00

ATOM 1257 C4A POP 175 13.880 90.220 70.390 1.00 0.00

ATOM 1258 C1B POP 175 9.810 83.040 80.320 1.00 0.00

ATOM 1259 C2B POP 175 12.390 83.530 75.210 1.00 0.00

ATOM 1260 D3B POP 175 14.380 81.580 73.120 1.00 0.00

ATOM 1261 C4B POP 175 18.200 79.270 72.380 1.00 0.00

ATOM 1262 C5B POP 175 20.860 78.750 67.980 1.00 0.00

ATOM 1263 NC3 POP 176 12.600 53.150 48.260 1.00 0.00

ATOM 1264 PO4 POP 176 10.760 56.470 48.670 1.00 0.00

ATOM 1265 GL1 POP 176 11.250 56.600 52.460 1.00 0.00

ATOM 1266 GL2 POP 176 11.810 59.740 54.030 1.00 0.00

ATOM 1267 C1A POP 176 15.280 60.500 54.920 1.00 0.00

ATOM 1268 C2A POP 176 17.980 63.580 56.550 1.00 0.00

ATOM 1269 C3A POP 176 20.310 62.850 60.710 1.00 0.00

ATOM 1270 C4A POP 176 18.630 62.320 64.750 1.00 0.00

ATOM 1271 C1B POP 176 10.940 54.320 56.480 1.00 0.00

ATOM 1272 C2B POP 176 9.470 51.300 58.330 1.00 0.00

ATOM 1273 D3B POP 176 7.540 48.060 59.340 1.00 0.00

ATOM 1274 C4B POP 176 3.820 47.640 60.930 1.00 0.00

ATOM 1275 C5B POP 176 3.140 43.740 61.890 1.00 0.00

ATOM 1276 NC3 POP 177 15.460 59.600 90.910 1.00 0.00

ATOM 1277 PO4 POP 177 15.430 55.280 92.360 1.00 0.00

ATOM 1278 GL1 POP 177 15.780 55.660 87.830 1.00 0.00

ATOM 1279 GL2 POP 177 13.040 57.810 87.100 1.00 0.00

ATOM 1280 C1A POP 177 11.530 57.120 82.530 1.00 0.00

ATOM 1281 C2A POP 177 10.310 58.910 78.250 1.00 0.00

ATOM 1282 C3A POP 177 11.430 61.060 73.590 1.00 0.00

ATOM 1283 C4A POP 177 10.910 65.150 71.400 1.00 0.00

ATOM 1284 C1B POP 177 16.760 57.560 84.330 1.00 0.00

ATOM 1285 C2B POP 177 16.130 57.450 79.560 1.00 0.00

ATOM 1286 D3B POP 177 15.890 58.820 75.530 1.00 0.00

ATOM 1287 C4B POP 177 15.890 54.480 73.310 1.00 0.00

ATOM 1288 C5B POP 177 15.500 50.990 71.730 1.00 0.00

ATOM 1289 NC3 POP 178 39.340 10.430 44.880 1.00 0.00

ATOM 1290 PO4 POP 178 37.330 6.510 44.490 1.00 0.00

ATOM 1291 GL1 POP 178 36.540 4.720 47.800 1.00 0.00

ATOM 1292 GL2 POP 178 36.710 7.550 50.070 1.00 0.00

ATOM 1293 C1A POP 178 33.770 5.740 52.670 1.00 0.00

ATOM 1294 C2A POP 178 34.740 2.580 56.260 1.00 0.00

ATOM 1295 C3A POP 178 33.040 0.490 60.940 1.00 0.00

ATOM 1296 C4A POP 178 30.810 4.150 63.100 1.00 0.00

ATOM 1297 C1B POP 178 37.220 2.400 51.980 1.00 0.00

ATOM 1298 C2B POP 178 39.320 0.310 54.120 1.00 0.00

ATOM 1299 D3B POP 178 40.690 1.820 57.720 1.00 0.00

ATOM 1300 C4B POP 178 39.210 2.330 62.460 1.00 0.00

ATOM 1301 C5B POP 178 35.820 4.240 64.450 1.00 0.00

ATOM 1302 NC3 POP 179 31.650 107.200 43.370 1.00 0.00

ATOM 1303 PO4 POP 179 35.910 107.010 43.970 1.00 0.00

ATOM 1304 GL1 POP 179 34.080 103.710 46.730 1.00 0.00

ATOM 1305 GL2 POP 179 37.270 101.490 46.480 1.00 0.00

ATOM 1306 C1A POP 179 36.630 99.180 50.620 1.00 0.00

ATOM 1307 C2A POP 179 36.140 98.170 54.850 1.00 0.00

ATOM 1308 C3A POP 179 33.080 98.100 58.130 1.00 0.00

ATOM 1309 C4A POP 179 31.900 97.190 61.660 1.00 0.00

ATOM 1310 C1B POP 179 33.340 103.830 51.060 1.00 0.00

ATOM 1311 C2B POP 179 31.630 102.300 55.960 1.00 0.00

ATOM 1312 D3B POP 179 29.000 100.530 59.310 1.00 0.00

ATOM 1313 C4B POP 179 29.630 101.230 63.300 1.00 0.00

ATOM 1314 C5B POP 179 28.470 100.680 68.020 1.00 0.00

ATOM 1315 NC3 POP 180 16.470 12.150 42.170 1.00 0.00

ATOM 1316 PO4 POP 180 20.540 14.290 43.930 1.00 0.00

ATOM 1317 GL1 POP 180 17.620 14.110 47.570 1.00 0.00

ATOM 1318 GL2 POP 180 14.590 13.870 46.750 1.00 0.00

ATOM 1319 C1A POP 180 13.080 12.080 50.250 1.00 0.00

ATOM 1320 C2A POP 180 13.970 10.170 53.780 1.00 0.00

ATOM 1321 C3A POP 180 12.310 7.140 55.500 1.00 0.00

ATOM 1322 C4A POP 180 10.420 4.190 59.980 1.00 0.00

ATOM 1323 C1B POP 180 17.260 14.440 52.450 1.00 0.00

ATOM 1324 C2B POP 180 15.480 16.480 56.430 1.00 0.00

ATOM 1325 D3B POP 180 14.420 15.270 60.340 1.00 0.00

ATOM 1326 C4B POP 180 11.440 15.980 63.200 1.00 0.00

ATOM 1327 C5B POP 180 8.590 17.420 65.880 1.00 0.00

ATOM 1328 NC3 POP 181 78.900 54.610 40.260 1.00 0.00

ATOM 1329 PO4 POP 181 76.870 53.280 44.450 1.00 0.00

ATOM 1330 GL1 POP 181 77.080 53.430 48.540 1.00 0.00

ATOM 1331 GL2 POP 181 78.260 49.710 49.480 1.00 0.00

ATOM 1332 C1A POP 181 79.680 50.840 53.430 1.00 0.00

ATOM 1333 C2A POP 181 83.670 53.000 55.890 1.00 0.00

ATOM 1334 C3A POP 181 87.270 54.170 58.210 1.00 0.00

ATOM 1335 C4A POP 181 86.860 58.110 60.870 1.00 0.00

ATOM 1336 C1B POP 181 75.920 54.110 52.840 1.00 0.00

ATOM 1337 C2B POP 181 73.420 55.830 57.100 1.00 0.00

ATOM 1338 D3B POP 181 74.210 53.850 61.300 1.00 0.00

ATOM 1339 C4B POP 181 74.860 56.670 63.350 1.00 0.00

ATOM 1340 C5B POP 181 78.280 57.570 64.170 1.00 0.00

ATOM 1341 NC3 POP 182 108.270 29.780 87.780 1.00 0.00

ATOM 1342 PO4 POP 182 103.530 31.620 87.480 1.00 0.00

ATOM 1343 GL1 POP 182 101.460 31.560 83.360 1.00 0.00

ATOM 1344 GL2 POP 182 98.650 29.930 84.360 1.00 0.00

ATOM 1345 C1A POP 182 96.770 32.090 80.890 1.00 0.00

ATOM 1346 C2A POP 182 94.970 32.620 77.150 1.00 0.00

ATOM 1347 C3A POP 182 94.940 32.900 71.520 1.00 0.00

ATOM 1348 C4A POP 182 95.270 31.200 66.110 1.00 0.00

ATOM 1349 C1B POP 182 101.780 33.630 78.230 1.00 0.00

ATOM 1350 C2B POP 182 99.890 34.130 74.340 1.00 0.00

ATOM 1351 D3B POP 182 98.450 35.920 70.680 1.00 0.00

ATOM 1352 C4B POP 182 101.510 38.190 68.770 1.00 0.00

ATOM 1353 C5B POP 182 105.240 40.160 70.350 1.00 0.00

ATOM 1354 NC3 POP 183 55.010 -2.850 43.840 1.00 0.00

ATOM 1355 PO4 POP 183 52.550 -2.160 47.490 1.00 0.00

ATOM 1356 GL1 POP 183 53.520 -1.220 51.330 1.00 0.00

ATOM 1357 GL2 POP 183 54.970 3.210 51.570 1.00 0.00

ATOM 1358 C1A POP 183 53.280 6.860 53.960 1.00 0.00

ATOM 1359 C2A POP 183 56.880 8.550 56.590 1.00 0.00

ATOM 1360 C3A POP 183 60.870 8.600 58.380 1.00 0.00

ATOM 1361 C4A POP 183 62.400 4.410 57.440 1.00 0.00

ATOM 1362 C1B POP 183 52.000 0.470 55.350 1.00 0.00

ATOM 1363 C2B POP 183 51.850 2.990 56.990 1.00 0.00

ATOM 1364 D3B POP 183 52.920 7.240 59.210 1.00 0.00

ATOM 1365 C4B POP 183 51.590 8.090 63.140 1.00 0.00

ATOM 1366 C5B POP 183 49.060 11.310 64.490 1.00 0.00

ATOM 1367 NC3 POP 184 85.150 40.390 93.740 1.00 0.00

ATOM 1368 PO4 POP 184 86.400 39.360 90.020 1.00 0.00

ATOM 1369 GL1 POP 184 87.360 39.940 85.550 1.00 0.00

ATOM 1370 GL2 POP 184 84.490 39.300 83.740 1.00 0.00

ATOM 1371 C1A POP 184 82.120 36.830 80.920 1.00 0.00

ATOM 1372 C2A POP 184 83.090 33.310 76.870 1.00 0.00

ATOM 1373 C3A POP 184 83.270 29.330 74.880 1.00 0.00

ATOM 1374 C4A POP 184 86.590 27.490 71.630 1.00 0.00

ATOM 1375 C1B POP 184 90.480 40.520 82.240 1.00 0.00

ATOM 1376 C2B POP 184 93.030 40.700 79.090 1.00 0.00

ATOM 1377 D3B POP 184 94.530 42.960 76.620 1.00 0.00

ATOM 1378 C4B POP 184 89.900 43.800 74.130 1.00 0.00

ATOM 1379 C5B POP 184 86.060 46.510 70.620 1.00 0.00

ATOM 1380 NC3 POP 185 53.450 19.860 85.460 1.00 0.00

ATOM 1381 PO4 POP 185 49.280 18.700 85.600 1.00 0.00

ATOM 1382 GL1 POP 185 44.680 18.510 83.070 1.00 0.00

ATOM 1383 GL2 POP 185 44.300 15.750 82.040 1.00 0.00

ATOM 1384 C1A POP 185 41.710 14.560 78.150 1.00 0.00

ATOM 1385 C2A POP 185 40.510 12.990 74.230 1.00 0.00

ATOM 1386 C3A POP 185 40.450 10.320 69.860 1.00 0.00

ATOM 1387 C4A POP 185 38.180 10.260 65.530 1.00 0.00

ATOM 1388 C1B POP 185 42.430 20.240 80.220 1.00 0.00

ATOM 1389 C2B POP 185 44.020 20.200 75.690 1.00 0.00

ATOM 1390 D3B POP 185 48.630 21.700 73.570 1.00 0.00

ATOM 1391 C4B POP 185 52.120 21.130 70.170 1.00 0.00

ATOM 1392 C5B POP 185 56.660 21.920 69.290 1.00 0.00

ATOM 1393 NC3 POP 186 74.060 45.640 86.050 1.00 0.00

ATOM 1394 PO4 POP 186 71.220 48.880 88.160 1.00 0.00

ATOM 1395 GL1 POP 186 70.120 48.630 84.520 1.00 0.00

ATOM 1396 GL2 POP 186 72.740 49.430 82.460 1.00 0.00

ATOM 1397 C1A POP 186 72.290 47.720 78.620 1.00 0.00

ATOM 1398 C2A POP 186 70.360 48.050 75.100 1.00 0.00

ATOM 1399 C3A POP 186 71.660 47.760 71.980 1.00 0.00

ATOM 1400 C4A POP 186 75.920 46.530 70.560 1.00 0.00

ATOM 1401 C1B POP 186 67.720 47.480 80.780 1.00 0.00

ATOM 1402 C2B POP 186 66.300 50.280 77.070 1.00 0.00

ATOM 1403 D3B POP 186 65.980 53.070 74.700 1.00 0.00

ATOM 1404 C4B POP 186 65.840 58.160 75.730 1.00 0.00

ATOM 1405 C5B POP 186 64.790 61.430 78.320 1.00 0.00

ATOM 1406 NC3 POP 187 19.470 66.460 90.790 1.00 0.00

ATOM 1407 PO4 POP 187 21.170 69.510 90.100 1.00 0.00

ATOM 1408 GL1 POP 187 21.160 70.090 86.130 1.00 0.00

ATOM 1409 GL2 POP 187 19.110 66.730 85.080 1.00 0.00

ATOM 1410 C1A POP 187 19.160 66.790 79.930 1.00 0.00

ATOM 1411 C2A POP 187 18.070 68.630 75.900 1.00 0.00

ATOM 1412 C3A POP 187 21.120 71.250 74.080 1.00 0.00

ATOM 1413 C4A POP 187 21.900 74.480 72.150 1.00 0.00

ATOM 1414 C1B POP 187 22.780 73.780 83.720 1.00 0.00

ATOM 1415 C2B POP 187 20.270 77.750 82.960 1.00 0.00

ATOM 1416 D3B POP 187 20.530 81.680 80.510 1.00 0.00

ATOM 1417 C4B POP 187 21.400 80.990 76.080 1.00 0.00

ATOM 1418 C5B POP 187 25.290 83.910 74.130 1.00 0.00

ATOM 1419 NC3 POP 188 93.600 19.360 87.570 1.00 0.00

ATOM 1420 PO4 POP 188 98.660 20.670 87.130 1.00 0.00

ATOM 1421 GL1 POP 188 98.320 21.420 82.940 1.00 0.00

ATOM 1422 GL2 POP 188 101.940 22.380 82.550 1.00 0.00

ATOM 1423 C1A POP 188 102.310 21.170 78.300 1.00 0.00

ATOM 1424 C2A POP 188 102.450 20.650 73.590 1.00 0.00

ATOM 1425 C3A POP 188 100.950 21.300 69.730 1.00 0.00

ATOM 1426 C4A POP 188 99.220 21.380 66.100 1.00 0.00

ATOM 1427 C1B POP 188 96.420 21.960 78.390 1.00 0.00

ATOM 1428 C2B POP 188 93.820 22.900 75.260 1.00 0.00

ATOM 1429 D3B POP 188 95.900 22.910 70.340 1.00 0.00

ATOM 1430 C4B POP 188 96.050 26.290 67.540 1.00 0.00

ATOM 1431 C5B POP 188 99.630 28.330 65.050 1.00 0.00

ATOM 1432 NC3 POP 189 40.280 33.070 85.720 1.00 0.00

ATOM 1433 PO4 POP 189 41.710 30.510 82.600 1.00 0.00

ATOM 1434 GL1 POP 189 43.140 30.090 79.450 1.00 0.00

ATOM 1435 GL2 POP 189 47.290 31.200 79.230 1.00 0.00

ATOM 1436 C1A POP 189 47.540 30.770 75.230 1.00 0.00

ATOM 1437 C2A POP 189 45.410 30.200 71.380 1.00 0.00

ATOM 1438 C3A POP 189 47.550 29.360 67.960 1.00 0.00

ATOM 1439 C4A POP 189 49.050 26.360 64.380 1.00 0.00

ATOM 1440 C1B POP 189 42.290 31.380 75.280 1.00 0.00

ATOM 1441 C2B POP 189 42.660 34.500 71.730 1.00 0.00

ATOM 1442 D3B POP 189 43.920 33.400 67.460 1.00 0.00

ATOM 1443 C4B POP 189 41.150 31.380 65.070 1.00 0.00

ATOM 1444 C5B POP 189 37.900 27.900 64.830 1.00 0.00

ATOM 1445 NC3 POP 190 11.370 70.210 46.570 1.00 0.00

ATOM 1446 PO4 POP 190 15.130 72.500 48.180 1.00 0.00

ATOM 1447 GL1 POP 190 16.080 70.160 51.380 1.00 0.00

ATOM 1448 GL2 POP 190 12.190 69.720 51.670 1.00 0.00

ATOM 1449 C1A POP 190 11.830 69.360 55.940 1.00 0.00

ATOM 1450 C2A POP 190 10.560 68.010 59.860 1.00 0.00

ATOM 1451 C3A POP 190 10.040 64.340 62.460 1.00 0.00

ATOM 1452 C4A POP 190 12.990 60.600 63.410 1.00 0.00

ATOM 1453 C1B POP 190 17.430 68.900 55.860 1.00 0.00

ATOM 1454 C2B POP 190 15.270 68.820 59.910 1.00 0.00

ATOM 1455 D3B POP 190 13.190 71.130 63.460 1.00 0.00

ATOM 1456 C4B POP 190 15.500 71.250 66.660 1.00 0.00

ATOM 1457 C5B POP 190 17.430 73.430 70.210 1.00 0.00

ATOM 1458 NC3 POP 191 59.090 59.700 39.700 1.00 0.00

ATOM 1459 PO4 POP 191 59.020 59.390 44.050 1.00 0.00

ATOM 1460 GL1 POP 191 57.730 60.710 48.620 1.00 0.00

ATOM 1461 GL2 POP 191 61.200 60.940 49.300 1.00 0.00

ATOM 1462 C1A POP 191 59.810 59.460 53.450 1.00 0.00

ATOM 1463 C2A POP 191 57.040 61.120 57.300 1.00 0.00

ATOM 1464 C3A POP 191 54.040 64.180 59.320 1.00 0.00

ATOM 1465 C4A POP 191 52.120 66.660 61.770 1.00 0.00

ATOM 1466 C1B POP 191 54.550 61.200 52.290 1.00 0.00

ATOM 1467 C2B POP 191 52.770 63.460 54.260 1.00 0.00

ATOM 1468 D3B POP 191 49.820 65.370 56.490 1.00 0.00

ATOM 1469 C4B POP 191 51.540 69.090 55.530 1.00 0.00

ATOM 1470 C5B POP 191 53.390 73.090 56.610 1.00 0.00

ATOM 1471 NC3 POP 192 46.360 97.630 89.080 1.00 0.00

ATOM 1472 PO4 POP 192 41.860 98.390 87.120 1.00 0.00

ATOM 1473 GL1 POP 192 39.720 96.780 83.920 1.00 0.00

ATOM 1474 GL2 POP 192 39.260 93.760 82.470 1.00 0.00

ATOM 1475 C1A POP 192 38.240 91.840 79.050 1.00 0.00

ATOM 1476 C2A POP 192 41.120 90.050 76.530 1.00 0.00

ATOM 1477 C3A POP 192 45.430 87.450 76.300 1.00 0.00

ATOM 1478 C4A POP 192 45.530 86.870 72.920 1.00 0.00

ATOM 1479 C1B POP 192 41.940 97.200 80.350 1.00 0.00

ATOM 1480 C2B POP 192 41.620 94.720 76.650 1.00 0.00

ATOM 1481 D3B POP 192 43.360 92.440 72.990 1.00 0.00

ATOM 1482 C4B POP 192 39.710 90.930 70.620 1.00 0.00

ATOM 1483 C5B POP 192 39.330 87.030 68.200 1.00 0.00

ATOM 1484 NC3 POP 193 2.900 13.390 88.720 1.00 0.00

ATOM 1485 PO4 POP 193 2.910 8.870 89.810 1.00 0.00

ATOM 1486 GL1 POP 193 0.840 9.090 85.490 1.00 0.00

ATOM 1487 GL2 POP 193 1.660 11.050 83.620 1.00 0.00

ATOM 1488 C1A POP 193 2.030 11.150 79.030 1.00 0.00

ATOM 1489 C2A POP 193 3.880 10.130 75.890 1.00 0.00

ATOM 1490 C3A POP 193 3.590 10.550 71.290 1.00 0.00

ATOM 1491 C4A POP 193 6.270 8.890 67.470 1.00 0.00

ATOM 1492 C1B POP 193 -1.820 9.250 81.250 1.00 0.00

ATOM 1493 C2B POP 193 -2.290 10.130 76.460 1.00 0.00

ATOM 1494 D3B POP 193 -0.410 12.600 73.890 1.00 0.00

ATOM 1495 C4B POP 193 -1.630 16.400 73.100 1.00 0.00

ATOM 1496 C5B POP 193 -4.020 18.730 70.040 1.00 0.00

ATOM 1497 NC3 POP 194 37.390 73.830 46.080 1.00 0.00

ATOM 1498 PO4 POP 194 39.270 71.190 49.060 1.00 0.00

ATOM 1499 GL1 POP 194 39.280 72.900 53.240 1.00 0.00

ATOM 1500 GL2 POP 194 35.890 73.990 52.980 1.00 0.00

ATOM 1501 C1A POP 194 34.260 75.870 55.880 1.00 0.00

ATOM 1502 C2A POP 194 32.740 77.690 60.080 1.00 0.00

ATOM 1503 C3A POP 194 33.410 81.490 62.540 1.00 0.00

ATOM 1504 C4A POP 194 32.800 86.490 63.240 1.00 0.00

ATOM 1505 C1B POP 194 41.250 74.240 57.660 1.00 0.00

ATOM 1506 C2B POP 194 42.850 75.910 61.560 1.00 0.00

ATOM 1507 D3B POP 194 44.610 76.040 65.150 1.00 0.00

ATOM 1508 C4B POP 194 49.870 74.650 64.840 1.00 0.00

ATOM 1509 C5B POP 194 53.500 73.870 67.310 1.00 0.00

ATOM 1510 NC3 POP 195 45.080 109.460 87.300 1.00 0.00

ATOM 1511 PO4 POP 195 45.740 107.200 83.500 1.00 0.00

ATOM 1512 GL1 POP 195 45.630 106.140 79.660 1.00 0.00

ATOM 1513 GL2 POP 195 45.040 103.180 80.530 1.00 0.00

ATOM 1514 C1A POP 195 45.300 99.560 77.100 1.00 0.00

ATOM 1515 C2A POP 195 46.830 95.800 75.390 1.00 0.00

ATOM 1516 C3A POP 195 48.070 91.640 74.470 1.00 0.00

ATOM 1517 C4A POP 195 48.810 89.970 70.080 1.00 0.00

ATOM 1518 C1B POP 195 44.710 104.520 75.600 1.00 0.00

ATOM 1519 C2B POP 195 41.040 104.600 73.220 1.00 0.00

ATOM 1520 D3B POP 195 41.620 106.610 69.080 1.00 0.00

ATOM 1521 C4B POP 195 43.160 110.480 67.220 1.00 0.00

ATOM 1522 C5B POP 195 46.710 114.240 66.200 1.00 0.00

ATOM 1523 NC3 POP 196 19.620 89.150 42.840 1.00 0.00

ATOM 1524 PO4 POP 196 16.090 86.990 44.880 1.00 0.00

ATOM 1525 GL1 POP 196 14.670 88.880 47.430 1.00 0.00

ATOM 1526 GL2 POP 196 13.210 92.400 47.870 1.00 0.00

ATOM 1527 C1A POP 196 12.730 92.710 52.650 1.00 0.00

ATOM 1528 C2A POP 196 13.630 92.070 57.490 1.00 0.00

ATOM 1529 C3A POP 196 16.290 93.710 60.000 1.00 0.00

ATOM 1530 C4A POP 196 15.750 94.100 64.590 1.00 0.00

ATOM 1531 C1B POP 196 13.280 87.500 51.020 1.00 0.00

ATOM 1532 C2B POP 196 15.720 88.620 53.810 1.00 0.00

ATOM 1533 D3B POP 196 16.530 87.480 57.860 1.00 0.00

ATOM 1534 C4B POP 196 18.280 89.570 62.600 1.00 0.00

ATOM 1535 C5B POP 196 16.320 89.520 66.220 1.00 0.00

ATOM 1536 NC3 POP 197 16.050 16.770 43.150 1.00 0.00

ATOM 1537 PO4 POP 197 11.870 19.060 42.650 1.00 0.00

ATOM 1538 GL1 POP 197 11.410 17.610 46.800 1.00 0.00

ATOM 1539 GL2 POP 197 13.840 18.390 47.920 1.00 0.00

ATOM 1540 C1A POP 197 11.790 16.290 52.230 1.00 0.00

ATOM 1541 C2A POP 197 10.450 17.310 56.620 1.00 0.00

ATOM 1542 C3A POP 197 7.880 14.850 60.360 1.00 0.00

ATOM 1543 C4A POP 197 5.530 11.500 61.470 1.00 0.00

ATOM 1544 C1B POP 197 7.500 17.610 49.940 1.00 0.00

ATOM 1545 C2B POP 197 3.430 18.970 52.620 1.00 0.00

ATOM 1546 D3B POP 197 1.960 21.210 56.930 1.00 0.00

ATOM 1547 C4B POP 197 2.360 19.050 60.880 1.00 0.00

ATOM 1548 C5B POP 197 4.220 17.170 63.000 1.00 0.00

ATOM 1549 NC3 POP 198 87.760 108.410 87.290 1.00 0.00

ATOM 1550 PO4 POP 198 84.100 105.330 86.950 1.00 0.00

ATOM 1551 GL1 POP 198 83.070 107.160 83.470 1.00 0.00

ATOM 1552 GL2 POP 198 86.570 106.520 82.580 1.00 0.00

ATOM 1553 C1A POP 198 86.320 104.730 78.110 1.00 0.00

ATOM 1554 C2A POP 198 89.590 104.960 76.410 1.00 0.00

ATOM 1555 C3A POP 198 93.040 103.600 73.260 1.00 0.00

ATOM 1556 C4A POP 198 95.360 102.330 69.790 1.00 0.00

ATOM 1557 C1B POP 198 81.990 107.000 79.450 1.00 0.00

ATOM 1558 C2B POP 198 81.540 105.770 74.750 1.00 0.00

ATOM 1559 D3B POP 198 80.670 103.660 70.700 1.00 0.00

ATOM 1560 C4B POP 198 83.350 106.200 68.490 1.00 0.00

ATOM 1561 C5B POP 198 81.180 109.610 64.840 1.00 0.00

ATOM 1562 NC3 POP 199 29.030 53.890 42.800 1.00 0.00

ATOM 1563 PO4 POP 199 28.900 56.750 46.050 1.00 0.00

ATOM 1564 GL1 POP 199 27.450 55.520 49.540 1.00 0.00

ATOM 1565 GL2 POP 199 26.360 57.470 51.880 1.00 0.00

ATOM 1566 C1A POP 199 24.920 57.480 56.260 1.00 0.00

ATOM 1567 C2A POP 199 21.990 55.010 59.600 1.00 0.00

ATOM 1568 C3A POP 199 21.650 51.110 58.750 1.00 0.00

ATOM 1569 C4A POP 199 21.590 47.480 58.830 1.00 0.00

ATOM 1570 C1B POP 199 26.460 53.460 53.970 1.00 0.00

ATOM 1571 C2B POP 199 27.200 52.960 58.780 1.00 0.00

ATOM 1572 D3B POP 199 25.050 52.870 62.640 1.00 0.00

ATOM 1573 C4B POP 199 25.130 56.410 65.290 1.00 0.00

ATOM 1574 C5B POP 199 23.890 56.000 69.420 1.00 0.00

ATOM 1575 NC3 POP 200 80.100 89.490 46.030 1.00 0.00

ATOM 1576 PO4 POP 200 83.620 90.950 47.820 1.00 0.00

ATOM 1577 GL1 POP 200 85.990 92.270 51.420 1.00 0.00

ATOM 1578 GL2 POP 200 87.170 95.880 52.290 1.00 0.00

ATOM 1579 C1A POP 200 88.340 97.530 56.300 1.00 0.00

ATOM 1580 C2A POP 200 87.410 96.630 61.120 1.00 0.00

ATOM 1581 C3A POP 200 88.220 97.030 65.800 1.00 0.00

ATOM 1582 C4A POP 200 87.780 94.950 70.370 1.00 0.00

ATOM 1583 C1B POP 200 85.320 93.200 56.210 1.00 0.00

ATOM 1584 C2B POP 200 85.150 91.190 60.020 1.00 0.00

ATOM 1585 D3B POP 200 85.300 87.580 63.370 1.00 0.00

ATOM 1586 C4B POP 200 88.740 86.780 64.600 1.00 0.00

ATOM 1587 C5B POP 200 92.370 84.890 65.970 1.00 0.00

ATOM 1588 NC3 POP 201 26.830 83.980 44.760 1.00 0.00

ATOM 1589 PO4 POP 201 30.080 81.880 46.960 1.00 0.00

ATOM 1590 GL1 POP 201 28.560 79.280 50.330 1.00 0.00

ATOM 1591 GL2 POP 201 27.940 81.710 52.350 1.00 0.00

ATOM 1592 C1A POP 201 28.120 85.220 54.930 1.00 0.00

ATOM 1593 C2A POP 201 26.180 85.950 58.630 1.00 0.00

ATOM 1594 C3A POP 201 27.600 84.890 63.210 1.00 0.00

ATOM 1595 C4A POP 201 31.300 83.850 67.190 1.00 0.00

ATOM 1596 C1B POP 201 27.180 76.900 54.290 1.00 0.00

ATOM 1597 C2B POP 201 26.650 76.010 59.010 1.00 0.00

ATOM 1598 D3B POP 201 27.690 74.220 63.240 1.00 0.00

ATOM 1599 C4B POP 201 30.610 76.160 64.910 1.00 0.00

ATOM 1600 C5B POP 201 32.730 78.990 68.090 1.00 0.00

ATOM 1601 NC3 POP 202 72.890 79.990 90.160 1.00 0.00

ATOM 1602 PO4 POP 202 68.880 80.060 89.290 1.00 0.00

ATOM 1603 GL1 POP 202 70.560 83.300 86.420 1.00 0.00

ATOM 1604 GL2 POP 202 73.160 82.360 82.920 1.00 0.00

ATOM 1605 C1A POP 202 77.230 82.530 81.090 1.00 0.00

ATOM 1606 C2A POP 202 79.740 82.570 77.290 1.00 0.00

ATOM 1607 C3A POP 202 82.280 80.280 74.520 1.00 0.00

ATOM 1608 C4A POP 202 85.300 79.350 74.500 1.00 0.00

ATOM 1609 C1B POP 202 69.630 85.690 82.380 1.00 0.00

ATOM 1610 C2B POP 202 70.830 88.950 78.180 1.00 0.00

ATOM 1611 D3B POP 202 70.500 89.390 74.100 1.00 0.00

ATOM 1612 C4B POP 202 74.820 88.010 72.010 1.00 0.00

ATOM 1613 C5B POP 202 78.900 88.950 69.360 1.00 0.00

ATOM 1614 NC3 POP 203 49.880 93.980 85.790 1.00 0.00

ATOM 1615 PO4 POP 203 54.070 92.500 83.970 1.00 0.00

ATOM 1616 GL1 POP 203 54.590 95.080 80.450 1.00 0.00

ATOM 1617 GL2 POP 203 51.690 93.680 78.390 1.00 0.00

ATOM 1618 C1A POP 203 53.380 91.630 74.140 1.00 0.00

ATOM 1619 C2A POP 203 51.530 94.020 71.100 1.00 0.00

ATOM 1620 C3A POP 203 47.070 94.820 70.540 1.00 0.00

ATOM 1621 C4A POP 203 43.010 95.660 68.390 1.00 0.00

ATOM 1622 C1B POP 203 57.490 93.520 76.600 1.00 0.00

ATOM 1623 C2B POP 203 58.870 90.630 75.020 1.00 0.00

ATOM 1624 D3B POP 203 57.210 88.750 72.630 1.00 0.00

ATOM 1625 C4B POP 203 53.270 87.400 70.740 1.00 0.00

ATOM 1626 C5B POP 203 50.060 84.020 71.000 1.00 0.00

ATOM 1627 NC3 POP 204 96.530 90.530 89.570 1.00 0.00

ATOM 1628 PO4 POP 204 95.620 87.310 88.510 1.00 0.00

ATOM 1629 GL1 POP 204 94.950 89.290 84.580 1.00 0.00

ATOM 1630 GL2 POP 204 94.200 93.400 85.860 1.00 0.00

ATOM 1631 C1A POP 204 94.630 93.840 81.770 1.00 0.00

ATOM 1632 C2A POP 204 95.090 93.980 76.390 1.00 0.00

ATOM 1633 C3A POP 204 96.670 94.860 73.140 1.00 0.00

ATOM 1634 C4A POP 204 96.870 97.120 69.180 1.00 0.00

ATOM 1635 C1B POP 204 95.570 88.880 80.500 1.00 0.00

ATOM 1636 C2B POP 204 96.290 88.770 76.590 1.00 0.00

ATOM 1637 D3B POP 204 96.640 89.740 72.520 1.00 0.00

ATOM 1638 C4B POP 204 97.790 86.520 69.080 1.00 0.00

ATOM 1639 C5B POP 204 101.340 84.030 67.000 1.00 0.00

ATOM 1640 NC3 POP 205 47.140 45.820 85.720 1.00 0.00

ATOM 1641 PO4 POP 205 45.180 50.000 83.770 1.00 0.00

ATOM 1642 GL1 POP 205 46.680 51.210 80.190 1.00 0.00

ATOM 1643 GL2 POP 205 44.550 52.860 77.440 1.00 0.00

ATOM 1644 C1A POP 205 42.050 54.950 74.300 1.00 0.00

ATOM 1645 C2A POP 205 39.760 52.870 70.520 1.00 0.00

ATOM 1646 C3A POP 205 37.690 50.930 68.580 1.00 0.00

ATOM 1647 C4A POP 205 35.280 48.490 65.110 1.00 0.00

ATOM 1648 C1B POP 205 49.280 54.110 77.550 1.00 0.00

ATOM 1649 C2B POP 205 52.080 54.840 74.400 1.00 0.00

ATOM 1650 D3B POP 205 55.190 55.030 71.660 1.00 0.00

ATOM 1651 C4B POP 205 58.270 53.060 69.570 1.00 0.00

ATOM 1652 C5B POP 205 63.030 53.620 70.330 1.00 0.00

ATOM 1653 NC3 POP 206 27.850 103.240 87.270 1.00 0.00

ATOM 1654 PO4 POP 206 27.480 99.020 87.020 1.00 0.00

ATOM 1655 GL1 POP 206 27.490 98.320 82.270 1.00 0.00

ATOM 1656 GL2 POP 206 29.670 96.080 81.010 1.00 0.00

ATOM 1657 C1A POP 206 28.860 92.570 78.840 1.00 0.00

ATOM 1658 C2A POP 206 32.390 91.750 76.880 1.00 0.00

ATOM 1659 C3A POP 206 36.690 89.230 75.120 1.00 0.00

ATOM 1660 C4A POP 206 39.650 86.710 73.050 1.00 0.00

ATOM 1661 C1B POP 206 30.850 100.510 79.240 1.00 0.00

ATOM 1662 C2B POP 206 34.540 100.820 77.120 1.00 0.00

ATOM 1663 D3B POP 206 35.940 103.540 74.660 1.00 0.00

ATOM 1664 C4B POP 206 38.100 108.240 72.880 1.00 0.00

ATOM 1665 C5B POP 206 35.560 111.610 69.580 1.00 0.00

ATOM 1666 NC3 POP 207 50.920 42.330 46.980 1.00 0.00

ATOM 1667 PO4 POP 207 55.640 43.160 46.980 1.00 0.00

ATOM 1668 GL1 POP 207 58.460 42.000 50.470 1.00 0.00

ATOM 1669 GL2 POP 207 60.410 45.030 51.320 1.00 0.00

ATOM 1670 C1A POP 207 56.910 46.030 52.930 1.00 0.00

ATOM 1671 C2A POP 207 54.370 46.070 56.810 1.00 0.00

ATOM 1672 C3A POP 207 56.710 47.310 60.120 1.00 0.00

ATOM 1673 C4A POP 207 61.000 46.460 62.240 1.00 0.00

ATOM 1674 C1B POP 207 58.710 39.590 53.280 1.00 0.00

ATOM 1675 C2B POP 207 61.550 39.730 55.570 1.00 0.00

ATOM 1676 D3B POP 207 63.340 37.040 58.810 1.00 0.00

ATOM 1677 C4B POP 207 66.870 37.370 61.870 1.00 0.00

ATOM 1678 C5B POP 207 67.040 35.660 65.790 1.00 0.00

ATOM 1679 NC3 POP 208 60.510 73.460 44.340 1.00 0.00

ATOM 1680 PO4 POP 208 60.370 68.880 45.350 1.00 0.00

ATOM 1681 GL1 POP 208 59.980 67.070 49.400 1.00 0.00

ATOM 1682 GL2 POP 208 62.500 65.960 49.750 1.00 0.00

ATOM 1683 C1A POP 208 63.340 64.230 53.210 1.00 0.00

ATOM 1684 C2A POP 208 62.570 62.850 56.420 1.00 0.00

ATOM 1685 C3A POP 208 59.150 64.470 60.240 1.00 0.00

ATOM 1686 C4A POP 208 56.990 66.630 62.930 1.00 0.00

ATOM 1687 C1B POP 208 58.510 64.760 53.230 1.00 0.00

ATOM 1688 C2B POP 208 56.160 66.450 55.500 1.00 0.00

ATOM 1689 D3B POP 208 56.640 69.880 58.350 1.00 0.00

ATOM 1690 C4B POP 208 59.360 72.360 60.430 1.00 0.00

ATOM 1691 C5B POP 208 62.080 75.270 60.930 1.00 0.00

ATOM 1692 NC3 POP 209 1.110 58.110 45.650 1.00 0.00

ATOM 1693 PO4 POP 209 0.440 56.270 49.910 1.00 0.00

ATOM 1694 GL1 POP 209 2.750 52.240 51.990 1.00 0.00

ATOM 1695 GL2 POP 209 2.130 48.590 51.570 1.00 0.00

ATOM 1696 C1A POP 209 1.510 47.520 56.240 1.00 0.00

ATOM 1697 C2A POP 209 -0.540 50.150 59.130 1.00 0.00

ATOM 1698 C3A POP 209 0.430 51.840 62.960 1.00 0.00

ATOM 1699 C4A POP 209 3.020 51.170 66.840 1.00 0.00

ATOM 1700 C1B POP 209 4.980 51.760 56.260 1.00 0.00

ATOM 1701 C2B POP 209 4.180 52.880 60.240 1.00 0.00

ATOM 1702 D3B POP 209 6.860 50.840 63.670 1.00 0.00

ATOM 1703 C4B POP 209 8.720 53.390 66.690 1.00 0.00

ATOM 1704 C5B POP 209 7.090 56.080 69.390 1.00 0.00

ATOM 1705 NC3 POP 210 103.860 40.580 44.680 1.00 0.00

ATOM 1706 PO4 POP 210 104.960 37.620 47.670 1.00 0.00

ATOM 1707 GL1 POP 210 104.290 34.790 51.300 1.00 0.00

ATOM 1708 GL2 POP 210 103.010 32.290 52.740 1.00 0.00

ATOM 1709 C1A POP 210 101.640 30.880 57.220 1.00 0.00

ATOM 1710 C2A POP 210 101.960 31.890 62.330 1.00 0.00

ATOM 1711 C3A POP 210 103.960 35.670 64.760 1.00 0.00

ATOM 1712 C4A POP 210 100.790 38.870 64.060 1.00 0.00

ATOM 1713 C1B POP 210 107.630 32.840 54.510 1.00 0.00

ATOM 1714 C2B POP 210 109.080 28.730 56.710 1.00 0.00

ATOM 1715 D3B POP 210 108.680 24.960 59.500 1.00 0.00

ATOM 1716 C4B POP 210 108.320 26.190 62.940 1.00 0.00

ATOM 1717 C5B POP 210 109.230 30.170 65.450 1.00 0.00

ATOM 1718 NC3 POP 211 70.350 11.430 38.900 1.00 0.00

ATOM 1719 PO4 POP 211 70.090 14.990 42.060 1.00 0.00

ATOM 1720 GL1 POP 211 68.550 15.230 46.330 1.00 0.00

ATOM 1721 GL2 POP 211 65.570 12.840 47.980 1.00 0.00

ATOM 1722 C1A POP 211 65.300 14.690 51.840 1.00 0.00

ATOM 1723 C2A POP 211 67.190 15.580 55.980 1.00 0.00

ATOM 1724 C3A POP 211 69.310 15.420 58.720 1.00 0.00

ATOM 1725 C4A POP 211 73.020 14.000 61.760 1.00 0.00

ATOM 1726 C1B POP 211 69.680 14.770 50.290 1.00 0.00

ATOM 1727 C2B POP 211 71.940 15.860 54.630 1.00 0.00

ATOM 1728 D3B POP 211 73.820 17.510 58.170 1.00 0.00

ATOM 1729 C4B POP 211 71.690 20.680 60.790 1.00 0.00

ATOM 1730 C5B POP 211 70.750 19.580 64.350 1.00 0.00

ATOM 1731 NC3 POP 212 31.370 40.200 45.270 1.00 0.00

ATOM 1732 PO4 POP 212 29.970 35.370 44.020 1.00 0.00

ATOM 1733 GL1 POP 212 31.130 34.130 47.560 1.00 0.00

ATOM 1734 GL2 POP 212 27.270 33.280 49.580 1.00 0.00

ATOM 1735 C1A POP 212 27.860 34.600 54.400 1.00 0.00

ATOM 1736 C2A POP 212 29.290 33.380 57.520 1.00 0.00

ATOM 1737 C3A POP 212 28.850 31.490 61.190 1.00 0.00

ATOM 1738 C4A POP 212 27.580 26.520 62.770 1.00 0.00

ATOM 1739 C1B POP 212 31.870 31.890 52.060 1.00 0.00

ATOM 1740 C2B POP 212 34.010 31.040 55.510 1.00 0.00

ATOM 1741 D3B POP 212 33.590 28.150 59.020 1.00 0.00

ATOM 1742 C4B POP 212 33.370 30.410 63.030 1.00 0.00

ATOM 1743 C5B POP 212 29.650 30.500 66.630 1.00 0.00

ATOM 1744 NC3 POP 213 96.040 49.940 45.670 1.00 0.00

ATOM 1745 PO4 POP 213 96.210 46.170 47.580 1.00 0.00

ATOM 1746 GL1 POP 213 95.960 49.610 50.880 1.00 0.00

ATOM 1747 GL2 POP 213 97.580 53.100 49.800 1.00 0.00

ATOM 1748 C1A POP 213 98.570 51.850 54.180 1.00 0.00

ATOM 1749 C2A POP 213 99.510 51.100 58.660 1.00 0.00

ATOM 1750 C3A POP 213 103.200 50.470 61.130 1.00 0.00

ATOM 1751 C4A POP 213 105.570 53.560 61.450 1.00 0.00

ATOM 1752 C1B POP 213 94.580 48.280 55.120 1.00 0.00

ATOM 1753 C2B POP 213 95.760 46.780 60.360 1.00 0.00

ATOM 1754 D3B POP 213 97.980 48.870 63.160 1.00 0.00

ATOM 1755 C4B POP 213 96.210 48.090 66.640 1.00 0.00

ATOM 1756 C5B POP 213 96.930 46.390 71.450 1.00 0.00

ATOM 1757 NC3 POP 214 58.900 95.840 38.480 1.00 0.00

ATOM 1758 PO4 POP 214 58.620 94.970 42.810 1.00 0.00

ATOM 1759 GL1 POP 214 55.750 94.310 46.440 1.00 0.00

ATOM 1760 GL2 POP 214 55.230 90.900 47.010 1.00 0.00

ATOM 1761 C1A POP 214 59.320 92.510 49.280 1.00 0.00

ATOM 1762 C2A POP 214 58.750 90.650 53.990 1.00 0.00

ATOM 1763 C3A POP 214 55.870 91.970 57.450 1.00 0.00

ATOM 1764 C4A POP 214 53.670 95.950 57.400 1.00 0.00

ATOM 1765 C1B POP 214 54.640 93.070 50.840 1.00 0.00

ATOM 1766 C2B POP 214 52.450 89.800 53.520 1.00 0.00

ATOM 1767 D3B POP 214 55.140 86.230 54.690 1.00 0.00

ATOM 1768 C4B POP 214 54.710 86.140 58.800 1.00 0.00

ATOM 1769 C5B POP 214 55.400 87.740 62.830 1.00 0.00

ATOM 1770 NC3 POP 215 98.910 33.080 88.990 1.00 0.00

ATOM 1771 PO4 POP 215 95.410 36.370 89.030 1.00 0.00

ATOM 1772 GL1 POP 215 94.250 38.540 85.350 1.00 0.00

ATOM 1773 GL2 POP 215 97.130 41.770 84.600 1.00 0.00

ATOM 1774 C1A POP 215 96.830 45.060 80.870 1.00 0.00

ATOM 1775 C2A POP 215 99.520 45.890 76.860 1.00 0.00

ATOM 1776 C3A POP 215 102.420 44.620 74.380 1.00 0.00

ATOM 1777 C4A POP 215 102.020 43.750 69.620 1.00 0.00

ATOM 1778 C1B POP 215 95.380 36.970 80.700 1.00 0.00

ATOM 1779 C2B POP 215 97.290 37.320 77.320 1.00 0.00

ATOM 1780 D3B POP 215 95.270 37.450 73.980 1.00 0.00

ATOM 1781 C4B POP 215 93.440 40.370 72.410 1.00 0.00

ATOM 1782 C5B POP 215 93.880 43.610 68.800 1.00 0.00

ATOM 1783 NC3 POP 216 25.910 40.000 84.680 1.00 0.00

ATOM 1784 PO4 POP 216 26.480 36.420 84.400 1.00 0.00

ATOM 1785 GL1 POP 216 25.380 36.040 80.940 1.00 0.00

ATOM 1786 GL2 POP 216 26.230 33.790 78.810 1.00 0.00

ATOM 1787 C1A POP 216 27.800 31.260 76.220 1.00 0.00

ATOM 1788 C2A POP 216 27.300 31.080 71.950 1.00 0.00

ATOM 1789 C3A POP 216 24.600 30.580 68.730 1.00 0.00

ATOM 1790 C4A POP 216 24.710 30.420 63.770 1.00 0.00

ATOM 1791 C1B POP 216 27.120 38.880 77.410 1.00 0.00

ATOM 1792 C2B POP 216 28.940 42.300 75.500 1.00 0.00

ATOM 1793 D3B POP 216 32.370 43.490 75.270 1.00 0.00

ATOM 1794 C4B POP 216 34.140 45.150 79.310 1.00 0.00

ATOM 1795 C5B POP 216 35.430 47.140 83.110 1.00 0.00

ATOM 1796 NC3 POP 217 47.130 37.820 87.360 1.00 0.00

ATOM 1797 PO4 POP 217 50.720 38.040 85.480 1.00 0.00

ATOM 1798 GL1 POP 217 48.650 36.430 81.790 1.00 0.00

ATOM 1799 GL2 POP 217 50.210 34.180 81.960 1.00 0.00

ATOM 1800 C1A POP 217 49.760 35.120 76.970 1.00 0.00

ATOM 1801 C2A POP 217 48.840 34.700 72.230 1.00 0.00

ATOM 1802 C3A POP 217 49.030 34.270 68.210 1.00 0.00

ATOM 1803 C4A POP 217 48.350 36.160 64.650 1.00 0.00

ATOM 1804 C1B POP 217 47.560 39.090 78.610 1.00 0.00

ATOM 1805 C2B POP 217 46.030 42.780 75.530 1.00 0.00

ATOM 1806 D3B POP 217 44.760 45.140 72.510 1.00 0.00

ATOM 1807 C4B POP 217 44.340 42.800 69.020 1.00 0.00

ATOM 1808 C5B POP 217 44.670 41.050 65.170 1.00 0.00

ATOM 1809 NC3 POP 218 59.240 46.050 46.270 1.00 0.00

ATOM 1810 PO4 POP 218 63.180 47.820 44.330 1.00 0.00

ATOM 1811 GL1 POP 218 63.950 47.160 48.740 1.00 0.00

ATOM 1812 GL2 POP 218 63.110 49.370 50.700 1.00 0.00

ATOM 1813 C1A POP 218 60.790 49.890 53.440 1.00 0.00

ATOM 1814 C2A POP 218 60.300 48.610 56.780 1.00 0.00

ATOM 1815 C3A POP 218 59.640 44.270 57.080 1.00 0.00

ATOM 1816 C4A POP 218 57.210 40.580 58.800 1.00 0.00

ATOM 1817 C1B POP 218 65.590 45.380 53.200 1.00 0.00

ATOM 1818 C2B POP 218 64.770 46.220 57.050 1.00 0.00

ATOM 1819 D3B POP 218 67.280 46.120 61.110 1.00 0.00

ATOM 1820 C4B POP 218 65.040 44.980 64.670 1.00 0.00

ATOM 1821 C5B POP 218 63.250 44.100 69.340 1.00 0.00

ATOM 1822 NC3 POP 219 16.320 69.610 43.930 1.00 0.00

ATOM 1823 PO4 POP 219 14.770 66.750 47.920 1.00 0.00

ATOM 1824 GL1 POP 219 14.460 65.300 52.910 1.00 0.00

ATOM 1825 GL2 POP 219 11.210 65.020 52.620 1.00 0.00

ATOM 1826 C1A POP 219 8.580 65.500 55.600 1.00 0.00

ATOM 1827 C2A POP 219 5.520 64.800 59.160 1.00 0.00

ATOM 1828 C3A POP 219 5.270 66.160 62.780 1.00 0.00

ATOM 1829 C4A POP 219 3.720 69.550 65.870 1.00 0.00

ATOM 1830 C1B POP 219 13.110 64.490 57.600 1.00 0.00

ATOM 1831 C2B POP 219 15.270 63.260 60.650 1.00 0.00

ATOM 1832 D3B POP 219 14.290 65.710 65.130 1.00 0.00

ATOM 1833 C4B POP 219 14.680 62.200 68.080 1.00 0.00

ATOM 1834 C5B POP 219 17.090 60.500 70.840 1.00 0.00

ATOM 1835 NC3 POP 220 66.760 95.330 44.260 1.00 0.00

ATOM 1836 PO4 POP 220 63.380 97.680 44.190 1.00 0.00

ATOM 1837 GL1 POP 220 63.390 96.650 48.300 1.00 0.00

ATOM 1838 GL2 POP 220 65.710 94.490 49.490 1.00 0.00

ATOM 1839 C1A POP 220 63.660 92.160 52.930 1.00 0.00

ATOM 1840 C2A POP 220 64.300 87.800 54.150 1.00 0.00

ATOM 1841 C3A POP 220 62.810 83.200 54.540 1.00 0.00

ATOM 1842 C4A POP 220 60.280 81.060 51.460 1.00 0.00

ATOM 1843 C1B POP 220 60.970 96.980 52.390 1.00 0.00

ATOM 1844 C2B POP 220 58.240 96.950 56.150 1.00 0.00

ATOM 1845 D3B POP 220 58.150 96.630 60.620 1.00 0.00

ATOM 1846 C4B POP 220 55.130 97.240 63.380 1.00 0.00

ATOM 1847 C5B POP 220 52.480 95.610 66.400 1.00 0.00

ATOM 1848 NC3 POP 221 42.390 88.500 44.260 1.00 0.00

ATOM 1849 PO4 POP 221 37.880 87.970 45.970 1.00 0.00

ATOM 1850 GL1 POP 221 37.170 88.810 50.010 1.00 0.00

ATOM 1851 GL2 POP 221 38.520 89.280 53.210 1.00 0.00

ATOM 1852 C1A POP 221 37.110 88.480 56.200 1.00 0.00

ATOM 1853 C2A POP 221 35.760 88.360 59.900 1.00 0.00

ATOM 1854 C3A POP 221 37.590 90.280 63.860 1.00 0.00

ATOM 1855 C4A POP 221 41.150 92.030 65.260 1.00 0.00

ATOM 1856 C1B POP 221 33.740 89.780 52.050 1.00 0.00

ATOM 1857 C2B POP 221 31.430 88.610 55.120 1.00 0.00

ATOM 1858 D3B POP 221 31.000 87.600 58.690 1.00 0.00

ATOM 1859 C4B POP 221 31.980 91.950 61.370 1.00 0.00

ATOM 1860 C5B POP 221 32.300 93.080 64.680 1.00 0.00

ATOM 1861 NC3 POP 222 46.060 84.790 47.130 1.00 0.00

ATOM 1862 PO4 POP 222 45.380 89.610 48.580 1.00 0.00

ATOM 1863 GL1 POP 222 46.330 88.690 51.740 1.00 0.00

ATOM 1864 GL2 POP 222 43.210 90.340 52.780 1.00 0.00

ATOM 1865 C1A POP 222 41.830 90.150 56.270 1.00 0.00

ATOM 1866 C2A POP 222 40.970 89.280 60.410 1.00 0.00

ATOM 1867 C3A POP 222 43.210 87.180 63.630 1.00 0.00

ATOM 1868 C4A POP 222 44.660 89.370 67.820 1.00 0.00

ATOM 1869 C1B POP 222 45.880 87.250 55.820 1.00 0.00

ATOM 1870 C2B POP 222 46.060 89.380 59.390 1.00 0.00

ATOM 1871 D3B POP 222 45.950 90.760 62.580 1.00 0.00

ATOM 1872 C4B POP 222 48.700 92.810 65.600 1.00 0.00

ATOM 1873 C5B POP 222 51.850 89.990 66.050 1.00 0.00

ATOM 1874 NC3 POP 223 22.050 75.870 42.730 1.00 0.00

ATOM 1875 PO4 POP 223 23.680 79.730 43.030 1.00 0.00

ATOM 1876 GL1 POP 223 24.640 80.680 47.760 1.00 0.00

ATOM 1877 GL2 POP 223 24.400 83.800 48.800 1.00 0.00

ATOM 1878 C1A POP 223 23.460 84.120 53.240 1.00 0.00

ATOM 1879 C2A POP 223 24.950 80.850 56.020 1.00 0.00

ATOM 1880 C3A POP 223 23.860 80.550 59.660 1.00 0.00

ATOM 1881 C4A POP 223 23.220 77.740 62.890 1.00 0.00

ATOM 1882 C1B POP 223 23.530 78.050 51.720 1.00 0.00

ATOM 1883 C2B POP 223 22.030 76.830 57.090 1.00 0.00

ATOM 1884 D3B POP 223 19.040 77.150 59.140 1.00 0.00

ATOM 1885 C4B POP 223 15.200 78.420 57.580 1.00 0.00

ATOM 1886 C5B POP 223 11.410 79.050 55.160 1.00 0.00

ATOM 1887 NC3 POP 224 57.530 84.230 87.740 1.00 0.00

ATOM 1888 PO4 POP 224 53.530 82.250 85.850 1.00 0.00

ATOM 1889 GL1 POP 224 56.570 80.710 83.130 1.00 0.00

ATOM 1890 GL2 POP 224 56.590 84.120 81.660 1.00 0.00

ATOM 1891 C1A POP 224 51.950 81.750 79.460 1.00 0.00

ATOM 1892 C2A POP 224 49.050 82.790 76.410 1.00 0.00

ATOM 1893 C3A POP 224 44.670 82.100 74.030 1.00 0.00

ATOM 1894 C4A POP 224 40.970 82.400 70.950 1.00 0.00

ATOM 1895 C1B POP 224 55.890 79.140 78.930 1.00 0.00

ATOM 1896 C2B POP 224 56.430 78.450 74.130 1.00 0.00

ATOM 1897 D3B POP 224 54.440 77.580 70.630 1.00 0.00

ATOM 1898 C4B POP 224 55.870 81.360 69.270 1.00 0.00

ATOM 1899 C5B POP 224 57.480 83.940 71.960 1.00 0.00

ATOM 1900 NC3 POP 225 46.180 33.860 42.680 1.00 0.00

ATOM 1901 PO4 POP 225 50.480 32.820 42.720 1.00 0.00

ATOM 1902 GL1 POP 225 51.610 31.810 47.210 1.00 0.00

ATOM 1903 GL2 POP 225 54.750 32.990 48.910 1.00 0.00

ATOM 1904 C1A POP 225 58.440 34.810 50.540 1.00 0.00

ATOM 1905 C2A POP 225 60.470 33.690 53.440 1.00 0.00

ATOM 1906 C3A POP 225 61.850 32.080 56.460 1.00 0.00

ATOM 1907 C4A POP 225 62.820 28.480 57.820 1.00 0.00

ATOM 1908 C1B POP 225 50.980 33.440 51.720 1.00 0.00

ATOM 1909 C2B POP 225 48.990 36.670 53.300 1.00 0.00

ATOM 1910 D3B POP 225 45.480 39.280 54.080 1.00 0.00

ATOM 1911 C4B POP 225 43.780 41.340 57.280 1.00 0.00

ATOM 1912 C5B POP 225 42.070 42.130 60.940 1.00 0.00

ATOM 1913 NC3 POP 226 31.310 33.250 82.970 1.00 0.00

ATOM 1914 PO4 POP 226 32.980 30.410 79.570 1.00 0.00

ATOM 1915 GL1 POP 226 33.020 32.000 76.480 1.00 0.00

ATOM 1916 GL2 POP 226 34.630 34.220 76.890 1.00 0.00

ATOM 1917 C1A POP 226 36.820 30.840 73.140 1.00 0.00

ATOM 1918 C2A POP 226 40.590 30.410 70.390 1.00 0.00

ATOM 1919 C3A POP 226 42.760 27.200 67.960 1.00 0.00

ATOM 1920 C4A POP 226 43.110 26.900 64.130 1.00 0.00

ATOM 1921 C1B POP 226 31.970 33.540 72.630 1.00 0.00

ATOM 1922 C2B POP 226 29.830 35.190 68.170 1.00 0.00

ATOM 1923 D3B POP 226 31.520 39.280 65.740 1.00 0.00

ATOM 1924 C4B POP 226 34.470 39.370 61.850 1.00 0.00

ATOM 1925 C5B POP 226 37.840 40.800 60.940 1.00 0.00

ATOM 1926 NC3 POP 227 85.200 29.660 90.370 1.00 0.00

ATOM 1927 PO4 POP 227 81.890 30.970 86.550 1.00 0.00

ATOM 1928 GL1 POP 227 84.610 32.610 83.150 1.00 0.00

ATOM 1929 GL2 POP 227 87.130 35.080 82.990 1.00 0.00

ATOM 1930 C1A POP 227 87.030 37.230 79.230 1.00 0.00

ATOM 1931 C2A POP 227 87.020 40.510 77.640 1.00 0.00

ATOM 1932 C3A POP 227 89.710 45.170 79.090 1.00 0.00

ATOM 1933 C4A POP 227 88.220 48.930 80.000 1.00 0.00

ATOM 1934 C1B POP 227 86.950 32.200 79.100 1.00 0.00

ATOM 1935 C2B POP 227 87.900 32.100 74.960 1.00 0.00

ATOM 1936 D3B POP 227 87.840 32.510 70.240 1.00 0.00

ATOM 1937 C4B POP 227 87.740 29.210 66.830 1.00 0.00

ATOM 1938 C5B POP 227 86.260 24.990 66.680 1.00 0.00

ATOM 1939 NC3 POP 228 61.910 34.040 90.850 1.00 0.00

ATOM 1940 PO4 POP 228 65.020 31.850 90.330 1.00 0.00

ATOM 1941 GL1 POP 228 66.160 31.910 86.250 1.00 0.00

ATOM 1942 GL2 POP 228 64.010 34.880 85.870 1.00 0.00

ATOM 1943 C1A POP 228 63.340 37.650 82.010 1.00 0.00

ATOM 1944 C2A POP 228 64.060 40.110 78.230 1.00 0.00

ATOM 1945 C3A POP 228 65.210 40.100 74.250 1.00 0.00

ATOM 1946 C4A POP 228 63.320 38.320 69.910 1.00 0.00

ATOM 1947 C1B POP 228 66.340 32.040 80.950 1.00 0.00

ATOM 1948 C2B POP 228 63.920 33.180 77.390 1.00 0.00

ATOM 1949 D3B POP 228 63.680 35.520 74.800 1.00 0.00

ATOM 1950 C4B POP 228 60.660 38.920 74.010 1.00 0.00

ATOM 1951 C5B POP 228 60.540 43.510 74.660 1.00 0.00

ATOM 1952 NC3 POP 229 104.890 84.220 41.400 1.00 0.00

ATOM 1953 PO4 POP 229 105.790 87.040 45.160 1.00 0.00

ATOM 1954 GL1 POP 229 106.910 85.000 50.010 1.00 0.00

ATOM 1955 GL2 POP 229 105.310 87.260 52.470 1.00 0.00

ATOM 1956 C1A POP 229 109.780 86.900 53.760 1.00 0.00

ATOM 1957 C2A POP 229 113.030 88.850 55.850 1.00 0.00

ATOM 1958 C3A POP 229 116.070 89.420 59.340 1.00 0.00

ATOM 1959 C4A POP 229 116.990 91.370 63.860 1.00 0.00

ATOM 1960 C1B POP 229 106.420 82.360 53.780 1.00 0.00

ATOM 1961 C2B POP 229 105.400 83.280 57.530 1.00 0.00

ATOM 1962 D3B POP 229 105.060 83.250 62.860 1.00 0.00

ATOM 1963 C4B POP 229 105.370 80.570 65.440 1.00 0.00

ATOM 1964 C5B POP 229 103.450 76.340 64.540 1.00 0.00

ATOM 1965 NC3 POP 230 106.170 13.850 41.890 1.00 0.00

ATOM 1966 PO4 POP 230 106.420 12.890 46.810 1.00 0.00

ATOM 1967 GL1 POP 230 104.430 16.230 49.850 1.00 0.00

ATOM 1968 GL2 POP 230 102.640 14.610 52.470 1.00 0.00

ATOM 1969 C1A POP 230 105.070 13.510 56.020 1.00 0.00

ATOM 1970 C2A POP 230 104.860 14.870 59.890 1.00 0.00

ATOM 1971 C3A POP 230 106.480 16.580 63.960 1.00 0.00

ATOM 1972 C4A POP 230 109.060 16.690 67.370 1.00 0.00

ATOM 1973 C1B POP 230 102.990 19.240 53.690 1.00 0.00

ATOM 1974 C2B POP 230 101.640 21.820 57.640 1.00 0.00

ATOM 1975 D3B POP 230 100.100 23.800 62.260 1.00 0.00

ATOM 1976 C4B POP 230 103.750 25.690 63.950 1.00 0.00

ATOM 1977 C5B POP 230 104.430 28.490 66.510 1.00 0.00

ATOM 1978 NC3 POP 231 18.930 96.390 85.520 1.00 0.00

ATOM 1979 PO4 POP 231 23.070 98.290 84.110 1.00 0.00

ATOM 1980 GL1 POP 231 23.600 101.660 81.910 1.00 0.00

ATOM 1981 GL2 POP 231 22.260 100.280 79.810 1.00 0.00

ATOM 1982 C1A POP 231 20.720 100.150 76.000 1.00 0.00

ATOM 1983 C2A POP 231 19.390 101.920 72.730 1.00 0.00

ATOM 1984 C3A POP 231 19.990 105.040 69.320 1.00 0.00

ATOM 1985 C4A POP 231 23.160 107.490 66.150 1.00 0.00

ATOM 1986 C1B POP 231 25.230 104.160 77.840 1.00 0.00

ATOM 1987 C2B POP 231 26.160 105.190 73.460 1.00 0.00

ATOM 1988 D3B POP 231 27.140 106.490 69.770 1.00 0.00

ATOM 1989 C4B POP 231 31.770 105.360 69.330 1.00 0.00

ATOM 1990 C5B POP 231 36.440 105.460 69.730 1.00 0.00

ATOM 1991 NC3 POP 232 16.310 106.120 37.880 1.00 0.00

ATOM 1992 PO4 POP 232 18.400 108.960 41.630 1.00 0.00

ATOM 1993 GL1 POP 232 18.210 108.300 47.000 1.00 0.00

ATOM 1994 GL2 POP 232 19.740 106.380 48.980 1.00 0.00

ATOM 1995 C1A POP 232 17.930 107.000 52.470 1.00 0.00

ATOM 1996 C2A POP 232 15.240 106.230 55.010 1.00 0.00

ATOM 1997 C3A POP 232 11.660 104.660 56.760 1.00 0.00

ATOM 1998 C4A POP 232 7.760 103.040 54.140 1.00 0.00

ATOM 1999 C1B POP 232 16.850 111.770 48.820 1.00 0.00

ATOM 2000 C2B POP 232 18.600 112.550 51.860 1.00 0.00

ATOM 2001 D3B POP 232 20.290 111.700 55.530 1.00 0.00

ATOM 2002 C4B POP 232 20.540 107.710 56.890 1.00 0.00

ATOM 2003 C5B POP 232 24.280 105.450 58.460 1.00 0.00

ATOM 2004 NC3 POP 233 98.890 80.740 92.650 1.00 0.00

ATOM 2005 PO4 POP 233 100.630 78.880 89.160 1.00 0.00

ATOM 2006 GL1 POP 233 100.380 80.550 85.990 1.00 0.00

ATOM 2007 GL2 POP 233 103.650 81.220 84.610 1.00 0.00

ATOM 2008 C1A POP 233 104.080 83.830 80.690 1.00 0.00

ATOM 2009 C2A POP 233 104.560 83.780 75.830 1.00 0.00

ATOM 2010 C3A POP 233 108.560 83.520 72.730 1.00 0.00

ATOM 2011 C4A POP 233 109.040 80.910 69.090 1.00 0.00

ATOM 2012 C1B POP 233 100.540 78.510 81.820 1.00 0.00

ATOM 2013 C2B POP 233 100.630 80.090 77.540 1.00 0.00

ATOM 2014 D3B POP 233 99.660 80.720 73.750 1.00 0.00

ATOM 2015 C4B POP 233 103.700 81.360 70.900 1.00 0.00

ATOM 2016 C5B POP 233 105.460 85.680 69.080 1.00 0.00

ATOM 2017 NC3 POP 234 44.790 58.240 45.210 1.00 0.00

ATOM 2018 PO4 POP 234 47.150 61.820 44.210 1.00 0.00

ATOM 2019 GL1 POP 234 47.570 61.760 48.650 1.00 0.00

ATOM 2020 GL2 POP 234 44.700 60.690 50.570 1.00 0.00

ATOM 2021 C1A POP 234 43.940 60.770 54.340 1.00 0.00

ATOM 2022 C2A POP 234 46.460 60.700 57.600 1.00 0.00

ATOM 2023 C3A POP 234 48.100 63.360 61.070 1.00 0.00

ATOM 2024 C4A POP 234 49.770 62.880 64.580 1.00 0.00

ATOM 2025 C1B POP 234 49.800 60.040 52.610 1.00 0.00

ATOM 2026 C2B POP 234 51.520 60.160 57.600 1.00 0.00

ATOM 2027 D3B POP 234 53.250 58.830 62.530 1.00 0.00

ATOM 2028 C4B POP 234 54.860 62.370 64.810 1.00 0.00

ATOM 2029 C5B POP 234 53.590 66.500 66.730 1.00 0.00

ATOM 2030 NC3 POP 235 115.740 35.890 86.100 1.00 0.00

ATOM 2031 PO4 POP 235 115.440 40.690 85.900 1.00 0.00

ATOM 2032 GL1 POP 235 111.140 40.800 84.310 1.00 0.00

ATOM 2033 GL2 POP 235 108.400 38.790 84.340 1.00 0.00

ATOM 2034 C1A POP 235 105.610 40.230 80.440 1.00 0.00

ATOM 2035 C2A POP 235 104.780 39.700 76.690 1.00 0.00

ATOM 2036 C3A POP 235 101.540 39.180 74.190 1.00 0.00

ATOM 2037 C4A POP 235 98.270 41.770 73.070 1.00 0.00

ATOM 2038 C1B POP 235 110.740 42.020 80.390 1.00 0.00

ATOM 2039 C2B POP 235 108.840 44.760 76.510 1.00 0.00

ATOM 2040 D3B POP 235 106.360 44.580 71.830 1.00 0.00

ATOM 2041 C4B POP 235 106.400 47.920 69.310 1.00 0.00

ATOM 2042 C5B POP 235 105.930 51.140 66.350 1.00 0.00

ATOM 2043 NC3 POP 236 17.590 46.520 45.680 1.00 0.00

ATOM 2044 PO4 POP 236 15.960 49.180 48.080 1.00 0.00

ATOM 2045 GL1 POP 236 15.750 46.340 52.040 1.00 0.00

ATOM 2046 GL2 POP 236 18.630 45.770 54.400 1.00 0.00

ATOM 2047 C1A POP 236 17.190 45.570 58.730 1.00 0.00

ATOM 2048 C2A POP 236 20.620 44.740 62.270 1.00 0.00

ATOM 2049 C3A POP 236 22.590 47.160 63.940 1.00 0.00

ATOM 2050 C4A POP 236 26.140 48.270 61.190 1.00 0.00

ATOM 2051 C1B POP 236 12.310 47.020 55.730 1.00 0.00

ATOM 2052 C2B POP 236 11.030 44.940 59.870 1.00 0.00

ATOM 2053 D3B POP 236 8.010 43.850 63.250 1.00 0.00

ATOM 2054 C4B POP 236 5.620 45.900 66.860 1.00 0.00

ATOM 2055 C5B POP 236 6.790 49.310 68.840 1.00 0.00

ATOM 2056 NC3 POP 237 23.740 45.390 94.230 1.00 0.00

ATOM 2057 PO4 POP 237 23.810 47.050 90.280 1.00 0.00

ATOM 2058 GL1 POP 237 24.090 46.980 86.280 1.00 0.00

ATOM 2059 GL2 POP 237 25.330 45.540 84.740 1.00 0.00

ATOM 2060 C1A POP 237 23.720 44.670 81.160 1.00 0.00

ATOM 2061 C2A POP 237 23.760 43.340 77.370 1.00 0.00

ATOM 2062 C3A POP 237 21.960 40.030 75.950 1.00 0.00

ATOM 2063 C4A POP 237 19.110 37.820 74.280 1.00 0.00

ATOM 2064 C1B POP 237 26.260 49.510 82.650 1.00 0.00

ATOM 2065 C2B POP 237 30.870 49.940 80.490 1.00 0.00

ATOM 2066 D3B POP 237 33.460 51.280 76.330 1.00 0.00

ATOM 2067 C4B POP 237 36.480 48.630 75.890 1.00 0.00

ATOM 2068 C5B POP 237 40.190 48.180 80.180 1.00 0.00

ATOM 2069 NC3 POP 238 89.120 15.930 39.730 1.00 0.00

ATOM 2070 PO4 POP 238 87.190 14.960 43.640 1.00 0.00

ATOM 2071 GL1 POP 238 87.380 12.950 46.750 1.00 0.00

ATOM 2072 GL2 POP 238 85.830 9.850 48.190 1.00 0.00

ATOM 2073 C1A POP 238 82.080 8.760 49.550 1.00 0.00

ATOM 2074 C2A POP 238 80.410 6.410 53.750 1.00 0.00

ATOM 2075 C3A POP 238 78.710 4.900 57.360 1.00 0.00

ATOM 2076 C4A POP 238 75.640 2.370 58.520 1.00 0.00

ATOM 2077 C1B POP 238 86.780 13.760 51.070 1.00 0.00

ATOM 2078 C2B POP 238 87.130 15.250 54.780 1.00 0.00

ATOM 2079 D3B POP 238 88.490 13.290 58.550 1.00 0.00

ATOM 2080 C4B POP 238 86.280 13.720 63.310 1.00 0.00

ATOM 2081 C5B POP 238 87.460 12.620 66.990 1.00 0.00

ATOM 2082 NC3 POP 239 59.170 67.680 87.540 1.00 0.00

ATOM 2083 PO4 POP 239 61.690 70.840 88.250 1.00 0.00

ATOM 2084 GL1 POP 239 61.110 72.430 85.050 1.00 0.00

ATOM 2085 GL2 POP 239 57.350 71.450 84.820 1.00 0.00

ATOM 2086 C1A POP 239 56.920 69.400 79.720 1.00 0.00

ATOM 2087 C2A POP 239 57.420 68.850 75.900 1.00 0.00

ATOM 2088 C3A POP 239 60.180 65.870 73.780 1.00 0.00

ATOM 2089 C4A POP 239 62.730 61.710 74.020 1.00 0.00

ATOM 2090 C1B POP 239 60.640 72.430 80.670 1.00 0.00

ATOM 2091 C2B POP 239 61.640 71.030 77.580 1.00 0.00

ATOM 2092 D3B POP 239 60.740 71.010 73.320 1.00 0.00

ATOM 2093 C4B POP 239 62.800 70.080 69.370 1.00 0.00

ATOM 2094 C5B POP 239 62.440 66.120 67.570 1.00 0.00

ATOM 2095 NC3 POP 240 82.660 83.060 45.120 1.00 0.00

ATOM 2096 PO4 POP 240 79.100 83.710 46.120 1.00 0.00

ATOM 2097 GL1 POP 240 81.460 80.300 49.470 1.00 0.00

ATOM 2098 GL2 POP 240 83.460 77.270 48.460 1.00 0.00

ATOM 2099 C1A POP 240 83.010 76.730 52.730 1.00 0.00

ATOM 2100 C2A POP 240 84.600 76.530 57.770 1.00 0.00

ATOM 2101 C3A POP 240 84.080 72.040 59.540 1.00 0.00

ATOM 2102 C4A POP 240 84.410 68.330 56.970 1.00 0.00

ATOM 2103 C1B POP 240 80.480 81.450 53.590 1.00 0.00

ATOM 2104 C2B POP 240 82.690 82.340 57.460 1.00 0.00

ATOM 2105 D3B POP 240 81.040 79.800 60.210 1.00 0.00

ATOM 2106 C4B POP 240 84.060 80.410 62.170 1.00 0.00

ATOM 2107 C5B POP 240 87.580 77.330 61.170 1.00 0.00

ATOM 2108 NC3 POP 241 77.840 103.210 43.510 1.00 0.00

ATOM 2109 PO4 POP 241 73.550 103.190 43.780 1.00 0.00

ATOM 2110 GL1 POP 241 71.600 102.180 47.320 1.00 0.00

ATOM 2111 GL2 POP 241 73.130 100.700 50.330 1.00 0.00

ATOM 2112 C1A POP 241 69.350 100.620 54.870 1.00 0.00

ATOM 2113 C2A POP 241 69.630 102.030 58.250 1.00 0.00

ATOM 2114 C3A POP 241 74.250 101.850 59.990 1.00 0.00

ATOM 2115 C4A POP 241 77.970 101.390 62.350 1.00 0.00

ATOM 2116 C1B POP 241 70.000 104.360 49.900 1.00 0.00

ATOM 2117 C2B POP 241 68.190 105.450 54.750 1.00 0.00

ATOM 2118 D3B POP 241 66.350 105.760 58.870 1.00 0.00

ATOM 2119 C4B POP 241 64.260 102.730 61.000 1.00 0.00

ATOM 2120 C5B POP 241 64.470 100.930 64.980 1.00 0.00

ATOM 2121 NC3 POP 242 26.260 43.920 42.780 1.00 0.00

ATOM 2122 PO4 POP 242 27.220 40.210 41.930 1.00 0.00

ATOM 2123 GL1 POP 242 26.190 40.770 46.770 1.00 0.00

ATOM 2124 GL2 POP 242 27.830 38.420 48.960 1.00 0.00

ATOM 2125 C1A POP 242 24.540 37.480 51.580 1.00 0.00

ATOM 2126 C2A POP 242 22.490 35.780 55.200 1.00 0.00

ATOM 2127 C3A POP 242 20.540 35.570 58.860 1.00 0.00

ATOM 2128 C4A POP 242 21.390 34.200 63.340 1.00 0.00

ATOM 2129 C1B POP 242 25.540 42.660 50.410 1.00 0.00

ATOM 2130 C2B POP 242 21.590 41.390 53.710 1.00 0.00

ATOM 2131 D3B POP 242 22.030 41.080 58.490 1.00 0.00

ATOM 2132 C4B POP 242 25.500 43.630 60.920 1.00 0.00

ATOM 2133 C5B POP 242 26.690 45.170 64.680 1.00 0.00

ATOM 2134 NC3 POP 243 13.850 78.660 41.280 1.00 0.00

ATOM 2135 PO4 POP 243 17.470 77.460 43.850 1.00 0.00

ATOM 2136 GL1 POP 243 19.240 78.240 48.520 1.00 0.00

ATOM 2137 GL2 POP 243 19.530 75.290 50.880 1.00 0.00

ATOM 2138 C1A POP 243 17.770 73.400 54.600 1.00 0.00

ATOM 2139 C2A POP 243 21.060 71.920 57.720 1.00 0.00

ATOM 2140 C3A POP 243 22.910 72.860 60.800 1.00 0.00

ATOM 2141 C4A POP 243 23.900 70.010 63.280 1.00 0.00

ATOM 2142 C1B POP 243 19.300 79.690 53.580 1.00 0.00

ATOM 2143 C2B POP 243 19.790 81.670 57.520 1.00 0.00

ATOM 2144 D3B POP 243 19.170 80.180 62.870 1.00 0.00

ATOM 2145 C4B POP 243 15.910 81.510 65.520 1.00 0.00

ATOM 2146 C5B POP 243 11.760 81.580 66.430 1.00 0.00

ATOM 2147 NC3 POP 244 82.320 54.780 46.520 1.00 0.00

ATOM 2148 PO4 POP 244 85.350 51.540 48.410 1.00 0.00

ATOM 2149 GL1 POP 244 85.590 50.650 52.360 1.00 0.00

ATOM 2150 GL2 POP 244 87.050 47.780 52.720 1.00 0.00

ATOM 2151 C1A POP 244 89.380 45.280 55.140 1.00 0.00

ATOM 2152 C2A POP 244 90.860 44.180 59.720 1.00 0.00

ATOM 2153 C3A POP 244 93.540 41.670 59.150 1.00 0.00

ATOM 2154 C4A POP 244 97.810 40.490 59.880 1.00 0.00

ATOM 2155 C1B POP 244 85.720 48.720 57.330 1.00 0.00

ATOM 2156 C2B POP 244 88.130 47.710 61.380 1.00 0.00

ATOM 2157 D3B POP 244 91.350 46.080 64.700 1.00 0.00

ATOM 2158 C4B POP 244 93.710 42.860 63.740 1.00 0.00

ATOM 2159 C5B POP 244 95.560 38.600 63.620 1.00 0.00

ATOM 2160 NC3 POP 245 56.610 105.330 88.570 1.00 0.00

ATOM 2161 PO4 POP 245 54.760 103.050 85.210 1.00 0.00

ATOM 2162 GL1 POP 245 55.810 103.950 80.190 1.00 0.00

ATOM 2163 GL2 POP 245 54.830 107.850 79.070 1.00 0.00

ATOM 2164 C1A POP 245 57.900 108.920 75.190 1.00 0.00

ATOM 2165 C2A POP 245 59.140 113.070 74.110 1.00 0.00

ATOM 2166 C3A POP 245 58.940 117.360 71.570 1.00 0.00

ATOM 2167 C4A POP 245 60.040 120.120 68.790 1.00 0.00

ATOM 2168 C1B POP 245 54.510 103.740 75.860 1.00 0.00

ATOM 2169 C2B POP 245 50.890 103.970 73.300 1.00 0.00

ATOM 2170 D3B POP 245 47.170 100.630 72.430 1.00 0.00

ATOM 2171 C4B POP 245 43.140 100.240 71.060 1.00 0.00

ATOM 2172 C5B POP 245 38.440 101.170 69.860 1.00 0.00

ATOM 2173 NC3 POP 246 60.380 77.390 85.880 1.00 0.00

ATOM 2174 PO4 POP 246 64.900 77.760 86.760 1.00 0.00

ATOM 2175 GL1 POP 246 66.150 79.660 83.100 1.00 0.00

ATOM 2176 GL2 POP 246 68.230 80.430 82.780 1.00 0.00

ATOM 2177 C1A POP 246 68.520 82.760 78.660 1.00 0.00

ATOM 2178 C2A POP 246 67.070 84.280 74.380 1.00 0.00

ATOM 2179 C3A POP 246 70.830 83.070 70.750 1.00 0.00

ATOM 2180 C4A POP 246 74.070 84.740 68.060 1.00 0.00

ATOM 2181 C1B POP 246 67.250 77.650 78.130 1.00 0.00

ATOM 2182 C2B POP 246 66.510 78.590 73.410 1.00 0.00

ATOM 2183 D3B POP 246 67.090 78.960 69.510 1.00 0.00

ATOM 2184 C4B POP 246 65.860 81.210 66.860 1.00 0.00

ATOM 2185 C5B POP 246 63.380 84.640 64.320 1.00 0.00

ATOM 2186 NC3 POP 247 90.910 80.390 48.770 1.00 0.00

ATOM 2187 PO4 POP 247 87.510 83.800 47.680 1.00 0.00

ATOM 2188 GL1 POP 247 87.850 82.770 51.750 1.00 0.00

ATOM 2189 GL2 POP 247 85.130 81.210 53.040 1.00 0.00

ATOM 2190 C1A POP 247 88.250 79.150 56.120 1.00 0.00

ATOM 2191 C2A POP 247 91.640 76.030 57.680 1.00 0.00

ATOM 2192 C3A POP 247 93.610 75.810 61.630 1.00 0.00

ATOM 2193 C4A POP 247 95.080 74.610 66.020 1.00 0.00

ATOM 2194 C1B POP 247 90.320 84.000 55.560 1.00 0.00

ATOM 2195 C2B POP 247 93.410 82.490 57.620 1.00 0.00

ATOM 2196 D3B POP 247 96.520 84.640 59.440 1.00 0.00

ATOM 2197 C4B POP 247 98.520 82.940 62.520 1.00 0.00

ATOM 2198 C5B POP 247 101.920 79.500 61.540 1.00 0.00

ATOM 2199 NC3 POP 248 55.570 29.450 85.620 1.00 0.00

ATOM 2200 PO4 POP 248 51.480 28.830 84.450 1.00 0.00

ATOM 2201 GL1 POP 248 52.450 28.910 80.220 1.00 0.00

ATOM 2202 GL2 POP 248 50.570 27.050 77.460 1.00 0.00

ATOM 2203 C1A POP 248 53.500 24.240 76.060 1.00 0.00

ATOM 2204 C2A POP 248 55.220 25.900 71.840 1.00 0.00

ATOM 2205 C3A POP 248 57.210 27.460 68.800 1.00 0.00

ATOM 2206 C4A POP 248 59.300 29.690 66.570 1.00 0.00

ATOM 2207 C1B POP 248 55.370 29.650 75.800 1.00 0.00

ATOM 2208 C2B POP 248 58.040 29.990 72.770 1.00 0.00

ATOM 2209 D3B POP 248 61.970 31.750 70.560 1.00 0.00

ATOM 2210 C4B POP 248 66.280 31.190 72.280 1.00 0.00

ATOM 2211 C5B POP 248 67.680 27.880 72.190 1.00 0.00

ATOM 2212 NC3 POP 249 64.630 108.120 87.350 1.00 0.00

ATOM 2213 PO4 POP 249 63.420 109.720 83.320 1.00 0.00

ATOM 2214 GL1 POP 249 66.990 107.920 80.510 1.00 0.00

ATOM 2215 GL2 POP 249 66.360 105.560 78.170 1.00 0.00

ATOM 2216 C1A POP 249 67.740 103.900 74.080 1.00 0.00

ATOM 2217 C2A POP 249 67.910 102.830 69.410 1.00 0.00

ATOM 2218 C3A POP 249 72.300 102.860 69.270 1.00 0.00

ATOM 2219 C4A POP 249 74.760 100.820 66.450 1.00 0.00

ATOM 2220 C1B POP 249 69.680 109.050 76.100 1.00 0.00

ATOM 2221 C2B POP 249 71.430 106.960 72.510 1.00 0.00

ATOM 2222 D3B POP 249 70.940 108.840 67.480 1.00 0.00

ATOM 2223 C4B POP 249 67.480 106.840 65.580 1.00 0.00

ATOM 2224 C5B POP 249 63.620 107.180 63.440 1.00 0.00

ATOM 2225 NC3 POP 250 7.220 53.120 90.560 1.00 0.00

ATOM 2226 PO4 POP 250 11.250 53.400 88.270 1.00 0.00

ATOM 2227 GL1 POP 250 11.030 52.630 85.320 1.00 0.00

ATOM 2228 GL2 POP 250 9.170 50.060 84.810 1.00 0.00

ATOM 2229 C1A POP 250 10.150 46.340 81.590 1.00 0.00

ATOM 2230 C2A POP 250 10.040 44.640 77.040 1.00 0.00

ATOM 2231 C3A POP 250 10.710 39.700 76.210 1.00 0.00

ATOM 2232 C4A POP 250 13.870 36.340 73.610 1.00 0.00

ATOM 2233 C1B POP 250 12.450 51.640 80.760 1.00 0.00

ATOM 2234 C2B POP 250 12.850 54.520 76.970 1.00 0.00

ATOM 2235 D3B POP 250 10.800 56.160 73.350 1.00 0.00

ATOM 2236 C4B POP 250 12.090 56.010 69.280 1.00 0.00

ATOM 2237 C5B POP 250 15.750 57.040 65.900 1.00 0.00

ATOM 2238 NC3 POP 251 100.090 3.600 88.860 1.00 0.00

ATOM 2239 PO4 POP 251 102.950 3.830 86.220 1.00 0.00

ATOM 2240 GL1 POP 251 104.520 3.380 83.280 1.00 0.00

ATOM 2241 GL2 POP 251 107.180 4.740 82.430 1.00 0.00

ATOM 2242 C1A POP 251 107.410 5.040 77.330 1.00 0.00

ATOM 2243 C2A POP 251 109.730 7.040 73.750 1.00 0.00

ATOM 2244 C3A POP 251 107.120 9.440 70.310 1.00 0.00

ATOM 2245 C4A POP 251 105.510 13.950 68.630 1.00 0.00

ATOM 2246 C1B POP 251 102.570 2.890 79.520 1.00 0.00

ATOM 2247 C2B POP 251 102.700 4.570 75.450 1.00 0.00

ATOM 2248 D3B POP 251 104.560 6.760 73.250 1.00 0.00

ATOM 2249 C4B POP 251 104.860 5.510 69.560 1.00 0.00

ATOM 2250 C5B POP 251 104.470 2.140 66.960 1.00 0.00

ATOM 2251 NC3 POP 252 100.460 104.620 87.290 1.00 0.00

ATOM 2252 PO4 POP 252 99.430 109.230 85.880 1.00 0.00

ATOM 2253 GL1 POP 252 100.090 108.490 82.240 1.00 0.00

ATOM 2254 GL2 POP 252 102.430 106.230 82.610 1.00 0.00

ATOM 2255 C1A POP 252 105.350 108.140 81.050 1.00 0.00

ATOM 2256 C2A POP 252 105.710 109.200 76.940 1.00 0.00

ATOM 2257 C3A POP 252 107.340 106.540 72.350 1.00 0.00

ATOM 2258 C4A POP 252 105.710 106.670 68.200 1.00 0.00

ATOM 2259 C1B POP 252 100.120 107.470 78.160 1.00 0.00

ATOM 2260 C2B POP 252 99.680 105.130 74.190 1.00 0.00

ATOM 2261 D3B POP 252 102.090 105.670 71.790 1.00 0.00

ATOM 2262 C4B POP 252 104.510 110.070 72.000 1.00 0.00

ATOM 2263 C5B POP 252 108.130 112.180 72.830 1.00 0.00

ATOM 2264 NC3 POP 253 7.760 36.600 48.200 1.00 0.00

ATOM 2265 PO4 POP 253 7.710 41.010 49.160 1.00 0.00

ATOM 2266 GL1 POP 253 7.290 40.730 52.870 1.00 0.00

ATOM 2267 GL2 POP 253 6.200 36.760 52.710 1.00 0.00

ATOM 2268 C1A POP 253 5.270 36.060 57.050 1.00 0.00

ATOM 2269 C2A POP 253 4.400 34.250 62.050 1.00 0.00

ATOM 2270 C3A POP 253 2.850 34.260 66.820 1.00 0.00

ATOM 2271 C4A POP 253 2.980 36.130 70.680 1.00 0.00

ATOM 2272 C1B POP 253 10.570 38.590 56.190 1.00 0.00

ATOM 2273 C2B POP 253 11.600 37.690 61.110 1.00 0.00

ATOM 2274 D3B POP 253 12.560 39.460 64.940 1.00 0.00

ATOM 2275 C4B POP 253 12.150 43.930 66.550 1.00 0.00

ATOM 2276 C5B POP 253 14.650 46.490 68.640 1.00 0.00

ATOM 2277 NC3 POP 254 78.830 45.070 47.440 1.00 0.00

ATOM 2278 PO4 POP 254 74.320 44.240 46.750 1.00 0.00

ATOM 2279 GL1 POP 254 75.370 45.060 51.460 1.00 0.00

ATOM 2280 GL2 POP 254 77.990 43.510 52.540 1.00 0.00

ATOM 2281 C1A POP 254 74.960 41.690 55.630 1.00 0.00

ATOM 2282 C2A POP 254 73.260 43.830 60.090 1.00 0.00

ATOM 2283 C3A POP 254 72.190 48.300 61.660 1.00 0.00

ATOM 2284 C4A POP 254 69.540 51.210 63.060 1.00 0.00

ATOM 2285 C1B POP 254 75.660 46.270 56.510 1.00 0.00

ATOM 2286 C2B POP 254 77.370 46.380 61.390 1.00 0.00

ATOM 2287 D3B POP 254 77.250 48.310 64.420 1.00 0.00

ATOM 2288 C4B POP 254 72.610 49.310 66.720 1.00 0.00

ATOM 2289 C5B POP 254 67.720 48.190 67.490 1.00 0.00

ATOM 2290 NC3 POP 255 75.630 31.790 44.820 1.00 0.00

ATOM 2291 PO4 POP 255 78.090 34.860 44.840 1.00 0.00

ATOM 2292 GL1 POP 255 77.240 33.920 48.730 1.00 0.00

ATOM 2293 GL2 POP 255 74.460 31.460 49.430 1.00 0.00

ATOM 2294 C1A POP 255 76.540 30.000 52.560 1.00 0.00

ATOM 2295 C2A POP 255 77.980 30.510 56.290 1.00 0.00

ATOM 2296 C3A POP 255 79.220 31.340 59.960 1.00 0.00

ATOM 2297 C4A POP 255 79.000 31.240 63.630 1.00 0.00

ATOM 2298 C1B POP 255 75.820 36.700 52.590 1.00 0.00

ATOM 2299 C2B POP 255 76.770 36.980 56.670 1.00 0.00

ATOM 2300 D3B POP 255 74.730 35.030 60.570 1.00 0.00

ATOM 2301 C4B POP 255 74.100 36.560 64.550 1.00 0.00

ATOM 2302 C5B POP 255 71.480 37.430 67.720 1.00 0.00

ATOM 2303 NC3 POP 256 69.000 79.210 45.790 1.00 0.00

ATOM 2304 PO4 POP 256 72.560 76.960 47.690 1.00 0.00

ATOM 2305 GL1 POP 256 73.280 76.730 52.410 1.00 0.00

ATOM 2306 GL2 POP 256 71.940 75.160 54.580 1.00 0.00

ATOM 2307 C1A POP 256 71.620 77.810 59.050 1.00 0.00

ATOM 2308 C2A POP 256 70.410 80.870 62.840 1.00 0.00

ATOM 2309 C3A POP 256 72.060 79.650 66.920 1.00 0.00

ATOM 2310 C4A POP 256 74.750 80.260 69.820 1.00 0.00

ATOM 2311 C1B POP 256 74.190 79.980 55.250 1.00 0.00

ATOM 2312 C2B POP 256 76.830 82.470 58.930 1.00 0.00

ATOM 2313 D3B POP 256 79.400 84.040 62.350 1.00 0.00

ATOM 2314 C4B POP 256 77.320 87.010 65.440 1.00 0.00

ATOM 2315 C5B POP 256 73.320 89.720 67.040 1.00 0.00

ATOM 2316 NC3 POP 257 67.570 67.700 46.110 1.00 0.00

ATOM 2317 PO4 POP 257 65.620 63.710 45.810 1.00 0.00

ATOM 2318 GL1 POP 257 67.120 64.290 50.030 1.00 0.00

ATOM 2319 GL2 POP 257 70.160 64.550 49.590 1.00 0.00

ATOM 2320 C1A POP 257 72.330 65.350 52.960 1.00 0.00

ATOM 2321 C2A POP 257 71.020 67.470 55.060 1.00 0.00

ATOM 2322 C3A POP 257 70.050 70.940 57.520 1.00 0.00

ATOM 2323 C4A POP 257 69.700 72.980 60.830 1.00 0.00

ATOM 2324 C1B POP 257 68.650 63.050 54.190 1.00 0.00

ATOM 2325 C2B POP 257 71.440 64.160 58.600 1.00 0.00

ATOM 2326 D3B POP 257 70.580 63.890 63.210 1.00 0.00

ATOM 2327 C4B POP 257 72.970 67.600 63.840 1.00 0.00

ATOM 2328 C5B POP 257 73.420 69.490 60.550 1.00 0.00

ATOM 2329 NC3 POP 258 3.660 63.930 89.960 1.00 0.00

ATOM 2330 PO4 POP 258 8.220 65.880 89.670 1.00 0.00

ATOM 2331 GL1 POP 258 6.630 63.850 85.970 1.00 0.00

ATOM 2332 GL2 POP 258 9.160 61.930 86.980 1.00 0.00

ATOM 2333 C1A POP 258 12.380 61.890 84.290 1.00 0.00

ATOM 2334 C2A POP 258 14.180 61.760 81.120 1.00 0.00

ATOM 2335 C3A POP 258 18.380 61.900 78.800 1.00 0.00

ATOM 2336 C4A POP 258 18.470 63.860 74.300 1.00 0.00

ATOM 2337 C1B POP 258 6.340 60.140 83.450 1.00 0.00

ATOM 2338 C2B POP 258 5.740 59.560 79.700 1.00 0.00

ATOM 2339 D3B POP 258 5.370 59.030 75.390 1.00 0.00

ATOM 2340 C4B POP 258 3.000 55.490 74.610 1.00 0.00

ATOM 2341 C5B POP 258 4.200 52.040 71.720 1.00 0.00

ATOM 2342 NC3 POP 259 87.740 50.290 90.840 1.00 0.00

ATOM 2343 PO4 POP 259 84.180 48.390 88.770 1.00 0.00

ATOM 2344 GL1 POP 259 84.670 47.340 83.720 1.00 0.00

ATOM 2345 GL2 POP 259 85.400 44.900 82.970 1.00 0.00

ATOM 2346 C1A POP 259 83.160 42.890 79.060 1.00 0.00

ATOM 2347 C2A POP 259 80.120 43.300 74.630 1.00 0.00

ATOM 2348 C3A POP 259 81.590 44.350 70.050 1.00 0.00

ATOM 2349 C4A POP 259 82.130 47.440 66.780 1.00 0.00

ATOM 2350 C1B POP 259 83.680 47.790 78.930 1.00 0.00

ATOM 2351 C2B POP 259 84.230 45.830 75.260 1.00 0.00

ATOM 2352 D3B POP 259 84.770 41.970 72.940 1.00 0.00

ATOM 2353 C4B POP 259 87.700 41.080 70.210 1.00 0.00

ATOM 2354 C5B POP 259 90.960 39.660 67.230 1.00 0.00

ATOM 2355 NC3 POP 260 2.580 37.160 88.990 1.00 0.00

ATOM 2356 PO4 POP 260 2.330 32.980 86.700 1.00 0.00

ATOM 2357 GL1 POP 260 1.510 35.140 82.640 1.00 0.00

ATOM 2358 GL2 POP 260 4.050 37.650 81.750 1.00 0.00

ATOM 2359 C1A POP 260 1.550 37.780 78.220 1.00 0.00

ATOM 2360 C2A POP 260 1.490 39.210 74.480 1.00 0.00

ATOM 2361 C3A POP 260 1.340 41.830 70.450 1.00 0.00

ATOM 2362 C4A POP 260 -1.800 42.820 66.880 1.00 0.00

ATOM 2363 C1B POP 260 -1.140 33.090 79.400 1.00 0.00

ATOM 2364 C2B POP 260 0.930 29.710 77.070 1.00 0.00

ATOM 2365 D3B POP 260 2.040 26.280 74.370 1.00 0.00

ATOM 2366 C4B POP 260 5.870 24.950 73.540 1.00 0.00

ATOM 2367 C5B POP 260 7.910 23.480 77.790 1.00 0.00

ATOM 2368 NC3 POP 261 57.680 16.360 39.420 1.00 0.00

ATOM 2369 PO4 POP 261 57.430 16.410 43.920 1.00 0.00

ATOM 2370 GL1 POP 261 56.790 15.830 47.970 1.00 0.00

ATOM 2371 GL2 POP 261 59.480 13.980 48.580 1.00 0.00

ATOM 2372 C1A POP 261 60.710 16.170 51.440 1.00 0.00

ATOM 2373 C2A POP 261 62.500 13.830 55.710 1.00 0.00

ATOM 2374 C3A POP 261 64.540 13.480 60.040 1.00 0.00

ATOM 2375 C4A POP 261 68.380 13.290 63.800 1.00 0.00

ATOM 2376 C1B POP 261 54.390 13.800 51.340 1.00 0.00

ATOM 2377 C2B POP 261 53.160 12.090 55.920 1.00 0.00

ATOM 2378 D3B POP 261 50.300 12.670 59.830 1.00 0.00

ATOM 2379 C4B POP 261 49.590 17.630 60.520 1.00 0.00

ATOM 2380 C5B POP 261 51.110 21.430 61.570 1.00 0.00

ATOM 2381 NC3 POP 262 89.090 3.580 39.590 1.00 0.00

ATOM 2382 PO4 POP 262 89.390 6.170 41.770 1.00 0.00

ATOM 2383 GL1 POP 262 90.920 5.780 45.860 1.00 0.00

ATOM 2384 GL2 POP 262 93.290 3.710 47.570 1.00 0.00

ATOM 2385 C1A POP 262 92.900 1.880 50.880 1.00 0.00

ATOM 2386 C2A POP 262 91.270 -0.920 53.660 1.00 0.00

ATOM 2387 C3A POP 262 88.900 -2.820 56.810 1.00 0.00

ATOM 2388 C4A POP 262 87.430 -3.490 61.170 1.00 0.00

ATOM 2389 C1B POP 262 89.770 6.960 50.070 1.00 0.00

ATOM 2390 C2B POP 262 88.610 10.030 53.140 1.00 0.00

ATOM 2391 D3B POP 262 84.840 10.270 56.370 1.00 0.00

ATOM 2392 C4B POP 262 84.140 5.760 57.330 1.00 0.00

ATOM 2393 C5B POP 262 83.050 1.690 60.130 1.00 0.00

ATOM 2394 NC3 POP 263 39.060 1.740 44.800 1.00 0.00

ATOM 2395 PO4 POP 263 34.530 2.280 43.000 1.00 0.00

ATOM 2396 GL1 POP 263 33.710 0.750 47.040 1.00 0.00

ATOM 2397 GL2 POP 263 35.410 -0.880 48.830 1.00 0.00

ATOM 2398 C1A POP 263 34.130 -1.100 53.700 1.00 0.00

ATOM 2399 C2A POP 263 35.540 -2.190 57.810 1.00 0.00

ATOM 2400 C3A POP 263 37.450 -2.670 61.710 1.00 0.00

ATOM 2401 C4A POP 263 37.980 -0.520 66.220 1.00 0.00

ATOM 2402 C1B POP 263 31.440 1.670 50.290 1.00 0.00

ATOM 2403 C2B POP 263 28.960 4.690 52.390 1.00 0.00

ATOM 2404 D3B POP 263 29.160 7.310 54.910 1.00 0.00

ATOM 2405 C4B POP 263 29.910 7.780 59.770 1.00 0.00

ATOM 2406 C5B POP 263 29.310 12.090 61.890 1.00 0.00

ATOM 2407 NC3 POP 264 48.190 40.320 92.090 1.00 0.00

ATOM 2408 PO4 POP 264 50.030 42.340 88.920 1.00 0.00

ATOM 2409 GL1 POP 264 50.680 42.660 84.280 1.00 0.00

ATOM 2410 GL2 POP 264 53.800 43.520 83.560 1.00 0.00

ATOM 2411 C1A POP 264 53.960 46.320 80.830 1.00 0.00

ATOM 2412 C2A POP 264 51.920 49.280 79.400 1.00 0.00

ATOM 2413 C3A POP 264 48.260 49.560 75.660 1.00 0.00

ATOM 2414 C4A POP 264 45.030 50.880 72.790 1.00 0.00

ATOM 2415 C1B POP 264 50.050 43.740 79.000 1.00 0.00

ATOM 2416 C2B POP 264 50.240 44.780 74.590 1.00 0.00

ATOM 2417 D3B POP 264 49.430 46.830 71.130 1.00 0.00

ATOM 2418 C4B POP 264 48.490 44.390 65.700 1.00 0.00

ATOM 2419 C5B POP 264 50.310 40.580 64.870 1.00 0.00

ATOM 2420 NC3 POP 265 100.120 98.930 92.990 1.00 0.00

ATOM 2421 PO4 POP 265 101.570 99.830 88.320 1.00 0.00

ATOM 2422 GL1 POP 265 101.770 101.440 83.600 1.00 0.00

ATOM 2423 GL2 POP 265 98.450 103.010 81.790 1.00 0.00

ATOM 2424 C1A POP 265 99.110 99.230 79.360 1.00 0.00

ATOM 2425 C2A POP 265 96.480 99.100 75.940 1.00 0.00

ATOM 2426 C3A POP 265 92.500 98.730 73.110 1.00 0.00

ATOM 2427 C4A POP 265 91.550 99.170 69.510 1.00 0.00

ATOM 2428 C1B POP 265 102.830 102.930 78.820 1.00 0.00

ATOM 2429 C2B POP 265 102.140 100.080 74.760 1.00 0.00

ATOM 2430 D3B POP 265 100.440 100.700 69.750 1.00 0.00

ATOM 2431 C4B POP 265 102.500 102.060 66.620 1.00 0.00

ATOM 2432 C5B POP 265 102.460 106.680 65.010 1.00 0.00

ATOM 2433 NC3 POP 266 8.800 21.150 88.530 1.00 0.00

ATOM 2434 PO4 POP 266 11.690 17.140 88.650 1.00 0.00

ATOM 2435 GL1 POP 266 11.800 18.200 84.270 1.00 0.00

ATOM 2436 GL2 POP 266 14.380 16.150 83.080 1.00 0.00

ATOM 2437 C1A POP 266 14.010 15.470 78.610 1.00 0.00

ATOM 2438 C2A POP 266 14.010 18.380 74.840 1.00 0.00

ATOM 2439 C3A POP 266 13.020 17.680 72.090 1.00 0.00

ATOM 2440 C4A POP 266 12.850 14.470 68.810 1.00 0.00

ATOM 2441 C1B POP 266 9.890 18.890 80.400 1.00 0.00

ATOM 2442 C2B POP 266 9.340 18.830 76.220 1.00 0.00

ATOM 2443 D3B POP 266 9.760 21.860 73.130 1.00 0.00

ATOM 2444 C4B POP 266 13.580 22.730 71.830 1.00 0.00

ATOM 2445 C5B POP 266 14.040 26.880 69.920 1.00 0.00

ATOM 2446 NC3 POP 267 3.950 55.100 93.230 1.00 0.00

ATOM 2447 PO4 POP 267 4.040 58.310 90.640 1.00 0.00

ATOM 2448 GL1 POP 267 5.620 56.480 87.300 1.00 0.00

ATOM 2449 GL2 POP 267 8.090 56.540 86.060 1.00 0.00

ATOM 2450 C1A POP 267 8.040 54.820 80.800 1.00 0.00

ATOM 2451 C2A POP 267 7.830 54.400 77.200 1.00 0.00

ATOM 2452 C3A POP 267 9.200 51.920 73.030 1.00 0.00

ATOM 2453 C4A POP 267 11.540 50.400 69.130 1.00 0.00

ATOM 2454 C1B POP 267 2.640 55.290 83.560 1.00 0.00

ATOM 2455 C2B POP 267 3.690 51.970 79.630 1.00 0.00

ATOM 2456 D3B POP 267 4.020 49.580 75.870 1.00 0.00

ATOM 2457 C4B POP 267 7.660 47.070 73.720 1.00 0.00

ATOM 2458 C5B POP 267 8.790 44.590 70.800 1.00 0.00

ATOM 2459 NC3 POP 268 3.460 86.910 93.300 1.00 0.00

ATOM 2460 PO4 POP 268 4.010 86.170 88.160 1.00 0.00

ATOM 2461 GL1 POP 268 3.760 85.960 84.310 1.00 0.00

ATOM 2462 GL2 POP 268 3.680 83.030 83.250 1.00 0.00

ATOM 2463 C1A POP 268 4.700 81.920 79.020 1.00 0.00

ATOM 2464 C2A POP 268 8.510 80.660 76.160 1.00 0.00

ATOM 2465 C3A POP 268 11.040 77.980 74.730 1.00 0.00

ATOM 2466 C4A POP 268 14.630 76.070 74.040 1.00 0.00

ATOM 2467 C1B POP 268 5.560 87.140 80.760 1.00 0.00

ATOM 2468 C2B POP 268 6.820 85.500 75.780 1.00 0.00

ATOM 2469 D3B POP 268 8.470 85.300 71.980 1.00 0.00

ATOM 2470 C4B POP 268 10.160 80.920 70.780 1.00 0.00

ATOM 2471 C5B POP 268 8.830 77.460 69.750 1.00 0.00

ATOM 2472 NC3 POP 269 36.020 62.030 48.240 1.00 0.00

ATOM 2473 PO4 POP 269 40.310 59.070 48.000 1.00 0.00

ATOM 2474 GL1 POP 269 38.880 57.730 51.590 1.00 0.00

ATOM 2475 GL2 POP 269 39.310 55.150 52.450 1.00 0.00

ATOM 2476 C1A POP 269 35.660 55.390 55.570 1.00 0.00

ATOM 2477 C2A POP 269 34.090 54.630 59.680 1.00 0.00

ATOM 2478 C3A POP 269 34.540 53.600 63.900 1.00 0.00

ATOM 2479 C4A POP 269 37.330 55.660 66.970 1.00 0.00

ATOM 2480 C1B POP 269 39.080 58.630 55.990 1.00 0.00

ATOM 2481 C2B POP 269 40.410 61.450 58.500 1.00 0.00

ATOM 2482 D3B POP 269 42.790 63.740 57.960 1.00 0.00

ATOM 2483 C4B POP 269 44.370 65.800 54.680 1.00 0.00

ATOM 2484 C5B POP 269 47.410 64.400 52.330 1.00 0.00

ATOM 2485 NC3 POP 270 72.460 54.190 46.610 1.00 0.00

ATOM 2486 PO4 POP 270 68.190 54.750 45.080 1.00 0.00

ATOM 2487 GL1 POP 270 66.280 55.550 49.660 1.00 0.00

ATOM 2488 GL2 POP 270 65.260 58.890 51.390 1.00 0.00

ATOM 2489 C1A POP 270 64.400 58.610 55.010 1.00 0.00

ATOM 2490 C2A POP 270 65.720 57.020 58.650 1.00 0.00

ATOM 2491 C3A POP 270 63.480 55.310 61.470 1.00 0.00

ATOM 2492 C4A POP 270 60.940 53.980 65.200 1.00 0.00

ATOM 2493 C1B POP 270 68.500 55.640 53.580 1.00 0.00

ATOM 2494 C2B POP 270 69.310 58.770 56.590 1.00 0.00

ATOM 2495 D3B POP 270 70.540 57.950 61.200 1.00 0.00

ATOM 2496 C4B POP 270 67.450 57.550 63.320 1.00 0.00

ATOM 2497 C5B POP 270 63.770 58.690 65.260 1.00 0.00

ATOM 2498 NC3 POP 271 11.990 12.500 86.810 1.00 0.00

ATOM 2499 PO4 POP 271 10.420 8.140 83.480 1.00 0.00

ATOM 2500 GL1 POP 271 11.750 6.740 79.830 1.00 0.00

ATOM 2501 GL2 POP 271 12.220 9.620 78.960 1.00 0.00

ATOM 2502 C1A POP 271 8.970 8.320 75.900 1.00 0.00

ATOM 2503 C2A POP 271 7.960 6.690 71.520 1.00 0.00

ATOM 2504 C3A POP 271 6.730 1.900 69.310 1.00 0.00

ATOM 2505 C4A POP 271 5.670 -1.740 67.060 1.00 0.00

ATOM 2506 C1B POP 271 13.480 7.230 75.180 1.00 0.00

ATOM 2507 C2B POP 271 15.700 4.550 71.470 1.00 0.00

ATOM 2508 D3B POP 271 15.490 0.490 68.990 1.00 0.00

ATOM 2509 C4B POP 271 18.920 0.100 64.620 1.00 0.00

ATOM 2510 C5B POP 271 20.590 2.300 61.260 1.00 0.00

ATOM 2511 NC3 POP 272 30.510 67.880 91.900 1.00 0.00

ATOM 2512 PO4 POP 272 33.720 70.900 89.780 1.00 0.00

ATOM 2513 GL1 POP 272 29.770 70.470 87.260 1.00 0.00

ATOM 2514 GL2 POP 272 29.520 72.900 85.140 1.00 0.00

ATOM 2515 C1A POP 272 27.410 72.410 81.210 1.00 0.00

ATOM 2516 C2A POP 272 25.700 74.180 77.840 1.00 0.00

ATOM 2517 C3A POP 272 25.240 78.000 74.710 1.00 0.00

ATOM 2518 C4A POP 272 24.220 80.750 70.500 1.00 0.00

ATOM 2519 C1B POP 272 26.410 68.500 84.790 1.00 0.00

ATOM 2520 C2B POP 272 23.270 68.270 82.260 1.00 0.00

ATOM 2521 D3B POP 272 23.790 69.190 77.820 1.00 0.00

ATOM 2522 C4B POP 272 26.820 68.330 74.640 1.00 0.00

ATOM 2523 C5B POP 272 28.250 72.410 73.170 1.00 0.00

ATOM 2524 NC3 POP 273 37.650 66.580 48.240 1.00 0.00

ATOM 2525 PO4 POP 273 40.270 63.980 50.080 1.00 0.00

ATOM 2526 GL1 POP 273 38.780 63.250 54.200 1.00 0.00

ATOM 2527 GL2 POP 273 39.800 66.760 54.280 1.00 0.00

ATOM 2528 C1A POP 273 41.410 67.930 58.440 1.00 0.00

ATOM 2529 C2A POP 273 43.760 71.380 61.060 1.00 0.00

ATOM 2530 C3A POP 273 45.160 70.950 66.030 1.00 0.00

ATOM 2531 C4A POP 273 50.230 69.890 67.800 1.00 0.00

ATOM 2532 C1B POP 273 37.010 64.650 57.540 1.00 0.00

ATOM 2533 C2B POP 273 37.880 64.680 61.580 1.00 0.00

ATOM 2534 D3B POP 273 40.200 67.130 65.290 1.00 0.00

ATOM 2535 C4B POP 273 43.010 66.010 66.980 1.00 0.00

ATOM 2536 C5B POP 273 46.970 66.090 67.430 1.00 0.00

ATOM 2537 NC3 POP 274 80.330 17.800 84.530 1.00 0.00

ATOM 2538 PO4 POP 274 79.350 20.980 83.390 1.00 0.00

ATOM 2539 GL1 POP 274 79.440 23.730 79.310 1.00 0.00

ATOM 2540 GL2 POP 274 82.440 23.230 79.690 1.00 0.00

ATOM 2541 C1A POP 274 84.760 23.950 76.370 1.00 0.00

ATOM 2542 C2A POP 274 89.020 24.600 75.470 1.00 0.00

ATOM 2543 C3A POP 274 91.670 24.490 71.540 1.00 0.00

ATOM 2544 C4A POP 274 92.150 29.070 69.420 1.00 0.00

ATOM 2545 C1B POP 274 79.320 24.920 75.710 1.00 0.00

ATOM 2546 C2B POP 274 79.850 27.020 72.080 1.00 0.00

ATOM 2547 D3B POP 274 77.770 30.210 69.380 1.00 0.00

ATOM 2548 C4B POP 274 74.890 33.690 70.390 1.00 0.00

ATOM 2549 C5B POP 274 72.900 36.260 72.390 1.00 0.00

ATOM 2550 NC3 POP 275 84.480 1.500 40.550 1.00 0.00

ATOM 2551 PO4 POP 275 85.560 4.100 44.010 1.00 0.00

ATOM 2552 GL1 POP 275 84.830 4.500 48.290 1.00 0.00

ATOM 2553 GL2 POP 275 82.250 3.790 48.550 1.00 0.00

ATOM 2554 C1A POP 275 77.970 4.550 49.740 1.00 0.00

ATOM 2555 C2A POP 275 75.560 2.320 53.500 1.00 0.00

ATOM 2556 C3A POP 275 75.690 -1.790 55.060 1.00 0.00

ATOM 2557 C4A POP 275 73.570 -5.190 54.180 1.00 0.00

ATOM 2558 C1B POP 275 85.430 5.460 52.510 1.00 0.00

ATOM 2559 C2B POP 275 88.760 2.970 55.010 1.00 0.00

ATOM 2560 D3B POP 275 88.980 1.550 59.540 1.00 0.00

ATOM 2561 C4B POP 275 86.480 4.020 62.690 1.00 0.00

ATOM 2562 C5B POP 275 87.320 8.010 64.740 1.00 0.00

ATOM 2563 NC3 POP 276 24.500 20.160 38.110 1.00 0.00

ATOM 2564 PO4 POP 276 27.180 21.330 41.590 1.00 0.00

ATOM 2565 GL1 POP 276 28.380 17.900 45.490 1.00 0.00

ATOM 2566 GL2 POP 276 25.410 16.950 46.710 1.00 0.00

ATOM 2567 C1A POP 276 26.190 14.290 50.330 1.00 0.00

ATOM 2568 C2A POP 276 23.890 13.100 53.960 1.00 0.00

ATOM 2569 C3A POP 276 21.030 10.900 57.150 1.00 0.00

ATOM 2570 C4A POP 276 16.600 11.100 57.680 1.00 0.00

ATOM 2571 C1B POP 276 28.450 18.860 50.300 1.00 0.00

ATOM 2572 C2B POP 276 25.660 19.390 54.560 1.00 0.00

ATOM 2573 D3B POP 276 25.400 16.190 58.840 1.00 0.00

ATOM 2574 C4B POP 276 26.960 18.500 62.350 1.00 0.00

ATOM 2575 C5B POP 276 25.950 21.920 65.030 1.00 0.00

ATOM 2576 NC3 POP 277 37.430 100.280 87.700 1.00 0.00

ATOM 2577 PO4 POP 277 36.740 104.790 85.550 1.00 0.00

ATOM 2578 GL1 POP 277 36.700 103.940 81.570 1.00 0.00

ATOM 2579 GL2 POP 277 34.360 105.050 80.280 1.00 0.00

ATOM 2580 C1A POP 277 34.470 107.930 77.320 1.00 0.00

ATOM 2581 C2A POP 277 35.610 112.120 74.340 1.00 0.00

ATOM 2582 C3A POP 277 31.810 115.250 73.120 1.00 0.00

ATOM 2583 C4A POP 277 31.940 118.630 71.490 1.00 0.00

ATOM 2584 C1B POP 277 39.160 101.060 79.180 1.00 0.00

ATOM 2585 C2B POP 277 40.160 99.170 75.160 1.00 0.00

ATOM 2586 D3B POP 277 38.880 97.100 72.670 1.00 0.00

ATOM 2587 C4B POP 277 36.690 94.140 74.320 1.00 0.00

ATOM 2588 C5B POP 277 33.230 92.470 71.680 1.00 0.00

ATOM 2589 NC3 POP 278 101.650 25.480 41.910 1.00 0.00

ATOM 2590 PO4 POP 278 102.000 21.970 44.400 1.00 0.00

ATOM 2591 GL1 POP 278 103.240 20.670 48.850 1.00 0.00

ATOM 2592 GL2 POP 278 100.020 19.960 49.610 1.00 0.00

ATOM 2593 C1A POP 278 98.260 19.880 53.870 1.00 0.00

ATOM 2594 C2A POP 278 96.430 20.100 58.060 1.00 0.00

ATOM 2595 C3A POP 278 96.490 20.240 61.750 1.00 0.00

ATOM 2596 C4A POP 278 94.280 21.310 65.960 1.00 0.00

ATOM 2597 C1B POP 278 104.940 23.850 51.210 1.00 0.00

ATOM 2598 C2B POP 278 105.890 24.390 55.390 1.00 0.00

ATOM 2599 D3B POP 278 103.720 26.850 59.190 1.00 0.00

ATOM 2600 C4B POP 278 106.190 30.590 60.180 1.00 0.00

ATOM 2601 C5B POP 278 110.390 32.610 58.940 1.00 0.00

ATOM 2602 NC3 POP 279 38.320 39.950 40.850 1.00 0.00

ATOM 2603 PO4 POP 279 36.220 41.290 44.080 1.00 0.00

ATOM 2604 GL1 POP 279 34.870 37.840 47.500 1.00 0.00

ATOM 2605 GL2 POP 279 36.490 34.490 48.370 1.00 0.00

ATOM 2606 C1A POP 279 36.900 34.860 54.110 1.00 0.00

ATOM 2607 C2A POP 279 34.970 37.230 56.880 1.00 0.00

ATOM 2608 C3A POP 279 33.660 34.150 59.470 1.00 0.00

ATOM 2609 C4A POP 279 30.290 36.630 61.890 1.00 0.00

ATOM 2610 C1B POP 279 33.140 36.810 51.390 1.00 0.00

ATOM 2611 C2B POP 279 29.550 39.180 53.380 1.00 0.00

ATOM 2612 D3B POP 279 26.400 40.010 55.870 1.00 0.00

ATOM 2613 C4B POP 279 26.750 39.130 59.550 1.00 0.00

ATOM 2614 C5B POP 279 26.090 38.600 64.000 1.00 0.00

ATOM 2615 NC3 POP 280 95.810 27.690 47.120 1.00 0.00

ATOM 2616 PO4 POP 280 94.720 31.840 45.100 1.00 0.00

ATOM 2617 GL1 POP 280 96.730 31.640 49.600 1.00 0.00

ATOM 2618 GL2 POP 280 98.540 29.610 50.700 1.00 0.00

ATOM 2619 C1A POP 280 96.820 29.060 54.540 1.00 0.00

ATOM 2620 C2A POP 280 96.250 32.220 57.870 1.00 0.00

ATOM 2621 C3A POP 280 97.280 34.050 62.120 1.00 0.00

ATOM 2622 C4A POP 280 98.770 35.000 65.700 1.00 0.00

ATOM 2623 C1B POP 280 98.960 34.690 52.100 1.00 0.00

ATOM 2624 C2B POP 280 98.730 36.450 57.430 1.00 0.00

ATOM 2625 D3B POP 280 102.780 35.430 59.430 1.00 0.00

ATOM 2626 C4B POP 280 106.400 36.200 58.260 1.00 0.00

ATOM 2627 C5B POP 280 110.090 36.980 55.830 1.00 0.00

ATOM 2628 NC3 POP 281 70.030 37.700 39.690 1.00 0.00

ATOM 2629 PO4 POP 281 67.860 38.350 44.000 1.00 0.00

ATOM 2630 GL1 POP 281 69.830 37.270 48.390 1.00 0.00

ATOM 2631 GL2 POP 281 72.290 39.230 47.800 1.00 0.00

ATOM 2632 C1A POP 281 71.920 41.290 51.800 1.00 0.00

ATOM 2633 C2A POP 281 70.320 44.920 55.810 1.00 0.00

ATOM 2634 C3A POP 281 70.370 47.850 57.200 1.00 0.00

ATOM 2635 C4A POP 281 69.330 52.510 58.310 1.00 0.00

ATOM 2636 C1B POP 281 69.580 36.690 53.220 1.00 0.00

ATOM 2637 C2B POP 281 71.930 37.950 56.690 1.00 0.00

ATOM 2638 D3B POP 281 71.320 39.150 61.080 1.00 0.00

ATOM 2639 C4B POP 281 68.470 42.100 62.910 1.00 0.00

ATOM 2640 C5B POP 281 67.360 41.260 66.850 1.00 0.00

ATOM 2641 NC3 POP 282 44.580 46.440 42.780 1.00 0.00

ATOM 2642 PO4 POP 282 47.100 46.770 46.750 1.00 0.00

ATOM 2643 GL1 POP 282 48.150 44.690 50.290 1.00 0.00

ATOM 2644 GL2 POP 282 51.190 45.150 51.020 1.00 0.00

ATOM 2645 C1A POP 282 50.800 48.130 54.450 1.00 0.00

ATOM 2646 C2A POP 282 47.920 48.530 57.040 1.00 0.00

ATOM 2647 C3A POP 282 46.340 51.670 58.830 1.00 0.00

ATOM 2648 C4A POP 282 45.860 54.390 62.990 1.00 0.00

ATOM 2649 C1B POP 282 48.860 42.390 55.270 1.00 0.00

ATOM 2650 C2B POP 282 51.730 42.770 60.230 1.00 0.00

ATOM 2651 D3B POP 282 54.480 44.480 64.200 1.00 0.00

ATOM 2652 C4B POP 282 55.250 47.860 66.820 1.00 0.00

ATOM 2653 C5B POP 282 58.910 47.470 69.890 1.00 0.00

ATOM 2654 NC3 POP 283 80.370 9.530 87.540 1.00 0.00

ATOM 2655 PO4 POP 283 81.920 9.660 83.270 1.00 0.00

ATOM 2656 GL1 POP 283 84.530 10.330 80.070 1.00 0.00

ATOM 2657 GL2 POP 283 86.270 13.120 80.460 1.00 0.00

ATOM 2658 C1A POP 283 88.630 14.660 76.580 1.00 0.00

ATOM 2659 C2A POP 283 93.020 14.410 76.580 1.00 0.00

ATOM 2660 C3A POP 283 95.830 11.180 77.600 1.00 0.00

ATOM 2661 C4A POP 283 97.340 6.720 79.420 1.00 0.00

ATOM 2662 C1B POP 283 88.620 9.530 77.800 1.00 0.00

ATOM 2663 C2B POP 283 90.470 8.460 73.640 1.00 0.00

ATOM 2664 D3B POP 283 94.430 8.040 70.840 1.00 0.00

ATOM 2665 C4B POP 283 93.590 8.910 66.570 1.00 0.00

ATOM 2666 C5B POP 283 91.120 10.920 63.220 1.00 0.00

ATOM 2667 NC3 POP 284 97.820 20.070 92.680 1.00 0.00

ATOM 2668 PO4 POP 284 97.890 16.160 89.670 1.00 0.00

ATOM 2669 GL1 POP 284 96.420 16.630 84.930 1.00 0.00

ATOM 2670 GL2 POP 284 97.200 12.720 85.650 1.00 0.00

ATOM 2671 C1A POP 284 99.170 12.300 81.810 1.00 0.00

ATOM 2672 C2A POP 284 100.960 16.520 79.610 1.00 0.00

ATOM 2673 C3A POP 284 104.660 17.960 80.870 1.00 0.00

ATOM 2674 C4A POP 284 106.740 18.580 84.150 1.00 0.00

ATOM 2675 C1B POP 284 96.190 17.080 80.520 1.00 0.00

ATOM 2676 C2B POP 284 97.550 17.300 76.480 1.00 0.00

ATOM 2677 D3B POP 284 97.490 19.490 74.020 1.00 0.00

ATOM 2678 C4B POP 284 97.240 17.240 69.960 1.00 0.00

ATOM 2679 C5B POP 284 96.840 16.290 65.050 1.00 0.00

ATOM 2680 NC3 POP 285 4.360 65.190 46.030 1.00 0.00

ATOM 2681 PO4 POP 285 2.520 62.070 48.810 1.00 0.00

ATOM 2682 GL1 POP 285 1.370 60.460 52.740 1.00 0.00

ATOM 2683 GL2 POP 285 -1.520 61.610 53.820 1.00 0.00

ATOM 2684 C1A POP 285 -2.950 59.230 57.960 1.00 0.00

ATOM 2685 C2A POP 285 -4.090 58.560 61.650 1.00 0.00

ATOM 2686 C3A POP 285 -1.340 56.400 65.240 1.00 0.00

ATOM 2687 C4A POP 285 0.780 55.650 69.320 1.00 0.00

ATOM 2688 C1B POP 285 2.330 60.000 56.360 1.00 0.00

ATOM 2689 C2B POP 285 1.790 62.000 60.570 1.00 0.00

ATOM 2690 D3B POP 285 0.030 62.310 64.230 1.00 0.00

ATOM 2691 C4B POP 285 2.150 60.180 66.610 1.00 0.00

ATOM 2692 C5B POP 285 3.960 60.460 69.730 1.00 0.00

ATOM 2693 NC3 POP 286 5.900 7.850 85.330 1.00 0.00

ATOM 2694 PO4 POP 286 7.410 11.330 87.660 1.00 0.00

ATOM 2695 GL1 POP 286 8.070 12.820 83.890 1.00 0.00

ATOM 2696 GL2 POP 286 10.650 13.110 82.370 1.00 0.00

ATOM 2697 C1A POP 286 9.050 14.110 77.970 1.00 0.00

ATOM 2698 C2A POP 286 7.830 13.920 74.820 1.00 0.00

ATOM 2699 C3A POP 286 8.340 12.100 70.340 1.00 0.00

ATOM 2700 C4A POP 286 8.480 12.540 65.770 1.00 0.00

ATOM 2701 C1B POP 286 5.810 14.870 81.230 1.00 0.00

ATOM 2702 C2B POP 286 4.940 18.520 79.610 1.00 0.00

ATOM 2703 D3B POP 286 3.250 21.260 76.660 1.00 0.00

ATOM 2704 C4B POP 286 5.040 19.840 72.390 1.00 0.00

ATOM 2705 C5B POP 286 7.960 17.440 71.200 1.00 0.00

ATOM 2706 NC3 POP 287 34.990 83.710 46.810 1.00 0.00

ATOM 2707 PO4 POP 287 36.980 79.980 47.040 1.00 0.00

ATOM 2708 GL1 POP 287 37.960 83.040 50.790 1.00 0.00

ATOM 2709 GL2 POP 287 38.150 79.560 52.550 1.00 0.00

ATOM 2710 C1A POP 287 38.410 78.440 56.080 1.00 0.00

ATOM 2711 C2A POP 287 40.590 78.850 58.730 1.00 0.00

ATOM 2712 C3A POP 287 43.520 80.980 60.740 1.00 0.00

ATOM 2713 C4A POP 287 46.500 84.750 60.870 1.00 0.00

ATOM 2714 C1B POP 287 39.440 83.570 54.700 1.00 0.00

ATOM 2715 C2B POP 287 40.000 84.830 59.000 1.00 0.00

ATOM 2716 D3B POP 287 39.070 85.230 63.680 1.00 0.00

ATOM 2717 C4B POP 287 42.570 83.010 65.590 1.00 0.00

ATOM 2718 C5B POP 287 45.000 84.020 68.810 1.00 0.00

ATOM 2719 NC3 POP 288 41.380 17.280 44.130 1.00 0.00

ATOM 2720 PO4 POP 288 37.890 15.040 45.300 1.00 0.00

ATOM 2721 GL1 POP 288 35.720 13.050 48.700 1.00 0.00

ATOM 2722 GL2 POP 288 32.290 13.780 49.780 1.00 0.00

ATOM 2723 C1A POP 288 30.080 11.370 51.410 1.00 0.00

ATOM 2724 C2A POP 288 26.390 8.680 50.940 1.00 0.00

ATOM 2725 C3A POP 288 24.090 6.290 54.350 1.00 0.00

ATOM 2726 C4A POP 288 22.780 6.140 58.290 1.00 0.00

ATOM 2727 C1B POP 288 34.860 11.280 53.340 1.00 0.00

ATOM 2728 C2B POP 288 33.650 7.820 56.860 1.00 0.00

ATOM 2729 D3B POP 288 35.390 6.780 60.430 1.00 0.00

ATOM 2730 C4B POP 288 33.450 9.300 63.040 1.00 0.00

ATOM 2731 C5B POP 288 29.830 11.300 66.680 1.00 0.00

ATOM 2732 NC3 POP 289 52.620 102.810 45.410 1.00 0.00

ATOM 2733 PO4 POP 289 53.520 99.200 47.940 1.00 0.00

ATOM 2734 GL1 POP 289 51.380 102.620 50.320 1.00 0.00

ATOM 2735 GL2 POP 289 48.840 104.430 51.180 1.00 0.00

ATOM 2736 C1A POP 289 51.580 105.020 54.860 1.00 0.00

ATOM 2737 C2A POP 289 51.330 104.070 59.330 1.00 0.00

ATOM 2738 C3A POP 289 51.850 104.450 63.870 1.00 0.00

ATOM 2739 C4A POP 289 53.530 100.630 66.180 1.00 0.00

ATOM 2740 C1B POP 289 50.230 100.610 54.640 1.00 0.00

ATOM 2741 C2B POP 289 48.530 98.890 58.200 1.00 0.00

ATOM 2742 D3B POP 289 50.300 98.540 61.540 1.00 0.00

ATOM 2743 C4B POP 289 49.110 99.220 66.330 1.00 0.00

ATOM 2744 C5B POP 289 51.240 98.960 70.240 1.00 0.00

ATOM 2745 NC3 POP 290 104.840 74.460 90.190 1.00 0.00

ATOM 2746 PO4 POP 290 104.600 70.890 88.380 1.00 0.00

ATOM 2747 GL1 POP 290 102.010 70.290 86.150 1.00 0.00

ATOM 2748 GL2 POP 290 103.530 67.120 83.440 1.00 0.00

ATOM 2749 C1A POP 290 103.910 69.030 79.270 1.00 0.00

ATOM 2750 C2A POP 290 104.960 69.830 76.160 1.00 0.00

ATOM 2751 C3A POP 290 105.810 73.180 72.950 1.00 0.00

ATOM 2752 C4A POP 290 108.770 75.750 70.440 1.00 0.00

ATOM 2753 C1B POP 290 100.650 71.610 82.010 1.00 0.00

ATOM 2754 C2B POP 290 97.530 71.910 79.210 1.00 0.00

ATOM 2755 D3B POP 290 97.790 72.340 75.060 1.00 0.00

ATOM 2756 C4B POP 290 101.350 69.730 72.400 1.00 0.00

ATOM 2757 C5B POP 290 105.320 67.790 71.690 1.00 0.00

ATOM 2758 NC3 POP 291 19.050 35.750 92.280 1.00 0.00

ATOM 2759 PO4 POP 291 18.740 32.650 88.800 1.00 0.00

ATOM 2760 GL1 POP 291 17.320 33.480 84.710 1.00 0.00

ATOM 2761 GL2 POP 291 14.500 35.180 86.030 1.00 0.00

ATOM 2762 C1A POP 291 13.700 35.310 81.860 1.00 0.00

ATOM 2763 C2A POP 291 15.100 35.570 78.080 1.00 0.00

ATOM 2764 C3A POP 291 17.660 32.340 75.180 1.00 0.00

ATOM 2765 C4A POP 291 17.180 27.580 74.980 1.00 0.00

ATOM 2766 C1B POP 291 18.720 35.880 81.600 1.00 0.00

ATOM 2767 C2B POP 291 21.270 35.420 78.090 1.00 0.00

ATOM 2768 D3B POP 291 24.410 35.390 73.970 1.00 0.00

ATOM 2769 C4B POP 291 27.690 37.740 72.180 1.00 0.00

ATOM 2770 C5B POP 291 27.210 41.240 68.550 1.00 0.00

ATOM 2771 NC3 POP 292 50.270 38.340 43.630 1.00 0.00

ATOM 2772 PO4 POP 292 52.760 36.880 46.210 1.00 0.00

ATOM 2773 GL1 POP 292 54.720 38.140 50.360 1.00 0.00

ATOM 2774 GL2 POP 292 52.500 39.820 51.280 1.00 0.00

ATOM 2775 C1A POP 292 53.940 41.480 54.760 1.00 0.00

ATOM 2776 C2A POP 292 51.640 38.750 57.110 1.00 0.00

ATOM 2777 C3A POP 292 53.780 37.520 60.710 1.00 0.00

ATOM 2778 C4A POP 292 54.580 33.220 61.950 1.00 0.00

ATOM 2779 C1B POP 292 55.370 36.470 54.000 1.00 0.00

ATOM 2780 C2B POP 292 56.340 35.990 56.510 1.00 0.00

ATOM 2781 D3B POP 292 59.150 35.010 59.670 1.00 0.00

ATOM 2782 C4B POP 292 59.430 37.660 62.600 1.00 0.00

ATOM 2783 C5B POP 292 56.630 40.130 63.840 1.00 0.00

ATOM 2784 NC3 POP 293 4.380 105.110 88.270 1.00 0.00

ATOM 2785 PO4 POP 293 6.560 104.490 85.100 1.00 0.00

ATOM 2786 GL1 POP 293 4.090 102.780 81.620 1.00 0.00

ATOM 2787 GL2 POP 293 5.950 103.950 79.210 1.00 0.00

ATOM 2788 C1A POP 293 4.580 103.550 74.970 1.00 0.00

ATOM 2789 C2A POP 293 2.880 103.080 70.690 1.00 0.00

ATOM 2790 C3A POP 293 0.060 103.650 67.350 1.00 0.00

ATOM 2791 C4A POP 293 -2.180 103.870 64.250 1.00 0.00

ATOM 2792 C1B POP 293 2.500 100.120 78.610 1.00 0.00

ATOM 2793 C2B POP 293 2.340 98.650 73.370 1.00 0.00

ATOM 2794 D3B POP 293 3.490 97.430 69.640 1.00 0.00

ATOM 2795 C4B POP 293 3.350 99.930 67.440 1.00 0.00

ATOM 2796 C5B POP 293 1.700 100.700 62.930 1.00 0.00

ATOM 2797 NC3 POP 294 0.730 73.590 47.000 1.00 0.00

ATOM 2798 PO4 POP 294 0.480 77.710 46.590 1.00 0.00

ATOM 2799 GL1 POP 294 1.690 78.200 51.060 1.00 0.00

ATOM 2800 GL2 POP 294 -0.960 77.430 52.140 1.00 0.00

ATOM 2801 C1A POP 294 -1.350 77.100 57.010 1.00 0.00

ATOM 2802 C2A POP 294 -0.550 78.650 61.170 1.00 0.00

ATOM 2803 C3A POP 294 1.060 82.510 61.530 1.00 0.00

ATOM 2804 C4A POP 294 3.740 85.920 61.780 1.00 0.00

ATOM 2805 C1B POP 294 2.560 81.790 52.920 1.00 0.00

ATOM 2806 C2B POP 294 1.820 83.360 57.050 1.00 0.00

ATOM 2807 D3B POP 294 -0.470 87.110 58.510 1.00 0.00

ATOM 2808 C4B POP 294 -0.710 87.020 62.760 1.00 0.00

ATOM 2809 C5B POP 294 0.490 85.660 65.900 1.00 0.00

ATOM 2810 NC3 POP 295 106.970 52.210 49.360 1.00 0.00

ATOM 2811 PO4 POP 295 109.020 47.930 46.960 1.00 0.00

ATOM 2812 GL1 POP 295 106.510 46.940 50.700 1.00 0.00

ATOM 2813 GL2 POP 295 103.530 48.740 50.930 1.00 0.00

ATOM 2814 C1A POP 295 103.200 48.370 55.420 1.00 0.00

ATOM 2815 C2A POP 295 105.040 46.310 59.620 1.00 0.00

ATOM 2816 C3A POP 295 107.790 46.580 62.390 1.00 0.00

ATOM 2817 C4A POP 295 109.560 47.450 65.880 1.00 0.00

ATOM 2818 C1B POP 295 104.860 43.690 53.710 1.00 0.00

ATOM 2819 C2B POP 295 107.070 41.750 56.970 1.00 0.00

ATOM 2820 D3B POP 295 107.800 42.150 60.470 1.00 0.00

ATOM 2821 C4B POP 295 107.050 38.960 62.680 1.00 0.00

ATOM 2822 C5B POP 295 108.820 34.900 62.850 1.00 0.00

ATOM 2823 NC3 POP 296 87.450 73.480 86.120 1.00 0.00

ATOM 2824 PO4 POP 296 84.140 76.080 85.810 1.00 0.00

ATOM 2825 GL1 POP 296 84.370 72.320 82.860 1.00 0.00

ATOM 2826 GL2 POP 296 80.690 73.990 80.530 1.00 0.00

ATOM 2827 C1A POP 296 81.660 75.010 76.570 1.00 0.00

ATOM 2828 C2A POP 296 81.780 75.880 72.450 1.00 0.00

ATOM 2829 C3A POP 296 84.250 76.310 69.310 1.00 0.00

ATOM 2830 C4A POP 296 86.710 78.930 66.370 1.00 0.00

ATOM 2831 C1B POP 296 84.390 71.210 79.080 1.00 0.00

ATOM 2832 C2B POP 296 86.390 68.310 75.640 1.00 0.00

ATOM 2833 D3B POP 296 85.070 66.120 72.670 1.00 0.00

ATOM 2834 C4B POP 296 82.200 64.330 74.240 1.00 0.00

ATOM 2835 C5B POP 296 78.150 63.520 76.520 1.00 0.00

ATOM 2836 NC3 POP 297 61.290 53.660 39.370 1.00 0.00

ATOM 2837 PO4 POP 297 59.160 55.070 41.940 1.00 0.00

ATOM 2838 GL1 POP 297 57.990 55.700 47.100 1.00 0.00

ATOM 2839 GL2 POP 297 61.140 55.790 49.230 1.00 0.00

ATOM 2840 C1A POP 297 61.890 54.800 52.710 1.00 0.00

ATOM 2841 C2A POP 297 60.680 53.550 56.180 1.00 0.00

ATOM 2842 C3A POP 297 59.110 52.420 60.160 1.00 0.00

ATOM 2843 C4A POP 297 56.500 51.660 63.990 1.00 0.00

ATOM 2844 C1B POP 297 56.280 56.290 50.940 1.00 0.00

ATOM 2845 C2B POP 297 54.570 56.910 55.000 1.00 0.00

ATOM 2846 D3B POP 297 55.220 55.660 59.540 1.00 0.00

ATOM 2847 C4B POP 297 58.750 58.430 62.980 1.00 0.00

ATOM 2848 C5B POP 297 60.140 62.380 64.820 1.00 0.00

ATOM 2849 NC3 POP 298 75.690 40.510 93.170 1.00 0.00

ATOM 2850 PO4 POP 298 74.690 38.440 89.120 1.00 0.00

ATOM 2851 GL1 POP 298 76.530 40.660 85.000 1.00 0.00

ATOM 2852 GL2 POP 298 79.730 42.130 83.170 1.00 0.00

ATOM 2853 C1A POP 298 78.830 39.960 78.890 1.00 0.00

ATOM 2854 C2A POP 298 81.590 38.230 75.300 1.00 0.00

ATOM 2855 C3A POP 298 83.830 37.290 70.620 1.00 0.00

ATOM 2856 C4A POP 298 86.880 37.080 67.120 1.00 0.00

ATOM 2857 C1B POP 298 74.290 40.160 80.860 1.00 0.00

ATOM 2858 C2B POP 298 73.160 36.680 77.990 1.00 0.00

ATOM 2859 D3B POP 298 73.980 32.800 75.820 1.00 0.00

ATOM 2860 C4B POP 298 71.250 31.150 72.060 1.00 0.00

ATOM 2861 C5B POP 298 70.110 32.400 67.980 1.00 0.00

ATOM 2862 NC3 POP 299 49.240 18.220 43.970 1.00 0.00

ATOM 2863 PO4 POP 299 45.010 18.280 41.500 1.00 0.00

ATOM 2864 GL1 POP 299 45.040 19.530 46.150 1.00 0.00

ATOM 2865 GL2 POP 299 45.550 16.780 47.230 1.00 0.00

ATOM 2866 C1A POP 299 49.040 16.430 50.910 1.00 0.00

ATOM 2867 C2A POP 299 51.080 16.700 55.520 1.00 0.00

ATOM 2868 C3A POP 299 53.890 16.630 58.980 1.00 0.00

ATOM 2869 C4A POP 299 57.330 20.570 58.530 1.00 0.00

ATOM 2870 C1B POP 299 45.360 20.450 50.890 1.00 0.00

ATOM 2871 C2B POP 299 43.340 23.270 54.370 1.00 0.00

ATOM 2872 D3B POP 299 42.420 24.080 58.330 1.00 0.00

ATOM 2873 C4B POP 299 39.800 27.840 60.040 1.00 0.00

ATOM 2874 C5B POP 299 37.080 31.200 60.450 1.00 0.00

ATOM 2875 NC3 POP 300 48.820 7.610 90.560 1.00 0.00

ATOM 2876 PO4 POP 300 48.240 9.790 87.030 1.00 0.00

ATOM 2877 GL1 POP 300 47.150 8.950 82.250 1.00 0.00

ATOM 2878 GL2 POP 300 43.600 7.800 81.730 1.00 0.00

ATOM 2879 C1A POP 300 45.560 5.270 77.800 1.00 0.00

ATOM 2880 C2A POP 300 47.330 1.450 76.190 1.00 0.00

ATOM 2881 C3A POP 300 49.740 -2.080 77.020 1.00 0.00

ATOM 2882 C4A POP 300 49.240 -6.230 77.830 1.00 0.00

ATOM 2883 C1B POP 300 45.890 9.960 77.300 1.00 0.00

ATOM 2884 C2B POP 300 45.830 11.990 73.970 1.00 0.00

ATOM 2885 D3B POP 300 45.450 15.910 73.930 1.00 0.00

ATOM 2886 C4B POP 300 42.990 16.960 70.250 1.00 0.00

ATOM 2887 C5B POP 300 40.500 18.490 67.170 1.00 0.00

ATOM 2888 NC3 POP 301 39.870 74.010 89.210 1.00 0.00

ATOM 2889 PO4 POP 301 40.200 77.490 88.430 1.00 0.00

ATOM 2890 GL1 POP 301 39.850 76.040 84.490 1.00 0.00

ATOM 2891 GL2 POP 301 43.300 76.770 83.370 1.00 0.00

ATOM 2892 C1A POP 301 47.100 74.720 80.130 1.00 0.00

ATOM 2893 C2A POP 301 46.150 71.590 76.220 1.00 0.00

ATOM 2894 C3A POP 301 46.060 70.470 71.330 1.00 0.00

ATOM 2895 C4A POP 301 41.860 69.900 70.190 1.00 0.00

ATOM 2896 C1B POP 301 38.700 78.320 79.840 1.00 0.00

ATOM 2897 C2B POP 301 37.460 76.310 76.800 1.00 0.00

ATOM 2898 D3B POP 301 35.060 77.210 72.890 1.00 0.00

ATOM 2899 C4B POP 301 37.490 79.510 71.470 1.00 0.00

ATOM 2900 C5B POP 301 40.860 77.540 71.870 1.00 0.00

ATOM 2901 NC3 POP 302 62.940 7.020 39.940 1.00 0.00

ATOM 2902 PO4 POP 302 63.160 8.800 43.990 1.00 0.00

ATOM 2903 GL1 POP 302 62.420 8.580 48.360 1.00 0.00

ATOM 2904 GL2 POP 302 59.450 8.880 49.140 1.00 0.00

ATOM 2905 C1A POP 302 59.000 10.910 53.000 1.00 0.00

ATOM 2906 C2A POP 302 57.260 14.630 55.240 1.00 0.00

ATOM 2907 C3A POP 302 58.700 15.470 58.690 1.00 0.00

ATOM 2908 C4A POP 302 57.700 16.620 62.260 1.00 0.00

ATOM 2909 C1B POP 302 64.440 10.070 52.460 1.00 0.00

ATOM 2910 C2B POP 302 65.460 9.050 56.750 1.00 0.00

ATOM 2911 D3B POP 302 65.160 6.390 60.570 1.00 0.00

ATOM 2912 C4B POP 302 62.730 9.320 63.600 1.00 0.00

ATOM 2913 C5B POP 302 59.710 11.430 63.750 1.00 0.00

ATOM 2914 NC3 POP 303 24.030 79.280 90.460 1.00 0.00

ATOM 2915 PO4 POP 303 19.250 79.700 91.070 1.00 0.00

ATOM 2916 GL1 POP 303 16.310 76.290 87.690 1.00 0.00

ATOM 2917 GL2 POP 303 15.780 73.530 87.070 1.00 0.00

ATOM 2918 C1A POP 303 15.810 70.540 84.520 1.00 0.00

ATOM 2919 C2A POP 303 13.920 67.160 82.990 1.00 0.00

ATOM 2920 C3A POP 303 12.770 66.310 79.260 1.00 0.00

ATOM 2921 C4A POP 303 13.970 65.030 75.610 1.00 0.00

ATOM 2922 C1B POP 303 15.500 75.710 82.390 1.00 0.00

ATOM 2923 C2B POP 303 15.690 72.250 77.650 1.00 0.00

ATOM 2924 D3B POP 303 14.030 70.830 73.980 1.00 0.00

ATOM 2925 C4B POP 303 10.270 68.560 75.150 1.00 0.00

ATOM 2926 C5B POP 303 8.540 68.740 79.040 1.00 0.00

ATOM 2927 NC3 POP 304 16.420 37.590 46.880 1.00 0.00

ATOM 2928 PO4 POP 304 16.870 42.000 47.890 1.00 0.00

ATOM 2929 GL1 POP 304 16.040 40.470 51.740 1.00 0.00

ATOM 2930 GL2 POP 304 17.720 38.000 51.520 1.00 0.00

ATOM 2931 C1A POP 304 17.620 38.290 56.130 1.00 0.00

ATOM 2932 C2A POP 304 15.340 35.910 58.530 1.00 0.00

ATOM 2933 C3A POP 304 15.800 34.430 62.000 1.00 0.00

ATOM 2934 C4A POP 304 17.560 35.320 66.240 1.00 0.00

ATOM 2935 C1B POP 304 14.000 42.130 56.270 1.00 0.00

ATOM 2936 C2B POP 304 15.750 41.070 60.570 1.00 0.00

ATOM 2937 D3B POP 304 18.150 39.370 61.830 1.00 0.00

ATOM 2938 C4B POP 304 21.540 39.300 63.190 1.00 0.00

ATOM 2939 C5B POP 304 22.140 42.090 66.540 1.00 0.00

ATOM 2940 NC3 POP 305 30.890 75.900 96.530 1.00 0.00

ATOM 2941 PO4 POP 305 29.060 79.880 93.030 1.00 0.00

ATOM 2942 GL1 POP 305 28.640 78.150 88.050 1.00 0.00

ATOM 2943 GL2 POP 305 31.510 78.430 86.100 1.00 0.00

ATOM 2944 C1A POP 305 34.040 77.010 82.880 1.00 0.00

ATOM 2945 C2A POP 305 37.230 73.640 81.390 1.00 0.00

ATOM 2946 C3A POP 305 36.880 69.580 78.940 1.00 0.00

ATOM 2947 C4A POP 305 36.390 67.320 76.130 1.00 0.00

ATOM 2948 C1B POP 305 27.080 77.470 84.030 1.00 0.00

ATOM 2949 C2B POP 305 24.730 78.130 80.830 1.00 0.00

ATOM 2950 D3B POP 305 26.070 81.210 77.980 1.00 0.00

ATOM 2951 C4B POP 305 29.550 82.320 77.020 1.00 0.00

ATOM 2952 C5B POP 305 32.240 85.870 76.560 1.00 0.00

ATOM 2953 NC3 POP 306 6.600 87.250 39.860 1.00 0.00

ATOM 2954 PO4 POP 306 4.150 90.750 42.210 1.00 0.00

ATOM 2955 GL1 POP 306 3.380 93.030 45.220 1.00 0.00

ATOM 2956 GL2 POP 306 6.640 92.990 45.900 1.00 0.00

ATOM 2957 C1A POP 306 8.890 89.890 49.040 1.00 0.00

ATOM 2958 C2A POP 306 8.490 89.340 53.300 1.00 0.00

ATOM 2959 C3A POP 306 10.940 88.310 55.750 1.00 0.00

ATOM 2960 C4A POP 306 11.680 84.940 58.330 1.00 0.00

ATOM 2961 C1B POP 306 3.990 94.370 49.950 1.00 0.00

ATOM 2962 C2B POP 306 2.870 93.790 53.550 1.00 0.00

ATOM 2963 D3B POP 306 1.170 94.610 57.030 1.00 0.00

ATOM 2964 C4B POP 306 2.010 97.050 60.160 1.00 0.00

ATOM 2965 C5B POP 306 4.130 95.930 64.610 1.00 0.00

ATOM 2966 NC3 POP 307 31.210 16.660 89.740 1.00 0.00

ATOM 2967 PO4 POP 307 33.450 18.010 87.210 1.00 0.00

ATOM 2968 GL1 POP 307 32.310 18.190 82.960 1.00 0.00

ATOM 2969 GL2 POP 307 33.540 20.430 81.990 1.00 0.00

ATOM 2970 C1A POP 307 33.020 20.690 77.140 1.00 0.00

ATOM 2971 C2A POP 307 34.630 22.360 73.530 1.00 0.00

ATOM 2972 C3A POP 307 36.550 21.600 69.200 1.00 0.00

ATOM 2973 C4A POP 307 39.620 23.310 67.440 1.00 0.00

ATOM 2974 C1B POP 307 30.130 16.920 78.770 1.00 0.00

ATOM 2975 C2B POP 307 28.460 16.650 74.820 1.00 0.00

ATOM 2976 D3B POP 307 24.860 17.480 73.080 1.00 0.00

ATOM 2977 C4B POP 307 23.000 21.470 72.550 1.00 0.00

ATOM 2978 C5B POP 307 20.780 25.700 72.700 1.00 0.00

ATOM 2979 NC3 POP 308 22.250 102.380 43.830 1.00 0.00

ATOM 2980 PO4 POP 308 17.770 103.850 42.270 1.00 0.00

ATOM 2981 GL1 POP 308 16.670 103.350 46.470 1.00 0.00

ATOM 2982 GL2 POP 308 19.300 100.990 47.310 1.00 0.00

ATOM 2983 C1A POP 308 19.610 97.080 49.020 1.00 0.00

ATOM 2984 C2A POP 308 20.040 93.150 51.610 1.00 0.00

ATOM 2985 C3A POP 308 19.480 92.420 55.320 1.00 0.00

ATOM 2986 C4A POP 308 21.510 92.440 58.920 1.00 0.00

ATOM 2987 C1B POP 308 15.070 102.220 50.980 1.00 0.00

ATOM 2988 C2B POP 308 15.080 101.060 55.530 1.00 0.00

ATOM 2989 D3B POP 308 13.410 97.650 58.280 1.00 0.00

ATOM 2990 C4B POP 308 13.580 98.770 62.170 1.00 0.00

ATOM 2991 C5B POP 308 12.030 100.560 65.920 1.00 0.00

ATOM 2992 NC3 POP 309 74.470 -0.180 90.220 1.00 0.00

ATOM 2993 PO4 POP 309 77.200 0.800 86.800 1.00 0.00

ATOM 2994 GL1 POP 309 78.620 -0.100 82.680 1.00 0.00

ATOM 2995 GL2 POP 309 76.780 -2.280 82.600 1.00 0.00

ATOM 2996 C1A POP 309 77.400 -3.410 78.690 1.00 0.00

ATOM 2997 C2A POP 309 76.840 -3.100 75.360 1.00 0.00

ATOM 2998 C3A POP 309 76.150 -2.580 70.100 1.00 0.00

ATOM 2999 C4A POP 309 76.540 0.620 66.190 1.00 0.00

ATOM 3000 C1B POP 309 78.960 2.090 78.350 1.00 0.00

ATOM 3001 C2B POP 309 79.420 1.270 73.940 1.00 0.00

ATOM 3002 D3B POP 309 80.790 1.290 70.090 1.00 0.00

ATOM 3003 C4B POP 309 79.740 4.580 68.040 1.00 0.00

ATOM 3004 C5B POP 309 82.310 5.830 64.930 1.00 0.00

ATOM 3005 NC3 POP 310 20.960 61.560 47.430 1.00 0.00

ATOM 3006 PO4 POP 310 22.230 57.120 48.210 1.00 0.00

ATOM 3007 GL1 POP 310 20.240 59.350 51.660 1.00 0.00

ATOM 3008 GL2 POP 310 22.350 62.020 52.260 1.00 0.00

ATOM 3009 C1A POP 310 23.440 62.030 56.890 1.00 0.00

ATOM 3010 C2A POP 310 24.980 60.690 61.440 1.00 0.00

ATOM 3011 C3A POP 310 24.460 61.580 65.360 1.00 0.00

ATOM 3012 C4A POP 310 20.900 58.550 67.280 1.00 0.00

ATOM 3013 C1B POP 310 19.850 59.160 56.410 1.00 0.00

ATOM 3014 C2B POP 310 18.360 58.180 61.960 1.00 0.00

ATOM 3015 D3B POP 310 20.400 54.060 64.590 1.00 0.00

ATOM 3016 C4B POP 310 22.660 51.310 66.820 1.00 0.00

ATOM 3017 C5B POP 310 25.950 51.400 69.930 1.00 0.00

ATOM 3018 NC3 POP 311 39.740 106.200 46.410 1.00 0.00

ATOM 3019 PO4 POP 311 44.530 103.650 48.230 1.00 0.00

ATOM 3020 GL1 POP 311 43.360 104.080 51.520 1.00 0.00

ATOM 3021 GL2 POP 311 39.660 103.510 50.900 1.00 0.00

ATOM 3022 C1A POP 311 37.550 103.860 54.550 1.00 0.00

ATOM 3023 C2A POP 311 36.200 101.970 58.670 1.00 0.00

ATOM 3024 C3A POP 311 34.410 101.540 61.420 1.00 0.00

ATOM 3025 C4A POP 311 33.230 104.760 63.400 1.00 0.00

ATOM 3026 C1B POP 311 42.100 105.360 54.550 1.00 0.00

ATOM 3027 C2B POP 311 40.580 104.940 58.170 1.00 0.00

ATOM 3028 D3B POP 311 39.710 102.230 62.040 1.00 0.00

ATOM 3029 C4B POP 311 37.900 103.440 65.620 1.00 0.00

ATOM 3030 C5B POP 311 33.970 101.590 67.030 1.00 0.00

ATOM 3031 NC3 POP 312 30.670 17.820 40.790 1.00 0.00

ATOM 3032 PO4 POP 312 32.820 16.700 45.100 1.00 0.00

ATOM 3033 GL1 POP 312 35.390 18.060 48.650 1.00 0.00

ATOM 3034 GL2 POP 312 39.210 17.860 49.610 1.00 0.00

ATOM 3035 C1A POP 312 37.140 15.740 53.150 1.00 0.00

ATOM 3036 C2A POP 312 34.890 16.740 56.360 1.00 0.00

ATOM 3037 C3A POP 312 35.340 18.750 59.890 1.00 0.00

ATOM 3038 C4A POP 312 35.550 20.310 64.300 1.00 0.00

ATOM 3039 C1B POP 312 35.020 20.560 52.440 1.00 0.00

ATOM 3040 C2B POP 312 31.100 21.190 54.160 1.00 0.00

ATOM 3041 D3B POP 312 30.440 19.760 57.670 1.00 0.00

ATOM 3042 C4B POP 312 30.980 21.730 62.160 1.00 0.00

ATOM 3043 C5B POP 312 31.480 21.490 66.680 1.00 0.00

ATOM 3044 NC3 POP 313 65.840 18.590 43.150 1.00 0.00

ATOM 3045 PO4 POP 313 69.330 20.680 43.920 1.00 0.00

ATOM 3046 GL1 POP 313 69.380 19.780 47.880 1.00 0.00

ATOM 3047 GL2 POP 313 71.680 18.140 48.230 1.00 0.00

ATOM 3048 C1A POP 313 74.770 18.780 51.560 1.00 0.00

ATOM 3049 C2A POP 313 76.970 15.910 54.040 1.00 0.00

ATOM 3050 C3A POP 313 79.130 15.470 58.360 1.00 0.00

ATOM 3051 C4A POP 313 76.940 17.870 62.830 1.00 0.00

ATOM 3052 C1B POP 313 68.140 19.960 53.020 1.00 0.00

ATOM 3053 C2B POP 313 67.660 19.890 57.210 1.00 0.00

ATOM 3054 D3B POP 313 66.290 18.860 61.930 1.00 0.00

ATOM 3055 C4B POP 313 65.320 20.590 66.850 1.00 0.00

ATOM 3056 C5B POP 313 66.190 25.000 67.930 1.00 0.00

ATOM 3057 NC3 POP 314 46.230 9.190 46.010 1.00 0.00

ATOM 3058 PO4 POP 314 44.370 12.830 44.530 1.00 0.00

ATOM 3059 GL1 POP 314 41.730 13.400 48.280 1.00 0.00

ATOM 3060 GL2 POP 314 45.300 12.600 49.740 1.00 0.00

ATOM 3061 C1A POP 314 44.550 15.280 52.350 1.00 0.00

ATOM 3062 C2A POP 314 41.820 14.780 55.250 1.00 0.00

ATOM 3063 C3A POP 314 40.060 16.150 59.460 1.00 0.00

ATOM 3064 C4A POP 314 37.330 15.120 63.070 1.00 0.00

ATOM 3065 C1B POP 314 39.630 11.340 52.300 1.00 0.00

ATOM 3066 C2B POP 314 38.600 11.700 56.710 1.00 0.00

ATOM 3067 D3B POP 314 35.300 13.160 59.210 1.00 0.00

ATOM 3068 C4B POP 314 31.120 12.730 57.210 1.00 0.00

ATOM 3069 C5B POP 314 28.750 15.280 54.270 1.00 0.00

ATOM 3070 NC3 POP 315 58.510 93.200 85.790 1.00 0.00

ATOM 3071 PO4 POP 315 58.220 97.020 82.880 1.00 0.00

ATOM 3072 GL1 POP 315 59.250 97.700 79.550 1.00 0.00

ATOM 3073 GL2 POP 315 57.020 99.570 78.230 1.00 0.00

ATOM 3074 C1A POP 315 57.910 99.180 73.410 1.00 0.00

ATOM 3075 C2A POP 315 55.290 101.830 70.380 1.00 0.00

ATOM 3076 C3A POP 315 56.110 104.820 66.800 1.00 0.00

ATOM 3077 C4A POP 315 54.470 108.710 66.370 1.00 0.00

ATOM 3078 C1B POP 315 62.650 99.280 76.630 1.00 0.00

ATOM 3079 C2B POP 315 63.220 101.100 72.820 1.00 0.00

ATOM 3080 D3B POP 315 63.860 105.250 70.810 1.00 0.00

ATOM 3081 C4B POP 315 66.570 108.560 71.000 1.00 0.00

ATOM 3082 C5B POP 315 65.480 112.050 74.040 1.00 0.00

ATOM 3083 NC3 POP 316 82.620 89.860 90.820 1.00 0.00

ATOM 3084 PO4 POP 316 84.330 86.110 89.150 1.00 0.00

ATOM 3085 GL1 POP 316 85.800 84.920 85.560 1.00 0.00

ATOM 3086 GL2 POP 316 87.410 81.770 84.080 1.00 0.00

ATOM 3087 C1A POP 316 88.790 81.800 80.870 1.00 0.00

ATOM 3088 C2A POP 316 89.080 81.910 76.080 1.00 0.00

ATOM 3089 C3A POP 316 88.950 82.750 72.220 1.00 0.00

ATOM 3090 C4A POP 316 87.570 83.880 68.650 1.00 0.00

ATOM 3091 C1B POP 316 85.550 85.790 81.160 1.00 0.00

ATOM 3092 C2B POP 316 85.000 83.910 77.470 1.00 0.00

ATOM 3093 D3B POP 316 83.540 85.000 73.170 1.00 0.00

ATOM 3094 C4B POP 316 83.390 81.310 70.160 1.00 0.00

ATOM 3095 C5B POP 316 81.020 78.900 66.530 1.00 0.00

ATOM 3096 NC3 POP 317 15.010 21.240 87.390 1.00 0.00

ATOM 3097 PO4 POP 317 18.690 19.260 84.160 1.00 0.00

ATOM 3098 GL1 POP 317 15.990 20.310 81.110 1.00 0.00

ATOM 3099 GL2 POP 317 15.310 22.810 82.570 1.00 0.00

ATOM 3100 C1A POP 317 13.300 22.940 77.750 1.00 0.00

ATOM 3101 C2A POP 317 11.510 26.290 75.570 1.00 0.00

ATOM 3102 C3A POP 317 9.610 27.380 72.290 1.00 0.00

ATOM 3103 C4A POP 317 6.620 25.980 68.660 1.00 0.00

ATOM 3104 C1B POP 317 17.820 21.920 76.530 1.00 0.00

ATOM 3105 C2B POP 317 17.880 20.540 71.960 1.00 0.00

ATOM 3106 D3B POP 317 17.460 17.140 69.080 1.00 0.00

ATOM 3107 C4B POP 317 20.930 16.870 68.760 1.00 0.00

ATOM 3108 C5B POP 317 24.230 15.080 64.950 1.00 0.00

ATOM 3109 NC3 POP 318 15.960 32.800 44.350 1.00 0.00

ATOM 3110 PO4 POP 318 19.550 35.360 43.800 1.00 0.00

ATOM 3111 GL1 POP 318 19.660 33.990 48.040 1.00 0.00

ATOM 3112 GL2 POP 318 21.810 31.950 47.820 1.00 0.00

ATOM 3113 C1A POP 318 23.250 31.130 52.030 1.00 0.00

ATOM 3114 C2A POP 318 27.220 29.030 53.480 1.00 0.00

ATOM 3115 C3A POP 318 28.140 28.400 57.230 1.00 0.00

ATOM 3116 C4A POP 318 29.940 25.100 58.230 1.00 0.00

ATOM 3117 C1B POP 318 19.080 33.750 52.760 1.00 0.00

ATOM 3118 C2B POP 318 18.370 31.560 56.050 1.00 0.00

ATOM 3119 D3B POP 318 19.960 30.620 60.060 1.00 0.00

ATOM 3120 C4B POP 318 19.080 30.300 64.440 1.00 0.00

ATOM 3121 C5B POP 318 18.660 28.330 68.660 1.00 0.00

ATOM 3122 NC3 POP 319 30.050 34.810 87.930 1.00 0.00

ATOM 3123 PO4 POP 319 31.270 37.460 85.450 1.00 0.00

ATOM 3124 GL1 POP 319 33.850 38.050 81.570 1.00 0.00

ATOM 3125 GL2 POP 319 31.240 37.320 79.600 1.00 0.00

ATOM 3126 C1A POP 319 32.240 38.180 75.340 1.00 0.00

ATOM 3127 C2A POP 319 32.230 39.380 71.620 1.00 0.00

ATOM 3128 C3A POP 319 31.580 43.360 68.770 1.00 0.00

ATOM 3129 C4A POP 319 31.110 46.880 67.590 1.00 0.00

ATOM 3130 C1B POP 319 35.640 39.650 78.840 1.00 0.00

ATOM 3131 C2B POP 319 36.510 40.040 74.610 1.00 0.00

ATOM 3132 D3B POP 319 37.680 42.710 70.810 1.00 0.00

ATOM 3133 C4B POP 319 35.810 45.590 69.780 1.00 0.00

ATOM 3134 C5B POP 319 33.240 48.690 72.370 1.00 0.00

ATOM 3135 NC3 POP 320 77.240 24.710 87.930 1.00 0.00

ATOM 3136 PO4 POP 320 76.820 28.980 86.280 1.00 0.00

ATOM 3137 GL1 POP 320 73.190 31.230 83.900 1.00 0.00

ATOM 3138 GL2 POP 320 74.050 35.720 83.420 1.00 0.00

ATOM 3139 C1A POP 320 76.980 35.600 80.890 1.00 0.00

ATOM 3140 C2A POP 320 77.910 35.210 76.880 1.00 0.00

ATOM 3141 C3A POP 320 79.250 33.250 73.060 1.00 0.00

ATOM 3142 C4A POP 320 82.870 31.850 69.990 1.00 0.00

ATOM 3143 C1B POP 320 71.470 32.420 80.030 1.00 0.00

ATOM 3144 C2B POP 320 69.230 30.530 76.900 1.00 0.00

ATOM 3145 D3B POP 320 68.340 26.200 76.640 1.00 0.00

ATOM 3146 C4B POP 320 71.320 23.850 78.980 1.00 0.00

ATOM 3147 C5B POP 320 74.470 26.090 78.970 1.00 0.00

ATOM 3148 NC3 POP 321 108.090 6.810 91.140 1.00 0.00

ATOM 3149 PO4 POP 321 104.530 8.480 88.200 1.00 0.00

ATOM 3150 GL1 POP 321 103.620 9.640 84.330 1.00 0.00

ATOM 3151 GL2 POP 321 103.130 7.650 81.540 1.00 0.00

ATOM 3152 C1A POP 321 101.680 9.620 77.760 1.00 0.00

ATOM 3153 C2A POP 321 102.570 10.990 73.460 1.00 0.00

ATOM 3154 C3A POP 321 101.420 9.380 68.790 1.00 0.00

ATOM 3155 C4A POP 321 101.860 6.070 65.440 1.00 0.00

ATOM 3156 C1B POP 321 104.410 12.520 80.340 1.00 0.00

ATOM 3157 C2B POP 321 104.020 15.220 76.360 1.00 0.00

ATOM 3158 D3B POP 321 100.890 15.650 72.810 1.00 0.00

ATOM 3159 C4B POP 321 97.950 12.720 73.740 1.00 0.00

ATOM 3160 C5B POP 321 98.160 7.980 74.000 1.00 0.00

ATOM 3161 NC3 POP 322 96.590 63.860 93.920 1.00 0.00

ATOM 3162 PO4 POP 322 99.100 60.680 91.350 1.00 0.00

ATOM 3163 GL1 POP 322 98.470 61.590 87.130 1.00 0.00

ATOM 3164 GL2 POP 322 97.190 59.510 85.970 1.00 0.00

ATOM 3165 C1A POP 322 94.680 56.500 83.200 1.00 0.00

ATOM 3166 C2A POP 322 95.040 54.350 79.620 1.00 0.00

ATOM 3167 C3A POP 322 96.670 55.210 74.340 1.00 0.00

ATOM 3168 C4A POP 322 99.140 56.670 71.450 1.00 0.00

ATOM 3169 C1B POP 322 99.350 61.390 82.300 1.00 0.00

ATOM 3170 C2B POP 322 100.700 62.230 77.800 1.00 0.00

ATOM 3171 D3B POP 322 102.830 65.340 75.500 1.00 0.00

ATOM 3172 C4B POP 322 106.290 63.310 72.570 1.00 0.00

ATOM 3173 C5B POP 322 109.890 59.590 72.880 1.00 0.00

ATOM 3174 NC3 POP 323 22.670 -0.770 84.740 1.00 0.00

ATOM 3175 PO4 POP 323 25.130 -3.030 83.540 1.00 0.00

ATOM 3176 GL1 POP 323 26.330 0.050 80.930 1.00 0.00

ATOM 3177 GL2 POP 323 26.960 2.640 79.200 1.00 0.00

ATOM 3178 C1A POP 323 25.330 5.080 74.250 1.00 0.00

ATOM 3179 C2A POP 323 22.940 6.590 69.920 1.00 0.00

ATOM 3180 C3A POP 323 23.260 5.390 65.710 1.00 0.00

ATOM 3181 C4A POP 323 25.420 4.270 62.400 1.00 0.00

ATOM 3182 C1B POP 323 25.090 0.090 75.410 1.00 0.00

ATOM 3183 C2B POP 323 24.840 0.910 71.230 1.00 0.00

ATOM 3184 D3B POP 323 26.950 1.420 66.110 1.00 0.00

ATOM 3185 C4B POP 323 28.310 -2.410 65.020 1.00 0.00

ATOM 3186 C5B POP 323 25.180 -5.810 63.080 1.00 0.00

ATOM 3187 NC3 POP 324 42.790 29.280 87.100 1.00 0.00

ATOM 3188 PO4 POP 324 46.870 27.640 86.140 1.00 0.00

ATOM 3189 GL1 POP 324 47.960 26.320 81.650 1.00 0.00

ATOM 3190 GL2 POP 324 47.830 22.550 82.220 1.00 0.00

ATOM 3191 C1A POP 324 50.190 20.600 78.350 1.00 0.00

ATOM 3192 C2A POP 324 53.170 19.420 75.690 1.00 0.00

ATOM 3193 C3A POP 324 57.250 21.640 74.170 1.00 0.00

ATOM 3194 C4A POP 324 61.430 21.240 71.240 1.00 0.00

ATOM 3195 C1B POP 324 46.460 25.170 76.880 1.00 0.00

ATOM 3196 C2B POP 324 44.480 24.890 72.570 1.00 0.00

ATOM 3197 D3B POP 324 43.420 21.930 70.540 1.00 0.00

ATOM 3198 C4B POP 324 43.920 22.360 65.530 1.00 0.00

ATOM 3199 C5B POP 324 46.120 22.990 61.700 1.00 0.00

ATOM 3200 NC3 POP 325 37.520 50.410 45.660 1.00 0.00

ATOM 3201 PO4 POP 325 40.210 47.270 44.920 1.00 0.00

ATOM 3202 GL1 POP 325 36.650 45.980 48.070 1.00 0.00

ATOM 3203 GL2 POP 325 33.640 44.500 47.190 1.00 0.00

ATOM 3204 C1A POP 325 32.390 43.180 51.500 1.00 0.00

ATOM 3205 C2A POP 325 29.120 43.970 54.000 1.00 0.00

ATOM 3206 C3A POP 325 24.440 45.410 55.400 1.00 0.00

ATOM 3207 C4A POP 325 23.310 48.970 54.340 1.00 0.00

ATOM 3208 C1B POP 325 37.450 45.460 52.240 1.00 0.00

ATOM 3209 C2B POP 325 40.460 45.180 56.710 1.00 0.00

ATOM 3210 D3B POP 325 42.380 48.600 58.370 1.00 0.00

ATOM 3211 C4B POP 325 43.850 48.970 62.400 1.00 0.00

ATOM 3212 C5B POP 325 46.140 48.780 66.860 1.00 0.00

ATOM 3213 NC3 POP 326 106.830 93.090 88.360 1.00 0.00

ATOM 3214 PO4 POP 326 102.790 95.530 89.790 1.00 0.00

ATOM 3215 GL1 POP 326 103.550 95.630 84.850 1.00 0.00

ATOM 3216 GL2 POP 326 105.230 97.590 83.510 1.00 0.00

ATOM 3217 C1A POP 326 103.700 98.180 80.040 1.00 0.00

ATOM 3218 C2A POP 326 103.190 95.200 77.030 1.00 0.00

ATOM 3219 C3A POP 326 101.390 95.420 71.740 1.00 0.00

ATOM 3220 C4A POP 326 103.340 95.770 68.210 1.00 0.00

ATOM 3221 C1B POP 326 106.270 93.170 80.960 1.00 0.00

ATOM 3222 C2B POP 326 107.140 92.400 76.010 1.00 0.00

ATOM 3223 D3B POP 326 110.170 94.250 72.370 1.00 0.00

ATOM 3224 C4B POP 326 111.980 91.880 70.420 1.00 0.00

ATOM 3225 C5B POP 326 112.440 89.730 66.120 1.00 0.00

ATOM 3226 NC3 POP 327 23.990 99.870 36.120 1.00 0.00

ATOM 3227 PO4 POP 327 25.340 99.860 40.520 1.00 0.00

ATOM 3228 GL1 POP 327 26.610 100.190 44.880 1.00 0.00

ATOM 3229 GL2 POP 327 29.870 101.840 44.630 1.00 0.00

ATOM 3230 C1A POP 327 29.660 101.270 49.110 1.00 0.00

ATOM 3231 C2A POP 327 27.480 104.130 51.880 1.00 0.00

ATOM 3232 C3A POP 327 29.840 106.550 54.860 1.00 0.00

ATOM 3233 C4A POP 327 29.840 110.930 56.540 1.00 0.00

ATOM 3234 C1B POP 327 24.590 99.720 49.160 1.00 0.00

ATOM 3235 C2B POP 327 21.230 100.590 51.980 1.00 0.00

ATOM 3236 D3B POP 327 19.980 102.290 54.990 1.00 0.00

ATOM 3237 C4B POP 327 20.030 103.010 59.180 1.00 0.00

ATOM 3238 C5B POP 327 19.680 104.050 63.440 1.00 0.00

ATOM 3239 NC3 POP 328 11.710 23.500 45.740 1.00 0.00

ATOM 3240 PO4 POP 328 9.340 26.410 44.650 1.00 0.00

ATOM 3241 GL1 POP 328 9.970 26.410 49.280 1.00 0.00

ATOM 3242 GL2 POP 328 13.840 25.380 49.610 1.00 0.00

ATOM 3243 C1A POP 328 15.430 25.670 54.280 1.00 0.00

ATOM 3244 C2A POP 328 17.690 25.510 58.890 1.00 0.00

ATOM 3245 C3A POP 328 18.470 25.420 63.410 1.00 0.00

ATOM 3246 C4A POP 328 16.510 23.020 67.730 1.00 0.00

ATOM 3247 C1B POP 328 10.270 25.830 53.930 1.00 0.00

ATOM 3248 C2B POP 328 9.830 26.180 58.250 1.00 0.00

ATOM 3249 D3B POP 328 8.080 27.550 62.070 1.00 0.00

ATOM 3250 C4B POP 328 7.510 22.560 64.650 1.00 0.00

ATOM 3251 C5B POP 328 5.150 19.640 67.780 1.00 0.00

ATOM 3252 NC3 POP 329 82.450 74.930 89.990 1.00 0.00

ATOM 3253 PO4 POP 329 84.730 70.740 88.820 1.00 0.00

ATOM 3254 GL1 POP 329 84.790 67.390 85.650 1.00 0.00

ATOM 3255 GL2 POP 329 82.920 63.840 86.100 1.00 0.00

ATOM 3256 C1A POP 329 83.090 64.530 82.030 1.00 0.00

ATOM 3257 C2A POP 329 81.870 66.960 78.520 1.00 0.00

ATOM 3258 C3A POP 329 81.630 69.820 74.670 1.00 0.00

ATOM 3259 C4A POP 329 85.650 73.010 73.560 1.00 0.00

ATOM 3260 C1B POP 329 87.590 68.450 81.610 1.00 0.00

ATOM 3261 C2B POP 329 88.990 71.840 79.330 1.00 0.00

ATOM 3262 D3B POP 329 91.140 71.610 74.840 1.00 0.00

ATOM 3263 C4B POP 329 89.540 69.660 71.570 1.00 0.00

ATOM 3264 C5B POP 329 85.800 70.170 68.350 1.00 0.00

ATOM 3265 NC3 POP 330 75.060 89.520 43.570 1.00 0.00

ATOM 3266 PO4 POP 330 76.280 92.520 45.540 1.00 0.00

ATOM 3267 GL1 POP 330 76.570 89.240 49.680 1.00 0.00

ATOM 3268 GL2 POP 330 79.020 91.550 51.160 1.00 0.00

ATOM 3269 C1A POP 330 79.770 92.620 54.680 1.00 0.00

ATOM 3270 C2A POP 330 78.340 95.540 57.510 1.00 0.00

ATOM 3271 C3A POP 330 79.160 96.070 61.740 1.00 0.00

ATOM 3272 C4A POP 330 79.320 97.370 66.810 1.00 0.00

ATOM 3273 C1B POP 330 77.730 87.320 53.860 1.00 0.00

ATOM 3274 C2B POP 330 76.180 87.880 58.150 1.00 0.00

ATOM 3275 D3B POP 330 72.900 86.730 63.050 1.00 0.00

ATOM 3276 C4B POP 330 74.940 82.940 63.920 1.00 0.00

ATOM 3277 C5B POP 330 76.220 79.730 63.680 1.00 0.00

ATOM 3278 NC3 POP 331 63.960 39.860 91.380 1.00 0.00

ATOM 3279 PO4 POP 331 61.560 38.380 88.750 1.00 0.00

ATOM 3280 GL1 POP 331 59.400 39.730 84.340 1.00 0.00

ATOM 3281 GL2 POP 331 58.410 42.700 84.780 1.00 0.00

ATOM 3282 C1A POP 331 62.130 42.810 81.860 1.00 0.00

ATOM 3283 C2A POP 331 63.690 45.370 78.650 1.00 0.00

ATOM 3284 C3A POP 331 66.230 45.230 75.460 1.00 0.00

ATOM 3285 C4A POP 331 67.540 43.840 71.710 1.00 0.00

ATOM 3286 C1B POP 331 57.510 41.850 80.330 1.00 0.00

ATOM 3287 C2B POP 331 55.730 44.410 76.790 1.00 0.00

ATOM 3288 D3B POP 331 53.840 48.940 74.820 1.00 0.00

ATOM 3289 C4B POP 331 53.140 50.130 70.790 1.00 0.00

ATOM 3290 C5B POP 331 51.290 50.620 66.500 1.00 0.00

ATOM 3291 NC3 POP 332 7.960 42.440 44.090 1.00 0.00

ATOM 3292 PO4 POP 332 7.580 45.240 46.940 1.00 0.00

ATOM 3293 GL1 POP 332 4.890 44.590 50.610 1.00 0.00

ATOM 3294 GL2 POP 332 6.210 46.510 52.950 1.00 0.00

ATOM 3295 C1A POP 332 6.890 43.800 57.180 1.00 0.00

ATOM 3296 C2A POP 332 7.280 40.470 59.820 1.00 0.00

ATOM 3297 C3A POP 332 6.970 38.300 64.340 1.00 0.00

ATOM 3298 C4A POP 332 5.420 40.890 68.280 1.00 0.00

ATOM 3299 C1B POP 332 1.140 43.440 52.340 1.00 0.00

ATOM 3300 C2B POP 332 2.510 42.370 56.370 1.00 0.00

ATOM 3301 D3B POP 332 2.480 38.760 61.000 1.00 0.00

ATOM 3302 C4B POP 332 2.270 39.190 65.350 1.00 0.00

ATOM 3303 C5B POP 332 -1.150 36.880 67.670 1.00 0.00

ATOM 3304 NC3 POP 333 97.350 35.270 42.860 1.00 0.00

ATOM 3305 PO4 POP 333 99.440 38.520 46.650 1.00 0.00

ATOM 3306 GL1 POP 333 100.870 38.840 51.310 1.00 0.00

ATOM 3307 GL2 POP 333 100.240 41.370 54.570 1.00 0.00

ATOM 3308 C1A POP 333 99.530 45.050 56.820 1.00 0.00

ATOM 3309 C2A POP 333 100.480 44.930 60.960 1.00 0.00

ATOM 3310 C3A POP 333 98.970 43.680 64.850 1.00 0.00

ATOM 3311 C4A POP 333 97.630 40.560 67.980 1.00 0.00

ATOM 3312 C1B POP 333 104.080 38.510 54.690 1.00 0.00

ATOM 3313 C2B POP 333 102.940 40.510 59.690 1.00 0.00

ATOM 3314 D3B POP 333 103.780 42.720 63.600 1.00 0.00

ATOM 3315 C4B POP 333 103.020 47.230 65.500 1.00 0.00

ATOM 3316 C5B POP 333 100.860 48.300 68.950 1.00 0.00

ATOM 3317 NC3 POP 334 32.380 73.560 43.760 1.00 0.00

ATOM 3318 PO4 POP 334 29.430 73.740 47.140 1.00 0.00

ATOM 3319 GL1 POP 334 26.480 72.430 50.590 1.00 0.00

ATOM 3320 GL2 POP 334 29.600 72.560 51.840 1.00 0.00

ATOM 3321 C1A POP 334 31.460 71.210 55.620 1.00 0.00

ATOM 3322 C2A POP 334 31.240 73.130 59.110 1.00 0.00

ATOM 3323 C3A POP 334 32.800 72.570 62.660 1.00 0.00

ATOM 3324 C4A POP 334 32.980 70.400 66.610 1.00 0.00

ATOM 3325 C1B POP 334 24.490 72.590 53.770 1.00 0.00

ATOM 3326 C2B POP 334 26.510 70.830 58.440 1.00 0.00

ATOM 3327 D3B POP 334 29.030 68.540 62.180 1.00 0.00

ATOM 3328 C4B POP 334 28.040 69.920 66.930 1.00 0.00

ATOM 3329 C5B POP 334 26.320 68.750 69.790 1.00 0.00

ATOM 3330 NC3 POP 335 107.530 44.970 88.050 1.00 0.00

ATOM 3331 PO4 POP 335 104.100 46.890 87.260 1.00 0.00

ATOM 3332 GL1 POP 335 103.380 48.890 83.300 1.00 0.00

ATOM 3333 GL2 POP 335 100.750 47.490 82.590 1.00 0.00

ATOM 3334 C1A POP 335 98.700 49.780 81.000 1.00 0.00

ATOM 3335 C2A POP 335 98.380 50.810 76.810 1.00 0.00

ATOM 3336 C3A POP 335 101.940 49.800 73.460 1.00 0.00

ATOM 3337 C4A POP 335 105.750 48.550 75.390 1.00 0.00

ATOM 3338 C1B POP 335 103.600 52.220 80.140 1.00 0.00

ATOM 3339 C2B POP 335 103.070 53.870 76.380 1.00 0.00

ATOM 3340 D3B POP 335 101.510 56.960 75.330 1.00 0.00

ATOM 3341 C4B POP 335 101.110 61.250 72.030 1.00 0.00

ATOM 3342 C5B POP 335 102.830 63.960 69.020 1.00 0.00

ATOM 3343 NC3 POP 336 73.180 53.420 88.770 1.00 0.00

ATOM 3344 PO4 POP 336 77.710 54.330 88.010 1.00 0.00

ATOM 3345 GL1 POP 336 77.920 54.650 83.730 1.00 0.00

ATOM 3346 GL2 POP 336 77.350 58.020 83.140 1.00 0.00

ATOM 3347 C1A POP 336 79.370 58.540 79.160 1.00 0.00

ATOM 3348 C2A POP 336 79.800 59.090 74.600 1.00 0.00

ATOM 3349 C3A POP 336 81.590 60.250 70.620 1.00 0.00

ATOM 3350 C4A POP 336 83.400 57.730 66.190 1.00 0.00

ATOM 3351 C1B POP 336 75.480 54.010 80.180 1.00 0.00

ATOM 3352 C2B POP 336 75.710 56.650 76.380 1.00 0.00

ATOM 3353 D3B POP 336 75.800 57.090 71.990 1.00 0.00

ATOM 3354 C4B POP 336 74.160 56.680 68.860 1.00 0.00

ATOM 3355 C5B POP 336 71.030 54.690 65.920 1.00 0.00

ATOM 3356 NC3 POP 337 23.980 94.250 91.010 1.00 0.00

ATOM 3357 PO4 POP 337 22.400 93.150 87.500 1.00 0.00

ATOM 3358 GL1 POP 337 22.470 91.990 82.570 1.00 0.00

ATOM 3359 GL2 POP 337 22.260 94.820 80.590 1.00 0.00

ATOM 3360 C1A POP 337 25.540 96.420 77.860 1.00 0.00

ATOM 3361 C2A POP 337 27.840 99.130 74.890 1.00 0.00

ATOM 3362 C3A POP 337 25.890 100.870 72.300 1.00 0.00

ATOM 3363 C4A POP 337 23.150 99.910 69.530 1.00 0.00

ATOM 3364 C1B POP 337 24.330 90.430 79.440 1.00 0.00

ATOM 3365 C2B POP 337 22.610 87.350 76.150 1.00 0.00

ATOM 3366 D3B POP 337 20.410 84.800 72.730 1.00 0.00

ATOM 3367 C4B POP 337 20.500 84.690 68.030 1.00 0.00

ATOM 3368 C5B POP 337 23.310 82.460 64.820 1.00 0.00

ATOM 3369 NC3 POP 338 51.980 77.110 88.310 1.00 0.00

ATOM 3370 PO4 POP 338 55.980 76.020 86.480 1.00 0.00

ATOM 3371 GL1 POP 338 56.830 75.440 82.110 1.00 0.00

ATOM 3372 GL2 POP 338 54.350 73.660 81.180 1.00 0.00

ATOM 3373 C1A POP 338 52.460 76.110 77.320 1.00 0.00

ATOM 3374 C2A POP 338 51.550 79.090 74.810 1.00 0.00

ATOM 3375 C3A POP 338 48.830 79.070 72.060 1.00 0.00

ATOM 3376 C4A POP 338 45.170 79.180 68.730 1.00 0.00

ATOM 3377 C1B POP 338 57.270 73.950 77.490 1.00 0.00

ATOM 3378 C2B POP 338 56.140 72.970 73.430 1.00 0.00

ATOM 3379 D3B POP 338 54.410 69.640 70.510 1.00 0.00

ATOM 3380 C4B POP 338 57.910 66.020 69.130 1.00 0.00

ATOM 3381 C5B POP 338 59.810 62.550 69.910 1.00 0.00

ATOM 3382 NC3 POP 339 38.440 81.460 90.660 1.00 0.00

ATOM 3383 PO4 POP 339 39.210 85.230 89.100 1.00 0.00

ATOM 3384 GL1 POP 339 37.160 84.940 84.990 1.00 0.00

ATOM 3385 GL2 POP 339 33.390 83.680 84.340 1.00 0.00

ATOM 3386 C1A POP 339 31.060 80.590 81.530 1.00 0.00

ATOM 3387 C2A POP 339 29.440 77.140 79.430 1.00 0.00

ATOM 3388 C3A POP 339 30.250 76.970 75.250 1.00 0.00

ATOM 3389 C4A POP 339 28.040 77.140 70.970 1.00 0.00

ATOM 3390 C1B POP 339 36.990 82.760 81.440 1.00 0.00

ATOM 3391 C2B POP 339 34.020 80.330 77.300 1.00 0.00

ATOM 3392 D3B POP 339 32.610 81.420 73.190 1.00 0.00

ATOM 3393 C4B POP 339 29.260 83.400 71.590 1.00 0.00

ATOM 3394 C5B POP 339 25.600 85.380 69.280 1.00 0.00

ATOM 3395 NC3 POP 340 65.520 82.750 87.250 1.00 0.00

ATOM 3396 PO4 POP 340 61.270 82.840 84.850 1.00 0.00

ATOM 3397 GL1 POP 340 61.590 81.070 80.650 1.00 0.00

ATOM 3398 GL2 POP 340 61.200 77.350 80.060 1.00 0.00

ATOM 3399 C1A POP 340 63.130 76.250 75.920 1.00 0.00

ATOM 3400 C2A POP 340 60.710 75.640 72.410 1.00 0.00

ATOM 3401 C3A POP 340 59.050 74.040 69.080 1.00 0.00

ATOM 3402 C4A POP 340 58.130 74.490 64.510 1.00 0.00

ATOM 3403 C1B POP 340 63.820 81.490 76.930 1.00 0.00

ATOM 3404 C2B POP 340 62.390 81.240 72.530 1.00 0.00

ATOM 3405 D3B POP 340 61.670 83.950 69.160 1.00 0.00

ATOM 3406 C4B POP 340 57.920 86.290 67.640 1.00 0.00

ATOM 3407 C5B POP 340 56.570 90.670 67.980 1.00 0.00

ATOM 3408 NC3 POP 341 65.840 90.090 42.520 1.00 0.00

ATOM 3409 PO4 POP 341 67.640 90.670 46.300 1.00 0.00

ATOM 3410 GL1 POP 341 71.490 89.200 47.030 1.00 0.00

ATOM 3411 GL2 POP 341 72.520 91.160 49.600 1.00 0.00

ATOM 3412 C1A POP 341 74.480 91.400 53.290 1.00 0.00

ATOM 3413 C2A POP 341 73.480 93.010 57.360 1.00 0.00

ATOM 3414 C3A POP 341 75.310 91.260 61.960 1.00 0.00

ATOM 3415 C4A POP 341 77.860 91.880 65.220 1.00 0.00

ATOM 3416 C1B POP 341 72.800 85.910 50.570 1.00 0.00

ATOM 3417 C2B POP 341 73.560 84.470 54.420 1.00 0.00

ATOM 3418 D3B POP 341 71.720 83.150 58.810 1.00 0.00

ATOM 3419 C4B POP 341 67.060 83.970 60.740 1.00 0.00

ATOM 3420 C5B POP 341 64.080 86.460 59.910 1.00 0.00

ATOM 3421 NC3 POP 342 7.440 79.440 90.250 1.00 0.00

ATOM 3422 PO4 POP 342 10.600 76.720 89.330 1.00 0.00

ATOM 3423 GL1 POP 342 10.840 73.600 85.940 1.00 0.00

ATOM 3424 GL2 POP 342 8.330 71.640 85.730 1.00 0.00

ATOM 3425 C1A POP 342 8.100 67.980 83.910 1.00 0.00

ATOM 3426 C2A POP 342 9.020 64.650 81.830 1.00 0.00

ATOM 3427 C3A POP 342 8.560 63.510 77.150 1.00 0.00

ATOM 3428 C4A POP 342 6.390 63.790 73.290 1.00 0.00

ATOM 3429 C1B POP 342 11.760 71.850 81.160 1.00 0.00

ATOM 3430 C2B POP 342 10.080 73.210 77.000 1.00 0.00

ATOM 3431 D3B POP 342 10.010 73.540 72.200 1.00 0.00

ATOM 3432 C4B POP 342 11.300 69.540 69.980 1.00 0.00

ATOM 3433 C5B POP 342 9.410 67.360 66.350 1.00 0.00

ATOM 3434 NC3 POP 343 55.910 5.460 46.930 1.00 0.00

ATOM 3435 PO4 POP 343 59.720 5.300 44.950 1.00 0.00

ATOM 3436 GL1 POP 343 60.370 3.780 49.340 1.00 0.00

ATOM 3437 GL2 POP 343 62.790 1.980 48.470 1.00 0.00

ATOM 3438 C1A POP 343 59.610 -0.090 52.880 1.00 0.00

ATOM 3439 C2A POP 343 56.260 -1.990 55.800 1.00 0.00

ATOM 3440 C3A POP 343 54.930 -1.630 59.880 1.00 0.00

ATOM 3441 C4A POP 343 51.740 -0.360 62.380 1.00 0.00

ATOM 3442 C1B POP 343 59.990 5.240 52.580 1.00 0.00

ATOM 3443 C2B POP 343 57.530 4.090 55.300 1.00 0.00

ATOM 3444 D3B POP 343 56.320 3.240 59.290 1.00 0.00

ATOM 3445 C4B POP 343 58.410 6.520 62.570 1.00 0.00

ATOM 3446 C5B POP 343 55.570 8.890 65.780 1.00 0.00

ATOM 3447 NC3 POP 344 58.820 99.090 45.790 1.00 0.00

ATOM 3448 PO4 POP 344 59.900 104.370 44.640 1.00 0.00

ATOM 3449 GL1 POP 344 56.560 103.380 48.140 1.00 0.00

ATOM 3450 GL2 POP 344 57.790 104.290 50.660 1.00 0.00

ATOM 3451 C1A POP 344 59.750 103.720 54.280 1.00 0.00

ATOM 3452 C2A POP 344 59.750 102.660 58.110 1.00 0.00

ATOM 3453 C3A POP 344 58.980 105.080 61.570 1.00 0.00

ATOM 3454 C4A POP 344 60.920 104.470 66.200 1.00 0.00

ATOM 3455 C1B POP 344 55.540 100.950 53.300 1.00 0.00

ATOM 3456 C2B POP 344 54.660 101.240 56.850 1.00 0.00

ATOM 3457 D3B POP 344 55.010 101.420 61.370 1.00 0.00

ATOM 3458 C4B POP 344 59.430 100.320 63.650 1.00 0.00

ATOM 3459 C5B POP 344 60.640 99.650 68.270 1.00 0.00

ATOM 3460 NC3 POP 345 58.010 8.030 84.940 1.00 0.00

ATOM 3461 PO4 POP 345 55.750 3.880 83.380 1.00 0.00

ATOM 3462 GL1 POP 345 56.450 6.330 80.400 1.00 0.00

ATOM 3463 GL2 POP 345 57.560 4.000 78.290 1.00 0.00

ATOM 3464 C1A POP 345 61.890 7.750 76.710 1.00 0.00

ATOM 3465 C2A POP 345 65.410 7.680 72.960 1.00 0.00

ATOM 3466 C3A POP 345 69.820 6.470 70.540 1.00 0.00

ATOM 3467 C4A POP 345 69.900 2.860 71.560 1.00 0.00

ATOM 3468 C1B POP 345 55.810 8.430 76.490 1.00 0.00

ATOM 3469 C2B POP 345 54.280 10.300 71.390 1.00 0.00

ATOM 3470 D3B POP 345 56.130 13.570 68.140 1.00 0.00

ATOM 3471 C4B POP 345 54.370 13.530 64.850 1.00 0.00

ATOM 3472 C5B POP 345 55.610 11.810 60.580 1.00 0.00

ATOM 3473 NC3 POP 346 93.190 83.320 45.880 1.00 0.00

ATOM 3474 PO4 POP 346 96.240 80.890 48.280 1.00 0.00

ATOM 3475 GL1 POP 346 97.010 82.460 52.710 1.00 0.00

ATOM 3476 GL2 POP 346 98.010 85.440 54.400 1.00 0.00

ATOM 3477 C1A POP 346 100.920 86.030 57.480 1.00 0.00

ATOM 3478 C2A POP 346 101.460 86.940 62.180 1.00 0.00

ATOM 3479 C3A POP 346 98.170 88.090 64.260 1.00 0.00

ATOM 3480 C4A POP 346 96.940 91.340 68.140 1.00 0.00

ATOM 3481 C1B POP 346 98.090 80.140 56.790 1.00 0.00

ATOM 3482 C2B POP 346 97.120 78.580 60.460 1.00 0.00

ATOM 3483 D3B POP 346 98.840 76.870 64.350 1.00 0.00

ATOM 3484 C4B POP 346 99.230 79.190 67.370 1.00 0.00

ATOM 3485 C5B POP 346 95.660 81.760 69.840 1.00 0.00

ATOM 3486 NC3 POP 347 85.910 88.200 44.200 1.00 0.00

ATOM 3487 PO4 POP 347 90.010 87.700 46.470 1.00 0.00

ATOM 3488 GL1 POP 347 89.820 88.520 50.550 1.00 0.00

ATOM 3489 GL2 POP 347 85.880 87.320 50.840 1.00 0.00

ATOM 3490 C1A POP 347 83.810 87.490 55.310 1.00 0.00

ATOM 3491 C2A POP 347 81.190 86.840 57.590 1.00 0.00

ATOM 3492 C3A POP 347 79.700 89.220 61.010 1.00 0.00

ATOM 3493 C4A POP 347 81.920 92.630 63.010 1.00 0.00

ATOM 3494 C1B POP 347 89.020 88.790 54.510 1.00 0.00

ATOM 3495 C2B POP 347 88.950 87.230 59.020 1.00 0.00

ATOM 3496 D3B POP 347 88.280 82.920 60.210 1.00 0.00

ATOM 3497 C4B POP 347 90.820 80.160 64.060 1.00 0.00

ATOM 3498 C5B POP 347 94.460 79.620 64.980 1.00 0.00

ATOM 3499 NC3 POP 348 11.620 60.420 44.880 1.00 0.00

ATOM 3500 PO4 POP 348 7.450 60.490 46.880 1.00 0.00

ATOM 3501 GL1 POP 348 6.440 59.590 51.560 1.00 0.00

ATOM 3502 GL2 POP 348 6.220 61.510 54.640 1.00 0.00

ATOM 3503 C1A POP 348 8.120 60.160 57.980 1.00 0.00

ATOM 3504 C2A POP 348 12.830 58.140 59.110 1.00 0.00

ATOM 3505 C3A POP 348 13.080 54.470 62.690 1.00 0.00

ATOM 3506 C4A POP 348 15.650 52.190 66.430 1.00 0.00

ATOM 3507 C1B POP 348 5.710 56.760 55.850 1.00 0.00

ATOM 3508 C2B POP 348 7.800 55.760 60.810 1.00 0.00

ATOM 3509 D3B POP 348 9.190 57.820 64.620 1.00 0.00

ATOM 3510 C4B POP 348 7.020 62.200 65.610 1.00 0.00

ATOM 3511 C5B POP 348 4.890 64.920 68.380 1.00 0.00

ATOM 3512 NC3 POP 349 82.630 104.810 37.440 1.00 0.00

ATOM 3513 PO4 POP 349 81.480 106.310 41.760 1.00 0.00

ATOM 3514 GL1 POP 349 80.430 107.860 45.740 1.00 0.00

ATOM 3515 GL2 POP 349 82.570 109.580 45.250 1.00 0.00

ATOM 3516 C1A POP 349 84.660 108.760 48.920 1.00 0.00

ATOM 3517 C2A POP 349 85.430 106.800 52.760 1.00 0.00

ATOM 3518 C3A POP 349 83.860 106.720 56.870 1.00 0.00

ATOM 3519 C4A POP 349 81.280 106.210 61.260 1.00 0.00

ATOM 3520 C1B POP 349 78.000 108.810 48.270 1.00 0.00

ATOM 3521 C2B POP 349 73.520 107.890 50.820 1.00 0.00

ATOM 3522 D3B POP 349 70.990 109.130 55.260 1.00 0.00

ATOM 3523 C4B POP 349 71.890 106.600 58.620 1.00 0.00

ATOM 3524 C5B POP 349 72.010 105.320 63.420 1.00 0.00

ATOM 3525 NC3 POP 350 64.320 103.400 41.800 1.00 0.00

ATOM 3526 PO4 POP 350 67.310 99.860 42.240 1.00 0.00

ATOM 3527 GL1 POP 350 67.310 100.340 46.220 1.00 0.00

ATOM 3528 GL2 POP 350 65.500 102.420 47.670 1.00 0.00

ATOM 3529 C1A POP 350 63.360 101.130 51.270 1.00 0.00

ATOM 3530 C2A POP 350 64.630 102.140 55.370 1.00 0.00

ATOM 3531 C3A POP 350 63.430 98.190 58.560 1.00 0.00

ATOM 3532 C4A POP 350 60.970 93.190 57.720 1.00 0.00

ATOM 3533 C1B POP 350 68.120 98.690 50.090 1.00 0.00

ATOM 3534 C2B POP 350 66.460 96.400 54.280 1.00 0.00

ATOM 3535 D3B POP 350 68.770 97.050 58.420 1.00 0.00

ATOM 3536 C4B POP 350 67.380 98.220 61.780 1.00 0.00

ATOM 3537 C5B POP 350 70.170 100.610 63.630 1.00 0.00

ATOM 3538 NC3 POP 351 79.450 62.300 46.760 1.00 0.00

ATOM 3539 PO4 POP 351 79.320 58.390 48.520 1.00 0.00

ATOM 3540 GL1 POP 351 78.150 59.410 52.540 1.00 0.00

ATOM 3541 GL2 POP 351 75.800 62.500 51.360 1.00 0.00

ATOM 3542 C1A POP 351 72.920 60.810 54.090 1.00 0.00

ATOM 3543 C2A POP 351 74.220 59.940 59.560 1.00 0.00

ATOM 3544 C3A POP 351 77.290 58.200 58.920 1.00 0.00

ATOM 3545 C4A POP 351 79.820 55.420 58.040 1.00 0.00

ATOM 3546 C1B POP 351 78.060 62.190 56.750 1.00 0.00

ATOM 3547 C2B POP 351 80.240 64.920 58.510 1.00 0.00

ATOM 3548 D3B POP 351 82.920 66.770 61.520 1.00 0.00

ATOM 3549 C4B POP 351 81.450 71.170 65.000 1.00 0.00

ATOM 3550 C5B POP 351 78.530 74.940 64.780 1.00 0.00

ATOM 3551 NC3 POP 352 36.460 27.830 88.550 1.00 0.00

ATOM 3552 PO4 POP 352 38.260 26.800 84.850 1.00 0.00

ATOM 3553 GL1 POP 352 39.680 26.050 80.440 1.00 0.00

ATOM 3554 GL2 POP 352 37.160 24.140 79.720 1.00 0.00

ATOM 3555 C1A POP 352 38.930 21.840 76.720 1.00 0.00

ATOM 3556 C2A POP 352 39.030 19.350 73.380 1.00 0.00

ATOM 3557 C3A POP 352 38.140 16.780 70.850 1.00 0.00

ATOM 3558 C4A POP 352 35.380 12.640 70.270 1.00 0.00

ATOM 3559 C1B POP 352 40.530 26.410 75.520 1.00 0.00

ATOM 3560 C2B POP 352 38.880 24.810 72.200 1.00 0.00

ATOM 3561 D3B POP 352 36.990 26.890 70.720 1.00 0.00

ATOM 3562 C4B POP 352 36.340 31.100 68.440 1.00 0.00

ATOM 3563 C5B POP 352 38.980 34.320 68.410 1.00 0.00

ATOM 3564 NC3 POP 353 3.070 5.650 93.270 1.00 0.00

ATOM 3565 PO4 POP 353 4.270 4.240 88.720 1.00 0.00

ATOM 3566 GL1 POP 353 2.610 4.050 83.870 1.00 0.00

ATOM 3567 GL2 POP 353 3.040 6.620 81.280 1.00 0.00

ATOM 3568 C1A POP 353 4.570 5.290 77.080 1.00 0.00

ATOM 3569 C2A POP 353 4.500 3.640 73.350 1.00 0.00

ATOM 3570 C3A POP 353 3.420 5.300 69.400 1.00 0.00

ATOM 3571 C4A POP 353 3.470 5.130 65.040 1.00 0.00

ATOM 3572 C1B POP 353 1.190 2.210 80.040 1.00 0.00

ATOM 3573 C2B POP 353 1.340 0.370 76.270 1.00 0.00

ATOM 3574 D3B POP 353 2.860 -1.450 72.030 1.00 0.00

ATOM 3575 C4B POP 353 0.070 0.730 68.440 1.00 0.00

ATOM 3576 C5B POP 353 1.840 0.250 64.470 1.00 0.00

ATOM 3577 NC3 POP 354 78.850 80.030 42.940 1.00 0.00

ATOM 3578 PO4 POP 354 77.100 78.750 47.150 1.00 0.00

ATOM 3579 GL1 POP 354 78.660 76.820 51.650 1.00 0.00

ATOM 3580 GL2 POP 354 79.560 73.160 50.540 1.00 0.00

ATOM 3581 C1A POP 354 79.380 70.970 55.250 1.00 0.00

ATOM 3582 C2A POP 354 78.710 69.290 59.570 1.00 0.00

ATOM 3583 C3A POP 354 76.870 70.060 64.130 1.00 0.00

ATOM 3584 C4A POP 354 75.170 71.900 66.640 1.00 0.00

ATOM 3585 C1B POP 354 78.570 76.760 55.240 1.00 0.00

ATOM 3586 C2B POP 354 80.020 74.490 59.540 1.00 0.00

ATOM 3587 D3B POP 354 82.930 75.560 62.840 1.00 0.00

ATOM 3588 C4B POP 354 85.880 73.660 65.110 1.00 0.00

ATOM 3589 C5B POP 354 88.820 74.200 67.540 1.00 0.00

ATOM 3590 NC3 POP 355 101.910 65.160 88.670 1.00 0.00

ATOM 3591 PO4 POP 355 98.060 66.700 90.190 1.00 0.00

ATOM 3592 GL1 POP 355 97.930 66.620 85.440 1.00 0.00

ATOM 3593 GL2 POP 355 95.100 64.080 84.350 1.00 0.00

ATOM 3594 C1A POP 355 92.190 65.830 81.030 1.00 0.00

ATOM 3595 C2A POP 355 93.850 68.730 77.700 1.00 0.00

ATOM 3596 C3A POP 355 94.860 68.910 72.350 1.00 0.00

ATOM 3597 C4A POP 355 93.840 72.370 70.190 1.00 0.00

ATOM 3598 C1B POP 355 97.200 66.300 80.650 1.00 0.00

ATOM 3599 C2B POP 355 98.630 67.790 77.060 1.00 0.00

ATOM 3600 D3B POP 355 98.330 65.380 73.870 1.00 0.00

ATOM 3601 C4B POP 355 98.450 67.400 69.320 1.00 0.00

ATOM 3602 C5B POP 355 102.360 68.390 66.980 1.00 0.00

ATOM 3603 NCO POP 356 52.550 14.870 45.750 1.00 0.00

ATOM 3604 PO4 POP 356 49.200 13.220 44.940 1.00 0.00

ATOM 3605 GL1 POP 356 50.630 11.650 49.200 1.00 0.00

ATOM 3606 GL2 POP 356 50.120 7.590 49.140 1.00 0.00

ATOM 3607 C1A POP 356 48.360 7.020 53.190 1.00 0.00

ATOM 3608 C2A POP 356 47.720 7.730 57.170 1.00 0.00

ATOM 3609 C3A POP 356 46.340 8.060 61.160 1.00 0.00

ATOM 3610 C4A POP 356 44.210 9.500 64.730 1.00 0.00

ATOM 3611 C1B POP 356 48.870 11.550 53.400 1.00 0.00

ATOM 3612 C2B POP 356 46.980 13.530 56.690 1.00 0.00

ATOM 3613 D3B POP 356 44.990 15.610 60.570 1.00 0.00

ATOM 3614 C4B POP 356 42.420 18.950 62.350 1.00 0.00

ATOM 3615 C5B POP 356 39.750 23.030 62.660 1.00 0.00

ATOM 3616 NCO POP 357 81.950 67.920 43.210 1.00 0.00

ATOM 3617 PO4 POP 357 78.590 67.050 44.370 1.00 0.00

ATOM 3618 GL1 POP 357 77.220 68.410 48.440 1.00 0.00

ATOM 3619 GL2 POP 357 74.700 72.880 48.720 1.00 0.00

ATOM 3620 C1A POP 357 73.980 70.830 52.910 1.00 0.00

ATOM 3621 C2A POP 357 75.660 72.820 57.490 1.00 0.00

ATOM 3622 C3A POP 357 75.140 73.900 61.450 1.00 0.00

ATOM 3623 C4A POP 357 72.490 75.840 63.820 1.00 0.00

ATOM 3624 C1B POP 357 77.320 67.090 52.200 1.00 0.00

ATOM 3625 C2B POP 357 75.770 66.590 56.670 1.00 0.00

ATOM 3626 D3B POP 357 75.950 64.550 60.820 1.00 0.00

ATOM 3627 C4B POP 357 75.030 61.660 64.380 1.00 0.00

ATOM 3628 C5B POP 357 70.740 59.590 66.260 1.00 0.00

ATOM 3629 NCO POP 358 87.200 65.560 43.090 1.00 0.00

ATOM 3630 PO4 POP 358 89.840 67.720 46.370 1.00 0.00

ATOM 3631 GL1 POP 358 87.370 69.040 50.280 1.00 0.00

ATOM 3632 GL2 POP 358 85.060 72.800 49.390 1.00 0.00

ATOM 3633 C1A POP 358 84.740 72.040 53.710 1.00 0.00

ATOM 3634 C2A POP 358 88.040 73.390 56.000 1.00 0.00

ATOM 3635 C3A POP 358 88.870 71.460 59.770 1.00 0.00

ATOM 3636 C4A POP 358 90.120 71.360 63.110 1.00 0.00

ATOM 3637 C1B POP 358 89.370 69.130 54.270 1.00 0.00

ATOM 3638 C2B POP 358 89.520 66.830 57.860 1.00 0.00

ATOM 3639 D3B POP 358 90.600 66.730 61.540 1.00 0.00

ATOM 3640 C4B POP 358 87.040 67.250 64.130 1.00 0.00

ATOM 3641 C5B POP 358 83.600 66.090 66.510 1.00 0.00

ATOM 3642 NCO POP 359 49.860 84.210 43.300 1.00 0.00

ATOM 3643 PO4 POP 359 53.030 86.310 45.440 1.00 0.00

ATOM 3644 GL1 POP 359 55.730 85.340 49.260 1.00 0.00

ATOM 3645 GL2 POP 359 55.240 81.890 50.210 1.00 0.00

ATOM 3646 C1A POP 359 55.220 81.450 54.100 1.00 0.00

ATOM 3647 C2A POP 359 53.880 80.180 59.300 1.00 0.00

ATOM 3648 C3A POP 359 53.980 75.450 61.200 1.00 0.00

ATOM 3649 C4A POP 359 54.500 71.220 62.710 1.00 0.00

ATOM 3650 C1B POP 359 59.490 86.020 51.970 1.00 0.00

ATOM 3651 C2B POP 359 59.650 86.720 56.590 1.00 0.00

ATOM 3652 D3B POP 359 59.570 89.180 60.240 1.00 0.00

ATOM 3653 C4B POP 359 58.460 91.990 63.140 1.00 0.00

ATOM 3654 C5B POP 359 59.190 95.040 66.540 1.00 0.00

ATOM 3655 NCO POP 360 41.110 91.720 47.820 1.00 0.00

ATOM 3656 PO4 POP 360 38.860 95.020 46.250 1.00 0.00

ATOM 3657 GL1 POP 360 37.730 94.560 51.260 1.00 0.00

ATOM 3658 GL2 POP 360 34.430 93.420 48.860 1.00 0.00

ATOM 3659 C1A POP 360 32.370 96.700 51.320 1.00 0.00

ATOM 3660 C2A POP 360 29.390 98.380 54.100 1.00 0.00

ATOM 3661 C3A POP 360 25.270 101.690 55.320 1.00 0.00

ATOM 3662 C4A POP 360 23.380 105.550 53.060 1.00 0.00

ATOM 3663 C1B POP 360 35.090 93.010 56.010 1.00 0.00

ATOM 3664 C2B POP 360 36.280 94.220 59.500 1.00 0.00

ATOM 3665 D3B POP 360 36.860 97.150 62.350 1.00 0.00

ATOM 3666 C4B POP 360 38.840 97.980 65.840 1.00 0.00

ATOM 3667 C5B POP 360 36.480 94.370 66.860 1.00 0.00

ATOM 3668 NCO POP 361 -3.470 71.480 44.420 1.00 0.00

ATOM 3669 PO4 POP 361 -0.500 67.980 46.980 1.00 0.00

ATOM 3670 GL1 POP 361 1.000 71.700 51.370 1.00 0.00

ATOM 3671 GL2 POP 361 0.030 71.520 56.310 1.00 0.00

ATOM 3672 C1A POP 361 -0.660 71.750 60.030 1.00 0.00

ATOM 3673 C2A POP 361 2.250 73.070 62.310 1.00 0.00

ATOM 3674 C3A POP 361 4.810 74.590 65.930 1.00 0.00

ATOM 3675 C4A POP 361 5.490 73.090 69.410 1.00 0.00

ATOM 3676 C1B POP 361 3.300 74.670 54.880 1.00 0.00

ATOM 3677 C2B POP 361 3.240 76.930 58.660 1.00 0.00

ATOM 3678 D3B POP 361 5.570 76.230 61.470 1.00 0.00

ATOM 3679 C4B POP 361 9.080 77.790 64.430 1.00 0.00

ATOM 3680 C5B POP 361 11.640 80.840 61.340 1.00 0.00

ATOM 3681 NCO POP 362 107.320 88.780 91.220 1.00 0.00

ATOM 3682 PO4 POP 362 102.190 89.720 90.530 1.00 0.00

ATOM 3683 GL1 POP 362 102.080 88.650 87.130 1.00 0.00

ATOM 3684 GL2 POP 362 99.930 91.840 85.170 1.00 0.00

ATOM 3685 C1A POP 362 100.860 88.500 81.590 1.00 0.00

ATOM 3686 C2A POP 362 103.330 89.120 78.790 1.00 0.00

ATOM 3687 C3A POP 362 107.310 88.060 77.510 1.00 0.00

ATOM 3688 C4A POP 362 110.860 85.770 77.150 1.00 0.00

ATOM 3689 C1B POP 362 105.340 88.420 84.340 1.00 0.00

ATOM 3690 C2B POP 362 108.650 85.640 82.150 1.00 0.00

ATOM 3691 D3B POP 362 109.000 81.720 78.980 1.00 0.00

ATOM 3692 C4B POP 362 105.800 79.160 80.230 1.00 0.00

ATOM 3693 C5B POP 362 108.730 80.590 84.120 1.00 0.00

ATOM 3694 NCO POP 363 23.320 87.800 46.370 1.00 0.00

ATOM 3695 PO4 POP 363 19.240 87.730 48.380 1.00 0.00

ATOM 3696 GL1 POP 363 20.590 88.130 51.910 1.00 0.00

ATOM 3697 GL2 POP 363 24.560 90.130 50.770 1.00 0.00

ATOM 3698 C1A POP 363 24.560 91.000 55.810 1.00 0.00

ATOM 3699 C2A POP 363 26.540 90.360 59.550 1.00 0.00

ATOM 3700 C3A POP 363 27.770 90.680 64.270 1.00 0.00

ATOM 3701 C4A POP 363 29.120 88.110 66.710 1.00 0.00

ATOM 3702 C1B POP 363 21.820 86.920 56.580 1.00 0.00

ATOM 3703 C2B POP 363 21.420 85.500 60.840 1.00 0.00

ATOM 3704 D3B POP 363 22.920 88.110 65.330 1.00 0.00

ATOM 3705 C4B POP 363 24.680 90.560 68.190 1.00 0.00

ATOM 3706 C5B POP 363 29.060 92.370 68.980 1.00 0.00

ATOM 3707 NCO POP 364 6.980 15.750 90.050 1.00 0.00

ATOM 3708 PO4 POP 364 4.590 19.110 88.480 1.00 0.00

ATOM 3709 GL1 POP 364 4.920 19.890 84.250 1.00 0.00

ATOM 3710 GL2 POP 364 1.900 16.130 84.040 1.00 0.00

ATOM 3711 C1A POP 364 0.080 16.070 80.490 1.00 0.00

ATOM 3712 C2A POP 364 2.050 16.620 76.610 1.00 0.00

ATOM 3713 C3A POP 364 3.080 15.140 71.710 1.00 0.00

ATOM 3714 C4A POP 364 3.960 14.610 67.820 1.00 0.00

ATOM 3715 C1B POP 364 1.140 21.020 81.240 1.00 0.00

ATOM 3716 C2B POP 364 -1.890 20.060 77.140 1.00 0.00

ATOM 3717 D3B POP 364 -0.190 20.820 72.920 1.00 0.00

ATOM 3718 C4B POP 364 0.470 21.180 69.430 1.00 0.00

ATOM 3719 C5B POP 364 -0.120 21.340 65.160 1.00 0.00

ATOM 3720 NCO POP 365 64.240 71.540 48.010 1.00 0.00

ATOM 3721 PO4 POP 365 65.310 75.930 44.870 1.00 0.00

ATOM 3722 GL1 POP 365 65.890 76.350 48.040 1.00 0.00

ATOM 3723 GL2 POP 365 68.850 76.930 51.090 1.00 0.00

ATOM 3724 C1A POP 365 69.120 78.790 54.790 1.00 0.00

ATOM 3725 C2A POP 365 66.470 80.800 57.030 1.00 0.00

ATOM 3726 C3A POP 365 63.210 80.690 59.930 1.00 0.00

ATOM 3727 C4A POP 365 62.180 79.450 64.190 1.00 0.00

ATOM 3728 C1B POP 365 64.000 76.980 52.220 1.00 0.00

ATOM 3729 C2B POP 365 61.710 78.580 56.160 1.00 0.00

ATOM 3730 D3B POP 365 58.870 81.560 57.100 1.00 0.00

ATOM 3731 C4B POP 365 58.720 84.060 61.070 1.00 0.00

ATOM 3732 C5B POP 365 58.350 82.190 64.960 1.00 0.00

ATOM 3733 NCO POP 366 16.230 61.760 49.240 1.00 0.00

ATOM 3734 PO4 POP 366 15.150 57.990 48.230 1.00 0.00

ATOM 3735 GL1 POP 366 16.240 56.220 52.210 1.00 0.00

ATOM 3736 GL2 POP 366 20.370 54.460 52.700 1.00 0.00

ATOM 3737 C1A POP 366 18.400 50.970 54.840 1.00 0.00

ATOM 3738 C2A POP 366 16.010 50.250 57.080 1.00 0.00

ATOM 3739 C3A POP 366 13.140 49.890 60.470 1.00 0.00

ATOM 3740 C4A POP 366 11.460 48.590 64.470 1.00 0.00

ATOM 3741 C1B POP 366 16.040 55.530 56.050 1.00 0.00

ATOM 3742 C2B POP 366 17.490 53.590 59.750 1.00 0.00

ATOM 3743 D3B POP 366 17.690 49.780 62.050 1.00 0.00

ATOM 3744 C4B POP 366 15.770 46.010 63.730 1.00 0.00

ATOM 3745 C5B POP 366 16.830 43.440 65.880 1.00 0.00

ATOM 3746 NCO POP 367 91.290 29.150 41.960 1.00 0.00

ATOM 3747 PO4 POP 367 92.190 26.090 43.360 1.00 0.00

ATOM 3748 GL1 POP 367 92.010 24.290 48.050 1.00 0.00

ATOM 3749 GL2 POP 367 95.420 23.690 50.210 1.00 0.00

ATOM 3750 C1A POP 367 93.700 23.780 53.900 1.00 0.00

ATOM 3751 C2A POP 367 91.500 21.780 56.550 1.00 0.00

ATOM 3752 C3A POP 367 91.460 21.030 61.010 1.00 0.00

ATOM 3753 C4A POP 367 90.600 17.340 62.980 1.00 0.00

ATOM 3754 C1B POP 367 89.220 25.830 51.560 1.00 0.00

ATOM 3755 C2B POP 367 90.490 27.280 55.600 1.00 0.00

ATOM 3756 D3B POP 367 92.750 25.940 59.020 1.00 0.00

ATOM 3757 C4B POP 367 94.950 25.660 62.670 1.00 0.00

ATOM 3758 C5B POP 367 91.060 25.840 65.110 1.00 0.00

ATOM 3759 NCO POP 368 99.710 103.360 40.740 1.00 0.00

ATOM 3760 PO4 POP 368 97.670 102.070 44.670 1.00 0.00

ATOM 3761 GL1 POP 368 96.590 104.430 47.500 1.00 0.00

ATOM 3762 GL2 POP 368 98.920 108.540 49.730 1.00 0.00

ATOM 3763 C1A POP 368 96.720 109.610 53.140 1.00 0.00

ATOM 3764 C2A POP 368 96.370 109.730 58.240 1.00 0.00

ATOM 3765 C3A POP 368 95.380 111.930 61.600 1.00 0.00

ATOM 3766 C4A POP 368 96.480 114.810 64.620 1.00 0.00

ATOM 3767 C1B POP 368 94.980 103.650 52.650 1.00 0.00

ATOM 3768 C2B POP 368 95.230 105.100 56.630 1.00 0.00

ATOM 3769 D3B POP 368 92.480 106.600 60.320 1.00 0.00

ATOM 3770 C4B POP 368 91.710 103.790 64.520 1.00 0.00

ATOM 3771 C5B POP 368 90.620 104.550 68.910 1.00 0.00

ATOM 3772 NCO POP 369 3.830 94.830 88.340 1.00 0.00

ATOM 3773 PO4 POP 369 1.790 91.150 89.840 1.00 0.00

ATOM 3774 GL1 POP 369 1.570 91.210 85.090 1.00 0.00

ATOM 3775 GL2 POP 369 6.250 91.720 84.550 1.00 0.00

ATOM 3776 C1A POP 369 6.290 91.610 79.070 1.00 0.00

ATOM 3777 C2A POP 369 7.530 90.260 73.720 1.00 0.00

ATOM 3778 C3A POP 369 7.840 91.030 69.570 1.00 0.00

ATOM 3779 C4A POP 369 10.820 91.990 67.160 1.00 0.00

ATOM 3780 C1B POP 369 1.230 90.140 80.840 1.00 0.00

ATOM 3781 C2B POP 369 2.910 90.210 75.960 1.00 0.00

ATOM 3782 D3B POP 369 3.170 86.720 72.210 1.00 0.00

ATOM 3783 C4B POP 369 4.810 84.870 68.840 1.00 0.00

ATOM 3784 C5B POP 369 7.590 84.000 67.000 1.00 0.00

ATOM 3785 NCO POP 370 10.630 3.340 37.980 1.00 0.00

ATOM 3786 PO4 POP 370 11.600 3.100 42.180 1.00 0.00

ATOM 3787 GL1 POP 370 9.830 6.550 45.310 1.00 0.00

ATOM 3788 GL2 POP 370 5.630 5.180 46.220 1.00 0.00

ATOM 3789 C1A POP 370 3.960 4.060 50.360 1.00 0.00

ATOM 3790 C2A POP 370 2.670 4.160 54.600 1.00 0.00

ATOM 3791 C3A POP 370 -1.240 4.130 54.990 1.00 0.00

ATOM 3792 C4A POP 370 -5.100 4.340 56.860 1.00 0.00

ATOM 3793 C1B POP 370 8.530 7.210 49.800 1.00 0.00

ATOM 3794 C2B POP 370 6.360 8.020 53.520 1.00 0.00

ATOM 3795 D3B POP 370 7.420 7.400 57.860 1.00 0.00

ATOM 3796 C4B POP 370 8.280 7.080 63.050 1.00 0.00

ATOM 3797 C5B POP 370 12.990 6.740 63.710 1.00 0.00

ATOM 3798 NCO POP 371 34.500 42.000 84.860 1.00 0.00

ATOM 3799 PO4 POP 371 30.950 42.880 87.870 1.00 0.00

ATOM 3800 GL1 POP 371 30.830 46.020 84.060 1.00 0.00

ATOM 3801 GL2 POP 371 32.700 49.940 86.300 1.00 0.00

ATOM 3802 C1A POP 371 34.910 52.320 82.740 1.00 0.00

ATOM 3803 C2A POP 371 33.790 55.950 80.310 1.00 0.00

ATOM 3804 C3A POP 371 31.680 58.300 76.870 1.00 0.00

ATOM 3805 C4A POP 371 31.770 58.580 72.080 1.00 0.00

ATOM 3806 C1B POP 371 28.750 45.560 79.970 1.00 0.00

ATOM 3807 C2B POP 371 29.180 48.690 75.810 1.00 0.00

ATOM 3808 D3B POP 371 28.450 46.770 71.480 1.00 0.00

ATOM 3809 C4B POP 371 24.890 46.360 68.940 1.00 0.00

ATOM 3810 C5B POP 371 24.890 43.280 72.160 1.00 0.00

ATOM 3811 NCO POP 372 70.170 86.610 42.900 1.00 0.00

ATOM 3812 PO4 POP 372 68.880 83.940 45.040 1.00 0.00

ATOM 3813 GL1 POP 372 67.910 86.130 48.630 1.00 0.00

ATOM 3814 GL2 POP 372 66.750 82.150 51.160 1.00 0.00

ATOM 3815 C1A POP 372 68.000 84.560 53.460 1.00 0.00

ATOM 3816 C2A POP 372 69.800 87.740 56.750 1.00 0.00

ATOM 3817 C3A POP 372 69.180 88.800 61.340 1.00 0.00

ATOM 3818 C4A POP 372 65.750 88.830 64.410 1.00 0.00

ATOM 3819 C1B POP 372 68.930 89.620 52.180 1.00 0.00

ATOM 3820 C2B POP 372 68.380 92.310 55.800 1.00 0.00

ATOM 3821 D3B POP 372 65.450 93.580 58.580 1.00 0.00

ATOM 3822 C4B POP 372 64.460 92.950 61.970 1.00 0.00

ATOM 3823 C5B POP 372 63.980 94.550 66.400 1.00 0.00

ATOM 3824 NCO POP 373 9.770 32.730 94.370 1.00 0.00

ATOM 3825 PO4 POP 373 9.590 30.090 90.330 1.00 0.00

ATOM 3826 GL1 POP 373 11.990 30.860 86.360 1.00 0.00

ATOM 3827 GL2 POP 373 7.310 31.250 85.000 1.00 0.00

ATOM 3828 C1A POP 373 8.510 31.650 80.330 1.00 0.00

ATOM 3829 C2A POP 373 10.130 33.620 76.400 1.00 0.00

ATOM 3830 C3A POP 373 9.620 33.510 72.750 1.00 0.00

ATOM 3831 C4A POP 373 7.200 35.450 69.090 1.00 0.00

ATOM 3832 C1B POP 373 12.880 30.840 82.690 1.00 0.00

ATOM 3833 C2B POP 373 13.880 30.590 77.520 1.00 0.00

ATOM 3834 D3B POP 373 13.580 30.840 72.560 1.00 0.00

ATOM 3835 C4B POP 373 16.440 32.900 70.010 1.00 0.00

ATOM 3836 C5B POP 373 16.610 38.340 69.770 1.00 0.00

ATOM 3837 NCO POP 374 15.390 107.440 83.870 1.00 0.00

ATOM 3838 PO4 POP 374 18.610 105.710 85.630 1.00 0.00

ATOM 3839 GL1 POP 374 20.340 105.460 81.710 1.00 0.00

ATOM 3840 GL2 POP 374 20.990 109.650 80.250 1.00 0.00

ATOM 3841 C1A POP 374 16.760 108.150 78.990 1.00 0.00

ATOM 3842 C2A POP 374 16.080 104.950 77.520 1.00 0.00

ATOM 3843 C3A POP 374 15.100 100.510 75.930 1.00 0.00

ATOM 3844 C4A POP 374 16.760 97.630 73.270 1.00 0.00

ATOM 3845 C1B POP 374 20.660 104.400 77.840 1.00 0.00

ATOM 3846 C2B POP 374 20.780 107.310 73.750 1.00 0.00

ATOM 3847 D3B POP 374 20.090 111.510 70.940 1.00 0.00

ATOM 3848 C4B POP 374 18.670 114.260 67.450 1.00 0.00

ATOM 3849 C5B POP 374 18.800 117.370 62.520 1.00 0.00

ATOM 3850 NCO POP 375 91.970 95.990 94.370 1.00 0.00

ATOM 3851 PO4 POP 375 95.490 94.630 92.730 1.00 0.00

ATOM 3852 GL1 POP 375 97.240 96.520 89.140 1.00 0.00

ATOM 3853 GL2 POP 375 97.150 100.640 86.360 1.00 0.00

ATOM 3854 C1A POP 375 94.370 98.830 82.220 1.00 0.00

ATOM 3855 C2A POP 375 91.810 100.560 78.550 1.00 0.00

ATOM 3856 C3A POP 375 87.710 100.230 76.340 1.00 0.00

ATOM 3857 C4A POP 375 86.420 98.070 73.610 1.00 0.00

ATOM 3858 C1B POP 375 98.180 96.050 84.210 1.00 0.00

ATOM 3859 C2B POP 375 99.800 94.070 80.960 1.00 0.00

ATOM 3860 D3B POP 375 99.840 92.150 76.680 1.00 0.00

ATOM 3861 C4B POP 375 102.230 90.710 73.630 1.00 0.00

ATOM 3862 C5B POP 375 101.690 89.850 69.320 1.00 0.00

ATOM 3863 NCO POP 376 62.780 53.170 86.680 1.00 0.00

ATOM 3864 PO4 POP 376 67.630 53.110 86.800 1.00 0.00

ATOM 3865 GL1 POP 376 69.500 53.770 82.740 1.00 0.00

ATOM 3866 GL2 POP 376 66.060 54.740 79.630 1.00 0.00

ATOM 3867 C1A POP 376 61.590 56.960 77.610 1.00 0.00

ATOM 3868 C2A POP 376 60.480 57.550 72.110 1.00 0.00

ATOM 3869 C3A POP 376 58.150 57.790 68.430 1.00 0.00

ATOM 3870 C4A POP 376 55.080 56.150 66.720 1.00 0.00

ATOM 3871 C1B POP 376 70.680 52.780 78.880 1.00 0.00

ATOM 3872 C2B POP 376 73.540 52.050 75.830 1.00 0.00

ATOM 3873 D3B POP 376 76.030 51.810 72.790 1.00 0.00

ATOM 3874 C4B POP 376 77.170 51.060 69.100 1.00 0.00

ATOM 3875 C5B POP 376 75.950 53.130 66.290 1.00 0.00

ATOM 3876 NCO POP 377 -0.580 59.740 88.420 1.00 0.00

ATOM 3877 PO4 POP 377 -1.890 63.700 89.840 1.00 0.00

ATOM 3878 GL1 POP 377 -1.550 67.700 87.450 1.00 0.00

ATOM 3879 GL2 POP 377 0.430 71.760 87.810 1.00 0.00

ATOM 3880 C1A POP 377 3.510 72.720 83.530 1.00 0.00

ATOM 3881 C2A POP 377 5.120 72.350 79.490 1.00 0.00

ATOM 3882 C3A POP 377 5.580 71.800 74.640 1.00 0.00

ATOM 3883 C4A POP 377 6.130 68.990 71.560 1.00 0.00

ATOM 3884 C1B POP 377 -1.450 70.720 82.830 1.00 0.00

ATOM 3885 C2B POP 377 -0.630 70.910 78.630 1.00 0.00

ATOM 3886 D3B POP 377 0.630 73.750 75.640 1.00 0.00

ATOM 3887 C4B POP 377 -0.790 78.260 75.220 1.00 0.00

ATOM 3888 C5B POP 377 -4.670 78.050 74.870 1.00 0.00

ATOM 3889 NCO POP 378 68.200 51.020 48.350 1.00 0.00

ATOM 3890 PO4 POP 378 72.700 48.890 47.170 1.00 0.00

ATOM 3891 GL1 POP 378 74.010 50.000 51.790 1.00 0.00

ATOM 3892 GL2 POP 378 69.710 50.590 52.930 1.00 0.00

ATOM 3893 C1A POP 378 66.010 51.310 54.420 1.00 0.00

ATOM 3894 C2A POP 378 64.380 51.030 58.460 1.00 0.00

ATOM 3895 C3A POP 378 64.560 50.870 62.690 1.00 0.00

ATOM 3896 C4A POP 378 62.010 48.870 66.710 1.00 0.00

ATOM 3897 C1B POP 378 73.950 50.770 56.270 1.00 0.00

ATOM 3898 C2B POP 378 76.600 51.190 58.360 1.00 0.00

ATOM 3899 D3B POP 378 79.140 51.450 61.420 1.00 0.00

ATOM 3900 C4B POP 378 83.750 50.840 62.260 1.00 0.00

ATOM 3901 C5B POP 378 82.280 55.210 61.610 1.00 0.00

ATOM 3902 NCO POP 379 63.580 7.610 86.480 1.00 0.00

ATOM 3903 PO4 POP 379 61.820 10.340 82.820 1.00 0.00

ATOM 3904 GL1 POP 379 60.420 12.670 78.650 1.00 0.00

ATOM 3905 GL2 POP 379 58.710 16.130 78.980 1.00 0.00

ATOM 3906 C1A POP 379 60.480 18.230 75.390 1.00 0.00

ATOM 3907 C2A POP 379 60.360 16.690 72.200 1.00 0.00

ATOM 3908 C3A POP 379 62.800 15.410 68.780 1.00 0.00

ATOM 3909 C4A POP 379 62.780 15.610 63.920 1.00 0.00

ATOM 3910 C1B POP 379 58.870 12.290 74.390 1.00 0.00

ATOM 3911 C2B POP 379 63.550 12.260 72.840 1.00 0.00

ATOM 3912 D3B POP 379 67.530 12.250 73.550 1.00 0.00

ATOM 3913 C4B POP 379 70.990 11.410 71.100 1.00 0.00

ATOM 3914 C5B POP 379 72.450 13.950 67.600 1.00 0.00

ATOM 3915 NCO POP 380 27.050 30.960 44.760 1.00 0.00

ATOM 3916 PO4 POP 380 24.410 27.200 43.960 1.00 0.00

ATOM 3917 GL1 POP 380 24.900 26.430 47.760 1.00 0.00

ATOM 3918 GL2 POP 380 21.080 26.290 48.740 1.00 0.00

ATOM 3919 C1A POP 380 20.750 26.850 53.040 1.00 0.00

ATOM 3920 C2A POP 380 22.710 28.550 56.440 1.00 0.00

ATOM 3921 C3A POP 380 24.790 32.620 58.370 1.00 0.00

ATOM 3922 C4A POP 380 25.100 34.770 60.220 1.00 0.00

ATOM 3923 C1B POP 380 26.030 24.000 51.950 1.00 0.00

ATOM 3924 C2B POP 380 24.270 24.130 56.670 1.00 0.00

ATOM 3925 D3B POP 380 22.860 20.450 59.030 1.00 0.00

ATOM 3926 C4B POP 380 21.560 18.710 63.270 1.00 0.00

ATOM 3927 C5B POP 380 21.020 21.250 67.540 1.00 0.00

ATOM 3928 NCO POP 381 103.180 67.320 46.620 1.00 0.00

ATOM 3929 PO4 POP 381 98.940 67.090 47.460 1.00 0.00

ATOM 3930 GL1 POP 381 101.000 66.720 51.100 1.00 0.00

ATOM 3931 GL2 POP 381 100.920 71.340 52.630 1.00 0.00

ATOM 3932 C1A POP 381 96.860 68.950 55.430 1.00 0.00

ATOM 3933 C2A POP 381 95.260 66.140 57.610 1.00 0.00

ATOM 3934 C3A POP 381 93.670 63.210 59.980 1.00 0.00

ATOM 3935 C4A POP 381 90.700 60.610 62.740 1.00 0.00

ATOM 3936 C1B POP 381 102.210 66.460 54.850 1.00 0.00

ATOM 3937 C2B POP 381 104.630 65.340 57.130 1.00 0.00

ATOM 3938 D3B POP 381 106.140 63.660 60.640 1.00 0.00

ATOM 3939 C4B POP 381 105.010 61.780 65.280 1.00 0.00

ATOM 3940 C5B POP 381 107.240 59.810 69.230 1.00 0.00

ATOM 3941 NCO POP 382 104.730 53.800 94.150 1.00 0.00

ATOM 3942 PO4 POP 382 103.320 52.410 91.370 1.00 0.00

ATOM 3943 GL1 POP 382 104.110 55.750 87.950 1.00 0.00

ATOM 3944 GL2 POP 382 106.910 56.170 84.970 1.00 0.00

ATOM 3945 C1A POP 382 107.510 54.940 80.820 1.00 0.00

ATOM 3946 C2A POP 382 108.080 52.500 77.660 1.00 0.00

ATOM 3947 C3A POP 382 109.040 51.570 73.130 1.00 0.00

ATOM 3948 C4A POP 382 111.350 47.510 71.760 1.00 0.00

ATOM 3949 C1B POP 382 102.150 57.810 86.510 1.00 0.00

ATOM 3950 C2B POP 382 103.400 57.950 81.740 1.00 0.00

ATOM 3951 D3B POP 382 104.880 60.110 78.730 1.00 0.00

ATOM 3952 C4B POP 382 106.750 63.580 78.060 1.00 0.00

ATOM 3953 C5B POP 382 108.380 65.720 81.170 1.00 0.00

ATOM 3954 NCO POP 383 104.670 50.390 44.170 1.00 0.00

ATOM 3955 PO4 POP 383 102.890 53.510 47.220 1.00 0.00

ATOM 3956 GL1 POP 383 103.290 54.900 52.190 1.00 0.00

ATOM 3957 GL2 POP 383 106.950 56.430 53.590 1.00 0.00

ATOM 3958 C1A POP 383 109.040 55.020 56.670 1.00 0.00

ATOM 3959 C2A POP 383 109.820 56.980 60.500 1.00 0.00

ATOM 3960 C3A POP 383 113.400 57.790 62.680 1.00 0.00

ATOM 3961 C4A POP 383 113.660 55.650 65.570 1.00 0.00

ATOM 3962 C1B POP 383 103.970 54.050 56.420 1.00 0.00

ATOM 3963 C2B POP 383 100.760 56.690 59.820 1.00 0.00

ATOM 3964 D3B POP 383 100.390 57.830 63.970 1.00 0.00

ATOM 3965 C4B POP 383 103.520 57.020 67.380 1.00 0.00

ATOM 3966 C5B POP 383 104.700 53.260 70.560 1.00 0.00

ATOM 3967 NCO POP 384 44.680 54.670 40.140 1.00 0.00

ATOM 3968 PO4 POP 384 45.470 52.840 44.440 1.00 0.00

ATOM 3969 GL1 POP 384 47.960 54.960 47.800 1.00 0.00

ATOM 3970 GL2 POP 384 51.840 54.710 48.840 1.00 0.00

ATOM 3971 C1A POP 384 54.090 51.340 51.580 1.00 0.00

ATOM 3972 C2A POP 384 55.550 51.140 55.710 1.00 0.00

ATOM 3973 C3A POP 384 52.060 50.950 58.550 1.00 0.00

ATOM 3974 C4A POP 384 50.500 53.270 62.010 1.00 0.00

ATOM 3975 C1B POP 384 47.210 56.650 50.720 1.00 0.00

ATOM 3976 C2B POP 384 45.510 55.510 55.760 1.00 0.00

ATOM 3977 D3B POP 384 43.230 57.440 60.430 1.00 0.00

ATOM 3978 C4B POP 384 41.550 57.740 64.910 1.00 0.00

ATOM 3979 C5B POP 384 38.410 60.500 65.900 1.00 0.00

ATOM 3980 NCO POP 385 89.570 86.850 91.200 1.00 0.00

ATOM 3981 PO4 POP 385 88.670 82.510 89.670 1.00 0.00

ATOM 3982 GL1 POP 385 91.730 82.510 86.320 1.00 0.00

ATOM 3983 GL2 POP 385 92.340 78.690 82.910 1.00 0.00

ATOM 3984 C1A POP 385 96.020 81.860 83.570 1.00 0.00

ATOM 3985 C2A POP 385 98.350 83.360 80.940 1.00 0.00

ATOM 3986 C3A POP 385 100.380 85.340 77.350 1.00 0.00

ATOM 3987 C4A POP 385 100.570 85.760 73.210 1.00 0.00

ATOM 3988 C1B POP 385 92.590 85.290 82.280 1.00 0.00

ATOM 3989 C2B POP 385 90.300 86.110 78.930 1.00 0.00

ATOM 3990 D3B POP 385 88.070 86.900 74.990 1.00 0.00

ATOM 3991 C4B POP 385 88.730 89.050 70.770 1.00 0.00

ATOM 3992 C5B POP 385 92.140 89.660 68.820 1.00 0.00

ATOM 3993 NCO POP 386 69.780 108.860 89.040 1.00 0.00

ATOM 3994 PO4 POP 386 68.950 107.370 84.790 1.00 0.00

ATOM 3995 GL1 POP 386 71.960 106.010 81.510 1.00 0.00

ATOM 3996 GL2 POP 386 73.830 109.780 79.880 1.00 0.00

ATOM 3997 C1A POP 386 74.420 110.720 75.360 1.00 0.00

ATOM 3998 C2A POP 386 75.610 112.300 70.340 1.00 0.00

ATOM 3999 C3A POP 386 75.290 116.320 69.650 1.00 0.00

ATOM 4000 C4A POP 386 72.710 118.320 66.790 1.00 0.00

ATOM 4001 C1B POP 386 71.870 104.220 76.570 1.00 0.00

ATOM 4002 C2B POP 386 72.330 101.600 74.400 1.00 0.00

ATOM 4003 D3B POP 386 71.390 98.590 72.120 1.00 0.00

ATOM 4004 C4B POP 386 71.100 97.660 67.580 1.00 0.00

ATOM 4005 C5B POP 386 73.750 94.650 65.940 1.00 0.00

ATOM 4006 NCO POP 387 78.050 87.160 89.780 1.00 0.00

ATOM 4007 PO4 POP 387 75.750 83.290 87.490 1.00 0.00

ATOM 4008 GL1 POP 387 80.110 83.890 86.470 1.00 0.00

ATOM 4009 GL2 POP 387 81.150 88.180 85.590 1.00 0.00

ATOM 4010 C1A POP 387 80.780 86.270 82.100 1.00 0.00

ATOM 4011 C2A POP 387 80.750 88.280 79.560 1.00 0.00

ATOM 4012 C3A POP 387 80.680 91.840 77.480 1.00 0.00

ATOM 4013 C4A POP 387 83.560 96.080 77.180 1.00 0.00

ATOM 4014 C1B POP 387 82.620 81.820 82.910 1.00 0.00

ATOM 4015 C2B POP 387 84.030 79.410 79.500 1.00 0.00

ATOM 4016 D3B POP 387 87.230 76.020 77.090 1.00 0.00

ATOM 4017 C4B POP 387 88.410 76.600 72.560 1.00 0.00

ATOM 4018 C5B POP 387 90.810 78.520 69.500 1.00 0.00

ATOM 4019 NCO POP 388 34.240 23.410 48.130 1.00 0.00

ATOM 4020 PO4 POP 388 36.290 25.830 44.600 1.00 0.00

ATOM 4021 GL1 POP 388 38.840 26.420 48.160 1.00 0.00

ATOM 4022 GL2 POP 388 42.310 23.550 49.410 1.00 0.00

ATOM 4023 C1A POP 388 38.980 23.520 52.010 1.00 0.00

ATOM 4024 C2A POP 388 37.920 26.980 55.500 1.00 0.00

ATOM 4025 C3A POP 388 39.120 31.010 56.130 1.00 0.00

ATOM 4026 C4A POP 388 42.010 31.880 59.200 1.00 0.00

ATOM 4027 C1B POP 388 42.570 27.950 51.390 1.00 0.00

ATOM 4028 C2B POP 388 44.380 29.020 54.950 1.00 0.00

ATOM 4029 D3B POP 388 47.090 32.350 56.910 1.00 0.00

ATOM 4030 C4B POP 388 49.640 34.660 59.430 1.00 0.00

ATOM 4031 C5B POP 388 52.400 33.150 56.570 1.00 0.00

ATOM 4032 NCO POP 389 99.120 99.310 37.860 1.00 0.00

ATOM 4033 PO4 POP 389 94.600 99.820 40.150 1.00 0.00

ATOM 4034 GL1 POP 389 93.070 100.140 44.870 1.00 0.00

ATOM 4035 GL2 POP 389 95.730 98.360 47.730 1.00 0.00

ATOM 4036 C1A POP 389 97.890 99.870 51.460 1.00 0.00

ATOM 4037 C2A POP 389 98.330 99.140 55.070 1.00 0.00

ATOM 4038 C3A POP 389 102.050 99.120 57.830 1.00 0.00

ATOM 4039 C4A POP 389 103.130 102.340 61.610 1.00 0.00

ATOM 4040 C1B POP 389 91.990 103.050 48.550 1.00 0.00

ATOM 4041 C2B POP 389 89.870 103.160 51.640 1.00 0.00

ATOM 4042 D3B POP 389 90.840 101.560 55.280 1.00 0.00

ATOM 4043 C4B POP 389 91.090 101.160 60.310 1.00 0.00

ATOM 4044 C5B POP 389 94.630 101.090 59.300 1.00 0.00

ATOM 4045 NCO POP 390 27.380 90.160 44.570 1.00 0.00

ATOM 4046 PO4 POP 390 28.100 87.420 48.380 1.00 0.00

ATOM 4047 GL1 POP 390 31.580 85.150 50.660 1.00 0.00

ATOM 4048 GL2 POP 390 33.310 80.170 50.670 1.00 0.00

ATOM 4049 C1A POP 390 31.820 80.030 55.180 1.00 0.00

ATOM 4050 C2A POP 390 30.270 82.250 57.830 1.00 0.00

ATOM 4051 C3A POP 390 28.240 80.340 61.580 1.00 0.00

ATOM 4052 C4A POP 390 27.610 79.690 66.530 1.00 0.00

ATOM 4053 C1B POP 390 34.960 84.400 54.530 1.00 0.00

ATOM 4054 C2B POP 390 35.600 81.610 58.270 1.00 0.00

ATOM 4055 D3B POP 390 38.280 80.660 62.640 1.00 0.00

ATOM 4056 C4B POP 390 37.850 82.000 66.640 1.00 0.00

ATOM 4057 C5B POP 390 40.220 78.680 66.580 1.00 0.00

ATOM 4058 NCO POP 391 37.290 2.610 88.550 1.00 0.00

ATOM 4059 PO4 POP 391 41.010 2.410 85.450 1.00 0.00

ATOM 4060 GL1 POP 391 39.600 3.870 81.440 1.00 0.00

ATOM 4061 GL2 POP 391 41.310 2.330 78.400 1.00 0.00

ATOM 4062 C1A POP 391 42.350 1.370 75.240 1.00 0.00

ATOM 4063 C2A POP 391 45.040 -0.410 72.030 1.00 0.00

ATOM 4064 C3A POP 391 46.770 -4.330 69.810 1.00 0.00

ATOM 4065 C4A POP 391 50.590 -3.480 68.420 1.00 0.00

ATOM 4066 C1B POP 391 36.630 5.760 79.970 1.00 0.00

ATOM 4067 C2B POP 391 33.100 7.320 78.070 1.00 0.00

ATOM 4068 D3B POP 391 29.030 8.150 76.740 1.00 0.00

ATOM 4069 C4B POP 391 27.350 9.370 72.390 1.00 0.00

ATOM 4070 C5B POP 391 25.340 12.020 75.210 1.00 0.00

ATOM 4071 NCO POP 392 92.380 4.840 90.570 1.00 0.00

ATOM 4072 PO4 POP 392 94.340 7.820 88.240 1.00 0.00

ATOM 4073 GL1 POP 392 95.080 7.750 83.780 1.00 0.00

ATOM 4074 GL2 POP 392 93.080 3.950 82.420 1.00 0.00

ATOM 4075 C1A POP 392 89.400 5.410 81.140 1.00 0.00

ATOM 4076 C2A POP 392 87.560 4.190 77.000 1.00 0.00

ATOM 4077 C3A POP 392 88.580 2.760 72.600 1.00 0.00

ATOM 4078 C4A POP 392 89.360 -0.180 69.090 1.00 0.00

ATOM 4079 C1B POP 392 92.820 8.950 80.200 1.00 0.00

ATOM 4080 C2B POP 392 93.010 5.130 77.640 1.00 0.00

ATOM 4081 D3B POP 392 94.230 3.720 73.450 1.00 0.00

ATOM 4082 C4B POP 392 92.940 3.490 68.840 1.00 0.00

ATOM 4083 C5B POP 392 90.060 4.670 67.050 1.00 0.00

ATOM 4084 NCO POP 393 0.760 67.890 92.110 1.00 0.00

ATOM 4085 PO4 POP 393 4.550 69.270 91.090 1.00 0.00

ATOM 4086 GL1 POP 393 3.630 68.570 86.840 1.00 0.00

ATOM 4087 GL2 POP 393 1.540 64.310 85.880 1.00 0.00

ATOM 4088 C1A POP 393 1.410 61.010 83.950 1.00 0.00

ATOM 4089 C2A POP 393 -0.080 59.710 81.300 1.00 0.00

ATOM 4090 C3A POP 393 0.260 57.870 78.020 1.00 0.00

ATOM 4091 C4A POP 393 -2.290 56.250 74.180 1.00 0.00

ATOM 4092 C1B POP 393 3.100 67.650 82.640 1.00 0.00

ATOM 4093 C2B POP 393 3.880 63.920 80.780 1.00 0.00

ATOM 4094 D3B POP 393 2.350 62.760 77.100 1.00 0.00

ATOM 4095 C4B POP 393 -0.170 67.080 74.380 1.00 0.00

ATOM 4096 C5B POP 393 4.070 67.610 76.700 1.00 0.00

ATOM 4097 NCO POP 394 40.770 55.010 44.420 1.00 0.00

ATOM 4098 PO4 POP 394 37.520 54.620 47.840 1.00 0.00

ATOM 4099 GL1 POP 394 36.170 51.030 51.700 1.00 0.00

ATOM 4100 GL2 POP 394 32.580 48.420 49.990 1.00 0.00

ATOM 4101 C1A POP 394 32.720 46.980 54.780 1.00 0.00

ATOM 4102 C2A POP 394 34.600 45.160 58.980 1.00 0.00

ATOM 4103 C3A POP 394 38.200 46.010 61.560 1.00 0.00

ATOM 4104 C4A POP 394 40.840 46.450 65.740 1.00 0.00

ATOM 4105 C1B POP 394 36.910 48.760 56.040 1.00 0.00

ATOM 4106 C2B POP 394 36.850 50.510 60.510 1.00 0.00

ATOM 4107 D3B POP 394 39.590 52.240 63.550 1.00 0.00

ATOM 4108 C4B POP 394 42.450 52.690 66.530 1.00 0.00

ATOM 4109 C5B POP 394 45.010 56.010 67.730 1.00 0.00

ATOM 4110 NCO POP 395 61.580 78.700 41.560 1.00 0.00

ATOM 4111 PO4 POP 395 57.100 77.400 43.750 1.00 0.00

ATOM 4112 GL1 POP 395 54.480 76.990 47.020 1.00 0.00

ATOM 4113 GL2 POP 395 56.920 76.480 50.490 1.00 0.00

ATOM 4114 C1A POP 395 58.120 75.550 54.280 1.00 0.00

ATOM 4115 C2A POP 395 57.510 76.200 57.930 1.00 0.00

ATOM 4116 C3A POP 395 58.610 78.760 61.140 1.00 0.00

ATOM 4117 C4A POP 395 54.740 78.710 64.940 1.00 0.00

ATOM 4118 C1B POP 395 52.520 77.450 51.560 1.00 0.00

ATOM 4119 C2B POP 395 50.080 78.570 56.000 1.00 0.00

ATOM 4120 D3B POP 395 48.740 79.220 60.500 1.00 0.00

ATOM 4121 C4B POP 395 46.880 81.170 64.250 1.00 0.00

ATOM 4122 C5B POP 395 50.100 78.960 67.310 1.00 0.00

ATOM 4123 NCO POP 396 104.610 24.390 93.940 1.00 0.00

ATOM 4124 PO4 POP 396 105.510 26.230 89.450 1.00 0.00

ATOM 4125 GL1 POP 396 106.060 25.470 84.740 1.00 0.00

ATOM 4126 GL2 POP 396 107.940 28.540 82.100 1.00 0.00

ATOM 4127 C1A POP 396 103.760 28.690 80.190 1.00 0.00

ATOM 4128 C2A POP 396 100.700 25.580 79.580 1.00 0.00

ATOM 4129 C3A POP 396 99.320 24.380 75.010 1.00 0.00

ATOM 4130 C4A POP 396 100.710 26.170 70.330 1.00 0.00

ATOM 4131 C1B POP 396 106.690 24.250 79.890 1.00 0.00

ATOM 4132 C2B POP 396 106.640 25.850 75.570 1.00 0.00

ATOM 4133 D3B POP 396 106.120 26.820 71.870 1.00 0.00

ATOM 4134 C4B POP 396 105.750 23.880 68.980 1.00 0.00

ATOM 4135 C5B POP 396 110.210 26.880 69.320 1.00 0.00

ATOM 4136 NCO POP 397 78.870 28.880 48.130 1.00 0.00

ATOM 4137 PO4 POP 397 74.810 26.620 46.880 1.00 0.00

ATOM 4138 GL1 POP 397 76.230 25.080 50.840 1.00 0.00

ATOM 4139 GL2 POP 397 80.230 25.630 52.060 1.00 0.00

ATOM 4140 C1A POP 397 80.600 25.890 56.040 1.00 0.00

ATOM 4141 C2A POP 397 83.120 28.360 58.090 1.00 0.00

ATOM 4142 C3A POP 397 83.700 30.840 62.440 1.00 0.00

ATOM 4143 C4A POP 397 84.030 33.920 65.310 1.00 0.00

ATOM 4144 C1B POP 397 75.230 25.910 55.420 1.00 0.00

ATOM 4145 C2B POP 397 75.180 26.620 58.980 1.00 0.00

ATOM 4146 D3B POP 397 71.810 26.690 62.090 1.00 0.00

ATOM 4147 C4B POP 397 67.530 23.770 62.980 1.00 0.00

ATOM 4148 C5B POP 397 66.820 28.430 61.480 1.00 0.00

ATOM 4149 NCO POP 398 23.180 13.040 88.290 1.00 0.00

ATOM 4150 PO4 POP 398 25.050 12.110 83.570 1.00 0.00

ATOM 4151 GL1 POP 398 25.920 14.690 80.830 1.00 0.00

ATOM 4152 GL2 POP 398 26.470 19.300 80.920 1.00 0.00

ATOM 4153 C1A POP 398 26.680 21.080 76.440 1.00 0.00

ATOM 4154 C2A POP 398 29.590 22.320 73.500 1.00 0.00

ATOM 4155 C3A POP 398 29.080 20.340 70.700 1.00 0.00

ATOM 4156 C4A POP 398 27.880 17.860 67.670 1.00 0.00

ATOM 4157 C1B POP 398 23.300 16.400 77.400 1.00 0.00

ATOM 4158 C2B POP 398 19.270 16.280 74.510 1.00 0.00

ATOM 4159 D3B POP 398 16.640 14.310 72.860 1.00 0.00

ATOM 4160 C4B POP 398 12.260 11.880 73.320 1.00 0.00

ATOM 4161 C5B POP 398 12.370 8.680 69.970 1.00 0.00

ATOM 4162 NCO POP 399 24.600 69.330 46.020 1.00 0.00

ATOM 4163 PO4 POP 399 23.750 65.500 48.410 1.00 0.00

ATOM 4164 GL1 POP 399 24.550 66.920 51.900 1.00 0.00

ATOM 4165 GL2 POP 399 28.340 67.850 53.400 1.00 0.00

ATOM 4166 C1A POP 399 27.370 65.410 57.150 1.00 0.00

ATOM 4167 C2A POP 399 25.260 65.780 60.910 1.00 0.00

ATOM 4168 C3A POP 399 27.920 65.010 65.610 1.00 0.00

ATOM 4169 C4A POP 399 30.480 65.090 69.400 1.00 0.00

ATOM 4170 C1B POP 399 22.230 67.380 55.720 1.00 0.00

ATOM 4171 C2B POP 399 20.730 68.060 60.070 1.00 0.00

ATOM 4172 D3B POP 399 18.880 68.390 63.870 1.00 0.00

ATOM 4173 C4B POP 399 19.710 69.580 68.580 1.00 0.00

ATOM 4174 C5B POP 399 15.810 66.930 71.330 1.00 0.00

ATOM 4175 NCO POP 400 65.050 -1.520 43.910 1.00 0.00

ATOM 4176 PO4 POP 400 68.280 0.440 44.020 1.00 0.00

ATOM 4177 GL1 POP 400 68.200 1.180 48.040 1.00 0.00

ATOM 4178 GL2 POP 400 65.970 -0.890 51.050 1.00 0.00

ATOM 4179 C1A POP 400 63.440 -2.420 54.030 1.00 0.00

ATOM 4180 C2A POP 400 61.420 -1.120 57.930 1.00 0.00

ATOM 4181 C3A POP 400 60.750 1.280 60.500 1.00 0.00

ATOM 4182 C4A POP 400 58.930 0.340 63.600 1.00 0.00

ATOM 4183 C1B POP 400 70.010 2.180 51.440 1.00 0.00

ATOM 4184 C2B POP 400 70.980 4.900 55.300 1.00 0.00

ATOM 4185 D3B POP 400 71.090 3.910 59.540 1.00 0.00

ATOM 4186 C4B POP 400 72.000 0.960 62.500 1.00 0.00

ATOM 4187 C5B POP 400 75.850 -1.410 61.610 1.00 0.00

ATOM 4188 NCO POP 401 28.340 57.610 89.250 1.00 0.00

ATOM 4189 PO4 POP 401 25.050 60.330 89.070 1.00 0.00

ATOM 4190 GL1 POP 401 24.940 59.770 85.690 1.00 0.00

ATOM 4191 GL2 POP 401 25.520 56.080 84.600 1.00 0.00

ATOM 4192 C1A POP 401 24.830 54.580 80.740 1.00 0.00

ATOM 4193 C2A POP 401 24.690 53.340 75.790 1.00 0.00

ATOM 4194 C3A POP 401 21.740 53.040 72.910 1.00 0.00

ATOM 4195 C4A POP 401 19.120 54.350 69.430 1.00 0.00

ATOM 4196 C1B POP 401 24.030 62.600 81.010 1.00 0.00

ATOM 4197 C2B POP 401 23.980 63.680 75.790 1.00 0.00

ATOM 4198 D3B POP 401 23.060 65.470 71.360 1.00 0.00

ATOM 4199 C4B POP 401 23.030 66.340 66.660 1.00 0.00

ATOM 4200 C5B POP 401 19.400 64.260 69.070 1.00 0.00

ATOM 4201 NCO POP 402 44.720 97.600 43.150 1.00 0.00

ATOM 4202 PO4 POP 402 42.010 99.740 45.850 1.00 0.00

ATOM 4203 GL1 POP 402 41.180 98.340 49.730 1.00 0.00

ATOM 4204 GL2 POP 402 44.040 95.120 51.920 1.00 0.00

ATOM 4205 C1A POP 402 43.600 94.950 55.870 1.00 0.00

ATOM 4206 C2A POP 402 45.630 94.270 58.230 1.00 0.00

ATOM 4207 C3A POP 402 49.030 93.980 60.350 1.00 0.00

ATOM 4208 C4A POP 402 52.780 92.560 61.340 1.00 0.00

ATOM 4209 C1B POP 402 40.480 99.740 53.930 1.00 0.00

ATOM 4210 C2B POP 402 40.610 99.390 58.240 1.00 0.00

ATOM 4211 D3B POP 402 44.110 99.230 60.180 1.00 0.00

ATOM 4212 C4B POP 402 45.300 95.770 63.270 1.00 0.00

ATOM 4213 C5B POP 402 41.210 94.700 60.790 1.00 0.00

ATOM 4214 NCO POP 403 28.020 109.840 86.020 1.00 0.00

ATOM 4215 PO4 POP 403 31.450 106.280 86.570 1.00 0.00

ATOM 4216 GL1 POP 403 31.430 108.800 81.990 1.00 0.00

ATOM 4217 GL2 POP 403 32.020 112.770 80.670 1.00 0.00

ATOM 4218 C1A POP 403 31.160 111.310 76.350 1.00 0.00

ATOM 4219 C2A POP 403 30.640 109.800 73.310 1.00 0.00

ATOM 4220 C3A POP 403 30.470 111.580 69.840 1.00 0.00

ATOM 4221 C4A POP 403 32.390 109.510 65.880 1.00 0.00

ATOM 4222 C1B POP 403 29.460 106.570 77.600 1.00 0.00

ATOM 4223 C2B POP 403 30.920 103.990 75.370 1.00 0.00

ATOM 4224 D3B POP 403 32.690 101.370 71.580 1.00 0.00

ATOM 4225 C4B POP 403 33.900 97.480 70.390 1.00 0.00

ATOM 4226 C5B POP 403 30.930 96.890 67.600 1.00 0.00

ATOM 4227 NCO POP 404 81.190 58.030 94.870 1.00 0.00

ATOM 4228 PO4 POP 404 81.180 57.610 90.080 1.00 0.00

ATOM 4229 GL1 POP 404 82.150 56.180 86.440 1.00 0.00

ATOM 4230 GL2 POP 404 82.490 59.310 83.080 1.00 0.00

ATOM 4231 C1A POP 404 84.350 60.020 79.370 1.00 0.00

ATOM 4232 C2A POP 404 84.540 60.430 74.990 1.00 0.00

ATOM 4233 C3A POP 404 88.640 60.620 71.610 1.00 0.00

ATOM 4234 C4A POP 404 91.140 59.820 68.280 1.00 0.00

ATOM 4235 C1B POP 404 82.270 54.080 81.820 1.00 0.00

ATOM 4236 C2B POP 404 79.660 53.290 77.590 1.00 0.00

ATOM 4237 D3B POP 404 80.420 54.440 73.420 1.00 0.00

ATOM 4238 C4B POP 404 79.510 55.400 69.500 1.00 0.00

ATOM 4239 C5B POP 404 81.230 52.950 65.950 1.00 0.00

ATOM 4240 NCO POP 405 43.190 26.980 45.780 1.00 0.00

ATOM 4241 PO4 POP 405 46.820 29.100 43.790 1.00 0.00

ATOM 4242 GL1 POP 405 46.540 29.990 48.330 1.00 0.00

ATOM 4243 GL2 POP 405 49.920 28.720 51.220 1.00 0.00

ATOM 4244 C1A POP 405 51.130 28.500 55.230 1.00 0.00

ATOM 4245 C2A POP 405 51.440 29.550 59.950 1.00 0.00

ATOM 4246 C3A POP 405 50.430 30.880 64.450 1.00 0.00

ATOM 4247 C4A POP 405 53.950 32.300 68.590 1.00 0.00

ATOM 4248 C1B POP 405 45.590 32.240 51.770 1.00 0.00

ATOM 4249 C2B POP 405 43.310 35.200 55.450 1.00 0.00

ATOM 4250 D3B POP 405 45.160 36.700 58.680 1.00 0.00

ATOM 4251 C4B POP 405 47.200 40.220 60.790 1.00 0.00

ATOM 4252 C5B POP 405 43.360 36.550 63.460 1.00 0.00

ATOM 4253 NCO POP 406 25.240 24.180 88.580 1.00 0.00

ATOM 4254 PO4 POP 406 24.090 26.210 84.970 1.00 0.00

ATOM 4255 GL1 POP 406 23.930 28.550 81.220 1.00 0.00

ATOM 4256 GL2 POP 406 22.180 33.440 83.530 1.00 0.00

ATOM 4257 C1A POP 406 19.250 30.860 79.940 1.00 0.00

ATOM 4258 C2A POP 406 16.150 26.830 79.960 1.00 0.00

ATOM 4259 C3A POP 406 11.100 27.000 80.440 1.00 0.00

ATOM 4260 C4A POP 406 7.980 23.550 82.340 1.00 0.00

ATOM 4261 C1B POP 406 22.940 28.330 77.240 1.00 0.00

ATOM 4262 C2B POP 406 22.050 30.750 73.280 1.00 0.00

ATOM 4263 D3B POP 406 20.920 34.780 70.170 1.00 0.00

ATOM 4264 C4B POP 406 22.770 38.500 70.500 1.00 0.00

ATOM 4265 C5B POP 406 24.570 35.170 67.050 1.00 0.00

ATOM 4266 NCO POP 407 48.170 78.460 90.790 1.00 0.00

ATOM 4267 PO4 POP 407 48.290 79.790 86.860 1.00 0.00

ATOM 4268 GL1 POP 407 48.100 80.800 83.260 1.00 0.00

ATOM 4269 GL2 POP 407 49.070 84.760 82.020 1.00 0.00

ATOM 4270 C1A POP 407 45.510 84.690 80.060 1.00 0.00

ATOM 4271 C2A POP 407 41.900 82.530 78.240 1.00 0.00

ATOM 4272 C3A POP 407 37.670 83.000 75.290 1.00 0.00

ATOM 4273 C4A POP 407 35.720 84.320 71.470 1.00 0.00

ATOM 4274 C1B POP 407 45.780 79.510 79.280 1.00 0.00

ATOM 4275 C2B POP 407 43.510 77.330 76.310 1.00 0.00

ATOM 4276 D3B POP 407 45.180 75.370 72.950 1.00 0.00

ATOM 4277 C4B POP 407 47.970 74.400 69.560 1.00 0.00

ATOM 4278 C5B POP 407 50.510 74.100 73.220 1.00 0.00

ATOM 4279 NCO POP 408 98.030 18.960 44.580 1.00 0.00

ATOM 4280 PO4 POP 408 95.110 19.940 47.290 1.00 0.00

ATOM 4281 GL1 POP 408 95.550 17.140 49.540 1.00 0.00

ATOM 4282 GL2 POP 408 90.640 16.520 48.040 1.00 0.00

ATOM 4283 C1A POP 408 92.550 13.520 50.560 1.00 0.00

ATOM 4284 C2A POP 408 92.510 12.080 54.950 1.00 0.00

ATOM 4285 C3A POP 408 92.090 10.110 58.070 1.00 0.00

ATOM 4286 C4A POP 408 88.750 6.900 58.690 1.00 0.00

ATOM 4287 C1B POP 408 96.710 15.050 52.990 1.00 0.00

ATOM 4288 C2B POP 408 96.550 13.960 56.720 1.00 0.00

ATOM 4289 D3B POP 408 98.260 14.370 60.760 1.00 0.00

ATOM 4290 C4B POP 408 101.510 17.930 61.660 1.00 0.00

ATOM 4291 C5B POP 408 100.740 16.800 57.340 1.00 0.00

ATOM 4292 NCO POP 409 56.700 11.550 81.810 1.00 0.00

ATOM 4293 PO4 POP 409 53.240 9.300 83.610 1.00 0.00

ATOM 4294 GL1 POP 409 51.660 10.680 79.940 1.00 0.00

ATOM 4295 GL2 POP 409 54.380 12.830 76.850 1.00 0.00

ATOM 4296 C1A POP 409 55.640 15.680 73.820 1.00 0.00

ATOM 4297 C2A POP 409 54.930 17.340 70.890 1.00 0.00

ATOM 4298 C3A POP 409 53.650 18.900 66.320 1.00 0.00

ATOM 4299 C4A POP 409 56.280 21.890 64.070 1.00 0.00

ATOM 4300 C1B POP 409 49.480 13.150 77.320 1.00 0.00

ATOM 4301 C2B POP 409 51.310 13.630 73.160 1.00 0.00

ATOM 4302 D3B POP 409 50.780 14.560 68.320 1.00 0.00

ATOM 4303 C4B POP 409 47.850 15.950 65.670 1.00 0.00

ATOM 4304 C5B POP 409 47.630 18.390 69.490 1.00 0.00

ATOM 4305 NCO POP 410 36.700 10.490 90.130 1.00 0.00

ATOM 4306 PO4 POP 410 37.950 10.790 85.770 1.00 0.00

ATOM 4307 GL1 POP 410 39.610 10.900 81.090 1.00 0.00

ATOM 4308 GL2 POP 410 40.480 8.850 78.330 1.00 0.00

ATOM 4309 C1A POP 410 41.380 7.110 73.830 1.00 0.00

ATOM 4310 C2A POP 410 43.990 6.680 70.250 1.00 0.00

ATOM 4311 C3A POP 410 48.060 3.850 71.760 1.00 0.00

ATOM 4312 C4A POP 410 51.230 0.860 71.560 1.00 0.00

ATOM 4313 C1B POP 410 36.250 11.240 77.650 1.00 0.00

ATOM 4314 C2B POP 410 36.780 8.270 73.890 1.00 0.00

ATOM 4315 D3B POP 410 36.540 7.320 69.650 1.00 0.00

ATOM 4316 C4B POP 410 32.260 6.380 67.100 1.00 0.00

ATOM 4317 C5B POP 410 28.160 6.490 68.540 1.00 0.00

ATOM 4318 NCO POP 411 69.260 14.930 79.720 1.00 0.00

ATOM 4319 PO4 POP 411 73.600 16.020 79.470 1.00 0.00

ATOM 4320 GL1 POP 411 76.690 13.040 79.510 1.00 0.00

ATOM 4321 GL2 POP 411 81.530 14.230 81.330 1.00 0.00

ATOM 4322 C1A POP 411 83.510 15.120 76.590 1.00 0.00

ATOM 4323 C2A POP 411 82.010 13.830 71.780 1.00 0.00

ATOM 4324 C3A POP 411 82.970 14.460 67.690 1.00 0.00

ATOM 4325 C4A POP 411 81.630 12.870 63.360 1.00 0.00

ATOM 4326 C1B POP 411 80.150 11.100 76.520 1.00 0.00

ATOM 4327 C2B POP 411 84.770 10.570 74.050 1.00 0.00

ATOM 4328 D3B POP 411 88.560 12.720 72.210 1.00 0.00

ATOM 4329 C4B POP 411 92.460 12.860 71.160 1.00 0.00

ATOM 4330 C5B POP 411 92.770 14.040 66.980 1.00 0.00

ATOM 4331 NCO POP 412 112.900 18.840 46.990 1.00 0.00

ATOM 4332 PO4 POP 412 110.820 16.640 44.700 1.00 0.00

ATOM 4333 GL1 POP 412 109.490 15.740 49.070 1.00 0.00

ATOM 4334 GL2 POP 412 108.650 20.170 50.260 1.00 0.00

ATOM 4335 C1A POP 412 107.740 20.430 53.500 1.00 0.00

ATOM 4336 C2A POP 412 106.260 20.480 58.300 1.00 0.00

ATOM 4337 C3A POP 412 105.140 21.840 61.680 1.00 0.00

ATOM 4338 C4A POP 412 104.090 20.820 65.350 1.00 0.00

ATOM 4339 C1B POP 412 109.050 15.980 53.310 1.00 0.00

ATOM 4340 C2B POP 412 109.810 16.470 56.950 1.00 0.00

ATOM 4341 D3B POP 412 109.930 13.450 60.200 1.00 0.00

ATOM 4342 C4B POP 412 107.660 12.040 63.180 1.00 0.00

ATOM 4343 C5B POP 412 106.120 8.770 65.100 1.00 0.00

ATOM 4344 NCO POP 413 4.560 69.710 48.290 1.00 0.00

ATOM 4345 PO4 POP 413 7.810 70.560 49.730 1.00 0.00

ATOM 4346 GL1 POP 413 4.270 69.080 53.690 1.00 0.00

ATOM 4347 GL2 POP 413 1.910 66.910 51.860 1.00 0.00

ATOM 4348 C1A POP 413 0.600 65.820 56.610 1.00 0.00

ATOM 4349 C2A POP 413 0.440 66.830 60.870 1.00 0.00

ATOM 4350 C3A POP 413 -0.450 67.030 64.480 1.00 0.00

ATOM 4351 C4A POP 413 0.120 66.570 69.060 1.00 0.00

ATOM 4352 C1B POP 413 4.200 69.580 58.660 1.00 0.00

ATOM 4353 C2B POP 413 6.780 70.890 61.780 1.00 0.00

ATOM 4354 D3B POP 413 8.800 72.110 66.180 1.00 0.00

ATOM 4355 C4B POP 413 11.640 73.650 67.480 1.00 0.00

ATOM 4356 C5B POP 413 13.910 77.930 69.350 1.00 0.00

ATOM 4357 NCO POP 414 85.320 58.410 44.800 1.00 0.00

ATOM 4358 PO4 POP 414 83.870 60.310 47.360 1.00 0.00

ATOM 4359 GL1 POP 414 85.190 59.580 51.850 1.00 0.00

ATOM 4360 GL2 POP 414 82.820 59.080 55.970 1.00 0.00

ATOM 4361 C1A POP 414 82.290 60.260 60.870 1.00 0.00

ATOM 4362 C2A POP 414 81.190 62.210 64.500 1.00 0.00

ATOM 4363 C3A POP 414 78.920 65.910 65.890 1.00 0.00

ATOM 4364 C4A POP 414 75.690 67.540 69.130 1.00 0.00

ATOM 4365 C1B POP 414 88.700 60.130 55.030 1.00 0.00

ATOM 4366 C2B POP 414 91.310 57.590 58.580 1.00 0.00

ATOM 4367 D3B POP 414 90.970 55.370 63.080 1.00 0.00

ATOM 4368 C4B POP 414 89.290 55.500 67.120 1.00 0.00

ATOM 4369 C5B POP 414 88.970 52.210 71.540 1.00 0.00

ATOM 4370 NCO POP 415 109.330 102.580 44.000 1.00 0.00

ATOM 4371 PO4 POP 415 104.750 103.550 41.910 1.00 0.00

ATOM 4372 GL1 POP 415 104.060 103.620 47.230 1.00 0.00

ATOM 4373 GL2 POP 415 103.730 108.700 47.750 1.00 0.00

ATOM 4374 C1A POP 415 108.160 105.700 48.220 1.00 0.00

ATOM 4375 C2A POP 415 112.060 105.210 50.510 1.00 0.00

ATOM 4376 C3A POP 415 114.550 102.010 50.500 1.00 0.00

ATOM 4377 C4A POP 415 114.070 98.850 52.310 1.00 0.00

ATOM 4378 C1B POP 415 101.260 103.940 50.810 1.00 0.00

ATOM 4379 C2B POP 415 99.940 104.220 54.380 1.00 0.00

ATOM 4380 D3B POP 415 99.040 103.000 58.810 1.00 0.00

ATOM 4381 C4B POP 415 97.100 102.950 63.550 1.00 0.00

ATOM 4382 C5B POP 415 97.810 107.510 63.160 1.00 0.00

ATOM 4383 NCO POP 416 64.650 100.940 80.960 1.00 0.00

ATOM 4384 PO4 POP 416 64.370 104.360 84.070 1.00 0.00

ATOM 4385 GL1 POP 416 61.180 105.500 80.900 1.00 0.00

ATOM 4386 GL2 POP 416 59.550 109.310 79.700 1.00 0.00

ATOM 4387 C1A POP 416 63.080 107.830 75.810 1.00 0.00

ATOM 4388 C2A POP 416 61.410 109.700 71.780 1.00 0.00

ATOM 4389 C3A POP 416 62.370 113.650 69.670 1.00 0.00

ATOM 4390 C4A POP 416 62.620 112.740 65.040 1.00 0.00

ATOM 4391 C1B POP 416 59.490 104.030 76.780 1.00 0.00

ATOM 4392 C2B POP 416 58.950 104.900 72.400 1.00 0.00

ATOM 4393 D3B POP 416 54.960 106.600 71.560 1.00 0.00

ATOM 4394 C4B POP 416 56.410 111.010 70.660 1.00 0.00

ATOM 4395 C5B POP 416 57.800 113.570 68.230 1.00 0.00

ATOM 4396 NCO POP 417 39.620 41.330 87.310 1.00 0.00

ATOM 4397 PO4 POP 417 42.650 37.850 85.540 1.00 0.00

ATOM 4398 GL1 POP 417 42.710 40.010 82.530 1.00 0.00

ATOM 4399 GL2 POP 417 38.710 42.800 81.700 1.00 0.00

ATOM 4400 C1A POP 417 40.640 39.840 78.470 1.00 0.00

ATOM 4401 C2A POP 417 41.420 39.940 72.650 1.00 0.00

ATOM 4402 C3A POP 417 41.850 38.480 68.780 1.00 0.00

ATOM 4403 C4A POP 417 40.240 39.580 64.870 1.00 0.00

ATOM 4404 C1B POP 417 44.150 43.220 80.700 1.00 0.00

ATOM 4405 C2B POP 417 44.450 47.020 78.000 1.00 0.00

ATOM 4406 D3B POP 417 40.880 50.210 75.170 1.00 0.00

ATOM 4407 C4B POP 417 40.640 47.560 70.880 1.00 0.00

ATOM 4408 C5B POP 417 39.530 45.000 75.420 1.00 0.00

ATOM 4409 NCO POP 418 35.830 56.120 93.800 1.00 0.00

ATOM 4410 PO4 POP 418 37.420 56.580 90.350 1.00 0.00

ATOM 4411 GL1 POP 418 36.420 56.980 85.890 1.00 0.00

ATOM 4412 GL2 POP 418 32.760 55.770 86.600 1.00 0.00

ATOM 4413 C1A POP 418 30.010 54.450 82.830 1.00 0.00

ATOM 4414 C2A POP 418 29.080 53.860 78.140 1.00 0.00

ATOM 4415 C3A POP 418 29.840 53.360 72.890 1.00 0.00

ATOM 4416 C4A POP 418 28.330 55.810 69.150 1.00 0.00

ATOM 4417 C1B POP 418 37.390 58.860 81.450 1.00 0.00

ATOM 4418 C2B POP 418 39.600 57.580 78.920 1.00 0.00

ATOM 4419 D3B POP 418 39.400 58.960 74.600 1.00 0.00

ATOM 4420 C4B POP 418 36.970 58.350 71.080 1.00 0.00

ATOM 4421 C5B POP 418 41.740 58.190 70.480 1.00 0.00

ATOM 4422 NCO POP 419 111.550 81.160 88.360 1.00 0.00

ATOM 4423 PO4 POP 419 111.190 78.050 89.650 1.00 0.00

ATOM 4424 GL1 POP 419 110.500 76.350 85.460 1.00 0.00

ATOM 4425 GL2 POP 419 106.430 75.700 83.970 1.00 0.00

ATOM 4426 C1A POP 419 104.950 74.040 80.430 1.00 0.00

ATOM 4427 C2A POP 419 101.610 74.450 77.220 1.00 0.00

ATOM 4428 C3A POP 419 100.810 75.520 72.910 1.00 0.00

ATOM 4429 C4A POP 419 103.330 76.780 70.190 1.00 0.00

ATOM 4430 C1B POP 419 111.100 76.260 80.380 1.00 0.00

ATOM 4431 C2B POP 419 114.020 76.820 76.760 1.00 0.00

ATOM 4432 D3B POP 419 114.790 76.850 73.080 1.00 0.00

ATOM 4433 C4B POP 419 113.940 79.370 69.860 1.00 0.00

ATOM 4434 C5B POP 419 113.370 81.540 73.230 1.00 0.00

ATOM 4435 NCO POP 420 27.260 64.930 45.190 1.00 0.00

ATOM 4436 PO4 POP 420 30.350 61.510 45.700 1.00 0.00

ATOM 4437 GL1 POP 420 30.820 60.540 49.750 1.00 0.00

ATOM 4438 GL2 POP 420 27.910 63.600 51.190 1.00 0.00

ATOM 4439 C1A POP 420 28.620 61.100 54.000 1.00 0.00

ATOM 4440 C2A POP 420 30.030 61.730 57.770 1.00 0.00

ATOM 4441 C3A POP 420 29.420 62.480 61.550 1.00 0.00

ATOM 4442 C4A POP 420 32.920 64.450 64.180 1.00 0.00

ATOM 4443 C1B POP 420 33.770 60.660 54.230 1.00 0.00

ATOM 4444 C2B POP 420 35.460 59.860 58.890 1.00 0.00

ATOM 4445 D3B POP 420 33.910 58.970 63.780 1.00 0.00

ATOM 4446 C4B POP 420 32.440 56.820 67.270 1.00 0.00

ATOM 4447 C5B POP 420 29.490 59.400 65.650 1.00 0.00

ATOM 4448 NCO POP 421 5.850 48.500 90.060 1.00 0.00

ATOM 4449 PO4 POP 421 1.540 49.530 89.650 1.00 0.00

ATOM 4450 GL1 POP 421 2.230 47.290 85.570 1.00 0.00

ATOM 4451 GL2 POP 421 0.160 50.550 83.100 1.00 0.00

ATOM 4452 C1A POP 421 -1.930 48.270 80.560 1.00 0.00

ATOM 4453 C2A POP 421 -5.140 45.270 79.250 1.00 0.00

ATOM 4454 C3A POP 421 -8.000 42.510 80.590 1.00 0.00

ATOM 4455 C4A POP 421 -8.350 38.530 81.230 1.00 0.00

ATOM 4456 C1B POP 421 3.920 46.800 80.120 1.00 0.00

ATOM 4457 C2B POP 421 3.990 44.510 76.240 1.00 0.00

ATOM 4458 D3B POP 421 5.770 42.110 73.690 1.00 0.00

ATOM 4459 C4B POP 421 9.470 39.280 71.380 1.00 0.00

ATOM 4460 C5B POP 421 13.170 41.880 71.720 1.00 0.00

ATOM 4461 NCO POP 422 108.120 9.140 39.830 1.00 0.00

ATOM 4462 PO4 POP 422 106.570 9.190 43.560 1.00 0.00

ATOM 4463 GL1 POP 422 104.550 7.000 46.660 1.00 0.00

ATOM 4464 GL2 POP 422 103.370 10.400 49.450 1.00 0.00

ATOM 4465 C1A POP 422 105.860 9.010 53.320 1.00 0.00

ATOM 4466 C2A POP 422 106.990 9.740 58.300 1.00 0.00

ATOM 4467 C3A POP 422 106.370 6.570 60.790 1.00 0.00

ATOM 4468 C4A POP 422 107.550 4.330 64.100 1.00 0.00

ATOM 4469 C1B POP 422 104.730 4.170 49.370 1.00 0.00

ATOM 4470 C2B POP 422 102.650 1.680 52.150 1.00 0.00

ATOM 4471 D3B POP 422 101.320 0.260 56.790 1.00 0.00

ATOM 4472 C4B POP 422 101.300 -1.500 59.830 1.00 0.00

ATOM 4473 C5B POP 422 105.090 0.620 61.430 1.00 0.00

ATOM 4474 NCO POP 423 78.190 2.980 44.650 1.00 0.00

ATOM 4475 PO4 POP 423 80.440 5.720 42.910 1.00 0.00

ATOM 4476 GL1 POP 423 78.440 9.300 45.050 1.00 0.00

ATOM 4477 GL2 POP 423 80.520 13.680 46.460 1.00 0.00

ATOM 4478 C1A POP 423 81.470 13.560 51.050 1.00 0.00

ATOM 4479 C2A POP 423 82.430 14.830 54.890 1.00 0.00

ATOM 4480 C3A POP 423 84.040 15.770 58.810 1.00 0.00

ATOM 4481 C4A POP 423 82.610 18.150 62.410 1.00 0.00

ATOM 4482 C1B POP 423 77.170 10.220 49.760 1.00 0.00

ATOM 4483 C2B POP 423 78.410 11.030 54.280 1.00 0.00

ATOM 4484 D3B POP 423 80.550 10.350 58.820 1.00 0.00

ATOM 4485 C4B POP 423 80.700 8.460 61.290 1.00 0.00

ATOM 4486 C5B POP 423 84.940 9.680 61.050 1.00 0.00

ATOM 4487 NCO POP 424 4.720 98.880 85.550 1.00 0.00

ATOM 4488 PO4 POP 424 0.690 97.350 84.310 1.00 0.00

ATOM 4489 GL1 POP 424 3.370 94.990 81.820 1.00 0.00

ATOM 4490 GL2 POP 424 6.330 98.210 80.960 1.00 0.00

ATOM 4491 C1A POP 424 9.180 94.950 81.740 1.00 0.00

ATOM 4492 C2A POP 424 13.930 93.150 81.110 1.00 0.00

ATOM 4493 C3A POP 424 14.840 88.450 79.300 1.00 0.00

ATOM 4494 C4A POP 424 17.820 88.450 75.740 1.00 0.00

ATOM 4495 C1B POP 424 2.240 95.110 78.020 1.00 0.00

ATOM 4496 C2B POP 424 5.590 94.900 74.000 1.00 0.00

ATOM 4497 D3B POP 424 8.120 98.440 71.510 1.00 0.00

ATOM 4498 C4B POP 424 8.110 97.320 67.650 1.00 0.00

ATOM 4499 C5B POP 424 10.210 95.790 63.670 1.00 0.00

ATOM 4500 NCO POP 425 54.600 69.060 89.210 1.00 0.00

ATOM 4501 PO4 POP 425 53.130 71.020 86.620 1.00 0.00

ATOM 4502 GL1 POP 425 54.730 67.070 84.430 1.00 0.00

ATOM 4503 GL2 POP 425 51.650 67.040 81.840 1.00 0.00

ATOM 4504 C1A POP 425 51.880 69.640 78.310 1.00 0.00

ATOM 4505 C2A POP 425 51.590 69.040 74.780 1.00 0.00

ATOM 4506 C3A POP 425 51.050 65.710 71.720 1.00 0.00

ATOM 4507 C4A POP 425 49.850 60.840 71.730 1.00 0.00

ATOM 4508 C1B POP 425 56.770 64.450 80.810 1.00 0.00

ATOM 4509 C2B POP 425 58.980 61.670 77.620 1.00 0.00

ATOM 4510 D3B POP 425 57.980 60.720 75.120 1.00 0.00

ATOM 4511 C4B POP 425 54.070 59.610 73.250 1.00 0.00

ATOM 4512 C5B POP 425 54.470 62.610 70.190 1.00 0.00

ATOM 4513 NCO POP 426 12.170 30.700 46.890 1.00 0.00

ATOM 4514 PO4 POP 426 12.310 34.840 46.500 1.00 0.00

ATOM 4515 GL1 POP 426 14.160 34.070 49.880 1.00 0.00

ATOM 4516 GL2 POP 426 12.990 29.840 52.140 1.00 0.00

ATOM 4517 C1A POP 426 13.780 29.480 56.850 1.00 0.00

ATOM 4518 C2A POP 426 14.770 29.090 61.990 1.00 0.00

ATOM 4519 C3A POP 426 14.730 30.580 65.800 1.00 0.00

ATOM 4520 C4A POP 426 10.170 30.110 66.510 1.00 0.00

ATOM 4521 C1B POP 426 13.330 34.390 54.280 1.00 0.00

ATOM 4522 C2B POP 426 10.530 34.170 57.310 1.00 0.00

ATOM 4523 D3B POP 426 11.250 31.980 60.460 1.00 0.00

ATOM 4524 C4B POP 426 9.070 33.840 63.680 1.00 0.00

ATOM 4525 C5B POP 426 12.510 35.150 67.080 1.00 0.00

ATOM 4526 PO1 PIP 427 53.270 32.390 94.230 1.00 0.00

ATOM 4527 PO2 PIP 427 48.760 35.250 91.660 1.00 0.00

ATOM 4528 RP1 PIP 427 51.970 34.050 91.970 1.00 0.00

ATOM 4529 RP2 PIP 427 51.480 36.460 90.190 1.00 0.00

ATOM 4530 RP3 PIP 427 53.000 34.200 89.090 1.00 0.00

ATOM 4531 PO3 PIP 427 53.690 34.220 86.240 1.00 0.00

ATOM 4532 GL1 PIP 427 55.660 35.130 83.080 1.00 0.00

ATOM 4533 GL2 PIP 427 54.310 38.050 81.470 1.00 0.00

ATOM 4534 C1A PIP 427 52.250 39.500 77.820 1.00 0.00

ATOM 4535 C2A PIP 427 51.410 38.920 73.560 1.00 0.00

ATOM 4536 C3A PIP 427 52.740 37.600 70.280 1.00 0.00

ATOM 4537 C4A PIP 427 53.220 36.740 65.870 1.00 0.00

ATOM 4538 C1B PIP 427 54.190 33.730 78.500 1.00 0.00

ATOM 4539 C2B PIP 427 53.450 33.740 73.890 1.00 0.00

ATOM 4540 C3B PIP 427 51.510 29.320 72.700 1.00 0.00

ATOM 4541 C4B PIP 427 49.190 26.100 72.580 1.00 0.00

ATOM 4542 C5B PIP 427 47.640 23.960 68.460 1.00 0.00

ATOM 4543 PO1 PIP 428 81.680 8.090 94.880 1.00 0.00

ATOM 4544 PO2 PIP 428 83.420 12.250 91.050 1.00 0.00

ATOM 4545 RP1 PIP 428 83.470 8.860 92.640 1.00 0.00

ATOM 4546 RP2 PIP 428 85.240 9.790 90.420 1.00 0.00

ATOM 4547 RP3 PIP 428 83.900 7.170 90.420 1.00 0.00

ATOM 4548 PO3 PIP 428 83.990 5.750 87.310 1.00 0.00

ATOM 4549 GL1 PIP 428 84.670 5.460 83.120 1.00 0.00

ATOM 4550 GL2 PIP 428 84.910 1.820 80.350 1.00 0.00

ATOM 4551 C1A PIP 428 85.580 -0.200 76.620 1.00 0.00

ATOM 4552 C2A PIP 428 86.170 -2.490 72.760 1.00 0.00

ATOM 4553 C3A PIP 428 86.790 -6.480 71.620 1.00 0.00

ATOM 4554 C4A PIP 428 84.500 -9.530 68.580 1.00 0.00

ATOM 4555 C1B PIP 428 82.650 5.880 78.400 1.00 0.00

ATOM 4556 C2B PIP 428 83.220 5.740 74.520 1.00 0.00

ATOM 4557 C3B PIP 428 83.850 5.170 71.270 1.00 0.00

ATOM 4558 C4B PIP 428 85.170 3.200 67.840 1.00 0.00

ATOM 4559 C5B PIP 428 86.220 -0.700 65.240 1.00 0.00

ATOM 4560 PO1 PIP 429 16.350 41.900 93.360 1.00 0.00

ATOM 4561 PO2 PIP 429 21.050 40.010 93.910 1.00 0.00

ATOM 4562 RP1 PIP 429 18.770 41.490 91.890 1.00 0.00

ATOM 4563 RP2 PIP 429 21.940 41.500 91.500 1.00 0.00

ATOM 4564 RP3 PIP 429 20.110 42.160 89.400 1.00 0.00

ATOM 4565 PO3 PIP 429 21.540 42.700 86.760 1.00 0.00

ATOM 4566 GL1 PIP 429 20.500 40.460 83.910 1.00 0.00

ATOM 4567 GL2 PIP 429 18.370 43.610 83.130 1.00 0.00

ATOM 4568 C1A PIP 429 14.320 43.700 80.630 1.00 0.00

ATOM 4569 C2A PIP 429 12.660 39.900 80.510 1.00 0.00

ATOM 4570 C3A PIP 429 8.560 36.910 79.700 1.00 0.00

ATOM 4571 C4A PIP 429 6.290 36.500 75.370 1.00 0.00

ATOM 4572 C1B PIP 429 18.810 40.080 79.500 1.00 0.00

ATOM 4573 C2B PIP 429 16.230 41.860 76.280 1.00 0.00

ATOM 4574 C3B PIP 429 14.840 45.470 74.640 1.00 0.00

ATOM 4575 C4B PIP 429 12.540 48.920 76.350 1.00 0.00

ATOM 4576 C5B PIP 429 8.650 49.390 78.250 1.00 0.00

ATOM 4577 PO1 PIP 430 66.670 90.370 90.460 1.00 0.00

ATOM 4578 PO2 PIP 430 63.980 95.220 89.310 1.00 0.00

ATOM 4579 RP1 PIP 430 66.620 92.450 88.680 1.00 0.00

ATOM 4580 RP2 PIP 430 66.760 95.210 87.390 1.00 0.00

ATOM 4581 RP3 PIP 430 66.870 92.850 85.630 1.00 0.00

ATOM 4582 PO3 PIP 430 67.160 91.690 83.370 1.00 0.00

ATOM 4583 GL1 PIP 430 67.520 92.930 79.330 1.00 0.00

ATOM 4584 GL2 PIP 430 65.800 94.840 78.330 1.00 0.00

ATOM 4585 C1A PIP 430 62.370 94.760 76.200 1.00 0.00

ATOM 4586 C2A PIP 430 61.860 94.850 71.690 1.00 0.00

ATOM 4587 C3A PIP 430 61.010 91.810 70.580 1.00 0.00

ATOM 4588 C4A PIP 430 61.120 89.180 65.780 1.00 0.00

ATOM 4589 C1B PIP 430 70.550 94.040 76.230 1.00 0.00

ATOM 4590 C2B PIP 430 73.810 97.040 76.330 1.00 0.00

ATOM 4591 C3B PIP 430 75.800 100.830 77.520 1.00 0.00

ATOM 4592 C4B PIP 430 79.290 101.340 77.790 1.00 0.00

ATOM 4593 C5B PIP 430 82.610 101.100 74.990 1.00 0.00

ATOM 4594 PO1 PIP 431 34.580 59.740 96.730 1.00 0.00

ATOM 4595 PO2 PIP 431 36.230 63.880 100.740 1.00 0.00

ATOM 4596 RP1 PIP 431 35.450 62.560 97.930 1.00 0.00

ATOM 4597 RP2 PIP 431 38.260 63.860 98.250 1.00 0.00

ATOM 4598 RP3 PIP 431 36.580 64.960 95.970 1.00 0.00

ATOM 4599 PO3 PIP 431 36.750 66.850 93.410 1.00 0.00

ATOM 4600 GL1 PIP 431 35.920 64.530 90.360 1.00 0.00

ATOM 4601 GL2 PIP 431 33.400 65.790 89.020 1.00 0.00

ATOM 4602 C1A PIP 431 32.200 67.340 84.520 1.00 0.00

ATOM 4603 C2A PIP 431 32.090 71.070 81.350 1.00 0.00

ATOM 4604 C3A PIP 431 33.460 73.170 78.310 1.00 0.00

ATOM 4605 C4A PIP 431 34.060 72.620 75.050 1.00 0.00

ATOM 4606 C1B PIP 431 37.010 66.220 85.190 1.00 0.00

ATOM 4607 C2B PIP 431 39.730 67.680 82.770 1.00 0.00

ATOM 4608 C3B PIP 431 42.050 67.630 78.180 1.00 0.00

ATOM 4609 C4B PIP 431 41.330 65.840 74.020 1.00 0.00

ATOM 4610 C5B PIP 431 39.700 63.030 70.810 1.00 0.00

ATOM 4611 PO1 PIP 432 80.690 48.560 94.840 1.00 0.00

ATOM 4612 PO2 PIP 432 80.930 43.370 93.000 1.00 0.00

ATOM 4613 RP1 PIP 432 80.490 46.420 91.950 1.00 0.00

ATOM 4614 RP2 PIP 432 78.260 44.460 91.210 1.00 0.00

ATOM 4615 RP3 PIP 432 79.500 45.780 89.100 1.00 0.00

ATOM 4616 PO3 PIP 432 79.390 46.530 86.240 1.00 0.00

ATOM 4617 GL1 PIP 432 77.990 47.060 81.950 1.00 0.00

ATOM 4618 GL2 PIP 432 75.970 44.760 80.990 1.00 0.00

ATOM 4619 C1A PIP 432 75.310 43.320 76.510 1.00 0.00

ATOM 4620 C2A PIP 432 76.280 39.290 74.350 1.00 0.00

ATOM 4621 C3A PIP 432 78.810 37.710 70.560 1.00 0.00

ATOM 4622 C4A PIP 432 79.140 34.900 67.750 1.00 0.00

ATOM 4623 C1B PIP 432 76.830 49.530 78.980 1.00 0.00

ATOM 4624 C2B PIP 432 77.550 47.650 75.380 1.00 0.00

ATOM 4625 C3B PIP 432 81.080 48.630 73.100 1.00 0.00

ATOM 4626 C4B PIP 432 83.890 50.910 70.350 1.00 0.00

ATOM 4627 C5B PIP 432 86.490 50.190 66.660 1.00 0.00

ATOM 4628 PO1 PIP 433 75.750 10.550 89.030 1.00 0.00

ATOM 4629 PO2 PIP 433 76.700 9.530 83.930 1.00 0.00

ATOM 4630 RP1 PIP 433 75.550 12.060 86.470 1.00 0.00

ATOM 4631 RP2 PIP 433 77.930 12.950 84.830 1.00 0.00

ATOM 4632 RP3 PIP 433 76.010 14.940 85.400 1.00 0.00

ATOM 4633 PO3 PIP 433 74.430 16.820 84.510 1.00 0.00

ATOM 4634 GL1 PIP 433 76.310 19.790 79.720 1.00 0.00

ATOM 4635 GL2 PIP 433 78.650 18.370 78.430 1.00 0.00

ATOM 4636 C1A PIP 433 80.790 19.400 74.780 1.00 0.00

ATOM 4637 C2A PIP 433 77.970 21.850 71.100 1.00 0.00

ATOM 4638 C3A PIP 433 79.040 25.250 67.260 1.00 0.00

ATOM 4639 C4A PIP 433 76.550 26.270 64.330 1.00 0.00

ATOM 4640 C1B PIP 433 75.200 20.500 74.580 1.00 0.00

ATOM 4641 C2B PIP 433 73.250 23.740 72.130 1.00 0.00

ATOM 4642 C3B PIP 433 72.450 26.940 69.050 1.00 0.00

ATOM 4643 C4B PIP 433 69.460 28.100 66.320 1.00 0.00

ATOM 4644 C5B PIP 433 64.790 30.860 65.960 1.00 0.00

ATOM 4645 PO1 PIP 434 39.510 77.200 93.660 1.00 0.00

ATOM 4646 PO2 PIP 434 44.070 76.430 96.070 1.00 0.00

ATOM 4647 RP1 PIP 434 41.640 74.890 93.620 1.00 0.00

ATOM 4648 RP2 PIP 434 44.320 76.030 92.680 1.00 0.00

ATOM 4649 RP3 PIP 434 43.620 73.280 92.070 1.00 0.00

ATOM 4650 PO3 PIP 434 43.700 70.920 90.600 1.00 0.00

ATOM 4651 GL1 PIP 434 43.460 70.730 85.930 1.00 0.00

ATOM 4652 GL2 PIP 434 42.460 71.520 84.200 1.00 0.00

ATOM 4653 C1A PIP 434 41.980 73.310 79.560 1.00 0.00

ATOM 4654 C2A PIP 434 41.100 71.760 75.440 1.00 0.00

ATOM 4655 C3A PIP 434 37.490 71.750 71.500 1.00 0.00

ATOM 4656 C4A PIP 434 33.320 73.850 70.160 1.00 0.00

ATOM 4657 C1B PIP 434 44.150 66.770 84.010 1.00 0.00

ATOM 4658 C2B PIP 434 44.790 64.180 80.280 1.00 0.00

ATOM 4659 C3B PIP 434 43.620 61.490 77.200 1.00 0.00

ATOM 4660 C4B PIP 434 44.650 59.350 74.310 1.00 0.00

ATOM 4661 C5B PIP 434 46.990 56.420 72.790 1.00 0.00

ATOM 4662 PO1 PIP 435 53.650 73.340 97.500 1.00 0.00

ATOM 4663 PO2 PIP 435 50.320 77.190 96.780 1.00 0.00

ATOM 4664 RP1 PIP 435 51.690 74.130 95.820 1.00 0.00

ATOM 4665 RP2 PIP 435 48.930 75.050 95.040 1.00 0.00

ATOM 4666 RP3 PIP 435 50.380 74.230 92.870 1.00 0.00

ATOM 4667 PO3 PIP 435 49.350 73.510 90.310 1.00 0.00

ATOM 4668 GL1 PIP 435 48.080 70.820 87.610 1.00 0.00

ATOM 4669 GL2 PIP 435 47.990 68.100 87.890 1.00 0.00

ATOM 4670 C1A PIP 435 48.660 64.680 84.960 1.00 0.00

ATOM 4671 C2A PIP 435 49.990 62.570 80.780 1.00 0.00

ATOM 4672 C3A PIP 435 51.470 64.780 77.120 1.00 0.00

ATOM 4673 C4A PIP 435 54.850 64.650 75.650 1.00 0.00

ATOM 4674 C1B PIP 435 47.650 70.180 82.890 1.00 0.00

ATOM 4675 C2B PIP 435 47.170 68.100 79.260 1.00 0.00

ATOM 4676 C3B PIP 435 47.080 65.510 75.150 1.00 0.00

ATOM 4677 C4B PIP 435 45.120 63.630 71.360 1.00 0.00

ATOM 4678 C5B PIP 435 46.900 60.600 67.690 1.00 0.00

ATOM 4679 PO1 PIP 436 25.960 52.970 38.760 1.00 0.00

ATOM 4680 PO2 PIP 436 21.720 50.140 39.790 1.00 0.00

ATOM 4681 RP1 PIP 436 24.500 51.790 40.730 1.00 0.00

ATOM 4682 RP2 PIP 436 24.040 49.010 41.860 1.00 0.00

ATOM 4683 RP3 PIP 436 25.220 51.030 43.550 1.00 0.00

ATOM 4684 PO3 PIP 436 27.490 50.200 45.430 1.00 0.00

ATOM 4685 GL1 PIP 436 28.140 50.540 48.840 1.00 0.00

ATOM 4686 GL2 PIP 436 27.290 48.790 51.430 1.00 0.00

ATOM 4687 C1A PIP 436 28.530 49.080 55.130 1.00 0.00

ATOM 4688 C2A PIP 436 31.110 50.930 58.300 1.00 0.00

ATOM 4689 C3A PIP 436 32.130 49.720 61.470 1.00 0.00

ATOM 4690 C4A PIP 436 32.120 44.490 63.270 1.00 0.00

ATOM 4691 C1B PIP 436 31.240 52.980 52.510 1.00 0.00

ATOM 4692 C2B PIP 436 30.740 56.320 55.540 1.00 0.00

ATOM 4693 C3B PIP 436 29.900 57.260 60.110 1.00 0.00

ATOM 4694 C4B PIP 436 29.380 54.650 64.090 1.00 0.00

ATOM 4695 C5B PIP 436 28.200 50.350 66.040 1.00 0.00

ATOM 4696 PO1 PIP 437 46.510 37.940 40.030 1.00 0.00

ATOM 4697 PO2 PIP 437 42.090 38.260 37.780 1.00 0.00

ATOM 4698 RP1 PIP 437 43.910 39.190 40.260 1.00 0.00

ATOM 4699 RP2 PIP 437 43.000 41.490 38.150 1.00 0.00

ATOM 4700 RP3 PIP 437 43.180 41.950 40.940 1.00 0.00

ATOM 4701 PO3 PIP 437 41.690 42.670 43.520 1.00 0.00

ATOM 4702 GL1 PIP 437 40.210 39.990 47.280 1.00 0.00

ATOM 4703 GL2 PIP 437 38.690 41.450 48.290 1.00 0.00

ATOM 4704 C1A PIP 437 36.810 39.840 51.690 1.00 0.00

ATOM 4705 C2A PIP 437 34.720 41.660 55.670 1.00 0.00

ATOM 4706 C3A PIP 437 31.030 41.890 58.380 1.00 0.00

ATOM 4707 C4A PIP 437 29.800 45.800 58.910 1.00 0.00

ATOM 4708 C1B PIP 437 42.730 37.000 49.740 1.00 0.00

ATOM 4709 C2B PIP 437 40.630 33.670 51.700 1.00 0.00

ATOM 4710 C3B PIP 437 38.170 30.120 51.560 1.00 0.00

ATOM 4711 C4B PIP 437 35.070 26.860 51.590 1.00 0.00

ATOM 4712 C5B PIP 437 31.000 25.680 53.380 1.00 0.00

ATOM 4713 PO1 PIP 438 36.200 67.440 38.470 1.00 0.00

ATOM 4714 PO2 PIP 438 36.790 71.730 41.480 1.00 0.00

ATOM 4715 RP1 PIP 438 35.430 68.500 41.360 1.00 0.00

ATOM 4716 RP2 PIP 438 36.410 69.620 44.230 1.00 0.00

ATOM 4717 RP3 PIP 438 34.320 67.420 44.160 1.00 0.00

ATOM 4718 PO3 PIP 438 32.960 66.390 46.570 1.00 0.00

ATOM 4719 GL1 PIP 438 32.630 65.110 50.500 1.00 0.00

ATOM 4720 GL2 PIP 438 35.060 67.870 51.860 1.00 0.00

ATOM 4721 C1A PIP 438 36.410 69.360 55.330 1.00 0.00

ATOM 4722 C2A PIP 438 37.130 71.780 59.290 1.00 0.00

ATOM 4723 C3A PIP 438 37.070 75.660 60.980 1.00 0.00

ATOM 4724 C4A PIP 438 35.900 76.660 65.260 1.00 0.00

ATOM 4725 C1B PIP 438 32.580 65.580 55.020 1.00 0.00

ATOM 4726 C2B PIP 438 32.940 67.230 59.190 1.00 0.00

ATOM 4727 C3B PIP 438 35.640 68.850 62.880 1.00 0.00

ATOM 4728 C4B PIP 438 38.160 72.120 64.410 1.00 0.00

ATOM 4729 C5B PIP 438 40.550 73.930 67.340 1.00 0.00

ATOM 4730 PO1 PIP 439 52.250 44.810 39.410 1.00 0.00

ATOM 4731 PO2 PIP 439 46.830 45.910 38.260 1.00 0.00

ATOM 4732 RP1 PIP 439 49.400 44.300 40.390 1.00 0.00

ATOM 4733 RP2 PIP 439 47.210 42.900 39.340 1.00 0.00

ATOM 4734 RP3 PIP 439 47.920 42.400 42.140 1.00 0.00

ATOM 4735 PO3 PIP 439 46.580 42.290 44.380 1.00 0.00

ATOM 4736 GL1 PIP 439 43.920 42.840 47.870 1.00 0.00

ATOM 4737 GL2 PIP 439 42.150 45.500 48.870 1.00 0.00

ATOM 4738 C1A PIP 439 40.630 49.350 50.900 1.00 0.00

ATOM 4739 C2A PIP 439 41.060 51.480 55.100 1.00 0.00

ATOM 4740 C3A PIP 439 39.650 54.400 58.970 1.00 0.00

ATOM 4741 C4A PIP 439 38.170 56.790 61.840 1.00 0.00

ATOM 4742 C1B PIP 439 41.320 42.720 52.300 1.00 0.00

ATOM 4743 C2B PIP 439 39.900 38.850 55.290 1.00 0.00

ATOM 4744 C3B PIP 439 39.510 36.010 58.700 1.00 0.00

ATOM 4745 C4B PIP 439 38.180 35.300 63.440 1.00 0.00

ATOM 4746 C5B PIP 439 35.170 34.640 65.250 1.00 0.00

ATOM 4747 PO1 PIP 440 86.830 107.540 37.640 1.00 0.00

ATOM 4748 PO2 PIP 440 88.390 108.600 42.420 1.00 0.00

ATOM 4749 RP1 PIP 440 86.420 106.640 40.400 1.00 0.00

ATOM 4750 RP2 PIP 440 85.850 106.660 43.260 1.00 0.00

ATOM 4751 RP3 PIP 440 86.240 103.860 41.780 1.00 0.00

ATOM 4752 PO3 PIP 440 87.050 101.060 42.800 1.00 0.00

ATOM 4753 GL1 PIP 440 88.260 99.850 46.850 1.00 0.00

ATOM 4754 GL2 PIP 440 85.650 99.510 47.640 1.00 0.00

ATOM 4755 C1A PIP 440 85.440 101.340 51.520 1.00 0.00

ATOM 4756 C2A PIP 440 85.070 101.210 55.920 1.00 0.00

ATOM 4757 C3A PIP 440 85.470 101.450 60.570 1.00 0.00

ATOM 4758 C4A PIP 440 86.780 103.660 65.580 1.00 0.00

ATOM 4759 C1B PIP 440 90.140 98.050 48.790 1.00 0.00

ATOM 4760 C2B PIP 440 93.070 98.100 52.380 1.00 0.00

ATOM 4761 C3B PIP 440 93.660 96.870 56.670 1.00 0.00

ATOM 4762 C4B PIP 440 92.950 96.070 60.820 1.00 0.00

ATOM 4763 C5B PIP 440 94.650 98.640 64.330 1.00 0.00

ATOM 4764 PO1 PIP 441 85.890 43.810 39.850 1.00 0.00

ATOM 4765 PO2 PIP 441 80.850 43.340 40.960 1.00 0.00

ATOM 4766 RP1 PIP 441 83.930 45.210 41.900 1.00 0.00

ATOM 4767 RP2 PIP 441 81.060 45.890 42.380 1.00 0.00

ATOM 4768 RP3 PIP 441 82.990 46.110 44.830 1.00 0.00

ATOM 4769 PO3 PIP 441 82.870 47.560 47.460 1.00 0.00

ATOM 4770 GL1 PIP 441 81.750 46.870 52.180 1.00 0.00

ATOM 4771 GL2 PIP 441 82.230 44.010 54.590 1.00 0.00

ATOM 4772 C1A PIP 441 79.310 41.180 57.040 1.00 0.00

ATOM 4773 C2A PIP 441 76.090 40.020 60.690 1.00 0.00

ATOM 4774 C3A PIP 441 73.500 41.290 64.990 1.00 0.00

ATOM 4775 C4A PIP 441 71.280 44.350 67.210 1.00 0.00

ATOM 4776 C1B PIP 441 80.980 47.690 57.410 1.00 0.00

ATOM 4777 C2B PIP 441 82.130 46.400 60.930 1.00 0.00

ATOM 4778 C3B PIP 441 81.280 41.520 61.730 1.00 0.00

ATOM 4779 C4B PIP 441 84.330 38.120 61.830 1.00 0.00

ATOM 4780 C5B PIP 441 87.160 34.620 60.600 1.00 0.00

ATOM 4781 PO1 PIP 442 43.730 4.690 37.880 1.00 0.00

ATOM 4782 PO2 PIP 442 41.300 8.960 39.530 1.00 0.00

ATOM 4783 RP1 PIP 442 41.950 5.680 40.230 1.00 0.00

ATOM 4784 RP2 PIP 442 42.190 7.820 42.200 1.00 0.00

ATOM 4785 RP3 PIP 442 42.240 5.030 43.050 1.00 0.00

ATOM 4786 PO3 PIP 442 42.960 4.570 45.820 1.00 0.00

ATOM 4787 GL1 PIP 442 41.560 5.200 50.260 1.00 0.00

ATOM 4788 GL2 PIP 442 42.750 7.640 50.990 1.00 0.00

ATOM 4789 C1A PIP 442 44.420 9.940 54.430 1.00 0.00

ATOM 4790 C2A PIP 442 43.240 10.770 58.310 1.00 0.00

ATOM 4791 C3A PIP 442 41.730 11.760 61.820 1.00 0.00

ATOM 4792 C4A PIP 442 42.250 14.330 65.240 1.00 0.00

ATOM 4793 C1B PIP 442 39.930 6.620 54.440 1.00 0.00

ATOM 4794 C2B PIP 442 39.920 6.890 58.610 1.00 0.00

ATOM 4795 C3B PIP 442 41.170 6.630 62.440 1.00 0.00

ATOM 4796 C4B PIP 442 40.710 5.410 66.340 1.00 0.00

ATOM 4797 C5B PIP 442 40.760 2.870 70.940 1.00 0.00

ATOM 4798 PO1 PIP 443 6.890 68.990 43.800 1.00 0.00

ATOM 4799 PO2 PIP 443 5.160 74.310 42.170 1.00 0.00

ATOM 4800 RP1 PIP 443 7.190 72.010 44.350 1.00 0.00

ATOM 4801 RP2 PIP 443 5.300 74.000 45.230 1.00 0.00

ATOM 4802 RP3 PIP 443 8.080 74.110 46.400 1.00 0.00

ATOM 4803 PO3 PIP 443 9.920 75.460 48.350 1.00 0.00

ATOM 4804 GL1 PIP 443 10.630 74.430 51.880 1.00 0.00

ATOM 4805 GL2 PIP 443 8.720 73.160 53.950 1.00 0.00

ATOM 4806 C1A PIP 443 7.970 73.810 57.510 1.00 0.00

ATOM 4807 C2A PIP 443 10.370 74.340 60.800 1.00 0.00

ATOM 4808 C3A PIP 443 13.700 76.690 62.350 1.00 0.00

ATOM 4809 C4A PIP 443 16.750 76.290 66.090 1.00 0.00

ATOM 4810 C1B PIP 443 13.000 74.070 55.480 1.00 0.00

ATOM 4811 C2B PIP 443 15.940 73.530 59.550 1.00 0.00

ATOM 4812 C3B PIP 443 18.390 73.030 62.360 1.00 0.00

ATOM 4813 C4B PIP 443 21.570 73.610 66.370 1.00 0.00

ATOM 4814 C5B PIP 443 25.730 73.720 68.540 1.00 0.00

ATOM 4815 PO1 PIP 444 72.930 6.890 38.210 1.00 0.00

ATOM 4816 PO2 PIP 444 67.590 6.120 37.560 1.00 0.00

ATOM 4817 RP1 PIP 444 70.630 6.480 39.950 1.00 0.00

ATOM 4818 RP2 PIP 444 68.120 7.210 40.560 1.00 0.00

ATOM 4819 RP3 PIP 444 69.310 5.580 42.660 1.00 0.00

ATOM 4820 PO3 PIP 444 69.620 5.250 45.300 1.00 0.00

ATOM 4821 GL1 PIP 444 66.980 5.900 48.730 1.00 0.00

ATOM 4822 GL2 PIP 444 65.880 4.860 51.810 1.00 0.00

ATOM 4823 C1A PIP 444 66.630 1.630 54.880 1.00 0.00

ATOM 4824 C2A PIP 444 66.700 1.130 59.620 1.00 0.00

ATOM 4825 C3A PIP 444 66.650 2.250 63.130 1.00 0.00

ATOM 4826 C4A PIP 444 67.160 2.760 67.300 1.00 0.00

ATOM 4827 C1B PIP 444 69.250 8.690 52.930 1.00 0.00

ATOM 4828 C2B PIP 444 70.190 10.710 56.210 1.00 0.00

ATOM 4829 C3B PIP 444 68.900 9.180 60.610 1.00 0.00

ATOM 4830 C4B PIP 444 69.480 6.880 63.980 1.00 0.00

ATOM 4831 C5B PIP 444 72.150 3.900 66.570 1.00 0.00

ATOM 4832 PO0 PI3 445 21.670 91.720 94.460 1.00 0.00

ATOM 4833 PO1 PI3 445 28.370 89.770 93.960 1.00 0.00

ATOM 4834 PO2 PI3 445 24.360 87.090 94.720 1.00 0.00

ATOM 4835 RP1 PI3 445 25.710 88.930 92.590 1.00 0.00

ATOM 4836 RP2 PI3 445 22.810 89.470 92.610 1.00 0.00

ATOM 4837 RP3 PI3 445 24.070 88.610 90.140 1.00 0.00

ATOM 4838 PO3 PI3 445 24.460 88.680 87.310 1.00 0.00

ATOM 4839 GL1 PI3 445 23.670 86.400 83.130 1.00 0.00

ATOM 4840 GL2 PI3 445 25.440 85.500 80.950 1.00 0.00

ATOM 4841 C1A PI3 445 27.590 88.010 76.790 1.00 0.00

ATOM 4842 C2A PI3 445 28.390 88.160 72.660 1.00 0.00

ATOM 4843 C3A PI3 445 31.950 87.490 70.740 1.00 0.00

ATOM 4844 C4A PI3 445 34.960 89.320 67.870 1.00 0.00

ATOM 4845 C1B PI3 445 20.400 87.230 80.330 1.00 0.00

ATOM 4846 C2B PI3 445 17.000 84.040 78.210 1.00 0.00

ATOM 4847 C3B PI3 445 16.420 79.510 77.810 1.00 0.00

ATOM 4848 C4B PI3 445 19.150 75.920 77.970 1.00 0.00

ATOM 4849 C5B PI3 445 20.670 72.690 79.690 1.00 0.00

ATOM 4850 PO0 PI3 446 11.600 49.550 97.920 1.00 0.00

ATOM 4851 PO1 PI3 446 6.080 44.290 94.830 1.00 0.00

ATOM 4852 PO2 PI3 446 6.260 50.340 94.720 1.00 0.00

ATOM 4853 RP1 PI3 446 7.830 46.980 94.320 1.00 0.00

ATOM 4854 RP2 PI3 446 9.730 48.950 95.380 1.00 0.00

ATOM 4855 RP3 PI3 446 10.170 47.910 92.570 1.00 0.00

ATOM 4856 PO3 PI3 446 11.250 47.550 90.220 1.00 0.00

ATOM 4857 GL1 PI3 446 13.920 49.020 86.900 1.00 0.00

ATOM 4858 GL2 PI3 446 14.220 47.350 84.350 1.00 0.00

ATOM 4859 C1A PI3 446 16.200 47.800 80.760 1.00 0.00

ATOM 4860 C2A PI3 446 19.290 45.710 78.840 1.00 0.00

ATOM 4861 C3A PI3 446 20.000 44.460 74.100 1.00 0.00

ATOM 4862 C4A PI3 446 18.320 42.910 70.620 1.00 0.00

ATOM 4863 C1B PI3 446 16.120 51.840 84.940 1.00 0.00

ATOM 4864 C2B PI3 446 18.720 52.730 80.330 1.00 0.00

ATOM 4865 C3B PI3 446 18.400 49.440 76.410 1.00 0.00

ATOM 4866 C4B PI3 446 19.640 48.220 71.160 1.00 0.00

ATOM 4867 C5B PI3 446 18.850 47.950 67.050 1.00 0.00

ATOM 4868 PO0 PI3 447 56.120 48.980 91.270 1.00 0.00

ATOM 4869 PO1 PI3 447 63.610 48.820 94.850 1.00 0.00

ATOM 4870 PO2 PI3 447 60.590 51.300 91.510 1.00 0.00

ATOM 4871 RP1 PI3 447 61.320 48.260 92.950 1.00 0.00

ATOM 4872 RP2 PI3 447 58.610 48.350 91.550 1.00 0.00

ATOM 4873 RP3 PI3 447 60.700 46.530 90.440 1.00 0.00

ATOM 4874 PO3 PI3 447 61.440 43.920 89.150 1.00 0.00

ATOM 4875 GL1 PI3 447 64.150 42.110 86.350 1.00 0.00

ATOM 4876 GL2 PI3 447 66.430 39.770 86.820 1.00 0.00

ATOM 4877 C1A PI3 447 68.420 36.360 83.600 1.00 0.00

ATOM 4878 C2A PI3 447 68.050 37.230 79.050 1.00 0.00

ATOM 4879 C3A PI3 447 68.700 35.590 75.080 1.00 0.00

ATOM 4880 C4A PI3 447 68.110 35.480 70.870 1.00 0.00

ATOM 4881 C1B PI3 447 66.620 41.320 82.060 1.00 0.00

ATOM 4882 C2B PI3 447 70.060 41.510 78.330 1.00 0.00

ATOM 4883 C3B PI3 447 71.690 41.670 72.720 1.00 0.00

ATOM 4884 C4B PI3 447 75.610 41.800 69.470 1.00 0.00

ATOM 4885 C5B PI3 447 77.940 43.660 66.380 1.00 0.00

ATOM 4886 PO0 PI3 448 46.980 2.990 91.460 1.00 0.00

ATOM 4887 PO1 PI3 448 52.540 -1.640 90.890 1.00 0.00

ATOM 4888 PO2 PI3 448 52.230 3.850 90.590 1.00 0.00

ATOM 4889 RP1 PI3 448 51.320 0.800 89.810 1.00 0.00

ATOM 4890 RP2 PI3 448 49.000 2.730 89.690 1.00 0.00

ATOM 4891 RP3 PI3 448 49.900 1.170 87.420 1.00 0.00

ATOM 4892 PO3 PI3 448 49.310 1.140 84.700 1.00 0.00

ATOM 4893 GL1 PI3 448 50.950 1.840 80.900 1.00 0.00

ATOM 4894 GL2 PI3 448 50.770 4.880 81.010 1.00 0.00

ATOM 4895 C1A PI3 448 50.890 6.870 76.940 1.00 0.00

ATOM 4896 C2A PI3 448 49.840 8.660 72.930 1.00 0.00

ATOM 4897 C3A PI3 448 48.180 9.680 69.510 1.00 0.00

ATOM 4898 C4A PI3 448 45.350 12.650 68.400 1.00 0.00

ATOM 4899 C1B PI3 448 52.560 1.740 76.470 1.00 0.00

ATOM 4900 C2B PI3 448 53.740 4.860 73.280 1.00 0.00

ATOM 4901 C3B PI3 448 52.140 5.280 69.750 1.00 0.00

ATOM 4902 C4B PI3 448 50.410 1.680 66.330 1.00 0.00

ATOM 4903 C5B PI3 448 47.560 -0.770 64.820 1.00 0.00

ATOM 4904 PO0 PI3 449 16.070 12.510 36.140 1.00 0.00

ATOM 4905 PO1 PI3 449 11.930 7.970 34.830 1.00 0.00

ATOM 4906 PO2 PI3 449 17.280 7.170 35.860 1.00 0.00

ATOM 4907 RP1 PI3 449 13.830 7.250 37.090 1.00 0.00

ATOM 4908 RP2 PI3 449 15.830 9.430 37.360 1.00 0.00

ATOM 4909 RP3 PI3 449 14.400 8.390 39.830 1.00 0.00

ATOM 4910 PO3 PI3 449 13.710 8.060 42.310 1.00 0.00

ATOM 4911 GL1 PI3 449 14.260 8.520 46.700 1.00 0.00

ATOM 4912 GL2 PI3 449 13.640 6.520 49.510 1.00 0.00

ATOM 4913 C1A PI3 449 13.210 3.690 52.360 1.00 0.00

ATOM 4914 C2A PI3 449 15.540 1.750 56.670 1.00 0.00

ATOM 4915 C3A PI3 449 16.550 -0.920 60.090 1.00 0.00

ATOM 4916 C4A PI3 449 15.510 -5.550 60.050 1.00 0.00

ATOM 4917 C1B PI3 449 17.270 9.430 50.140 1.00 0.00

ATOM 4918 C2B PI3 449 18.020 7.500 53.670 1.00 0.00

ATOM 4919 C3B PI3 449 17.210 5.990 57.680 1.00 0.00

ATOM 4920 C4B PI3 449 15.990 4.240 61.250 1.00 0.00

ATOM 4921 C5B PI3 449 14.440 2.000 63.880 1.00 0.00

ATOM 4922 PO0 PI3 450 11.660 84.690 39.340 1.00 0.00

ATOM 4923 PO1 PI3 450 5.540 82.790 38.010 1.00 0.00

ATOM 4924 PO2 PI3 450 9.820 80.080 38.950 1.00 0.00

ATOM 4925 RP1 PI3 450 7.310 82.170 40.030 1.00 0.00

ATOM 4926 RP2 PI3 450 10.030 82.920 41.050 1.00 0.00

ATOM 4927 RP3 PI3 450 7.420 82.990 42.880 1.00 0.00

ATOM 4928 PO3 PI3 450 6.670 83.070 45.680 1.00 0.00

ATOM 4929 GL1 PI3 450 7.390 83.880 50.170 1.00 0.00

ATOM 4930 GL2 PI3 450 5.920 86.140 49.950 1.00 0.00

ATOM 4931 C1A PI3 450 3.650 89.590 50.430 1.00 0.00

ATOM 4932 C2A PI3 450 -0.850 91.110 51.060 1.00 0.00

ATOM 4933 C3A PI3 450 -3.040 91.420 55.360 1.00 0.00

ATOM 4934 C4A PI3 450 -4.390 89.900 59.510 1.00 0.00

ATOM 4935 C1B PI3 450 8.030 84.020 54.050 1.00 0.00

ATOM 4936 C2B PI3 450 6.180 84.970 57.290 1.00 0.00

ATOM 4937 C3B PI3 450 8.930 85.920 62.320 1.00 0.00

ATOM 4938 C4B PI3 450 11.420 87.310 66.790 1.00 0.00

ATOM 4939 C5B PI3 450 14.020 85.090 69.700 1.00 0.00

ATOM 4940 PO0 PI3 451 102.090 3.860 38.610 1.00 0.00

ATOM 4941 PO1 PI3 451 94.570 4.130 38.870 1.00 0.00

ATOM 4942 PO2 PI3 451 97.430 -0.640 38.150 1.00 0.00

ATOM 4943 RP1 PI3 451 96.500 2.280 39.910 1.00 0.00

ATOM 4944 RP2 PI3 451 99.400 2.340 39.850 1.00 0.00

ATOM 4945 RP3 PI3 451 97.870 2.300 42.520 1.00 0.00

ATOM 4946 PO3 PI3 451 97.970 1.810 45.300 1.00 0.00

ATOM 4947 GL1 PI3 451 99.750 4.370 48.440 1.00 0.00

ATOM 4948 GL2 PI3 451 97.620 4.870 50.420 1.00 0.00

ATOM 4949 C1A PI3 451 98.550 4.410 54.800 1.00 0.00

ATOM 4950 C2A PI3 451 99.320 4.410 59.360 1.00 0.00

ATOM 4951 C3A PI3 451 100.390 2.270 63.330 1.00 0.00

ATOM 4952 C4A PI3 451 97.980 1.410 66.870 1.00 0.00

ATOM 4953 C1B PI3 451 101.440 7.070 52.440 1.00 0.00

ATOM 4954 C2B PI3 451 102.060 9.030 56.850 1.00 0.00

ATOM 4955 C3B PI3 451 102.190 10.840 60.590 1.00 0.00

ATOM 4956 C4B PI3 451 102.110 13.270 64.950 1.00 0.00

ATOM 4957 C5B PI3 451 101.900 16.960 67.240 1.00 0.00

ATOM 4958 PO0 PI3 452 67.840 21.040 34.550 1.00 0.00

ATOM 4959 PO1 PI3 452 60.660 19.110 36.110 1.00 0.00

ATOM 4960 PO2 PI3 452 66.250 17.060 37.610 1.00 0.00

ATOM 4961 RP1 PI3 452 63.180 18.940 37.880 1.00 0.00

ATOM 4962 RP2 PI3 452 65.700 20.310 36.790 1.00 0.00

ATOM 4963 RP3 PI3 452 64.690 21.420 38.950 1.00 0.00

ATOM 4964 PO3 PI3 452 65.000 22.980 41.340 1.00 0.00

ATOM 4965 GL1 PI3 452 64.990 22.800 45.900 1.00 0.00

ATOM 4966 GL2 PI3 452 68.200 24.740 47.780 1.00 0.00

ATOM 4967 C1A PI3 452 70.300 23.890 51.660 1.00 0.00

ATOM 4968 C2A PI3 452 71.860 21.920 55.190 1.00 0.00

ATOM 4969 C3A PI3 452 76.330 21.780 57.440 1.00 0.00

ATOM 4970 C4A PI3 452 77.780 22.120 60.840 1.00 0.00

ATOM 4971 C1B PI3 452 63.200 20.330 50.260 1.00 0.00

ATOM 4972 C2B PI3 452 63.300 19.980 54.800 1.00 0.00

ATOM 4973 C3B PI3 452 62.720 18.910 58.740 1.00 0.00

ATOM 4974 C4B PI3 452 60.800 20.120 61.970 1.00 0.00

ATOM 4975 C5B PI3 452 59.960 19.500 66.210 1.00 0.00

TER

ENDMDL
